# Intermolecular [3+3] ring expansion of aziridines to dehydropiperi-dines through the intermediacy of aziridinium ylides

**DOI:** 10.1038/s41467-020-15134-x

**Published:** 2020-03-09

**Authors:** Josephine Eshon, Kate A. Nicastri, Steven C. Schmid, William T. Raskopf, Ilia A. Guzei, Israel Fernández, Jennifer M. Schomaker

**Affiliations:** 1 Department of Chemistry, University of Wisconsin, 1101 University Avenue, Madison, WI 53706 USA; 2 Departamento de Química Orgánica I and Centro de Innovación en Química Avazanda (ORFEO-CINQA), Facultad de Ciencias Químicas, Universidad Complutense de Madrid, 28040 Madrid, Spain

**Keywords:** Homogeneous catalysis, Reaction mechanisms, Stereochemistry, Synthetic chemistry methodology

## Abstract

The importance of *N-*heterocycles in drugs has stimulated diverse methods for their efficient syntheses. Methods that introduce significant stereochemical complexity are attractive for identifying new bioactive amine chemical space. Here, we report a [3 + 3] ring expansion of bicyclic aziridines and rhodium-bound vinyl carbenes to form complex dehydropiperidines in a highly stereocontrolled rearrangement. Mechanistic studies and DFT computations indicate that the reaction proceeds through formation of a vinyl aziridinium ylide; this reactive intermediate undergoes a pseudo-[1,4]-sigmatropic rearrangement to directly furnish heterocyclic products with net retention at the new C-C bond. In combination with asymmetric silver-catalyzed aziridination, enantioenriched scaffolds with up to three contiguous stereocenters are rapidly delivered. The mild reaction conditions, functional group tolerance, and high stereospecificity of this method are well-suited for appending piperidine motifs to natural product and complex molecules. Ultimately, our work establishes the value of underutilized aziridinium ylides as key intermediates for converting small, strained rings to larger *N-*heterocycles.

## Abstract (web summary)

Traditional synthesis of stereodefined piperidines requires selective installation of functional groups that can lower efficiency and modularity. Here, the authors assemble stereochemically complex and highly substituted dehydropiperidines via an intermolecular ring expansion between bicyclic aziridines and Rh-supported vinyl carbenes.

## Introduction

The importance of nitrogenated heterocycles in pharmaceuticals, natural products, and fine chemicals continues to drive innovative strategies for their efficient syntheses from readily available precursors^1–14^. The ability to improve upon existing preparations of known compounds, enable alternate retrosynthetic approaches to useful building blocks, and increase opportunities to explore novel chemical space outside of ‘flatland’ are all compelling reasons to develop new approaches to *N*-heterocycles^15–20^. Piperidines rank as the most prominent *N*-heterocyclic pharmacophore in current drugs on the market, appearing in ~55% of all FDA-approved drugs containing at least one *N-*heterocycle, as well as in numerous bioactive natural products^3^. Convergent methods that unite multiple reactive fragments, particularly hetero-[4+2] cycloadditions, deliver substituted dehydropiperidines in enantioenriched form^21–31^; however, critical substrate and/or catalyst control over regio- and stereoselectivity is challenging and often results in narrow scope. Traditional preparations of stereodefined piperidines using intramolecular S_N_2-type reactions require selective installation of functional groups prior to ring closure, resulting in lower efficiency, modularity, and step economy as the desired target’s complexity increases^3^. A less common approach is to engage an aziridine and a simple coupling partner in a stereocontrolled, and ideally stereospecific, ring expansion reaction.

**Fig. 1:**
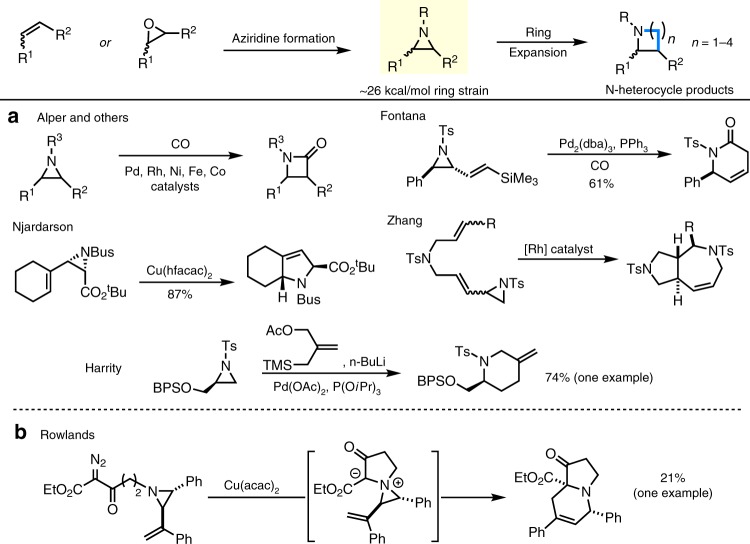
Transformations of aziridines to *N*-heterocycles. **a** Typical transition metal-catalyzed ring expansions. **b** Cu-catalyzed aziridine ring expansion through the intermediacy of an aziridinium ylide.

Aziridines are an ideal starting material for conversion into larger nitrogenated heterocycles. They are easily accessible from a variety of alkenes by nitrene transfer or from simple manipulations of epoxides. In addition, methods for asymmetric aziridination enable these strained rings to be prepared with substantial stereochemical and substitutional complexity^32–36^. An attractive feature of aziridines is their ~26 kcal mol^−1^ of ring strain, ensuring a favorable thermodynamic driving force for ring-opening. Figure 1 illustrates a selection of transition metal-catalyzed aziridine expansions that furnish 4–7 membered *N*-heterocycles^37–48^. For example, Alper and others have achieved metal-catalyzed carbonylations of aziridines to yield valuable β-lactams, although good regioselectivity depends on the substitution pattern of the three-membered ring^45^. Njardarson has described a series of Cu-catalyzed transformations of aziridines to pyrrolidines and related rings; while these reactions are often stereospecific, they are largely limited to intramolecular examples^40–42^. Scattered examples describing the conversion of aziridines to piperidines, dehydropiperidines, and azepines (Fig. 1a) are known, but these reactions are also intramolecular or have limited scope^44,46,47^. In contrast, we were encouraged by Rowlands report of a single example of the formation of a dehydropiperidine in 21% yield (Fig. 1b) from a vinylaziridine^49^. This reaction presumably occurs through intramolecular formation of an aziridinium ylide, followed by [2,3]-rearrangement; however, only one invertomer undergoes the desired cyclization. The low yield is ascribed to the required coplanarity of the anion and the vinyl group; if the ylide forms with the opposite stereochemistry at nitrogen, [1,5]-hydrogen shifts and other decomposition^50^ pathways compete with productive ring expansion.

As noted in Fig. 1, aziridines must often be highly engineered to achieve effective ring expansions. In particular, inclusion of an adjacent vinyl group provides the kinetic impetus to drive successful metal-mediated isomerizations and other functionalizations^39–41,44,46,47^. In contrast, utilizing unbiased aziridines for stereocontrolled expansions to larger *N*-heterocycles can be challenging, as epimerization or racemization of the aziridine must be avoided to successfully relay stereochemical information at *sp*^3^ stereocenters to the product with excellent fidelity^51–54^. Despite these difficulties, the ease of aziridine preparation and their strain-loaded reactivity make them attractive scaffolds for the discovery of new reactivity^32–36^.

**Fig. 2:**
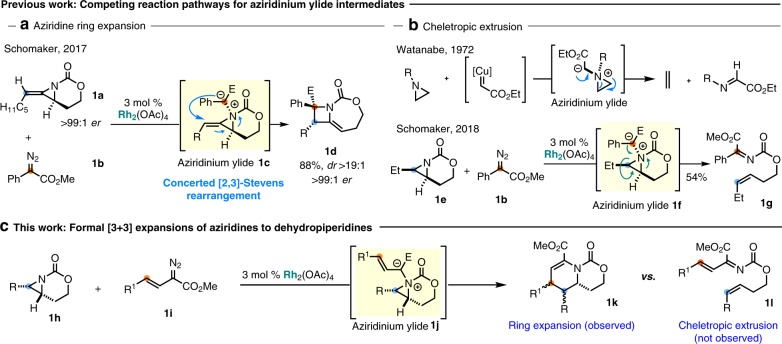
Reaction design. **a** Methyleneazetidines from ring expansion of aziridines. **b** Competing cheletropic extrusion pathways. **c** Favoring ring expansion over cheletropic extrusion for the synthesis of dehydropiperidines.

We previously leveraged the unusual strain (~42 kcal/mol) in methyleneaziridine **1a** to achieve a formal [3+1] reaction to furnish methyleneazetidine **1d** upon exposure to rhodium-supported carbene **1b** (Fig. 2a)^55–62^. Mechanistic studies support initial formation of the aziridinium ylide **1c**, which subsequently undergoes a highly asynchronous, concerted [2,3]-Stevens rearrangement to form **1d**^63^. The complete transfer of the chirality in **1a**–**d** provides further experimental evidence to support this mechanism. This efficient transformation, which forms new C–C and C–N bonds and two adjacent stereocenters in a stereospecific intermolecular two-fragment coupling, prompted us to undertake further studies of aziridinium ylides to extend the scope to unbiased aziridines. Unfortunately, removing the exocyclic alkene of **1a** in **1e** (Fig. 2b) gave only cheletropic extrusion with **1b** to furnish **1g**, a pathway also observed by Watanabe in Cu-catalyzed reaction of aziridines with ethyldiazoacetate^50^. We reasoned appending a vinyl group to the carbene precursor **1i** (Fig. 2c) could facilitate the desired ring expansion of aziridinium ylide **1j–k** over competing cheletropic extrusion to **1l**. This transformation represents a net [3+3] annulation of a vinyl carbenoid and a bicyclic aziridine; depending on the mechanism, transfer of stereochemical information from the aziridine to the dehydropiperidine with good fidelity could be envisaged. Herein, we report an attractive strategy to assemble stereochemically complex and highly substituted dehydropiperidines via an intermolecular ring expansion between simple bicyclic aziridines and Rh-supported vinyl carbenes. The intermediacy of an unusual aziridinium ylide species is followed by a stereospecific rearrangement that secures access to enantioenriched products.

## Results

### Scope of the [3+3] ring expansion

To our delight, reaction of *cis*-alkene-derived aziridine **2a** (Table 1) with Davies’ styrenyl diazoacetate^64^ **3a** under dirhodium catalysis produced **4aa** in 75% yield and in excellent >19:1 diastereoselectivity. Optimization of conditions for the [3+3] aziridine ring expansion revealed the dirhodium paddlewheel complex Rh_2_(OAc)_4_ was the superior catalyst using slow addition of the vinyl diazoacetate (see the Supplementary Information for further details). With these conditions in hand, the aziridine scope was further explored using **3a** as the vinyl carbene precursor. Linear alkyl groups on aziridines **2a–d**, including benzyl, methyl, ethyl, and *n*-butyl, gave good yields of the products **4aa–da** in high *dr*. ^1^H NMR spectroscopy indicated a *dr* of at least >19:1 for the dehydropiperidines, with only trace amounts of the presumed diastereomers noted. Increasing the bulk of the substituent on the aziridine to an isopropyl group in **2e** furnished **4ea** in 71% yield and excellent *dr*. Alkyl chloride and ether functionalities were also well tolerated to deliver dehydropiperidines **4fa** and **4ga**. Alkyl substitution *α* to the carbamate tether in **2h** (*dr* > 19:1) gave a 74% yield of **4ha** as a single diastereomer. Aziridine **2i**, unsubstituted at the terminal carbon, gave **4ia** in 67% yield and >19:1 *dr*. Finally, it was not necessary to have the carbamate contained in a six-membered ring, as the [5.3]-bicyclic aziridine **2j** (R^1^ = Et, *n* = 0) gave the [5.6]-bicyclic ring **4ja** in 42% yield and in >19:1 *dr*.

**Table 1:** Scope of the aziridine and diazoester in Rh-catalyzed ring expansions to dehydropiperidines.

^a^Conditions: 3 mol% Rh_2_(OAc)_4_, 0.05 M CH_2_Cl_2_, rt, slow addition of diazoacetate as a solution in CH_2_Cl_2_.

^b^NMR yield.

We next examined the scope of the carbene precursor using ethyl-substituted aziridine **2c** (Table 1). The impact of the electronics of a series of phenyl-substituted diazo acetates **3a–f** was investigated first. Similar yields were obtained for **4ca** and **4cb–cc**, irrespective of whether the diazoester carbene precursor contains electron-donating or neutral substituents, highlighting there is little effect of the styrene electronics on the reaction outcome. A single-crystal X-ray structure of **4cc** established the relative stereochemical configuration of the heterocycle product (see expansion for **4cc** and the Supplementary Information for further details), corroborating nOe studies. Moving the Br to the *meta* position in **3d** resulted in a similar 79% yield of **4cd**. Diazoester **3e**, bearing a strongly electron-withdrawing trifluoromethyl group, gave a 69% yield of **4ce**, also in good *dr*, providing a convenient way to introduce valuable fluorines into the dehydropiperidine products.

Carbene transfer of the naphthyl-substituted **3f** provided **4cf** in similar yield and *dr* as compared with **4ca**. It was not necessary to employ a styrenyl-derived diazoester, as a series of β-alkyl-substituted diazoesters **3g–i** all resulted in good yields of unsaturated piperidines **4cg**–**ci** as single diastereomers as determined by ^1^H NMR. The furan-substituted **3j** gave **4cj** in only 14% isolated yield, as these oxygen heterocycles have been reported to be reactive in the presence of metal-supported carbenoids^65,66^. Bulking up the methyl ester on the diazoester to a cyclohexyl ester was also successful, producing **4ck** in 56% yield and >19:1 *dr*.

### Computational studies of the mechanism

**Fig. 3:**
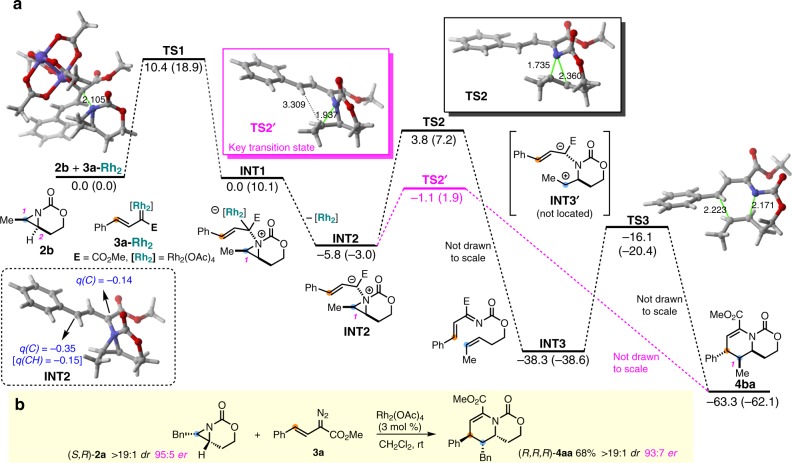
Mechanistic studies of the [3+3] ring expansion. **a** Computed reaction profile for the process involving **2b** and **Rh**_**2**_-bound carbene **3a-Rh**_**2**_. Relative free energies (ΔG, computed at 298.15 K and 1 M) and bond distances are in kcal/mol and Å, respectively. All data are computed at the SMD(CH_2_Cl_2_)-B3LYP-D3/def2-SVP level. Values within parentheses are computed at the SMD(CH_2_Cl_2_)-B3LYP-D3/def2-TZVPP//SMD(CH_2_Cl_2_)-B3LYP-D3/def2-SVP level of theory. **b** Stereochemical retention experiment.

The difficulty of studying carbene transfer using traditional kinetics, particularly when slow addition is required, led us to turn to DFT calculations (computational details are in the Supplementary Information and Supplementary Data 1) to gain more insight into the mechanism of the ring expansion (Fig. 3). The reaction of the methyl-substituted aziridine **2b** and the [Rh_2_]-carbenoid derived from diazoacetate **3a** to furnish **4ba** was explored computationally. Nucleophilic attack of the aziridine nitrogen atom on the carbon of the Rh-supported carbenoid occurs as the first step to form aziridinium ylide **INT1** via transition state **TS1**^62,63^. Then, barrierless and exergonic dissociation of the dirhodim catalyst from the nitrogen produces zwitterion **INT2**, where the negative charge is fully delocalized into the allylic system (Fig. 3b).

Two fates were envisaged for **INT2**. In the first case, **INT2** forms azadiene **INT3** through a cheletropic extrusion (via **TS2**), followed by a concerted aza-Diels–Alder cycloaddition (via **TS3**) to furnish **4ba**. However, subjecting enantioenriched (*S,R*)*-***2a**^67^ to the standard reaction conditions resulted in transfer of the chirality to (*R,R,R*)-**4aa** with good fidelity (Fig. 3b). This effectively rules out cheletropic extrusion, followed by aza-Diels–Alder reaction, as the operative mechanism for formation of the dehydropiperidine. We also considered that Stevens rearrangements involving ammonium ylides are occasionally reported to proceed through diradical intermediates^68^; however, the chirality transfer from (*S,R*)-**2a** to (*R,R,R*)-**4aa** suggests that a diradical pathway cannot be operative in the absence of a solvent cage.

An alternate fate for the aziridinium ylide **INT2** is suggested by the computations in Scheme 3 and involves a rare pseudo-[1,4]-sigmatropic rearrangement of **INT2** via **TS2′**. This pathway has never been observed for an aziridinium ylide; however, a single example of a [1,4]-rearrangement of an ammonium benzylide has been reported^69,70^. According to our calculations, this path proceeds with a lower barrier than the competing cheletropic extrusion (∆∆G^≠^ = 5.3 kcal/mol). In addition, this mechanism would be expected to directly parlay absolute and relative stereochemical information from the aziridine into the product, supporting our experimental observations of chirality transfer from (*S,R*)-**2a** to (*R,R,R*)-**4aa**^62,63^. Zwitterion **INT3′** could not be located on the potential energy surface. Indeed, intrinsic reaction calculations (IRCs) starting from **TS2′** show the final closing of the C–C bond to form the piperidine exists on a plateau-like energy pathway, where ring closure only begins to occur following completion of the C–N bond rupture.

The retention of stereochemistry at the internal aziridine carbon C1 of **2b** in **4ba** provided an interesting clue to the exact nature of the rearrangement of **INT2** to **4ba**. One possibility involves intramolecular S_N_2 attack of the benzylic carbon on C1 of **INT2** (Fig. 3a) to close the ring. However, it is unlikely a π orbital on the benzylic carbon can overlap effectively with the σ* of the aziridinium ylide to enable S_N_2. More importantly, S_N_2 attack at C1 would invert the stereochemistry at C1, which is ruled out by the X-ray crystal structure of **4cc**. Rather, we propose ring expansion occurs via an unusual stereoretentive nucleophilic substitution; examples of this rare mechanism include the chlorination of secondary alcohols with thionyl chloride (S_N_i) and benzylic substitution in supramolecular cavities^71,72^. Computations also support a stereoretentive ‘S_N_1-like’ mechanism. First, the lower energy **TS2′** is comprised mainly of C–N bond breakage at the external C1–N bond, which elongates to 1.937 Å. This contrasts to the bond-breaking sequence in the disfavored **TS2**, where the internal bicyclic C2–N bond shows more elongation at 2.360 Å, as compared with the C1–N bond (1.735 Å). Thus, even though both **TS2** and **TS2′** can be described as relatively low-barrier, early transition states, the extent to which the C–N bond breaks appears biased. Second, and consistent with our experimental results, **TS2′** predicts that ring opening of the C1–N aziridine bond and the subsequent C–C bond formation must proceed with retention. This is largely due to the stereochemical relationship established between the nitrogen and the carbon substituents of the aziridine during formation of the aziridinium ylide; the carbamate tether controls this stereochemistry and likely plays a key role in restricting the conformational flexibility of the intermediates. Thus, the Me-bearing C1 of the original aziridine **2b** is essentially a full carbocation, with nearly complete C–N rupture in **TS2′** just prior to stereoretentive ring closure.

### Chirality transfer

**Fig. 4:**
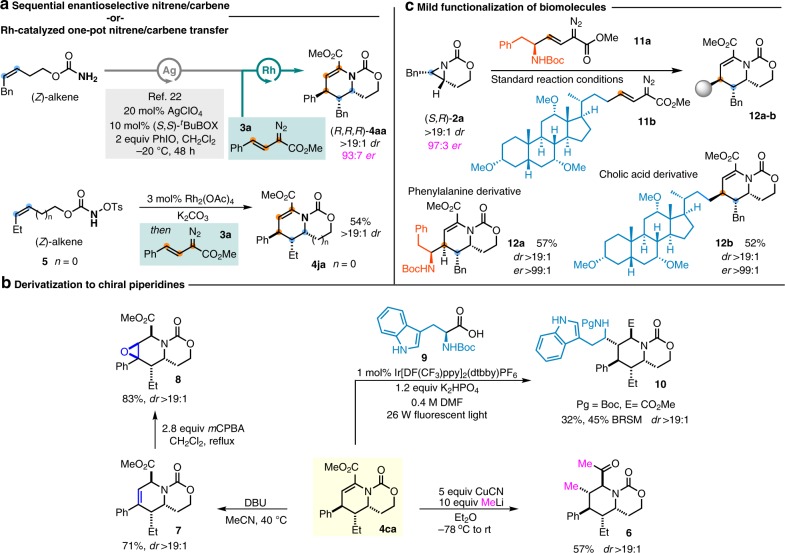
Tandem nitrene/carbene chemistry and derivatization of products. **a** Streamlining the nitrene/carbene transfer sequence. **b** Late-stage functionalization of complex molecules. **c** Further derivatization of dehydropiperidines.

The synthetic utility of the sequential nitrene/carbene transfer reaction could be amplified by running it in tandem with asymmetric alkene aziridination to form enantioenriched piperidines over two steps. In 2017, we disclosed an asymmetric aziridination protocol using silver bisoxazoline complexes to enact intramolecular aziridination of homoallylic carbamate esters to achieve high enantioselectivities with a cost-effective catalyst^67^. Application of our asymmetric aziridination to the carbamate of (3Z)-3-penten-5-phenyl-1-ol gave enantioenriched (*S,R*)*-***2a**^67^ in 95:5 *er* (Figs. 3b and 4a). Treatment of (*S,R*)*-***2a** under the standard reaction conditions gives (*R,R,R*)-**4aa** with minimal loss of *ee*. Alternatively, a single catalyst could be used to accomplish the sequential nitrene/carbene transfer. Preparation of the allylic *N*-tosyloxycarbamate ester **5**, according to the method described by Lebel et al.^73^, followed by treatment with Rh_2_(OAc)_4_ and K_2_CO_3_ generated the intermediate [5.3]-bicyclic aziridine. Filtration and solvent exchange, followed by slow addition of **3a**, gave the unsaturated piperidine **4ja** in 54% and >19:1 *dr*. Efforts to identify chemo- and enantioselective Rh-catalyzed aziridination catalysts are underway to secure access to these valuable heterocycles in one pot with good *ee*.

### Functionalization of dehydropiperidine products

Further functionalization of the unsaturated piperidine **4ca** provides fully substituted, stereochemically rich piperidines in just two steps from the bicyclic aziridine. Indeed, treatment of **4ca** with a higher order cuprate furnished **6** in 57% yield and >19:1 *dr* through a diastereoselective conjugate addition reaction; the relative stereochemistry was verified by both NOE and ^1^H NMR-coupling constants (Fig. 4b, see the Supplementary Information for details)^74–76^. In an initial effort to expand the conjugate addition reaction to include other heteroatom nucleophiles, we found treatment of **4ca** with DBU and various nucleophiles did not furnish a conjugate addition product, but rather produced styrene **7** in 71% yield and excellent *dr* (Fig. 4b). Further treatment of **7** with *m*CPBA yielded epoxide **8** in equally good yield and *dr*. Furthermore, inspired by MacMillan and coworkers^77^, **4ca** was found to undergo a radical Michael addition with Boc-protected tryptophan **9** to yield fully elaborated piperidine **10** in 32% yield as a single diastereomer. Although the yield was modest, this transformation rapidly builds complexity in two steps from a simple aziridine.

Finally, the mild reaction conditions and the transfer of the stereochemical information in the aziridine (*S,R*)-**2a** to (*R,R,R*)-**4aa** with good fidelity promoted us to explore the potential of this chemistry to append biomolecules to our unsaturated piperidine scaffolds. d-Phenylalanine and cholic acid were transformed into suitable diazoesters **11a** and **11b** (Fig. 4c), then subjected to treatment with (*S*,*R*)-**2a** (94% *ee*) under the standard reaction conditions. The products **12a** and **12b** were obtained in good yields, with excellent diastereoselectivities and enantiomeric ratios. We envisage this strategy could be effectively applied to explorations of new bioactive chemical space uncovered through fragment-based screening approaches.

In conclusion, aziridinium ylides, accessed in high diastereoselectivity from the intermolecular reaction of simple aziridines with metal-bound vinyl carbenes, are shown to be efficient intermediates for the conversion of small ring heterocycles to complex piperidines, a privileged motif in bioactive compounds. DFT computations, in tandem with transfer of chirality experiments, revealed that the ylides undergo a concerted, asynchronous, pseudo-[1,4]-sigmatropic rearrangement to yield products in high diastereoselectivity and with retention of *ee* installed in the aziridine precursor. Surprisingly, this chemistry bypasses deleterious cheletropic extrusion using unbiased aziridines to give synthetically useful yields of *N-*heterocycles. In addition, this mechanism proceeds with retention at the C–C bond, a unique consequence of the S_N_1-like closing of the vinyl anion tether. We anticipate this report will spur further research into the reactivity of both aziridinium ylides and other onium ylides derived from small-ring heterocycles.

## Methods

### General procedure for the dehydropiperidine synthesis

A flame-dried round bottom flask is placed under nitrogen and charged with Rh_2_(OAc)_4_ (0.03 equiv), followed by a solution of the aziridine (0.1 M in dry CH_2_Cl_2_). Upon the addition of the aziridine substrate, a color change of green to purple is observed. To this mixture is added a solution of the diazoester compound (1.2 equiv diluted to 0.1 M in CH_2_Cl_2_) dropwise over 2 h using a syringe pump. The conversion is checked by TLC and ^1^H NMR after the addition of the diazoester is complete; once all the starting material is consumed, the reaction mixture is concentrated and loaded directly onto a silica gel column for purification by chromatography using a gradient of 0–50% EtOAc/hexanes.

## Supplementary information

Supplementary Information

Description of Additional Supplementary Files

Supplementary Data 1

## Data Availability

The authors declare that all data supporting the findings of this study are available within the paper and its Supplementary Information and Supplementary Data 1 files, including experimental procedures, computational details, and characterization data for all new compounds. The crystallographic data for Compound **4cc** are available in the CCDC repository https://www.ccdc.cam.ac.uk/solutions/csd-system/components/csd/ under deposition number 1921208.
