# Supplementary material for: Intermolecular [3+3] ring expansion of aziridines to dehydropiperi-dines through the intermediacy of aziridinium ylides
Source: Nat Commun. 2020 Mar 9;11:1273. doi: 10.1038/s41467-020-15134-x (PMC7062875; doi:10.1038/s41467-020-15134-x)
Supplement: Supplementary file 1 — Supplementary Information [file 41467_2020_15134_MOESM1_ESM.pdf]

Supplementary Information

Intermolecular [3+3] Ring-Expansion of Aziridines to Dehydropiperidines through the Intermediacy of Aziridinium Ylides

Schomaker, J. M. et al.

Supplementary Methods

Unless otherwise specified, all reactions were run under an inert atmosphere of N_2. Glassware was either oven-dried overnight at 130 °C or flame-dried under a stream of dry nitrogen prior to use. Unless otherwise specified, reagents were used as obtained from the vendor without further purification. Procedures for compounds requiring purification were obtained from "Purification of Laboratory Chemicals".[1] Dichloromethane and acetonitrile were dried over CaH_2 and freshly distilled prior to use. All other solvents were also purified in accordance with methods reported in "Purification of Laboratory Chemicals".[1] Analytical thin layer chromatography (TLC) was performed utilizing pre-coated silica gel 60 F_{254} plates containing a fluorescent indicator, while preparative chromatography was performed using SilicaFlash P60 silica gel (230-400 mesh) via Still's method.[2] The mobile phases for column chromatography varied depending on the substrate; however, hexanes/ether, hexanes/ethyl acetate, or toluene/ethyl acetate were commonly employed. Columns were typically run using a gradient method, beginning with 100% of the less polar eluent and gradually increasing the polarity with the other solvent. For reactions producing products without a UV signature, a potassium permanganate stain or CAM stain was employed to visualize the reaction products. ^{1}H NMR and ^{13}C NMR spectra were obtained using Bruker Avance-400, Bruker Avance-500 or Varian Inova-600 NMR spectrometers. For ^{1}H NMR, chemical shifts are reported relative to residual protiated solvent peaks (δ 7.26, 2.49, 7.15 and 4.80 ppm for $CDCl_3$, $(CD_3)_2SO$, C_6D_6 and CD_3OD respectively). ^{13}C NMR spectra were measured at either 125 MHz or 150 MHz on the same instruments noted above for recording ^{1}H NMR spectra. Chemical shifts were again reported in accordance to residual protiated solvent peaks (δ 77.1, 39.5, 128.0 and 49.0 ppm for $CDCl_3$, $(CD_3)_2SO$, C_6D_6, and CD_3OD, respectively). Accurate mass measurements were acquired at the University of Wisconsin, Madison using a Micromass LCT (electrospray ionization, time-of-flight analyzer or electron impact methods). The NMR and Mass Spectrometry facilities are funded by the NSF (CHE-1048642), the NIH (S10 OD012245, 1S10 OD020022-1), the Bender Fund, UW2020, and the University of Wisconsin-Madison.

Preparation of carbamate aziridine precursors. All carbamates, if not explicitly mentioned in the subsequent section, were synthesized according to reported literature procedures.[3]

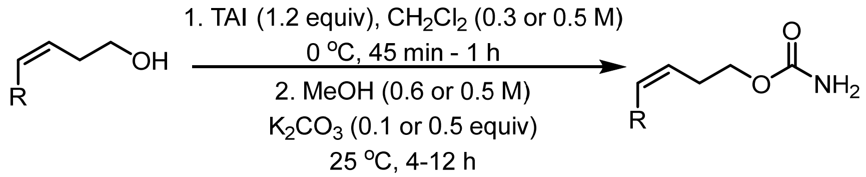

General procedure for carbamate formation. The homoallylic alcohol (1 equiv) was dissolved in CH_2Cl_2 (0.5 or 0.3 M) and cooled to 0 ˚C. Trichloroacetylisocyanate (TAI) (1.2 equiv) was then added dropwise to the cooled solution. The reaction was stirred at 0 ˚C until TLC indicated complete consumption of the starting material (usually between 45 min to 1 h). The solvent was then removed under reduced pressure and the crude reaction mixture was dissolved in MeOH (0.6 or 0.5 M). K_2CO_3 (0.1 or 0.5 equiv) was added in a single portion, and the mixture was stirred at room temperature until TLC indicated complete consumption of the starting material. Saturated aqueous NH_4Cl was added to quench the reaction and the mixture was extracted with three portions of CH_2Cl_2. The combined organic phases were washed with brine. The crude product was then diluted with CH_2Cl_2 (0.05 M) and 1 M aqueous NaOH solution (10 mL/mmol) was added. The biphasic solution was vigorously stirred for 30 min to remove the trichloroacetamide impurity. *Note 1: it is imperative that this step is done carefully. If not, it is likely that the aziridination will not proceed to completion.* The mixture was extracted with three portions of CH_2Cl_2. The combined organic phases were washed with brine, dried over Na_2SO_4 or Mg_2SO_4 and concentrated under reduced pressure. The crude product was purified by silica gel column chromatography (40% EtOAc/ Hexanes).

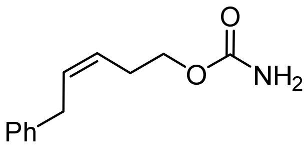

Carbamate precursor to Compound 2a. Following the general procedure, 0.5 M CH_2Cl_2, 0.6 M of MeOH, and 0.1 equiv of K_2CO_3 were used. The reaction to furnish the carbamate precursor to **2a** was conducted on 10 mmol scale. The product was purified by silica gel flash column chromatography (20% to 50% EtOAc/Hex) to yield **2a** as a white solid (1.81g, 88% yield). ^{1}H NMR (500 MHz, $CDCl_3$) δ 7.31 – 7.27 (m, 2H), 7.22 – 7.17 (m, 3H), 5.72 (dtt, *J* = 10.8, 7.5, 1.6 Hz, 1H), 5.52 (dtt, *J* = 10.7, 7.3, 1.7 Hz, 1H), 4.55 (br s, 2H), 4.12 (t, *J* = 6.7 Hz, 2H), 3.43 (dd, *J* = 7.5, 1.5 Hz, 2H), 2.50 (qd, *J* = 6.8, 1.6 Hz, 2H). ^{13}C NMR (126 MHz, $CDCl_3$) δ 156.9, 140.8, 131.1, 128.6, 128.5, 126.1, 125.8, 64.6, 33.6, 27.3. HRMS (ESI) *m/z* calculated for $C_{12}H_{15}NO_2$ $[M+H]^+$ 206.1176; found, 206.1173.

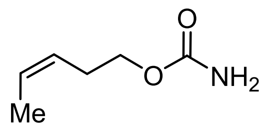

Carbamate precursor to Compound 2b. Following the general procedure, the reaction to furnish carbamate precursor to **2b** was conducted on 1.2 mmol scale using 0.5 M CH_2Cl_2, 0.6 M of MeOH, and 0.1 equiv. of K_2CO_3. The product was purified by silica gel flash column chromatography (10% to 50% Et_2O/Pentane) to yield **2b** as a white solid (154.0 mg, 92% yield). The minor NMR impurity is the isomerized *trans* product, which can be carried on, as it is unreactive in the carbene transfer reaction. ^{1}H NMR (500 MHz, $CDCl_3$) δ 5.58 (dddd, *J* = 13.6, 6.8, 4.9, 3.4 Hz, 1H), 5.38 (dtq, *J* = 10.8, 7.3, 1.9 Hz, 1H), 4.58 (br s, 2H), 4.07 (t, *J* = 6.9 Hz, 2H), 2.39 (q, *J* = 7.0 Hz, 2H), 1.64 (dd, *J* = 6.8, 1.7 Hz, 3H). δ ^{13}C NMR (126 MHz, $CDCl_3$) δ 156.9, 126.8, 125.3, 64.6, 26.8, 12.9. HRMS (ESI) *m/z* calculated for $C_6H_{11}NO_2$ $[M+ Na+]^+$ 152.0862; found, 152.0863.

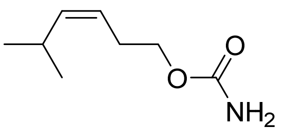

Carbamate precursor to Compound 2e. Following the general procedure, 0.3 M CH_2Cl_2, 0.5 M of MeOH, and 0.5 equiv of K_2CO_3 were used on a 6.02-mmol scale. The product was purified by silica gel flash column chromatography (40% ethyl acetate/ hexanes) to yield **2e** as a white solid (0.69 g, 73% yield). ^{1}H NMR (500 MHz, $CDCl_3$) δ 5.34 (ddt, *J* = 11.0, 9.4, 1.5 Hz, 1H), 5.23 (dt, *J* = 10.7, 7.2 Hz, 1H), 4.53 (br s, 2H), 4.06 (t, *J* = 6.9 Hz, 2H), 2.60 (dp, *J* = 9.4, 6.6 Hz, 1H), 2.39 (qd, *J* = 7.0, 1.5 Hz, 2H), 0.96 (d, *J* = 6.6 Hz, 6H).^{13}C NMR (126 MHz, $CDCl_3$) δ 156.9, 140.4, 121.9, 64.8, 27.2, 26.6, 23.1. HRMS (ESI) *m/z* calculated for $C_8H_{15}NO_2$ $[M+H]^+$ 158.1176; found, 158.1175.

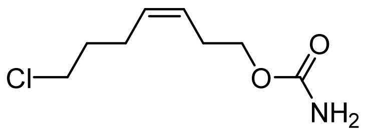

Carbamate precursor to Compound 2f. Following the general procedure, 0.5 M CH_2Cl_2, 0.6 M of MeOH, and 0.1 equiv of K_2CO_3 were used on a 4.6 mmol scale. The product was purified by silica gel flash column chromatography (30% EtOAc/Hex) to yield **2f** as a white solid (830.0 mg, 95% yield) ^{1}H NMR (500 MHz, $CDCl_3$) δ 5.54 – 5.27 (m, 2H), 4.60 (br s, 2H), 4.08 (t, *J* = 6.8 Hz, 2H), 3.54 (t, *J* = 6.5 Hz, 2H), 2.51 – 2.33 (m, 2H), 2.33 – 2.17 (m, 2H), 1.93 – 1.74 (m, 2H). ^{13}C NMR (126 MHz, $CDCl_3$) δ 156.7, 130.5, 126.3, 64.5, 44.4, 32.2, 27.1, 24.4. HRMS (ESI) *m/z* calculated for $C_8H_{14}NO_2$ $[M+H]^+$ 192.0786; found, 192.0784.

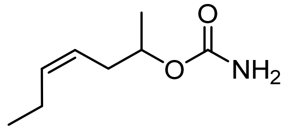

Carbamate precursor to Compound 2h. Following the general procedure, 0.5 M CH_2Cl_2, 0.6 M of MeOH, and 0.1 equiv of K_2CO_3 were used on a 12.2 mmol scale. The product was purified by silica gel flash column chromatography (20% to 50% EtOAc/Hex) to yield **2h** as a yellow low-melting solid (1.17g, 61% yield). ^{1}H NMR (500 MHz, $CDCl_3$) δ 5.63 – 5.40 (m, 1H), 5.40 – 5.11 (m, 1H), 4.81 (h, *J* = 6.3 Hz, 1H), 4.60 (br s, 2H), 2.41 – 2.30 (m, 1H), 2.30 – 2.21 (m, 1H), 2.11 – 2.00 (m, 2H), 1.22 (d, *J* = 6.3

Hz, 3H), 0.96 (t, $J = 7.5$ Hz, 3H). δ ^{13}C NMR (126 MHz, $CDCl_3$) δ 156.7, 134.6, 123.7, 71.6, 33.8, 20.8, 19.8, 14.3. HRMS (ESI) *m/z* calculated for $C_8H_{15}NO_2$ $[M+Na]^+$ 180.0995; found, 180.0994.

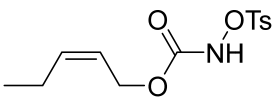

Carbamate precursor to Compound 2i. The *N*-tosyl carbamate was synthesized following Lebel's reported preparation.[4,5] ^{1}H NMR (500 MHz, $CDCl_3$) δ 7.89 – 7.86 (m, 2H), 7.77 (br s, 1H), 7.36 (d, $J = 8.1$ Hz, 2H), 5.62 (dtt, $J = 10.3$, 7.5, 1.4 Hz, 1H), 5.29 (dtt, $J = 10.4$, 7.0, 1.6 Hz, 1H), 4.56 (dd, $J = 7.0$, 1.2 Hz, 2H), 2.46 (s, 3H), 2.04 (pd, $J = 7.6$, 1.6 Hz, 2H), 0.96 (t, $J = 7.5$ Hz, 3H). ^{13}C NMR (126 MHz, $CDCl_3$) δ 155.5, 146.2, 138.2, 130.5, 129.9, 129.7, 121.5, 62.8, 21.9, 21.0, 14.1. δ HRMS (ESI) *m/z* calculated for $C_{13}H_{21}N2O_5S$ $[M+NH_4]^+$ 317.1166; found, 317.1166.

Preparation of aziridine precursors. All aziridines, if not explicitly mentioned in the subsequent section, were synthesized according to reported literature procedures.[3]

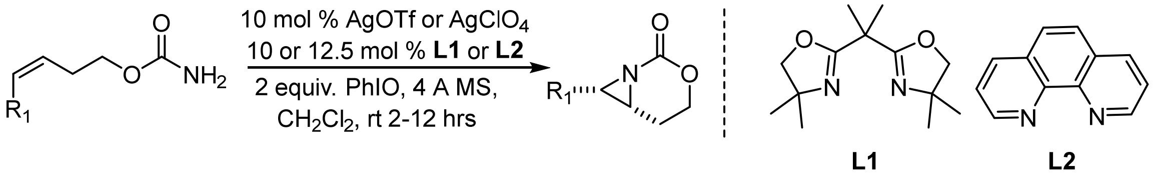

General procedure for silver-catalyzed aziridination of homoallylic carbamates. A cooled, flame-dried roundbottom flask was charged with $AgClO_4$ or AgOTf (0.1 equiv) and dimethyl bisoxazoline **L1** or 1,10-phenanthroline ligand **L2** (0.1 equiv). Dry CH_2Cl_2 (0.05 or 0.1 M) was added to the round-bottom and the mixture was stirred vigorously for 15 minutes. *Note: Both **L1** and **L2** will furnish the aziridine product. **L2** may result in varying ratios of aziridine to C-H insertion product if the metal/ligand ratio is not carefully measured to be 1:1.*[3] ***L1** is the preferable ligand, as the ratio of metal/ligand is not as important to the aziridine/C-H insertion product ratio. However, **L2** is a good*

commercially available alternative if ***L1*** *is unavailable.* After 15 minutes of pre-stirring, powdered 4Å molecular sieves (1g of sieves /mmol of substrate) were added. *Note: It is not necessary to use rigorously dried molecular sieves*. After 5 minutes, the carbamate ester (1 equiv) and iodosobenzene (2 equiv) were added. *Note: it is not essential to wait for 5 minutes, provided the 4Å molecular sieves, carbamate ester, and iodosobenzene are added sequentially in this order.* The reaction was then capped, covered in aluminum foil (if AgOTf is used), and allowed to stir at room temperature for 2-12 h. After TLC indicated complete consumption of the starting material, the reaction mixture was filtered over celite and concentrated. The crude mixture was then purified by column chromatography – once with a gradient elution of 0-2.5% MeOH/CH_2Cl_2, followed by a second column with a gradient elution of 0-50% EtOAc/hexanes or 5% EtOAc/CH_2Cl_2. *Note*: *omitting the first plug with CH_2Cl_2 leaves silver byproducts in the mixture, which, upon concentration, can degrade the aziridine over time or cause decreased reactivity in the subsequent carbene transfer reaction.* The compounds were stored at -20 or -78 ˚C to prevent decomposition. All aziridines not described below were prepared according to published literature procedures.

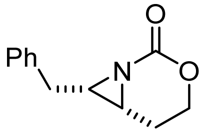

Compound 2a. Following the general procedure, the aziridination reaction to furnish **2a** was conducted on 0.48 mmol scale. The product was purified by silica gel flash column chromatography (0-2.5% MeOH/CH_2Cl_2, then 0 to 5% EtOAc/CH_2Cl_2) to yield **2a** as a white solid (84.9 mg, 86%). ^{1}H NMR (500 MHz, $CDCl_3$ δ 7.34 (tt, *J* = 8.1, 1.5 Hz, 2H), 7.30 – 7.27 (m, 1H), 7.25 – 7.22 (m, 2H), 4.50 – 4.39 (m, 2H), 3.34 (dd, *J* = 15.4, 3.9 Hz, 1H), 2.98 (ddd, *J* = 8.9, 6.8, 4.8 Hz, 1H), 2.90 (ddd, *J* = 10.1, 4.8, 3.9 Hz, 1H), 2.41 (dd, *J* = 15.3, 10.1 Hz, 1H), 2.29 (ddt, *J* = 14.6, 6.8, 1.9 Hz, 1H), 1.69 (dddd, *J* = 14.4, 12.1, 8.9, 4.7 Hz, 1H). ^{13}C NMR (126 MHz, $CDCl_3$) δ 158.6, 136.7, 129.0, 128.5, 127.2, 68.1, 42.8, 37.8, 31.1, 19.7. HRMS (ESI) *m/z* calculated for $C_{12}H_{13}NO_2[M+H]^+$ 204.1019; found, 204.1020.

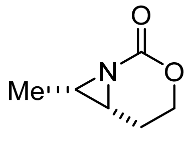

Compound 2b. Following the general procedure, the aziridination reaction to furnish **2b** was conducted on a 0.97 mmol scale. The product was purified by silica gel flash column chromatography (0-2.5% MeOH/CH_2Cl_2, then 0% to 50% EtOAc/Hexanes) to yield **2b** as a clear oil (65.2 mg, 53%). ^{1}H NMR (500 MHz, $CDCl_3$) δ 4.39 (ddd, *J* = 12.4, 10.4, 2.2 Hz, 1H), 4.34 (ddd, *J* = 10.5, 4.6, 1.7 Hz, 1H), 2.89 (ddd, *J* = 8.9, 7.0, 4.9 Hz, 1H), 2.81 – 2.72 (m, 1H), 2.18 (ddt, *J* = 14.7, 6.9, 2.0 Hz, 1H), 1.55 – 1.44 (m, 1H), 1.29 (d, *J* = 6.0 Hz, 3H). ^{13}C NMR (126 MHz, $CDCl_3$) δ 158.5, 67.7, 38.3, 37.6, 18.9, 10.2 HRMS (ESI) *m/z* calculated for $C_6H_9NO_2$[M+H]$^+$ 128.0706; found, 128.0704.

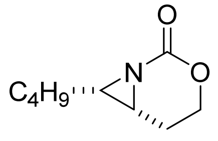

Compound 2d. Following the general procedure, the aziridination reaction to furnish **2d** was conducted on a 1.75 mmol scale. The product was purified by silica gel flash column chromatography (2.5% MeOH/CH_2Cl_2 and 5% EtOAc/CH_2Cl_2). ^{1}H NMR (500 MHz, $CDCl_3$) δ 4.48 – 4.29 (m, 2H), 2.87 (ddd, *J* = 8.9, 6.9, 4.9 Hz, 1H), 2.63 (dt, *J* = 9.4, 4.9 Hz, 1H), 2.18 (ddt, *J* = 14.6, 6.9, 2.0 Hz, 1H), 1.88 (ddt, *J* = 14.2, 9.6, 5.2 Hz, 1H), 1.63 – 1.32 (m, 5H), 1.16 (dtd, *J* = 14.6, 9.2, 5.2 Hz, 1H), 0.92 (t, *J* = 7.0 Hz, 3H). ^{13}C NMR (126 MHz, $CDCl_3$) δ 158.8, 68.0, 42.9, 37.5, 28.8, 25.2, 22.6, 19.3, 14.1. HRMS (ESI) *m/z* calculated for $C_9H_{16}NO_2$ [M+H]$^+$ 170.1176; found, 170.1174.

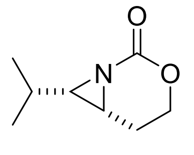

Compound 2e. Following the general procedure, the aziridination reaction to furnish **2e** was conducted on a 3.18 mmol scale. The product was purified by silica gel flash column chromatography (2.5% MeOH/CH_2Cl_2 and 5% EtOAc/CH_2Cl_2). ^{1}H NMR (500 MHz, $CDCl_3$) δ 4.45 – 4.22 (m, 2H), 2.81 (ddd, *J* = 9.1, 6.8, 5.0 Hz, 1H), 2.38 (dd, *J* = 9.9, 5.0 Hz, 1H), 2.19 (ddt, *J* = 14.6, 6.8, 2.0 Hz, 1H), 1.62 – 1.35 (m, 2H), 1.30 (d, *J* = 6.4 Hz, 3H), 1.01 (d, *J* = 6.7 Hz, 3H).^{13}C NMR (126 MHz, $CDCl_3$) δ 158.5, 67.7, 49.4, 36.9, 26.9, 20.1, 20.0, 19.6. HRMS-(ESI) *m/z* calculated for $C_8H_{14}NO_4$ $[M+Na]^+$ 156.1019; found, 156.1019.

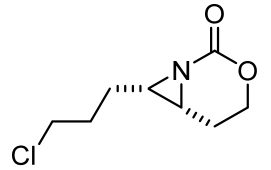

Compound 2f. Following the general procedure, the aziridination reaction to furnish **2f** was conducted on 0.42 mmol scale. The product was purified by silica gel flash column chromatography (2.5% MeOH/CH_2Cl_2 and then 0 to 50% EtOAc/Hexanes) to yield **2f** as a colorless oil (49.7 mg, 62%). ^{1}H NMR (500 MHz, $CDCl_3$) δ 4.46 – 4.33 (m, 2H), 3.63 (ddd, *J* = 11.0, 6.8, 5.2 Hz, 1H), 3.55 (ddd, *J* = 11.0, 7.6, 5.2 Hz, 1H), 2.90 (ddd, *J* = 9.0, 6.9, 4.9 Hz, 1H), 2.65 (ddd, *J* = 7.5, 6.0, 4.9 Hz, 1H), 2.30 – 2.11 (m, 2H), 1.97 – 1.87 (m, 1H), 1.83 (dddd, *J* = 14.5, 9.6, 5.9, 4.8 Hz, 1H), 1.65 – 1.47 (m, 2H). ^{13}C NMR (126 MHz, $CDCl_3$) δ 158.6, 68.0, 44.3, 42.0, 37.4, 29.6, 23.2, 19.3 HRMS (ESI) *m/z* calculated for $C_8H_{12}ClNO_2[M+H]^+$ 190.0629; found, 190.0630.

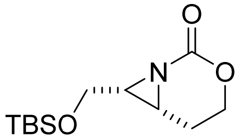

Compound 2g. Following the general procedure, the aziridination reaction to furnish **2g** was conducted on 1.09 mmol scale. The product was purified by silica gel flash column chromatography (2.5% MeOH/CH_2Cl_2 and 5% ethyl acetate/CH_2Cl_2). ^{1}H NMR (500 MHz, $CDCl_3$) δ 4.40 – 4.24 (m, 2H), 4.13 (dd, *J* = 11.8, 3.6 Hz, 1H), 3.39 (dd, *J* = 11.8,

8.4 Hz, 1H), 2.83 (ddd, J = 9.0, 7.0, 4.8 Hz, 1H), 2.70 (ddd, J = 8.4, 4.8, 3.5 Hz, 1H), 2.13 (ddt, J = 14.7, 6.9, 2.0 Hz, 1H), 1.68 (dddd, J = 14.7, 12.2, 8.9, 4.7 Hz, 1H), 0.82 (s, 9H), 0.01 (s, 6H). ^{13}C NMR (126 MHz, $CDCl_3$) δ 158.3, 68.1, 58.5, 41.7, 37.4, 25.8, 19.0, -5.4. HRMS-(ESI) *m/z* calculated for $C_{12}H_{24}NO_3Si$ $[M+H]^+$ 258.1520; found, 258.1518.

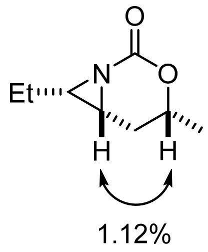

Compound 2h. Following the general procedure, the aziridination reaction to furnish **2h** was conducted on 2.7 mmol scale. The product was purified by silica gel flash column chromatography (2.5% MeOH/CH_2Cl_2 and then 0 to 20% EtOAc/CH_2Cl_2) to yield **2h** as a colorless oil (174.1 mg, 38%). The minor diastereomer was separated *via* automated flash chromatography using the EtOAc/CH_2Cl_2 solvent system. ^{1}H NMR (500 MHz, $CDCl_3$) δ 4.57 (dqd, J = 11.1, 6.3, 2.1 Hz, 1H), 2.82 (ddd, J = 9.0, 6.9, 4.9 Hz, 1H), 2.61 (dt, J = 8.3, 5.1 Hz, 1H), 2.18 (ddd, J = 14.4, 6.9, 2.1 Hz, 1H), 1.86 (ddd, J = 14.4, 7.3, 5.4 Hz, 1H), 1.37 (d, J = 6.2 Hz, 3H), 1.30 – 1.12 (m, 2H),1.10 (t, J = 7.4 Hz, 3H). ^{13}C NMR (126 MHz, $CDCl_3$) δ 159.2, 75.8, 44.8, 37.1, 26.2, 20.7, 19.1, 10.9 HRMS-(ESI) *m/z* calculated for $C_8H_{13}NO_2$$[M+H]^+$ 156.1019; found, 156.1015.

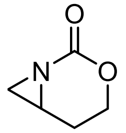

Compound 2i. Following the general procedure, the aziridination reaction to furnish **2i** was conducted on 1.74 mmol scale. The product was purified by silica gel flash column chromatography (0-2.5% MeOH/CH_2Cl_2 and then 0 to 70% EtOAc/Hexanes) to yield **2i** as a colorless oil (53.8 mg, 27% yield). The minor diastereomer was separated *via*

automated flash chromatography using the EtOAc/CH_2Cl_2 solvent system. ^{1}H NMR (500 MHz, $CDCl_3$) δ 4.45 (ddd, J = 12.5, 10.6, 2.0 Hz, 1H), 4.33 (ddd, J = 10.6, 4.1, 1.9 Hz, 1H), 2.81 (ddt, J = 8.4, 6.1, 4.0 Hz, 1H), 2.55 (d, J = 4.4 Hz, 1H), 2.42 (dd, J = 14.7, 6.1 Hz, 1H), 2.07 (d, J = 3.6 Hz, 1H), 1.36 (dddd, J = 14.6, 12.6, 8.7, 4.1 Hz, 1H). ^{13}C NMR (126 MHz, $CDCl_3$) δ 160.91, 68.10, 35.24, 33.91, 25.51. HRMS-(ESI) *m/z* calculated for $C_5H_7NO_2[M+H]^+$ 114.0550; found, 114.0548.

Preparation of diazoesters. All diazoester compounds, if not directly mentioned in the subsequent section, were synthesized according to the reported literature procedures and characterized as described therein.[6,7]

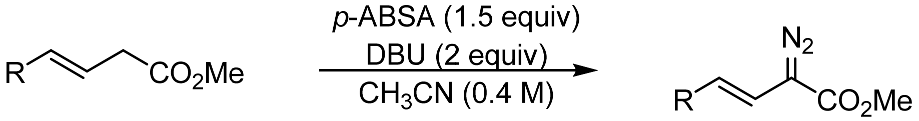

A flame-dried, round bottom flask was placed under N_2 and charged with ester (1 equiv, brought to 0.2 M in CH_3CN). *p*-ABSA (4-acetamidobenzenesulfonyl azide 1.2-10 equiv) was then added in a single portion and the mixture cooled to 0 °C. After 5 minutes of cooling, freshly distilled DBU (2.0-2.2 equiv) was added dropwise to the mixture. This reaction was allowed to warm up to room temperature and stir until the TLC indicated completion. Once complete, the CH_3CN was removed under reduced pressure at room temperature. *Note: diazoesters are heat-sensitive, and can become explosive if they are heated too high.* The concentrated mixture was then taken back up in ether and transferred to a separatory funnel. The organics were then washed twice with saturated aqueous NH_4Cl, dried with $MgSO_4$ and concentrated *in vacuo*. The residue was subjected to silica gel flash column chromatography (generally, 2% Et_2O/pentane to 10% Et_2O/pentane).

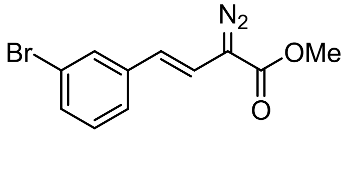

Compound 3c. Following a procedure published by Davies and coworkers[8] from the corresponding aldehyde, the reaction to furnish diazoester **3c** was conducted on 10 mmol scale. The product was purified by silica gel flash column chromatography (2% Et_2O/Pentane to 10% Et_2O/Pentane) to yield **3c** as an orange solid (950.0 mg, 34% yield). ^{1}H NMR (500 MHz, $CDCl_3$) δ 7.50 (t, *J* = 1.9 Hz, 1H), 7.32 (ddd, *J* = 7.8, 1.9, 1.0 Hz, 1H), 7.27 (s, 1H), 7.18 (t, *J* = 7.8 Hz, 1H), 6.50 (d, *J* = 16.3 Hz, 1H), 6.13 (d, *J* = 16.3 Hz, 1H), 3.86 (s, 3H). ^{13}C NMR (126 MHz, $CDCl_3$) δ 165.4, 139.1, 130.3, 130.0, 128.8, 124.6, 123.1, 121.5, 113.4, 52.6, $C_{11}H_9BrN_2O_2$[M+H]$^+$ 280.9920; found, 280.9919.

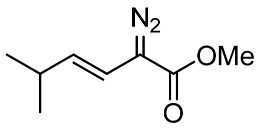

Compound 3g. Following the procedure described above, the reaction to furnish diazoester **3g** was conducted on 8.44 mmol scale. The product was purified by silica gel flash column chromatography (2% Et_2O/Pentane to 10% Et_2O/Pentane) to yield **3g** as a red oil (980.6 mg, 69% yield). ^{1}H NMR (500 MHz, $CDCl_3$) δ 5.69 (dd, *J* = 15.9, 1.4 Hz, 1H), 5.29 (dd, *J* = 15.9, 6.7 Hz, 1H), 3.80 (s, 3H), 2.44 (hd, *J* = 6.7, 1.4 Hz, 1H), 1.03 (d, *J* = 6.8 Hz, 6H). ^{13}C NMR (126 MHz, $CDCl_3$) δ 166.1, 132.6, 109.2, 52.1, 31.40, 22.5. $C_8H_{12}N_2O_2$ [M+H]$^+$ 169.0972; found, 169.0969.

General procedure for piperidine synthesis.

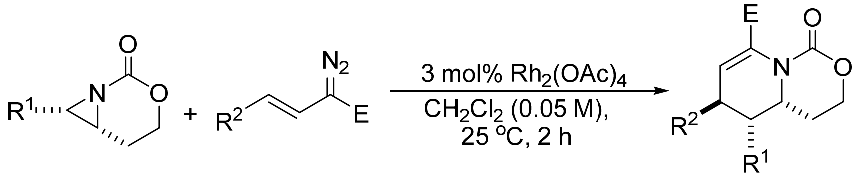

A flame-dried round bottom flask was placed under nitrogen and charged with Rh_2OAc_4 (0.03 equiv), followed by a solution of the aziridine (0.1 mM in dry CH_2Cl_2). *Note: Upon the addition of aziridine, a color change of green to purple will be observed. This does not signify catalyst death; rather, experiments show that if this color change is not observed, the reaction is less likely to proceed.* To this mixture was added a solution

of the diazoester compound (1.2 equiv, brought to 0.1 mM in CH_2Cl_2) dropwise over 2 h using a syringe pump. The conversion was checked by TLC and NMR after the addition was complete; once all the starting material had been consumed, the reaction was concentrated and loaded directly onto silica for column chromatography (0% to 50% EtOAc/Hexanes). *Note: The R_f differences between the aziridine and the piperidine product can be very small.*

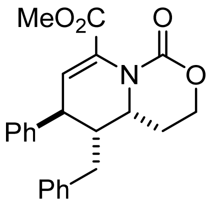

Compound 4aa. Following the general procedure, the carbene transfer to furnish **4aa** was conducted on 0.1 mmol scale. The product was purified by silica gel flash column chromatography (0 to 50% EtOAc/hexanes, 10% increments) to yield **4aa** as a white solid (30.8 mg, 76% yield, >19:1 *dr*). ^{1}H NMR (500 MHz, $CDCl_3$) δ 7.38 (t, *J* = 7.5 Hz, 2H), 7.32 – 7.27 (m, 1H), 7.26 – 7.23 (m, 4H), 7.23 – 7.18 (m, 1H), 6.87 (dd, *J* = 7.0, 1.9 Hz, 2H), 6.12 (dd, *J* = 4.6, 1.4 Hz, 1H), 4.45 – 4.25 (m, 2H), 3.87 (s, 3H), 3.87 – 3.81 (m, 1H), 3.50 (d, *J* = 4.6 Hz, 1H), 2.98 (dd, *J* = 13.7, 3.0 Hz, 1H), 2.34 – 2.20 (m, 2H), 2.13 (ddq, *J* = 11.4, 2.8, 1.4 Hz, 1H), 2.02 (ddt, *J* = 14.2, 8.0, 2.0 Hz, 1H).^{13}C NMR (126 MHz, $CDCl_3$) δ 164.1, 152.6, 143.1, 139.0, 133.3, 129.3, 128.8, 128.6, 128.1, 126.9, 126.6, 120.0, 64.6, 52.6, 51.6, 46.7, 41.0, 32.6, 25.5. HRMS (ESI) *m/z* calculated for $C_{23}H_{23}NO_4$ [M+H]+ 378.1700; found, 378.1696.

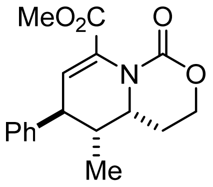

Compound 4ba. Following the general procedure, the carbene transfer to furnish **4ba** was conducted on 0.196 mmol scale. The product was purified by silica gel flash column

chromatography (0 to 50% EtOAc/Hexanes, 10% increments) to yield **4ba** as a white solid (38.4 mg, 65% yield, >19:1 *dr*). ^{1}H NMR (500 MHz, $CDCl_3$) δ 7.35 (dd, J = 8.3, 6.9 Hz, 2H), 7.29 – 7.27 (m, 1H), 7.24 – 7.13 (m, 2H), 6.11 (dd, J = 4.6, 1.5 Hz, 1H), 4.35 – 4.23 (m, 2H), 3.85 (s, 3H), 3.70 (ddd, J = 10.4, 7.9, 1.8 Hz, 1H), 3.44 (d, J = 4.6 Hz, 1H), 2.08 – 1.93 (m, 2H), 1.88 (ddt, J = 14.3, 7.8, 2.1 Hz, 1H), 1.07 (d, J = 6.9 Hz, 3H). ^{13}C NMR (126 MHz, $CDCl_3$) δ 164.5, 152.9, 143.5, 133.0, 128.9, 128.4, 127.2, 120.1, 64.7, 52.7, 51.9, 46.1, 39.1, 25.9, 13.9. HRMS (ESI) *m/z* calculated for $C_{17}H_{19}NO_4$ $[2M+NH_4]^+$; 620.2966 found, 620.2973.

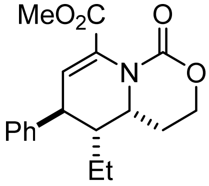

Compound 4ca. Following the general procedure, the carbene transfer to furnish **4ca** was conducted on 1.00 mmol scale. The product was purified by silica gel flash column chromatography (0 to 50% EtOAc/hexanes, 10% increments) to yield **4ca** as a white solid (237.9 mg, 75% yield, >19:1 *dr*). ^{1}H NMR (500 MHz, $CDCl_3$) δ 7.35 (t, J = 7.6 Hz, 2H), 7.27 (d, J = 8.3 Hz, 1H), 7.20 – 7.14 (m, 2H), 6.12 (dd, J = 4.6, 1.4 Hz, 1H), 4.33 – 4.24 (m, 2H), 3.84 (s, 3H), 3.72 (ddd, J = 10.1, 8.1, 1.7 Hz, 1H), 3.66 (d, J = 4.6 Hz, 1H), 2.12 – 2.01 (m, 1H), 1.90 (ddt, J = 14.3, 8.1, 2.0 Hz, 1H), 1.65 – 1.54 (m, 3H), 1.16 – 1.13 (m, 3H). ^{13}C NMR (126 MHz, $CDCl_3$) δ 164.2, 152.7, 143.3, 133.3, 128.7, 128.3, 126.9, 120.3, 64.6, 52.5, 51.7, 47.0, 41.8, 25.5, 19.0, 12.2 HRMS (ESI) *m/z* calculated for $C_{18}H_{21}NO_4[M+H]^+$ 316.1543; found, 316.1541.

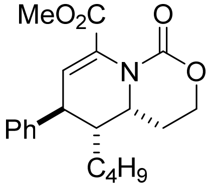

Compound 4da. Following the general procedure, the carbene transfer to furnish **4da** was conducted on 0.29 mmol scale. The product was purified by silica gel flash column

chromatography (0 to 40% EtOAc/Hexanes, 10% increments) to yield **4da** as a white solid (275.2 mg, 92% yield, >19:1 *dr*). ^{1}H NMR (500 MHz, $CDCl_3$) δ 7.35 (t, *J* = 7.5 Hz, 3H), 7.20 – 7.13 (m, 2H), 6.11 (dd, *J* = 4.6, 1.5 Hz, 1H), 4.33 – 4.25 (m, 2H), 3.84 (s, 3H), 3.76 – 3.67 (m, 1H), 3.63 (d, *J* = 4.6 Hz, 1H), 2.05 (dt, *J* = 14.2, 5.0 Hz, 1H), 1.89 (ddt, *J* = 14.3, 8.0, 2.0 Hz, 1H), 1.75 – 1.60 (m, 2H), 1.53 – 1.33 (m, 3H), 1.15 (dddd, *J* = 13.7, 10.4, 8.9, 4.6 Hz, 1H), 0.95 (t, *J* = 6.9 Hz, 3H). ^{13}C NMR (126 MHz, $CDCl_3$) δ 164.3, 152.7, 143.4, 133.3, 128.7, 128.3, 127.0, 120.4, 64.6, 52.5, 51.7, 45.1, 42.6, 30.1, 25.9, 25.5, 22.8, 14.1. HRMS (ESI) *m/z* calculated for $C_{20}H_{26}NO_4$ $[M+H]^+$ 344.1856; found, 344.1853.

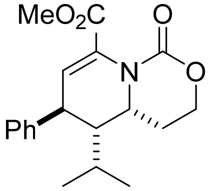

Compound 4ea. Following the general procedure, the carbene transfer to furnish **4ea** was conducted on 0.32 mmol scale. The product was purified by silica gel flash column chromatography (0 to 40% EtOAc/Hexanes, 10% increments) to yield **4ea** as a white solid (74.2 mg, 70% yield, >19:1 *dr*). ^{1}H NMR (500 MHz, $CDCl_3$) δ 7.34 (dd, *J* = 8.2, 7.0 Hz, 2H), 7.25 (d, *J* = 7.4 Hz, 1H), 7.21 – 7.16 (m, 2H), 6.13 (dd, *J* = 4.3, 1.2 Hz, 1H), 4.37 – 4.29 (m, 2H), 3.83 (m, 4H), 3.63 (d, *J* = 4.4 Hz, 1H), 2.27 (ddt, *J* = 14.2, 11.3, 8.4 Hz, 1H), 2.15 (m, 1H), 1.86 (ddt, *J* = 14.3, 7.4, 2.0 Hz, 1H), 1.72 (hept, *J* = 2.5, 1.1 Hz, 1H), 1.18 (d, *J* = 6.8 Hz, 3H), 0.91 (d, *J* = 6.9 Hz, 3H). ^{13}C NMR (126 MHz, $CDCl_3$) δ 164.3, 151.9, 144.6, 133.4, 128.8, 128.0, 126.8, 121.8, 64.8, 52.5, 52.2, 49.5, 37.9, 25.7, 25.1, 24.1, 17.4.

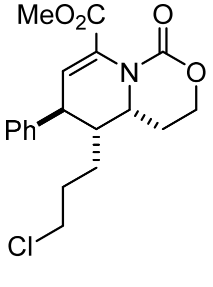

Compound 4fa. Following the general procedure, the carbene transfer to furnish **4fa** was conducted on 0.1 mmol scale. The product was purified by silica gel flash chromatography (0 to 50% EtOAc/Hexanes, 10% increments) to yield compound **4fa** as a white solid (25.1 mg, 69% yield, >19:1 *dr*). ^{1}H NMR (500 MHz, $CDCl_3$) δ 7.36 (t, *J* = 7.6 Hz, 2H), 7.30 – 7.27 (m, 1H), 7.17 – 7.14 (m, 2H), 6.11 (dd, *J* = 4.7, 1.5 Hz, 1H), 4.35 – 4.26 (m, 2H), 3.85 (s, 3H), 3.75 (ddd, *J* = 10.3, 8.2, 1.7 Hz, 1H), 3.64 – 3.50 (m, 3H), 2.25 – 2.12 (m, 1H), 2.12 – 2.03 (m, 1H), 1.97 – 1.84 (m, 2H), 1.78 – 1.65 (m, 1H), 1.26 (tt, *J* = 10.1, 4.7 Hz, 2H). ^{13}C NMR (126 MHz, $CDCl_3$) δ 164.1, 152.5, 142.9, 133.4, 128.8, 128.2, 127.1, 119.8, 64.5, 52.6, 51.5, 44.3, 44.2, 42.6, 30.7, 25.5, 23.6. HRMS (ESI) *m/z* calculated for $C_{19}H_{22}ClNO_4$ [M+H]+ 364.1310; found, 364.1306.

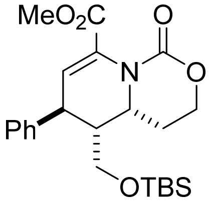

Compound 4ga. Following the general procedure, the carbene transfer to furnish **4ga** was conducted on 0.19 mmol scale. The product was purified by silica gel flash column chromatography (0 to 40% EtOAc/Hexanes, 10% increments) to yield **4ga** as a white solid (70.6 mg, 74% yield, >19:1 *dr*). ^{1}H NMR (500 MHz, $CDCl_3$) δ 7.39 – 7.30 (m, 2H), 7.28 (t, *J* = 1.3 Hz, 1H), 7.21 – 7.11 (m, 2H), 6.11 (dd, *J* = 4.6, 1.5 Hz, 1H), 4.37 – 4.21 (m, 2H), 3.87 – 3.71 (m, 6H), 3.46 (dd, *J* = 10.3, 8.4 Hz, 1H), 2.31 (ddt, *J* = 14.4, 11.1, 8.6 Hz, 1H), 2.06 (ddd, *J* = 9.1, 5.4, 1.8 Hz, 1H), 2.03 – 1.93 (m, 1H), 0.93 (s, 9H), 0.08 (s, 6H). ^{13}C NMR (126 MHz, $CDCl_3$) δ 164.4, 152.3, 143.5, 133.2, 128.9, 128.4, 127.1, 120.4, 64.9, 60.8, 52.7, 50.9, 47.7, 40.6, 26.0, 0.2, -5.3. HRMS (ESI) *m/z* calculated for $C_{23}H_{34}NO_5Si$ $[M+H]^+$ 432.2201; found, 432.2201.

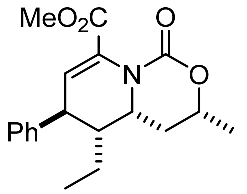

Compound 4ha. Following the general procedure, the carbene transfer to furnish **4ha** was conducted on 0.1 mmol scale. The product was purified by silica gel flash column chromatography (0 to 35% EtOAc/Hexanes, 10% increments) to yield compound **4ha** as a colorless waxy solid (24.0 mg, 72% yield, >19:1 *dr*). A residual minor NMR impurity results from the difficulty of separating the C-H insertion product from the aziridine product in the preceding step. ^{1}H NMR (500 MHz, $CDCl_3$) δ 7.34 (dd, *J* = 8.2, 7.0 Hz, 2H), 7.27 (d, *J* = 1.4 Hz, 1H), 7.22 – 7.03 (m, 2H), 6.10 (dd, *J* = 4.6, 1.4 Hz, 1H), 4.49 (dqd, *J* = 12.5, 6.3, 1.9 Hz, 1H), 3.84 (s, 3H), 3.71 – 3.66 (m, 1H), 3.65 (d, *J* = 4.6 Hz, 1H), 1.90 (ddd, *J* = 14.0, 8.0, 2.0 Hz, 1H), 1.77 (dt, *J* = 14.0, 11.1 Hz, 1H),1.62 – 1.53 (m, 2H)1.37 (d, *J* = 6.2 Hz, 3H), 1.19 – 1.11 (m, 4H). ^{13}C NMR (126 MHz, $CDCl_3$) δ 164.5, 152.8, 143.5, 133.4, 128.8, 128.4, 127.0, 120.3, 72.1, 52.6, 51.4, 47.1, 42.0, 32.7, 20.3, 19.1, 12.3 HRMS (ESI) *m/z* calculated $C_{19}H_{23}NO_4$ [M+H]+ 330.1700; found, 330.1698.

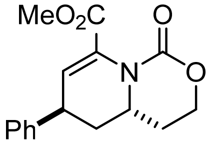

Compound 4ia. Following the general procedure, the carbene transfer to furnish **4ia** was conducted on 0.17 mmol scale. The product was purified by silica gel flash column chromatography (0 to 50% EtOAc/Hexanes, 10% increments) to yield **4ia** as a white solid (33.8 mg, 67% yield, >19:1 *dr*). ^{1}H NMR (500 MHz, $CDCl_3$) δ 7.43 – 7.32 (m, 2H), 7.28 (d, *J* = 7.4 Hz, 1H), 7.21 – 7.16 (m, 2H), 6.17 (dd, *J* = 4.3, 1.3 Hz, 1H), 4.38 – 4.23 (m, 2H), 3.84 (s, 3H), 3.72 (ddd, *J* = 6.1, 4.4, 1.6 Hz, 1H), 3.63 (tdd, *J* = 10.5, 6.7, 2.9 Hz, 1H), 2.12 (ddt, *J* = 14.3, 6.7, 2.1 Hz, 1H), 2.09 – 1.95 (m, 2H), 1.90 – 1.73 (m, 1H). ^{13}C NMR (126 MHz, $CDCl_3$) δ 164.7, 151.5, 143.7, 133.1, 128.8, 127.9, 127.1, 121.9, 64.7, 52.5, 49.1, 38.5, 37.2, 29.3. HRMS (ESI) *m/z* calculated for $C_{16}H_{17}NO_4$ $[M+H]^+$ 288.1230; found, 299.1225.

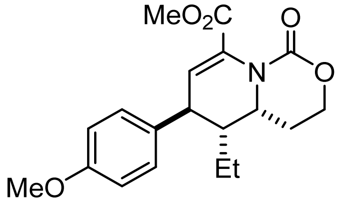

Compound 4cb. Following the general procedure, the carbene transfer to furnish **4cb** was conducted on 0.16 mmol scale. The product was purified by silica gel flash column chromatography (0 to 50% EtOAc/hexanes, 10% increments) to yield compound **4cb** as a white solid (42.9 mg, 78% yield, >19:1 *dr*). ^{1}H NMR (500 MHz, $CDCl_3$) δ 7.08 (d, *J* = 8.6 Hz, 2H), 6.87 (d, *J* = 8.7 Hz, 2H), 6.10 (dd, *J* = 4.6, 1.4 Hz, 1H), 4.39 – 4.21 (m, 2H), 3.84 (s, 3H), 3.80 (s, 3H), 3.70 (ddd, *J* = 10.1, 8.1, 1.6 Hz, 1H), 3.60 (d, *J* = 4.6 Hz, 1H), 2.06 (dtd, *J* = 14.2, 10.7, 6.6 Hz, 1H), 1.89 (ddt, *J* = 14.3, 8.1, 2.0 Hz, 1H), 1.66 – 1.49 (m, 2H), 1.15 – 1.10 (m, 4H). ^{13}C NMR (126 MHz, $CDCl_3$) δ 164.3, 158.5, 152.7, 135.4, 133.0, 129.3, 120.7, 114.0, 64.6, 55.3, 52.5, 51.6, 47.2, 41.0, 25.5, 18.9, 12.2 HRMS (ESI) *m/z* calculated for $C_{19}H_{23}NO_5$ [M+H+] 346.164; found, 346.1646.

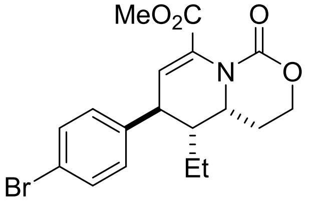

Compound 4cc. Following the general procedure, the carbene transfer to furnish **4cc** was conducted on 0.35 mmol scale. The product was purified by silica gel flash column chromatography (0 to 40% EtOAc/Hexanes, 10% increments) to yield **4cc** as a white solid (90.6 mg, 72% yield, >19:1 *dr*).^{1}H NMR (500 MHz, $CDCl_3$) δ 7.51 – 7.39 (m, 2H), 7.09 – 7.02 (m, 2H), 6.06 (dd, *J* = 4.6, 1.5 Hz, 1H), 4.34 – 4.24 (m, 2H), 3.84 (s, 3H), 3.65 (ddd, *J* = 10.3, 8.2, 1.6 Hz, 1H), 3.61 (d, *J* = 4.6 Hz, 1H), 2.13 – 2.01 (m, 1H), 1.90 (m, , 1H), 1.65 – 1.52 (m, 3H), 1.16 – 1.11 (m, 3H). ^{13}C NMR (126 MHz, $CDCl_3$) δ 164.3, 152.7, 142.5, 133.8, 132.0, 130.2, 121.1, 119.6, 64.7, 52.8, 51.8, 47.1, 41.4, 25.7, 19.1, 12.3. HRMS (ESI) *m/z* calculated for $C_{18}H_{20}BrNO_4Na$ $[M+Na]^+$ 416.0468; found, 416.0461.

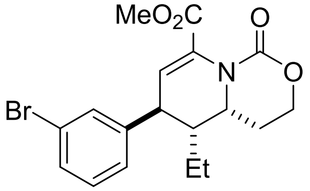

Compound 4cd. Following the general procedure, the carbene transfer to furnish **4cd** was conducted on 0.12 mmol scale. The product was purified by silica gel flash chromatography (0 to 50% EtOAc/hexanes, 10% increments) to yield **4cd** as a white solid (34.6 mg, 73% yield, >19:1 *dr*). ^{1}H NMR (500 MHz, $CDCl_3$) δ 7.40 (dt, *J* = 8.4, 1.2 Hz, 1H), 7.29 (d, *J* = 1.9 Hz, 1H), 7.22 (t, *J* = 7.8 Hz, 1H), 7.10 (dt, *J* = 7.7, 1.4 Hz, 1H), 6.05 (dd, *J* = 4.6, 1.5 Hz, 1H), 4.42 – 4.24 (m, 2H), 3.84 (s, 3H), 3.67 (ddd, *J* = 10.2, 8.1, 1.6 Hz, 1H), 3.62 (d, *J* = 4.6 Hz, 1H), 2.07 (dtd, *J* = 14.3, 11.0, 5.9 Hz, 1H), 1.92 (ddt, *J* = 14.3, 8.1, 2.0 Hz, 1H), 1.66 – 1.53 (m, 2H), 1.13 (dd, *J* = 6.0, 3.5 Hz, 4H). ^{13}C NMR (126 MHz, $CDCl_3$) δ 164.2, 152.8, 145.9, 134.0, 131.4, 130.4, 130.3, 127.2, 123.1, 119.3, 64.7, 52.8, 51.9, 47.1, 41.6, 25.6, 19.1, 12.3. HRMS (ESI) *m/z* calculated for $C_{18}H_{20}BrNO_4$ [2M+NH4]+ 804.1490; found, 804.1501.

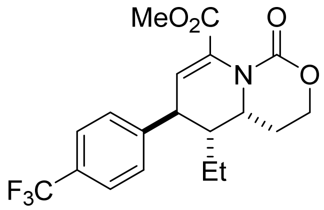

Compound 4ce. Following the general procedure, the carbene transfer to furnish **4ce** was conducted on 0.25 mmol scale using 4 mol% of Rh_2OAc_4. The product was purified by silica gel flash column chromatography (0 to 40% EtOAc/Hexanes, 10% increments) to yield **4ce** as a white solid (67.8 mg, 69% yield, >19:1 *dr*). ^{1}H NMR (500 MHz, $CDCl_3$) δ 7.61 (d, *J* = 8.1 Hz, 2H), 7.30 (d, *J* = 8.0 Hz, 2H), 6.08 (dd, *J* = 4.6, 1.4 Hz, 1H), 4.31 (t, *J* = 1.6 Hz, 1H), 3.84 (s, 3H), 3.72 (d, *J* = 4.6 Hz, 1H), 3.67 (ddd, *J* = 10.1, 8.1, 1.6 Hz, 1H), 2.12 – 2.05 (m, 1H), 1.92 (ddt, *J* = 14.3, 8.1, 2.0 Hz, 1H), 1.67 – 1.56 (m, 2H), 1.21 – 1.12 (m, 3H). ^{13}C NMR (126 MHz, $CDCl_3$) δ 164.2, 152.7, 147.5, 134.0, 129.5, 128.8,

125.8, 119.1, 64.7, 52.7, 51.8, 46.9, 41.7, 25.5, 19.1, 12.2. HRMS (ESI) *m/z* calculated for $C_{19}H_{21}F_3NO_4$ [M+H]+ 384.1417; found, 384.1407.

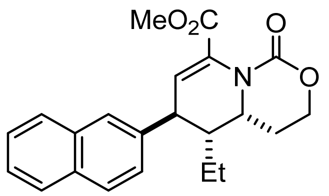

Compound 4cf. Following the general procedure, the carbene transfer to furnish **4cf** was conducted on 0.1 mmol scale. The product was purified by silica gel flash chromatography (0 to 50% EtOAc/hexanes, 10% increments) to yield **4cf** as a white solid (28.4 mg, 78% yield, >19:1 *dr*). ^{1}H NMR (500 MHz, $CDCl_3$) δ 7.98 – 7.61 (m, 3H), 7.62 – 7.41 (m, 3H), 7.31 (dd, *J* = 8.5, 1.9 Hz, 1H), 6.23 (dd, *J* = 4.6, 1.5 Hz, 1H), 4.37 – 4.22 (m, 2H), 3.89 (s, 3H), 3.81 (d, *J* = 4.6 Hz, 1H), 3.76 (ddd, *J* = 10.2, 8.2, 1.7 Hz, 1H), 2.17 – 1.98 (m, 1H), 1.84 (ddt, *J* = 14.2, 8.1, 2.0 Hz, 1H), 1.73 – 1.67 (m, 1H), 1.68 – 1.58 (m, 1H), 1.24 – 1.16 (m, 4H). ^{13}C NMR (126 MHz, $CDCl_3$) δ 164.4, 152.9, 140.8, 133.7, 133.3, 132.4, 128.8, 127.9, 127.7, 127.1, 126.7, 126.6, 126.2, 120.4, 64.7, 52.7, 51.8, 46.8, 42.0, 25.6, 19.1, 12.3. HRMS (ESI) *m/z* calculated for $C_{22}H_{23}NO_4$ [M+H]+ 366.1700; found, 366.1697.

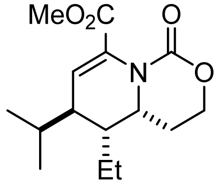

Compound 4cg. Following the general procedure, the carbene transfer to furnish **4cg** was conducted on 0.18 mmol scale. The product was purified by silica gel flash chromatography (0 to 50% EtOAc/hexanes, 10% increments) to yield (0 to 50% EtOAc/hexanes, 10% increments) **4cg** as a white solid (38.0 mg, 75% yield, >19:1 *dr*). ^{1}H NMR (500 MHz, $CDCl_3$) δ 6.11 (dd, *J* = 4.5, 1.5 Hz, 1H), 4.31 (dd, *J* = 9.0, 2.2 Hz, 2H), 3.79 (s, 3H), 3.67 (ddd, *J* = 10.3, 8.2, 1.8 Hz, 1H), 2.20 – 2.03 (m, 2H), 1.89 (dd, *J* =

8.3, 4.5 Hz, 1H), 1.62 – 1.56 (m, 1H), 1.49 (ttd, *J* = 10.7, 7.5, 6.6, 4.2 Hz, 2H), 1.03 (d, *J* = 6.7 Hz, 3H), 1.00 – 0.89 (m, 7H). ^{13}C NMR (126 MHz, $CDCl_3$) δ 164.3, 152.7, 131.2, 123.6, 64.6, 53.7, 52.3, 43.7, 41.0, 33.0, 25.8, 21.1, 20.7, 19.0, 11.7 HRMS (ESI) *m/z* calculated for $C_{15}H_{23}NO_4$ $[M+H]^+$ 282.1700; found, 282.1698.

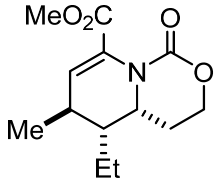

Compound 4ch. Following the general procedure, the carbene transfer to furnish **4ch** was conducted on 0.18 mmol scale. The product was purified by silica gel flash column chromatography (0 to 50% EtOAc/hexanes, 10% increments) to yield **4ch** as a white solid (30.5 mg, 69% yield, >19:1 *dr*). ^{1}H NMR (500 MHz, $CDCl_3$) δ 6.02 (dd, *J* = 4.5, 1.4 Hz, 1H), 4.41 – 4.26 (m, 2H), 3.77 (s, 3H), 3.73 (ddd, *J* = 10.2, 8.2, 1.7 Hz, 1H), 2.41 (qd, *J* = 7.2, 4.4 Hz, 1H), 2.23 – 2.02 (m, 2H), 1.49 (ddt, *J* = 13.4, 7.1, 3.9 Hz, 1H), 1.35 (d, *J* = 9.4 Hz, 1H), 1.09 (d, *J* = 7.2 Hz, 3H), 0.94 (m, 4H).^{13}C NMR (126 MHz, $CDCl_3$) δ 164.4, 152.9, 130.9, 126.4, 64.7, 52.6, 52.5, 45.0, 31.3, 25.9, 21.8, 19.1, 12.1 HRMS (ESI) *m/z* calculated for $C_{13}H_{19}NO_4$ [M+H]+ 254.1387; found, 254.1385.

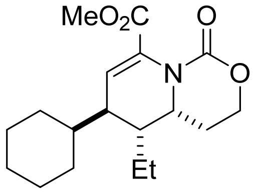

Compound 4ci. Following the general procedure, the carbene transfer to furnish **4ci** was conducted on 0.35 mmol scale. The product was purified by silica gel flash column chromatography (0 to 50% EtOAc/Hexanes, 10% increments) to yield **4ci** as a white solid (77.3 mg, 70% yield, >19:1 *dr*). ^{1}H NMR (500 MHz, $CDCl_3$) δ 6.10 (dd, *J* = 4.6, 1.4 Hz, 1H), 4.31 (dd, *J* = 7.7, 1.9 Hz, 2H), 3.78 (s, 3H), 3.68 (ddd, *J* = 10.2, 8.2, 1.7 Hz, 1H), 2.17 – 2.06 (m, 1H), 1.97 (dd, *J* = 7.7, 4.5 Hz, 1H), 1.88 – 1.44 (m, 9H), 1.33 – 1.07 (m, 5H), 1.00 – 0.86 (m, 4H). ^{13}C NMR (126 MHz, $CDCl_3$) δ 164.5, 152.9, 131.3, 123.8,

64.8, 54.1, 52.5, 43.0, 42.7, 41.0, 31.5, 31.2, 26.5, 26.0, 19.2, 12.0. HRMS (ESI) *m/z* calculated for $C_{18}H_{28}NO_4$ $[M+H]^+$ 322.2013; found, 322.2010.

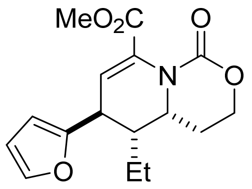

Compound 4cj. Following the general procedure, the carbene transfer to furnish **4cj** was conducted on 0.3542 mmol scale. The product was purified by silica gel flash column chromatography (0 to 50% EtOAc/Hexanes, 10% increments) to yield **4cj** as a yellow solid (15.7 mg, 14% yield, >19:1 *dr*). ^1H NMR (500 MHz, $CDCl_3$) δ 7.36 (dd, *J* = 1.9, 0.8 Hz, 1H), 6.31 (dd, *J* = 3.2, 1.9 Hz, 1H), 6.08 (dd, *J* = 4.6, 1.4 Hz, 1H), 6.03 (dd, *J* = 3.2, 0.9 Hz, 1H), 4.43 – 4.23 (m, 2H), 3.84 – 3.74 (m, 5H), 3.65 (d, *J* = 4.7 Hz, 1H), 2.15 – 2.05 (m, 1H), 2.01 (ddt, *J* = 14.3, 8.2, 2.1 Hz, 1H), 1.90 – 1.80 (m, 1H), 1.62 – 1.49 (m, 1H), 1.12 – 1.02 (m, 3H). ^{13}C NMR (126 MHz, $CDCl_3$) δ 164.3, 155.4, 142.2, 133.3, 118.2, 110.4, 107.4, 64.8, 53.2, 52.7, 43.0, 36.4, 25.7, 18.6, 12.1. HRMS (ESI) *m/z* calculated for $C_{16}H_{20}NO_5$ $[M+H]^+$ 306.1336; found, 306.1331.

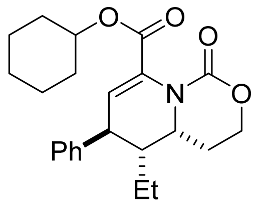

Compound 4ck. Following the general procedure, the carbene transfer to furnish **4ck** was conducted on 0.25 mmol scale. The product was purified by silica gel flash column chromatography (0 to 50% EtOAc/Hexanes, 10% increments) to yield **4ck** as a white solid (52.9 mg, 56% yield, >19:1 *dr*). ^1H NMR (500 MHz, $CDCl_3$) δ 7.35 (dd, *J* = 8.2, 6.9 Hz, 2H), 7.30 – 7.26 (m, 1H), 7.19 – 7.13 (m, 2H), 6.10 (dd, *J* = 4.6, 1.4 Hz, 1H), 4.91 (tt, *J* = 8.9, 3.9 Hz, 1H), 4.31 – 4.25 (m, 2H), 3.72 (ddd, *J* = 10.4, 8.2, 1.5 Hz, 1H), 3.65 (d, *J* = 4.5 Hz, 1H), 2.13 – 1.85 (m, 2H), 1.75 (td, *J* = 8.6, 8.1, 3.9 Hz, 2H), 1.66 – 1.46 (m, 6H), 1.45 – 1.34 (m, 1H), 1.33 – 1.16 (m, 2H), 1.15 (t, *J* = 4.8 Hz, 3H). ^{13}C NMR

(126 MHz, $CDCl_3$) δ 163.3, 152.8, 143.8, 134.2, 128.9, 128.5, 127.0, 120.1, 64.7, 51.9, 47.3, 42.0, 31.7, 31.5, 25.8, 25.6, 24.0, 19.2, 12.4, 0.2. HRMS (ESI) *m/z* calculated for $C_{23}H_{30}NO_4$ $[M+H]^+$ 384.2169; found, 384.2165.

Piperidine derivatizations

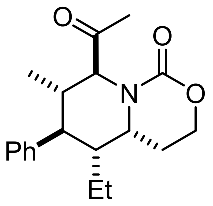

Compound 6. In accordance with literature conditions,[9] a flame-dried round bottom flask was charged with piperidine **4ca (**0.23 mmol, 1 equiv) and diluted to 0.01 M with freshly distilled ether. This solution was cooled to -78 ˚C and allowed to stir while a second flame-dried round bottom flask was charged with CuCN (1.2 mmol, 5 equiv) and diluted with 12 mL of freshly distilled ether (0.1 M) and cooled to -35 ˚C. To the flask containing CuCN was added a solution of MeLi dropwise (1.5 mL, 1.6 M solution in Et_2O, 10 equiv). After five minutes, the mixture was warmed up to 0 ˚C and allowed to stir for 30 additional minutes. After this period, the solution was cannula transferred to the cooled mixture of **4ca** in Et_2O. After five minutes, the reaction mixture was allowed to warm to room temperature and stirred until TLC indicated complete consumption of the starting material. The reaction was then quenched with saturated NH_4Cl and diluted with CH_2Cl_2. This biphasic mixture was filtered to remove solids and the resulting biphasic mixture transferred to a separatory funnel. The layers were separated, and the aqueous layer extracted with three portions of CH_2Cl_2. The organics were combined, dried with Na_2SO_4, and the volatiles concentrated under reduced pressure and purified by silica gel flash chromatography. (0 to 20% EtOAc/CH_2Cl_2, 10% increments) to yield **6** as a light-yellow oil (42.0 mg 57% yield). The minor NMR impurity is attributed to the conjugate addition product containing the ester. ^1H NMR (500 MHz, $CDCl_3$) δ 7.38 – 7.29 (m, 3H), 7.26 – 7.20 (m, 1H), 7.19 – 7.14 (m, 3H), 4.41 (d, *J* = 7.6 Hz, 1H), 4.39 – 4.35 (m, 1H), 4.35 – 4.26 (m, 2H), 2.48 (ddd, *J* = 14.0, 6.8, 3.4 Hz, 1H), 2.34 (s, 3H), 2.17 (dd, *J* = 10.6, 6.8 Hz, 1H), 2.13 – 2.02 (m, 2H), 1.96 (ddt, *J* = 13.8, 5.4, 2.8 Hz, 1H), 1.77 (qd, *J* = 6.9, 4.6 Hz, 1H), 1.40 – 1.30 (m, 1H), 1.12 (dt, *J* = 14.3, 7.2 Hz, 1H), 0.93 –

0.81 (m, 8H). ^{13}C NMR (126 MHz, $CDCl_3$) δ 208.5, 154.8, 144.4, 128.9, 127.9, 126.9, 66.7, 65.4, 52.9, 50.2, 45.0, 36.8, 29.4, 24.1, 23.1, 18.3, 11.6. HRMS (ESI) *m/z* calculated for $C_{19}H_{25}NO_3$ $[M+H]^+$ 316.1907; found, 316.108.

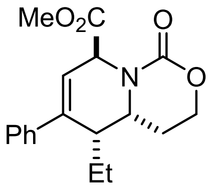

Compound 7. A flame-dried round bottom flask was charged with piperidine **4ca** and diluted with MeCN (0.1 mmol). To this solution was added DBU, dropwise (0.951 mmol, 3 equiv). This mixture was heated to 50 °C and was allowed to stir until TLC indicated completion (approximately 18 h). After TLC indicated complete consumption of the starting material the solution was cooled to room temperature and quenched with saturated NH_4Cl^+ and extracted with three portions of EtOAc. The combined organic layers were dried with Na_2SO_4, concentrated under reduced pressure and purified by silica gel flash column chromatography (0 to 60% EtOAc/Hexanes) to yield Compound **7** as a viscous light-yellow oil (75.3 mg, 74% yield). ^1H NMR (500 MHz, $CDCl_3$) δ 7.36 (d, J = 4.3 Hz, 4H), 7.32 (dd, J = 4.9, 3.7 Hz, 1H), 6.08 (d, J = 3.8 Hz, 1H), 5.28 (d, J = 3.8 Hz, 1H), 4.44 – 4.29 (m, 2H), 4.01 (ddd, J = 9.6, 6.1, 3.2 Hz, 1H), 3.76 (s, 3H), 2.77 (dp, J = 5.4, 3.4, 2.2 Hz, 1H), 2.29 (dtd, J = 14.3, 9.8, 4.6 Hz, 1H), 2.15 (dddd, J = 13.7, 6.5, 4.1, 2.8 Hz, 1H), 1.57 (ddtd, J = 32.4, 14.4, 7.4, 5.4 Hz, 2H), 0.76 (t, J = 7.5 Hz, 3H). ^{13}C NMR (126 MHz, $CDCl_3$) δ 170.7, 154.8, 142.8, 140.0, 128.8, 128.2, 126.1, 118.6, 64.8, 57.1, 53.9, 52.8, 41.3, 25.6, 21.8, 11.8 δ HRMS (ESI) *m/z* calculated for $C_{18}H_{21}NO_4$ $[M+H]^+$ 316.1543; found, 316.1540.

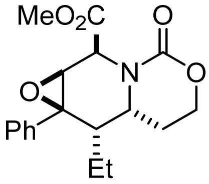

Compound 8. To a flame dried round bottom flask was added Compound **7**, CH_2Cl_2 (0.05 M), and *m*CPBA (2.8 equiv 0.22 mmol). The round bottom flask was fitted with a

reflux condenser and allowed to stir at reflux until TLC indicated complete consumption of the starting material. Once complete, the reaction was quenched with H_2O and diluted with CH_2Cl_2. The biphasic mixture was transferred to a separatory funnel and the layers separated. The aqueous layer was extracted with three portions of CH_2Cl_2 and the combined organics dried over $MgSO_4$. The volatiles were then concentrated under reduced pressure and purified by silica gel flash column chromatography (0 to 60% EtOAc/Hexanes) to yield **8** as an off-white solid (21.7 mg, 83% yield). ^{1}H NMR (500 MHz, $CDCl_3$) δ 7.48 – 7.34 (m, 5H), 5.17 (d, J = 3.6 Hz, 1H), 4.42 – 4.28 (m, 2H), 4.16 (ddd, J = 11.2, 6.1, 2.8 Hz, 1H), 4.06 (dd, J = 3.6, 1.0 Hz, 1H), 3.82 (s, 3H), 2.24 (q, J = 3.5 Hz, 1H), 2.20 – 2.09 (m, 1H), 1.95 (ddt, J = 13.9, 5.9, 2.5 Hz, 1H), 1.48 (dtd, J = 9.7, 7.5, 4.0 Hz, 2H), 0.76 (t, J = 7.6 Hz, 3H). ^{13}C NMR (126 MHz, $CDCl_3$) δ 169.2, 154.6, 137.4, 129.1, 128.9, 127.7, 65.1, 64.8, 56.0, 54.8, 53.0, 50.7, 43.0, 25.5, 19.0, 12.8. HRMS (ESI) *m/z* calculated for $C_{18}H_{21}NO_5$ $[M+H]^+$ 332.1493; found, 332.1484.

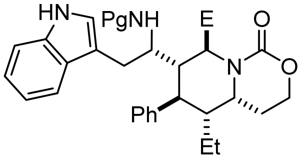

Pg = Boc, E= CO_2Me

Compound 10. In accordance with literature conditions,[27] to a flame dried vial equipped with a Teflon septum was added piperidine **4ca**, Compound **9**, Ir[DF(CF_3)ppy]$_2$(dtbby)PF_6 (.01 equiv, 1.1mg), K_2HPO_4 (1.2 equiv, 20.9 mg), and DMF (0.4 M). The reaction mixture was degassed *via* a stream of nitrogen for 15 minutes and then irradiated with a 26W fluorescent lamp (2 cm from light source). After 36 hours the reaction mixture was diluted with saturated aqueous $NaHCO_3$ solution and extracted with three portions of Et_2O. The combined organics were washed with water and brine, then dried with $MgSO_4$. The crude material was concentrated under reduced pressure and purified by silica gel flash column chromatography (20 to 100% EtOAc/Hex) to yield **10** as a light yellow solid (18.0 mg, 32% yield, 45% yield BRSM). The remainder of the mass balance appeared to be a Boc deprotected-**10** with no other products or diastereomer detected *via* NMR. ^{1}H NMR (500 MHz, $CDCl_3$) δ 7.86 (s, 1H), 7.32 (d, J = 7.9 Hz, 1H), 7.24 (d, J = 8.1 Hz, 1H), 7.19 (t, J = 7.4 Hz, 2H), 7.14 (d, J = 7.1 Hz, 1H), 7.12 – 7.07

(m, 2H), 6.94 (t, J = 7.5 Hz, 1H), 6.59 (d, J = 2.4 Hz, 1H), 5.31 – 5.28 (m, 1H), 4.49 – 4.42 (m, 1H), 4.36 (td, J = 11.2, 4.3 Hz, 1H), 4.22 – 4.13 (m, 1H), 4.14 – 4.04 (m, 1H), 3.85 (bs, 1H), 3.73 (s, 3H), 2.90 (dd, J = 15.1, 7.2 Hz, 1H), 2.86 – 2.72 (m, 2H), 2.49 (dd, J = 12.6, 9.8 Hz, 1H), 1.90 (m, 2H), 1.75 (td, J = 13.1, 12.1, 5.6 Hz, 1H), 1.63 (bs, 1H), 1.42 (s, 9H), 1.07 – 0.97 (m, 1H), 0.92 – 0.80 (m, 1H), 0.67 (t, J = 7.4 Hz, 3H). ^{13}C NMR (126 MHz, $CDCl_3$) δ 173.2, 156.5, 154.2, 141.8, 136.1, 129.0, 128.6, 127.6, 126.7, 122.1, 121.8, 119.3, 118.9, 112.1, 110.8, 78.8, 66.7, 53.9, 53.0, 52.7, 50.8, 49.4, 43.1, 40.6, 29.9, 28.6, 22.3, 22.0, 11.6. HRMS (ESI) *m/z* calculated for $C_{33}H_{41}N_3O_6$ $[M+H]^+$ 576.3068; found, 576.3063.

Transfer of chirality experiments

The enantioenriched bicyclic aziridine (*S,R*)**-2a** was synthesized as described above and its enantiopurity determined by SFC/MS. SFC/MS analyses were performed on a Waters ACQUITY UPC^2 equipped with ACQUITY UPC^2 PDA and ACQUITY QDa Detector. A CHIRALPAK IC-3 column (3mm ID × 150 mm L, 3 µm PS) was used for enantioselective separations. The eluent was a mixture (97:3 CO_2/ iPrOH) with a flow rate of 0.8 mL/min at 40 °C with an ABPR at 2200 psi.

Supplementary Figure 1. Racemic standard for (*S,R*)-**2a.**

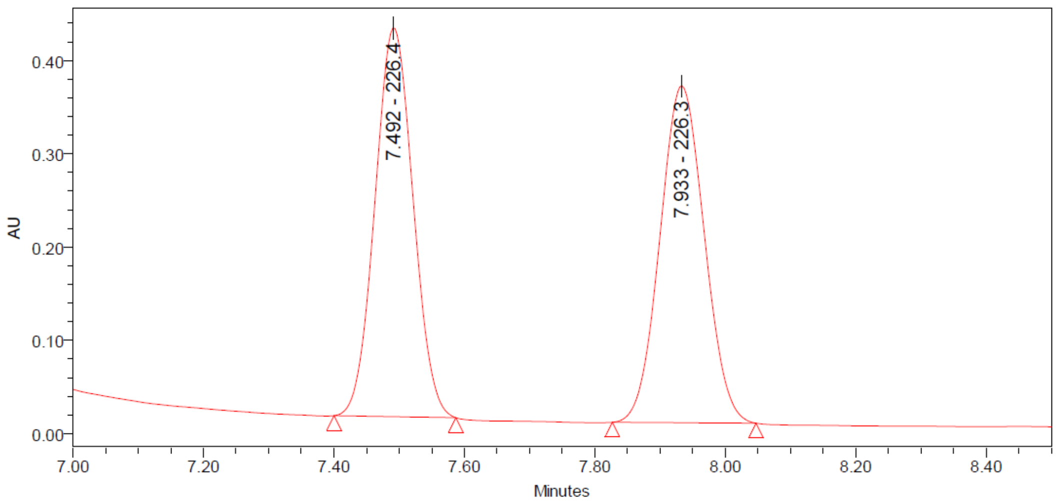

Peak #	Retention Time	Area	% Area
1	7.492	1694026	49.8%
2	7.933	1704523	50.2%

Supplementary Figure 2. Scalemic aziridine (*S,R*)-**2a** utilized in the synthesis of (*R,R,R*)-**4aa.**

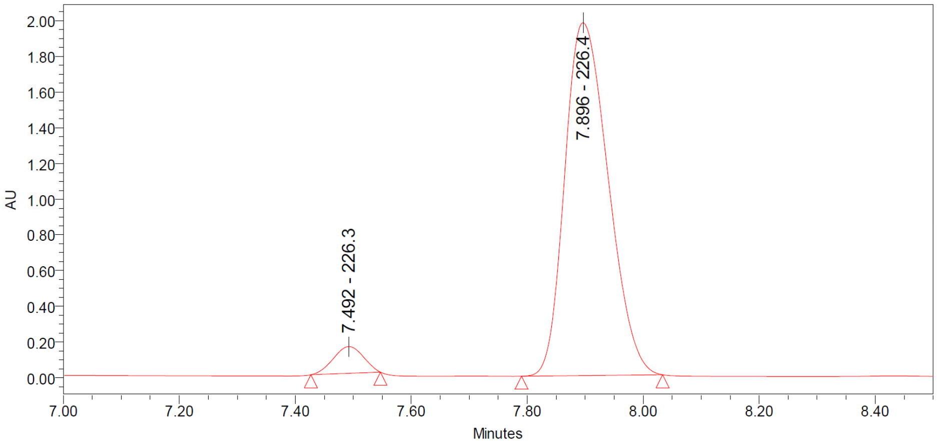

Peak #	Retention Time	Area	% Area
1	7.492	605058	5.5%
2	7.896	10330501	94.5%

Supplementary Figure 3. Scalemic aziridine (*S,R*)-**2a** utilized in the synthesis of **7b**.

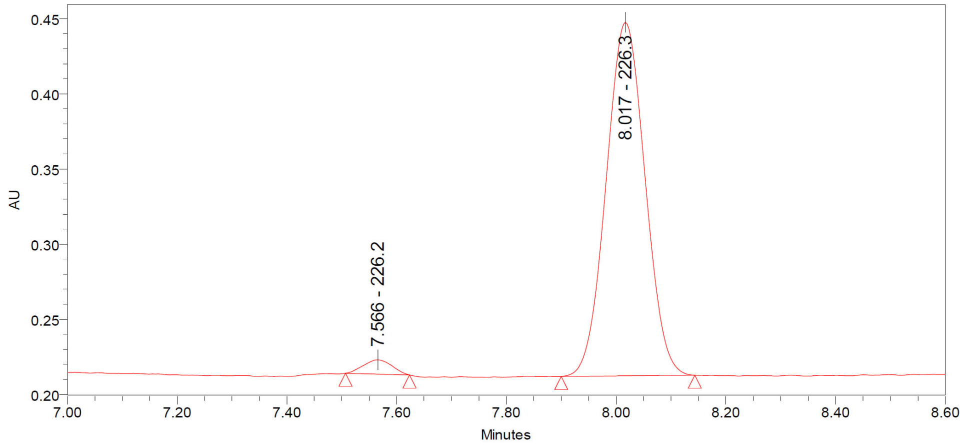

Peak #	Retention Time	Area	% Area

1	7.566	40759	2.89%
2	8.017	1367360	97.11%

HPLC conditions – chromatograms of piperidine products were acquired on a Shimadzu Prominence HPLC equipped with a Chiracel AD-H column. Flow rate: 1.00 mL/min.; Oven temp 25.0 °C for compound **4a**, (*rac*)-**4a**, and scalemic (*S,R*)-**4a**; Solvent: 5:95 *i*PrOH:hexane, increasing to 30:70 *i*PrOH:hexane over 30 minutes. The eluent was held at the more polar composition for another 20 minutes to complete the analysis. Detector: UV @ 254 nm:

Supplementary Figure 4. Racemic piperidine (*R,R,R*)-**4aa.**

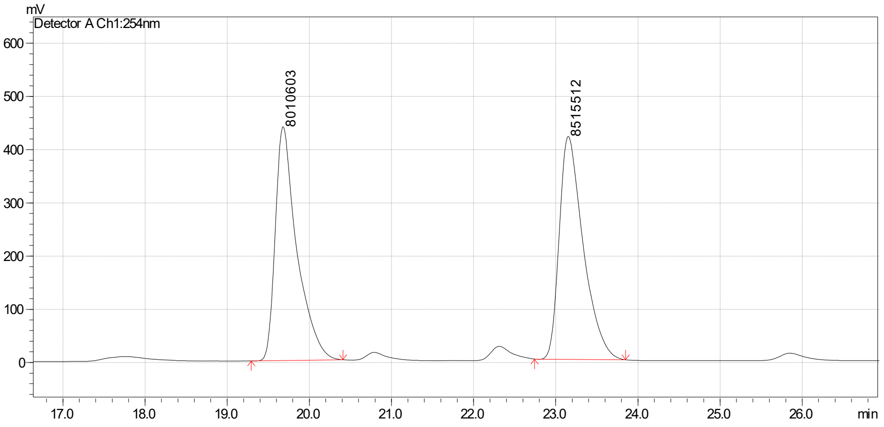

Peak #	Retention Time	Area	% Area
1	19.676	8010603	48.5%
2	23.148	8515512	51.5%

Supplementary Figure 5. Scalemic piperidine (*R,R,R*)-**4aa.**

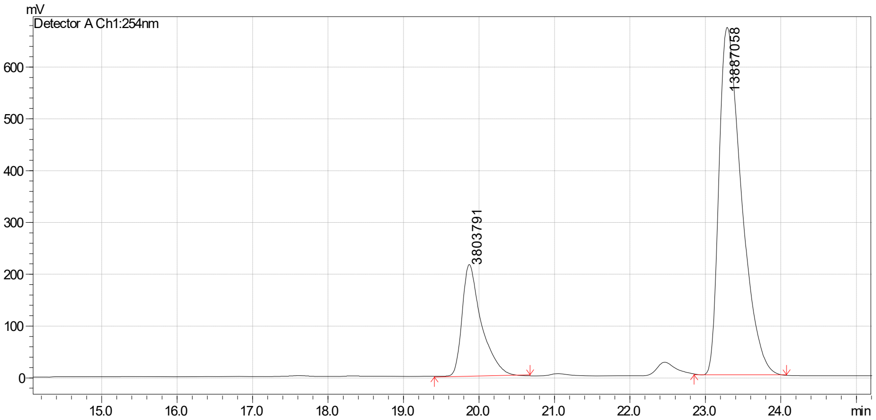

Peak #	Retention Time	Area	% Area
1	19.870	3803791	21.5%
2	23.218	13887058	78.5%

Piperidine analogues of select biomolecules

The following molecules shown are synthetic intermediates accessed in pursuit of **7b** and **8b**, as well as characterization data regarding **7b** and **8b**. All intermediates not shown are known compounds and were synthesized according to published literature procedures and characterized therein.[8-10]

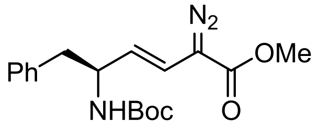

Compound 11a. Following the general procedure provided in Section **IV** the reaction to furnish diazoester **11a** was conducted on 2.50 mmol scale utilizing 10 equiv of *p*-ABSA and 2.6 equiv of DBU. The product was purified by silica gel flash column chromatography (30% Et_2O/pentane) to yield **11a** as a red oil (741.1 mg, 86% yield). ^1H NMR (500 MHz, $CDCl_3$) δ 7.22 (td, *J* = 7.2, 6.3, 1.4 Hz, 2H), 7.18 – 7.12 (m, 1H), 7.09 (dd, *J* = 7.0, 1.8 Hz, 2H), 5.92 – 5.73 (m, 1H), 5.26 (dd, *J* = 15.8, 5.0 Hz, 1H), 4.45 (s, 2H), 3.72 (s, 3H), 2.78 (d, *J* = 5.7 Hz, 2H), 1.33 (s, 9H). ^{13}C NMR (126 MHz, $CDCl_3$) δ

164.5, 154.0, 136.1, 128.4, 127.4, 125.6, 123.2, 112.5, 52.0, 51.1, 40.9, 27.3. HRMS (ESI) *m/z* calculated for $C_{18}H_{23}N_3O_4$ $[M+NH_4]^+$ 363.2027; found, 363.2025.

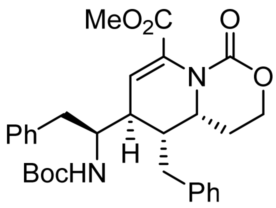

Compound 12a. The general procedure was followed, utilizing (*S,R*)-**2a** as the substrate. The carbene transfer to furnish **12a** was conducted on 0.1 mmol scale. The product was purified by silica gel flash chromatography (0 to 30% EtOAc/CH_2Cl_2, 10% increments) to yield **12a** as a white solid (24.4 mg, 47% yield, >19:1 *dr*). ^{1}H NMR (500 MHz, $CDCl_3$) δ 7.25 – 7.18 (m, 3H), 7.16 – 7.13 (m, 3H), 7.03 (d, *J* = 7.2 Hz, 2H), 6.78 (dd, *J* = 6.5, 2.9 Hz, 2H), 6.00 (d, *J* = 4.3 Hz, 1H), 4.41 – 4.34 (m, 2H), 4.25 (d, *J* = 10.1 Hz, 1H), 4.02 (dd, *J* = 10.4, 8.3 Hz, 1H), 3.81 (s, 3H), 3.76 (q, *J* = 5.7 Hz, 1H), 2.91 – 2.82 (m, 1H), 2.58 (qd, *J* = 14.1, 6.9 Hz, 2H), 2.36 – 2.19 (m, 3H), 2.13 (dd, *J* = 14.2, 8.3 Hz, 1H), 1.36 (s, 9H). ^{13}C NMR (126 MHz, $CDCl_3$) δ 164.0, 155.4, 152.6, 138.5, 136.7, 133.6, 129.1, 128.9, 128.7, 128.6, 126.6, 126.5, 79.8, 64.7, 55.9, 53.9, 52.5, 42.8, 39.2, 38.7, 32.5, 29.7, 28.2, 25.7. HRMS (ESI) *m/z* calculated for $C_{30}H_{36}N_2O_6$ $[2M+NH_4]^+$ 1058.5485; found, 1058.5497.

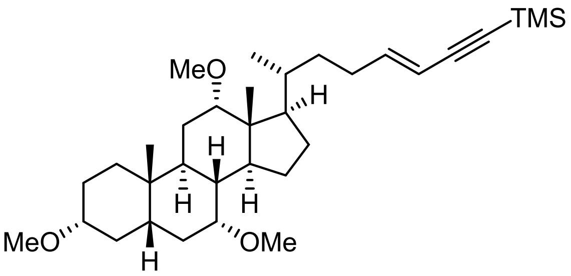

Compound S1. In accordance with literature conditions,[8] the reaction to furnish ene-yne **S1** was conducted on a 4.94 mmol scale. The product was purified *via* automated flash chromatography using a 0-10% gradient of EtOAc/CH_2Cl_2 to yield **S1** as clear viscous oil. This was isolated as an inseparable mixture of *trans* and *cis*-isomers, with only the major *trans* product characterized (1.32 g, 51%, 4:1 *trans:cis*). ^{1}H NMR (500 MHz,

C_6D_6) δ 6.27 (dt, *J* = 15.8, 7.1 Hz, 1H), 5.60 (dt, *J* = 16.0, 1.5 Hz, 1H), 3.26 (s, 3H), 3.23 (s, 1H), 3.07 (s, 1H), 3.05 (s, 3H), 3.02 (s, 3H), 2.96 (tt, *J* = 11.0, 4.2 Hz, 1H), 2.56 – 2.40 (m, 3H), 2.31 (td, *J* = 12.3, 4.4 Hz, 1H), 2.07 (q, *J* = 9.8 Hz, 1H), 1.98 (tt, *J* = 11.3, 5.6 Hz, 1H), 1.88 – 1.70 (m, 7H), 1.57 – 1.48 (m, 3H), 1.48 – 1.35 (m, 5H), 1.30 (ddq, *J* = 10.2, 6.8, 2.9 Hz, 2H), 1.26 – 1.18 (m, 1H), 1.13 – 1.01 (m, 4H), 0.58 (s, 3H), 0.42 (s, 3H), 0.22 (s, 9H). ^{13}C NMR (126 MHz,C_6D_6) δ 147.0, 110.3, 105.3, 92.8, 82.0, 81.0, 77.4, 55.8, 55.4, 46.8, 46.6, 42.9, 42.4, 40.2, 35.8, 35.5, 35.3, 35.2, 30.1, 28.4, 28.1, 27.8, 27.6, 23.7, 23.3, 22.3, 17.8, 12.7, 0.2. HRMS (ESI) *m/z* calculated for $C_{33}H_{56}O_3Si$ $[M+NH_4]^+$ 546.4337; found, 546.336.

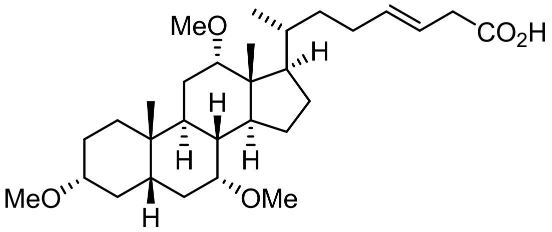

Compound S2. In accordance with literature conditions,[11] the hydroboration oxidation reaction of ene-yne **S1** to furnish carboxylic acid **S2** was conducted on a 2.5 mmol scale. The product was purified by acid-base extraction to remove the cyclohexanol impurity and was isolated as a white solid. The crude material was utilized in the next reaction (0.834 g, 67%, 6:1 *trans:cis*). Overintegration in the ^{1}H NMR is attributed to the *cis*-isomer. ^{1}H NMR (500 MHz, C_6D_6) δ 5.60 – 5.51 (m, 1H), 5.39 (dt, *J* = 14.6, 6.7 Hz, 1H), 3.28 (d, *J* = 3.0 Hz, 1H), 3.27 (s, 3H), 3.08 (s, 3H), 3.06 – 3.04 (m, 1H), 3.04 (s, 3H), 2.97 (tt, *J* = 11.5, 4.6 Hz, 1H), 2.81 (d, *J* = 6.9 Hz, 2H), 2.56 – 2.42 (m, 2H), 2.33 (td, *J* = 12.3, 4.2 Hz, 1H), 2.17 (q, *J* = 9.8 Hz, 1H), 2.12 – 2.03 (m, 1H), 1.97 – 1.70 (m, 8H), 1.70 – 1.60 (m, 3H), 1.61 – 1.28 (m, 9H), 1.28 – 1.19 (m, 2H), 1.13 – 1.02 (m, 3H), 0.98 (d, *J* = 6.6 Hz, 3H), 0.87 (s, 3H), 0.65 (s, 3H). ^{13}C NMR (126 MHz, C_6D_6) δ 176.6, 135.8, 121.6, 82.2, 81.2, 77.6, 56.0, 55.6, 55.5, 47.1, 46.8, 43.0, 42.6, 40.4, 38.0, 36.0, 35.9, 35.9, 35.4, 35.4, 29.8, 28.6, 28.3, 28.1, 27.8, 24.0, 23.4, 22.5, 18.1, 13.0. HRMS (ESI) *m/z* calculated for $C_{30}H_{50}O_5$ $[M^-H]^-$ 489.3586; found, 489.3584.

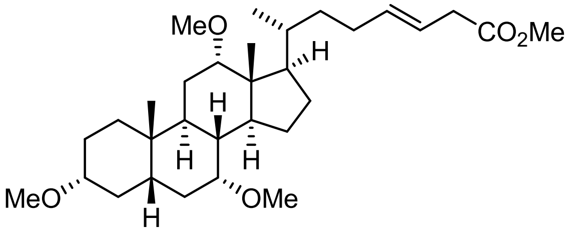

Compound S3. Carboxylic acid **S2** (1.70 mmol, 1 equiv) was dissolved in 10 mL of MeOH (0.6 M), 0.1 mL of H_2SO_4 (1.88 mmol, 1.1 equiv) was added, and the mixture allowed to stir until TLC indicated completion. The reaction was poured into a solution of saturated aqueous $NaHCO_3$ and extracted with three portions of Et_2O. The combined organics were then dried over $MgSO_4$ and concentrated *in vacuo*. The crude material was purified *via* silica column flash chromatography using a 0-20% gradient of EtOAc/Pentanes to yield methyl ester **S3** as clear viscous oil (0.53 g, 65%, 6:1 *trans:cis*). ^{1}H NMR (500 MHz, C_6D_6) δ 5.65 (dt, *J* = 14.6, 6.9 Hz, 1H), 5.45 (dd, *J* = 14.7, 7.3 Hz, 1H), 3.32 (s, 3H), 3.28 (s, 2H), 3.27 (s, 3H), 3.07 (s, 3H), 3.03 (s, 3H), 2.97 (tt, *J* = 11.1, 3.6 Hz, 1H), 2.88 (d, *J* = 6.9 Hz, 2H), 2.48 (dt, *J* = 19.8, 12.6 Hz, 2H), 2.33 (td, *J* = 12.4, 4.1 Hz, 1H), 2.24 – 2.06 (m, 2H), 2.00 – 1.68 (m, 6H), 1.64 (ddt, *J* = 12.3, 9.7, 4.5 Hz, 2H), 1.59 – 1.32 (m, 6H), 1.28 – 1.12 (m, 3H), 1.12 – 1.01 (m, 2H), 0.98 (d, *J* = 6.5 Hz, 3H), 0.87 (s, 3H), 0.65 (s, 3H), ^{13}C NMR (126 MHz,C_6D_6) δ 171.75, 135.2, 122.1, 82.0, 81.0, 77.4, 55.8, 55.4, 55.3, 51.2, 47.0, 46.6, 42.9, 42.4, 40.2, 4.2, 35.9, 35.8, 35.7, 35.2, 29.6, 28.4, 28.1, 29.0 27.5, 23.7, 23.2, 22.3, 17.9, 12.7. HRMS (ESI) *m/z* calculated for $C_{31}H_{52}O_5$ $[M+NH_4]^+$ 522.4153; found, 522.4152.

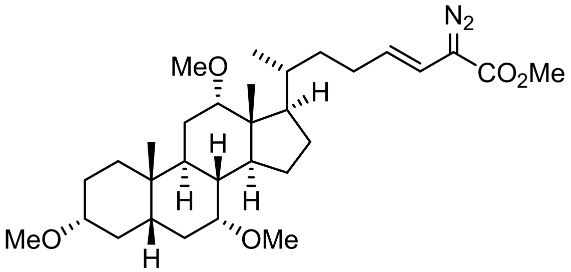

Compound 11b. Following the general preparation provided in section **IV** the reaction to furnish diazoester **11b** was conducted on methyl ester **S3** (1.0 mmol) utilizing 10 equiv of

p-ABSA, and 2.6 equiv of DBU. The product was purified by silica gel flash column chromatography (30% Et_2O/pentane) to yield **11b** as an orange solid (0.200 g, 38%, all *trans*). *Note: if compound was left of vacuum for longer than a period of 1-2 h the sample began to noticeably decompose. For best results, the diazoester compound should be freshly made.* ^{1}H NMR (500 MHz, C_6D_6). ^{1}H NMR δ 5.86 (d, *J* = 15.8 Hz, 1H), 5.21 (dt, *J* = 14.8, 6.9 Hz, 1H), 3.33 (s, 3H), 3.28 (d, *J* = 2.9 Hz, 1H), 3.26 (s, 3H), 3.09 (s, 3H), 3.06 (d, *J* = 4.1 Hz, 1H), 3.04 (s, 3H), 2.97 (tt, *J* = 11.1, 4.1 Hz, 1H), 2.56 – 2.41 (m, 3H), 2.33 (td, *J* = 12.3, 4.4 Hz, 1H), 2.21 – 2.10 (m, 2H), 2.05 – 1.93 (m, 1H), 1.90 – 1.70 (m, 6H), 1.65 (m, 3H), 1.57 – 1.34 (m, 5H), 1.30 – 1.17 (m, 2H), 1.18 – 1.02 (m, 2H), 0.98 (d, *J* = 6.5 Hz, 3H), 0.97 – 0.88 (m, 1H), 0.87 (s, 3H) 0.64 (s, 3H). ^{13}C NMR (126 MHz, C_6D_6) δ 165.4, 129.7, 126.6, 112.0, 82.0, 81.0, 77.4, 55.0, 55.4., 55.3, 51.5, 46.8, 46.6, 42.9, 42.4, 40.2, 36.2, 35.8, 35.7, 35.2, 30.0, 28.4, 28.1, 28.0, 27.5, 23.7, 23.2, 22.3, 18.0, 12.70. HRMS (ESI) *m/z* calculated for $C_{31}H_{50}N_2O_5$ $[M+NH_4]^+$; 548.4058 found, 548.4053.

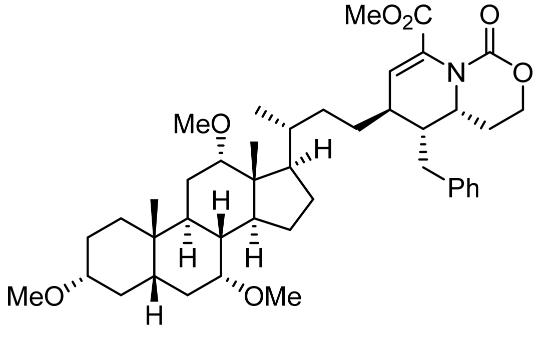

Compound 12b. The general procedure was followed, utilizing (*S,R*)-**2a** as the substrate. The carbene transfer to furnish **12b** was conducted on 0.12 mmol scale. The product was purified by silica gel flash chromatography (0 to 50% EtOAc/Hexanes, 10% increments) to yield **12b** as a white solid (44.0 mg, 52% yield, >19:1 *dr*). ^{1}H NMR (500 MHz, CD_3CN) δ 7.29 – 7.16 (m, 3H), 7.16 – 7.05 (m, 2H), 5.91 (dd, *J* = 4.5, 1.3 Hz, 1H), 4.37 – 4.11 (m, 2H), 3.77 (td, *J* = 9.0, 8.1, 1.5 Hz, 1H), 3.62 (s, 3H), 3.24 (t, *J* = 2.8 Hz, 1H), 3.17 (s, 3H), 3.12 (s, 3H), 3.12 (s, 3H), 3.06 (q, *J* = 3.1 Hz, 1H), 2.91 – 2.84 (m, 1H), 2.83 – 2.79 (m, 1H), 2.78 – 2.72 (m, 1H), 2.46 – 2.36 (m, 1H), 2.17 (dddd, *J* = 15.2, 8.7, 5.1, 2.6 Hz, 1H), 1.92 – 1.80 (m, 8H), 1.76 – 1.48 (m, 6H), 1.45 – 1.32 (m, 3H), 1.28 –

0.83 (m, 8H), 0.82 – 0.81 (m, 1H), 0.80 (s, 3H), 0.69 (d, J = 6.6 Hz, 3H), 0.52 (s, 3H). ^{13}C NMR (126 MHz, CD_3CN) δ 165.2, 153.6, 140.8, 131.9, 130.2, 129.6, 129.3, 125.4, 82.8, 81.1, 78.2, 69.3, 66.0, 56.1, 56.0, 55.5, 54.0, 52.6, 47.5, 46.8, 43.4, 43.3, 42.6, 40.3, 37.4, 36.3, 35.8, 35.5, 33.8, 33.2, 32.9, 31.7, 28.6, 28.5, 28.1, 27.7, 26.3, 23.8, 23.1, 22.6, 20.0, 18.1, 12.7. HRMS (ESI) *m/z* calculated for $C_{43}H_{67}N_2O_7$ $[M+NH_4]^+$ 723.4943; found, 723.4945.

One-pot nitrene-carbene transfer reaction

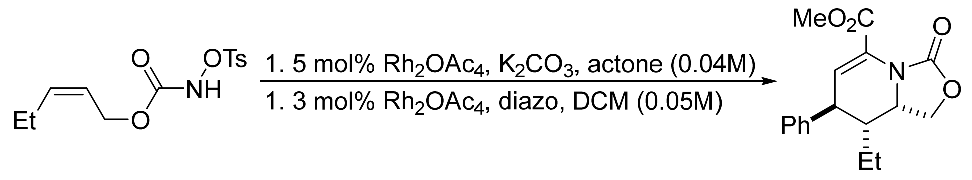

A flame-dried, round bottom flask was placed under nitrogen and charged with Rh_2OAc_4 (4.20 mg, 0.01 mmol, 0.05 equiv), *N*-tosyl carbamate (50.00 mg, 0.17 mmol, 1 equiv), K_2CO_3 (0.184 g, 1.17 mmol, 7 equiv) and acetone (4.2 mL, 0.04 M).[3] The mixture was stirred vigorously at room temperature for 16 h (TLC was used to determine when the reaction was complete). Once the starting material was consumed, the reaction mixture was passed through a plug of celite to remove K_2CO_3. The filter pad was washed with EtOAc and the volatiles removed under reduced pressure. The resulting oil was diluted with CH_2Cl_2 (3.5 mL, 0.05 M) and Rh_2OAc_4 (2.2 mg, 0.01 mmol, 0.03 equiv). To this mixture was added a solution of the diazoester compound (40.5 mg, 0.20 mmol, 1.2 equiv) dropwise over 2 h using a syringe pump. The conversion was checked by TLC and NMR after the addition was finished; once the reaction was complete, it was concentrated and loaded directly onto silica for column chromatography (0% to 40% ethyl acetate/hexanes).

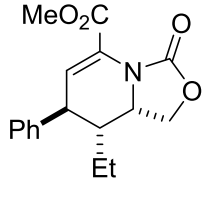

Compound 4ja. Following the general procedure, the nitrene and carbene transfer to furnish **4ja** was conducted on 0.17 mmol scale. The product was purified by silica gel flash column chromatography (0 to 40% EtOAc/Hexanes, 10% increments) to yield **4ja** as a white solid (53.6 mg, 54% yield, >19:1 *dr*). ^{1}H NMR (500 MHz, $CDCl_3$) δ 7.36 (d, J = 7.3 Hz, 2H), 7.28 (d, J = 7.3 Hz, 1H), 7.15 – 7.10 (m, 2H), 6.24 (dd, J = 4.9, 1.1 Hz, 1H), 4.49 (t, J = 8.8 Hz, 1H), 4.16 (dd, J = 9.2, 2.5 Hz, 1H), 3.90 (s, 3H), 3.90 – 3.85 (m, 1H), 3.65 (d, J = 5.0 Hz, 1H), 1.70 – 1.57 (m, 3H), 1.17 (t, J = 3.8 Hz, 3H).^{13}C NMR (126 MHz, $CDCl_3$) δ 163.2, 155.1, 142.5, 130.3, 129.0, 128.4, 127.3, 121.6, 65.4, 52.8, 51.7, 46.1, 41.5, 18.9, 12.3. HRMS (ESI) *m/z* calculated for $C_{17}H_{20}NO_4$ $[M+H]^+$ 302.1387; found, 302.1384.

X-Crystallographic information for 4cc.

A colorless crystal with approximate dimensions 0.20 x 0.15 x 0.11 mm^3 was selected under oil under ambient conditions and attached to the tip of a MiTeGen MicroMount©. The crystal was mounted in a stream of cold nitrogen at 100(1) K and centered in the X-ray beam by using a video camera.

The crystal evaluation and data collection were performed on a Bruker SMART APEXII diffractometer with Cu K_α (λ = 1.54178 Å) radiation and the diffractometer to crystal distance of 4.03 cm .[11]

The initial cell constants were obtained from three series of ω scans at different starting angles. Each series consisted of 41 frames collected at intervals of 0.6º in a 25º range about ω with the exposure time of 10 seconds per frame. The reflections were successfully indexed by an automated indexing routine built in the APEX3 program. The final cell constants were calculated from a set of 9913 strong reflections from the actual data collection.

The data were collected by using the full sphere data collection routine to survey the reciprocal space to the extent of a full sphere to a resolution of 0.81 Å. A total of 24327 data were harvested by collecting 14 sets of frames with 0.5º scans in ω and φ with an exposure time 10 sec per frame. These highly redundant datasets were corrected for Lorentz and polarization effects. The absorption correction was based on fitting a

function to the empirical transmission surface as sampled by multiple equivalent measurements.[12] The crystallographic data for this compound were deposited with CDCC with the deposition number 1921208.

Structure Solution and Refinement

The systematic absences in the diffraction data were consistent for the space groups $P\bar{1}$ and $P1$. The E-statistics strongly suggested the centrosymmetric space group $P\bar{1}$ that yielded chemically reasonable and computationally stable results of refinement.[13-18]

A successful solution by the direct methods provided most non-hydrogen atoms from the E-map. The remaining non-hydrogen atoms were located in an alternating series of least-squares cycles and difference Fourier maps. All non-hydrogen atoms were refined with anisotropic displacement coefficients. All hydrogen atoms were included in the structure factor calculation at idealized positions and were allowed to ride on the neighboring atoms with relative isotropic displacement coefficients.

There are two organic molecules per asymmetric unit. These molecules each have three chiral centers and are enantiomers of each other, therefore the second molecule was labeled with the suffix "A". The chiral centers are C3 - *S*, C4 - *S*, and C5 - *R* for the first molecule, and C3A-*R*, C4A - *R*, and C5A - *S* for the second.

The final least-squares refinement of 437 parameters against 6575 data resulted in residuals R (based on F^2 for $I \geq 2\sigma$) and wR (based on F^2 for all data) of 0.0249 and 0.0659, respectively. The final difference Fourier map was featureless.

Crystal Data for $C_{18}H_{20}BrNO_4$ (M =394.26 g/mol): triclinic, space group $P\bar{1}$ (no. 2), a = 7.2645(4) Å, b = 15.1045(15) Å, c = 17.5158(14) Å, α = 65.499(5)°, β = 84.097(5)°, γ = 82.736(4)°, V = 1732.1(2) Å^3, Z = 4, T = 100.02 K, μ(CuKα) = 3.422 mm^{-1}, $Dcalc$ = 1.512 g/cm^3, 24314 reflections measured (5.554° ≤ 2Θ ≤ 144.264°), 6575 unique (R_{int} = 0.0222, R_{sigma} = 0.0190) which were used in all calculations. The final R_1 was 0.0249 (I > 2σ(I)) and wR_2 was 0.0659 (all data).

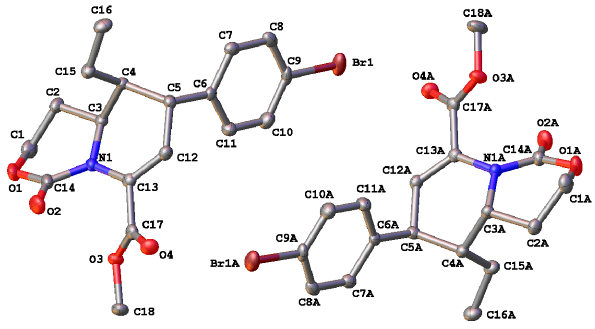

Supplementary Figure 6. A molecular drawing of **4cc** shown with 50% probability ellipsoids. All H atoms are omitted.

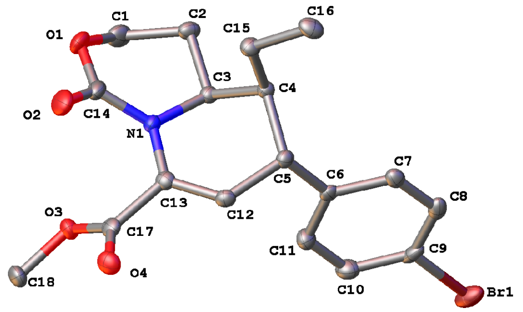

Supplementary Figure 7. A molecular drawing of one organic molecule from **4cc** shown with 50% ellipsoids. The other molecule and all hydrogen atoms are omitted.

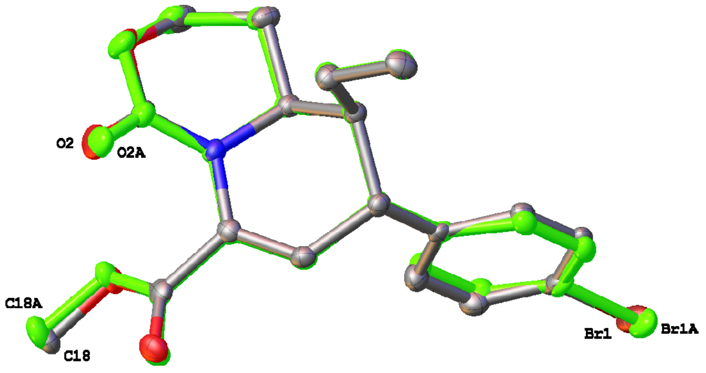

Supplementary Figure 8. A molecular drawing of the overlay of the two molecules from **4cc** shown with 50% ellipsoids. The molecule with suffix "A" was inverted and drawn in green.

Supplementary Table 1. Crystal data and structure refinement for **4cc**.

Identification code	**4cc**
Empirical formula	$C_{18}H_{20}BrNO_4$
Formula weight	394.26
Temperature/K	100.02
Crystal system	triclinic
Space group	$P\overline{1}$
a/Å	7.2645(4)
b/Å	15.1045(15)
c/Å	17.5158(14)
α/°	65.499(5)
β/°	84.097(5)
γ/°	82.736(4)
Volume/Å^3	1732.1(2)
Z	4
ρ_{calc}g/cm^3	1.512
μ/mm^{-1}	3.422
F(000)	808.0
Crystal size/mm^3	0.203 × 0.154 × 0.108
Radiation	CuKα (λ = 1.54178)
2Θ range for data collection/°	5.554 to 144.264
Index ranges	$-8 \leq h \leq 8$, $-18 \leq k \leq 18$, $-21 \leq l \leq 20$
Reflections collected	24314
Independent reflections	6575 [R_{int} = 0.0222, R_{sigma} = 0.0190]
Data/restraints/parameters	6575/0/437
Goodness-of-fit on F^2	1.021
Final R indexes [I>=2σ (I)]	R_1 = 0.0249, wR_2 = 0.0643
Final R indexes [all data]	R_1 = 0.0273, wR_2 = 0.0659
Largest diff. peak/hole / e Å^{-3}	0.43/-0.48

Supplementary Table 2. Fractional Atomic Coordinates (×10^4) and Equivalent Isotropic Displacement Parameters (Å^2×10^3) for **4cc**. U_{eq} is defined as 1/3 of of the trace of the orthogonalised U_{IJ} tensor.

**Atom**	***x***	***y***	***z***	**U(eq)**
Br1	9180.5(2)	7210.2(2)	3815.2(2)	31.44(7)
O1	4946.8(19)	3932.8(9)	9846.0(8)	30.2(3)
O2	2007.7(19)	4038.4(10)	9558.1(8)	32.6(3)

O3	1629.4(15)	6100.0(8)	8450.2(7)	20.3(2)
O4	-379.0(15)	5671.9(9)	7795.9(8)	23.6(2)
N1	4161.7(18)	4562.9(9)	8462.8(8)	15.9(3)
C1	6790(3)	4214.5(14)	9488.7(12)	28.5(4)
C2	7341(2)	3818.3(11)	8830.1(10)	19.5(3)
C3	6022(2)	4326.4(10)	8117.3(9)	14.8(3)
C4	5798(2)	3744.1(10)	7595.1(9)	14.6(3)
C5	4599(2)	4395.7(11)	6844.7(10)	15.6(3)
C6	5718(2)	5096.4(11)	6110.5(9)	15.3(3)
C7	6793(2)	4754.8(12)	5562.9(10)	18.3(3)
C8	7821(2)	5370.2(13)	4879.3(10)	20.5(3)
C9	7790(2)	6343.3(12)	4748.5(10)	21.4(3)
C10	6753(2)	6703.9(12)	5281.3(11)	24.1(3)
C11	5711(2)	6080.7(12)	5956.7(11)	21.4(3)
C12	2954(2)	4916.6(11)	7130.5(10)	16.3(3)
C13	2762(2)	4961.2(11)	7878.7(10)	16.1(3)
C14	3608(3)	4161.8(12)	9302.0(10)	23.0(3)
C15	4937(2)	2775.4(11)	8111.7(10)	18.1(3)
C16	5174(2)	2088.5(12)	7660.7(11)	22.7(3)
C17	1140(2)	5586.8(11)	8049.4(10)	18.2(3)
C18	158(2)	6769.9(12)	8588.1(11)	23.9(3)
Br1A	473.8(2)	7575.1(2)	6100.8(2)	29.44(6)
O1A	5567.6(17)	11359.3(9)	157.8(7)	26.3(3)
O2A	8357.2(16)	11257.7(9)	608.3(8)	27.2(3)
O3A	8705.3(15)	9150.7(9)	1472.8(7)	22.2(2)
O4A	10433.5(15)	9341.1(9)	2379.6(8)	22.4(2)
N1A	6034.0(17)	10544.1(9)	1573.4(8)	15.2(3)
C1A	3710(2)	11034.1(14)	397.9(11)	25.5(4)
C2A	2907(2)	11291.0(12)	1115.7(10)	19.9(3)
C3A	4092(2)	10723.6(11)	1872.1(9)	14.7(3)
C4A	4117(2)	11224.1(11)	2478.6(10)	15.0(3)
C5A	5210(2)	10523.5(11)	3250.8(10)	15.6(3)
C6A	4029(2)	9796.7(11)	3933.4(10)	15.5(3)
C7A	3065(2)	10052.1(12)	4556.7(10)	18.1(3)
C8A	2005(2)	9398.9(12)	5200.6(10)	20.1(3)
C9A	1886(2)	8486.6(12)	5212.9(10)	20.5(3)
C10A	2800(2)	8216.6(12)	4598.3(11)	23.6(3)
C11A	3877(2)	8871.0(12)	3966.3(10)	20.9(3)

C12A	6926(2)	10025.4(11)	2987.6(10)	16.1(3)
C13A	7304(2)	10074.8(11)	2210.6(10)	15.2(3)
C14A	6753(2)	11073.6(12)	776.0(10)	19.8(3)
C15A	4970(2)	12210.3(11)	2061.5(10)	19.1(3)
C16A	4586(2)	12812.5(12)	2589.5(11)	22.9(3)
C17A	9007(2)	9502.2(11)	2032.2(10)	17.0(3)
C18A	10271(3)	8570.5(15)	1279.2(12)	31.4(4)

Supplementary Table 3. Anisotropic Displacement Parameters ($Å^2 \times 10^3$) for **4cc**. The Anisotropic displacement factor exponent takes the form: $-2\pi^2[h^2a^{*2}U_{11}+2hka^*b^*U_{12}+\ldots]$.

Atom	U_{11}	U_{22}	U_{33}	U_{23}	U_{13}	U_{12}
Br1	20.46(10)	36.89(11)	20.84(11)	3.93(8)	0.54(7)	-2.80(7)
O1	42.3(8)	32.2(7)	12.7(6)	-8.2(5)	-4.7(5)	7.3(6)
O2	35.5(7)	31.8(7)	20.0(7)	-3.4(5)	10.3(5)	-1.5(5)
O3	19.6(6)	21.6(5)	20.3(6)	-11.0(5)	0.7(4)	3.3(4)
O4	16.3(6)	25.1(6)	27.9(7)	-10.0(5)	1.5(5)	-1.6(4)
N1	19.8(7)	14.7(6)	12.5(7)	-5.3(5)	-0.6(5)	0.0(5)
C1	37.5(10)	28.8(9)	22.5(10)	-13.2(8)	-13.0(7)	4.2(7)
C2	24.3(8)	16.6(7)	18.4(8)	-7.2(6)	-8.1(6)	1.3(6)
C3	17.3(7)	12.7(7)	14.3(8)	-5.4(6)	-1.7(6)	-0.5(5)
C4	16.3(7)	13.4(7)	14.0(8)	-5.7(6)	-1.3(6)	-0.3(5)
C5	18.0(7)	15.8(7)	13.7(8)	-6.8(6)	-1.9(6)	-1.1(6)
C6	16.8(7)	17.1(7)	11.4(8)	-5.0(6)	-4.9(6)	0.7(6)
C7	17.1(8)	20.6(7)	19.4(9)	-10.5(7)	-5.3(6)	2.5(6)
C8	14.4(7)	32.0(9)	16.1(8)	-11.5(7)	-3.1(6)	1.9(6)
C9	16.2(8)	27.3(8)	11.5(8)	1.5(6)	-2.1(6)	-1.8(6)
C10	26.4(9)	18.2(8)	23.4(9)	-4.6(7)	-1.7(7)	-0.7(6)
C11	26.2(8)	19.2(8)	19.4(9)	-9.5(7)	0.1(6)	0.2(6)
C12	14.9(7)	16.1(7)	16.6(8)	-4.9(6)	-3.0(6)	-0.9(6)
C13	16.4(7)	13.8(7)	16.4(8)	-4.3(6)	0.6(6)	-3.1(6)
C14	35.1(10)	16.5(7)	13.5(8)	-4.1(6)	0.2(7)	2.8(7)
C15	21.4(8)	14.6(7)	18.4(8)	-6.6(6)	-1.8(6)	-2.1(6)
C16	24.1(8)	16.3(7)	30.9(10)	-12.3(7)	-6.2(7)	0.4(6)
C17	19.7(8)	16.5(7)	13.8(8)	-2.2(6)	3.7(6)	-3.2(6)
C18	22.9(8)	25.0(8)	22.1(9)	-11.2(7)	2.5(6)	6.5(7)
Br1A	24.27(10)	29.54(10)	21.57(11)	1.44(8)	4.20(7)	-3.43(7)

O1A	24.9(6)	33.7(7)	13.3(6)	-2.9(5)	-2.0(5)	-1.3(5)
O2A	20.9(6)	33.7(7)	19.8(6)	-4.2(5)	4.3(5)	-5.3(5)
O3A	18.5(6)	29.1(6)	22.8(6)	-16.1(5)	-1.6(4)	4.7(5)
O4A	14.4(5)	27.6(6)	24.5(6)	-10.5(5)	-2.0(4)	0.9(4)
N1A	14.2(6)	16.7(6)	13.4(7)	-5.1(5)	-1.3(5)	-0.2(5)
C1A	25.0(9)	31.8(9)	18.1(9)	-7.3(7)	-6.6(7)	-2.6(7)
C2A	18.3(8)	20.4(7)	18.5(9)	-5.0(7)	-5.3(6)	0.0(6)
C3A	12.7(7)	15.0(7)	14.8(8)	-4.7(6)	-0.6(6)	-1.0(5)
C4A	15.0(7)	13.7(7)	15.2(8)	-5.0(6)	0.3(6)	-1.0(5)
C5A	16.9(7)	16.1(7)	15.3(8)	-8.2(6)	-0.6(6)	-1.3(6)
C6A	16.8(7)	16.5(7)	12.5(8)	-5.3(6)	-4.1(6)	1.8(6)
C7A	16.7(7)	20.7(7)	18.9(8)	-10.5(7)	-3.5(6)	1.5(6)
C8A	15.8(8)	29.7(8)	15.2(8)	-10.4(7)	-1.9(6)	2.6(6)
C9A	16.8(7)	24.3(8)	12.3(8)	0.5(6)	0.0(6)	-2.4(6)
C10A	28.9(9)	17.4(7)	22.4(9)	-6.2(7)	0.5(7)	-2.6(6)
C11A	28.0(9)	18.8(7)	16.8(8)	-9.1(7)	1.4(6)	-1.2(6)
C12A	15.3(7)	15.9(7)	16.4(8)	-5.4(6)	-3.3(6)	-1.2(6)
C13A	14.6(7)	14.0(7)	16.5(8)	-5.2(6)	-0.7(6)	-2.8(5)
C14A	22.7(8)	19.7(7)	14.5(8)	-5.3(6)	-0.5(6)	0.1(6)
C15A	20.3(8)	14.6(7)	21.7(9)	-6.8(6)	1.4(6)	-3.1(6)
C16A	24.0(8)	17.2(7)	28.8(9)	-11.0(7)	0.0(7)	-2.5(6)
C17A	16.7(8)	16.5(7)	14.9(8)	-3.8(6)	2.0(6)	-2.7(6)
C18A	25.0(9)	43.3(11)	31.6(11)	-24.8(9)	-1.2(7)	10.2(8)

Supplementary Table 4. Bond Lengths for **4cc**.

**Atom**	**Atom**	**Length/Å**	**Atom**	**Atom**	**Length/Å**
Br1	C9	1.9044(16)	Br1A	C9A	1.9026(16)
O1	C1	1.455(2)	O1A	C1A	1.456(2)
O1	C14	1.344(2)	O1A	C14A	1.345(2)
O2	C14	1.211(2)	O2A	C14A	1.211(2)
O3	C17	1.339(2)	O3A	C17A	1.3397(19)
O3	C18	1.4493(18)	O3A	C18A	1.4460(19)
O4	C17	1.204(2)	O4A	C17A	1.203(2)
N1	C3	1.4865(19)	N1A	C3A	1.4857(19)
N1	C13	1.411(2)	N1A	C13A	1.411(2)
N1	C14	1.375(2)	N1A	C14A	1.376(2)

C1	C2	1.505(2)	C1A	C2A	1.506(2)
C2	C3	1.527(2)	C2A	C3A	1.529(2)
C3	C4	1.5397(19)	C3A	C4A	1.541(2)
C4	C5	1.550(2)	C4A	C5A	1.549(2)
C4	C15	1.539(2)	C4A	C15A	1.541(2)
C5	C6	1.520(2)	C5A	C6A	1.520(2)
C5	C12	1.504(2)	C5A	C12A	1.505(2)
C6	C7	1.397(2)	C6A	C7A	1.398(2)
C6	C11	1.396(2)	C6A	C11A	1.393(2)
C7	C8	1.387(2)	C7A	C8A	1.389(2)
C8	C9	1.388(2)	C8A	C9A	1.383(2)
C9	C10	1.382(2)	C9A	C10A	1.384(2)
C10	C11	1.389(2)	C10A	C11A	1.388(2)
C12	C13	1.332(2)	C12A	C13A	1.333(2)
C13	C17	1.500(2)	C13A	C17A	1.500(2)
C15	C16	1.529(2)	C15A	C16A	1.530(2)

Supplementary Table 5. Bond Angles for **4cc**.

**Atom**	**Atom**	**Atom**	**Angle/°**	**Atom**	**Atom**	**Atom**	**Angle/°**
C14	O1	C1	116.72(13)	C14A	O1A	C1A	116.81(13)
C17	O3	C18	114.96(13)	C17A	O3A	C18A	115.05(13)
C13	N1	C3	115.39(12)	C13A	N1A	C3A	115.21(12)
C14	N1	C3	123.84(13)	C14A	N1A	C3A	123.50(13)
C14	N1	C13	117.64(13)	C14A	N1A	C13A	117.29(13)
O1	C1	C2	108.38(14)	O1A	C1A	C2A	108.84(13)
C1	C2	C3	108.09(13)	C1A	C2A	C3A	108.60(13)
N1	C3	C2	109.59(12)	N1A	C3A	C2A	109.40(12)
N1	C3	C4	108.98(12)	N1A	C3A	C4A	108.62(12)
C2	C3	C4	114.87(12)	C2A	C3A	C4A	114.86(12)
C3	C4	C5	108.70(12)	C3A	C4A	C5A	108.87(12)
C15	C4	C3	113.10(12)	C3A	C4A	C15A	112.74(12)
C15	C4	C5	110.39(12)	C15A	C4A	C5A	110.17(12)
C6	C5	C4	112.77(12)	C6A	C5A	C4A	113.53(12)
C12	C5	C4	110.44(12)	C12A	C5A	C4A	111.01(12)
C12	C5	C6	112.15(12)	C12A	C5A	C6A	111.96(12)
C7	C6	C5	119.48(13)	C7A	C6A	C5A	119.60(13)

C11	C6	C5	122.34(14)	C11A	C6A	C5A	122.16(14)
C11	C6	C7	118.18(15)	C11A	C6A	C7A	118.24(15)
C8	C7	C6	121.48(15)	C8A	C7A	C6A	121.19(14)
C7	C8	C9	118.77(14)	C9A	C8A	C7A	118.97(14)
C8	C9	Br1	119.94(13)	C8A	C9A	Br1A	119.52(12)
C10	C9	Br1	118.80(13)	C8A	C9A	C10A	121.27(15)
C10	C9	C8	121.25(15)	C10A	C9A	Br1A	119.21(13)
C9	C10	C11	119.25(15)	C9A	C10A	C11A	119.12(15)
C10	C11	C6	121.05(15)	C10A	C11A	C6A	121.20(15)
C13	C12	C5	124.16(14)	C13A	C12A	C5A	124.02(14)
N1	C13	C17	118.67(13)	N1A	C13A	C17A	118.14(13)
C12	C13	N1	122.20(14)	C12A	C13A	N1A	122.02(14)
C12	C13	C17	118.44(14)	C12A	C13A	C17A	119.31(14)
O1	C14	N1	116.33(15)	O1A	C14A	N1A	115.82(14)
O2	C14	O1	120.18(15)	O2A	C14A	O1A	119.95(15)
O2	C14	N1	123.45(16)	O2A	C14A	N1A	124.18(15)
C16	C15	C4	112.58(13)	C16A	C15A	C4A	112.46(13)
O3	C17	C13	111.84(13)	O3A	C17A	C13A	111.54(13)
O4	C17	O3	124.29(14)	O4A	C17A	O3A	124.39(14)
O4	C17	C13	123.62(14)	O4A	C17A	C13A	123.98(14)

Supplementary Table 6. Torsion Angles for **4cc**.

A	B	C	D	Angle/˚	A	B	C	D	Angle/˚
Br1	C9	C10	C11	179.03(12)	Br1A	C9A	C10A	C11A	-177.94(13)
O1	C1	C2	C3	65.08(17)	O1A	C1A	C2A	C3A	-63.65(17)
N1	C3	C4	C5	62.67(15)	N1A	C3A	C4A	C5A	-62.53(15)
N1	C3	C4	C15	-60.29(16)	N1A	C3A	C4A	C15A	60.04(16)
N1	C13	C17	O3	33.16(19)	N1A	C13A	C17A	O3A	-28.31(18)
N1	C13	C17	O4	-152.32(15)	N1A	C13A	C17A	O4A	155.02(15)
C1	O1	C14	O2	-172.58(15)	C1A	O1A	C14A	O2A	174.46(15)
C1	O1	C14	N1	5.4(2)	C1A	O1A	C14A	N1A	-3.1(2)
C1	C2	C3	N1	-31.90(17)	C1A	C2A	C3A	N1A	29.20(17)
C1	C2	C3	C4	-154.95(14)	C1A	C2A	C3A	C4A	151.62(13)
C2	C3	C4	C5	-173.95(13)	C2A	C3A	C4A	C5A	174.63(12)
C2	C3	C4	C15	63.09(17)	C2A	C3A	C4A	C15A	-62.81(17)
C3	N1	C13	C12	15.2(2)	C3A	N1A	C13A	C12A	-16.7(2)

C3	N1	C13	C17	-155.16(13)	C3A	N1A	C13A	C17A	154.89(13)
C3	N1	C14	O1	31.4(2)	C3A	N1A	C14A	O1A	-35.5(2)
C3	N1	C14	O2	-150.75(15)	C3A	N1A	C14A	O2A	147.12(16)
C3	C4	C5	C6	82.22(15)	C3A	C4A	C5A	C6A	-85.40(15)
C3	C4	C5	C12	-44.14(16)	C3A	C4A	C5A	C12A	41.77(16)
C3	C4	C15	C16	-165.30(13)	C3A	C4A	C15A	C16A	166.54(13)
C4	C5	C6	C7	79.54(17)	C4A	C5A	C6A	C7A	-88.25(16)
C4	C5	C6	C11	-100.75(16)	C4A	C5A	C6A	C11A	92.25(17)
C4	C5	C12	C13	12.2(2)	C4A	C5A	C12A	C13A	-9.0(2)
C5	C4	C15	C16	72.67(16)	C5A	C4A	C15A	C16A	-71.62(16)
C5	C6	C7	C8	179.30(13)	C5A	C6A	C7A	C8A	-178.58(14)
C5	C6	C11	C10	179.84(15)	C5A	C6A	C11A	C10A	179.60(15)
C5	C12	C13	N1	3.8(2)	C5A	C12A	C13A	N1A	-5.2(2)
C5	C12	C13	C17	174.16(13)	C5A	C12A	C13A	C17A	-176.63(13)
C6	C5	C12	C13	-114.50(16)	C6A	C5A	C12A	C13A	119.07(16)
C6	C7	C8	C9	0.8(2)	C6A	C7A	C8A	C9A	-1.0(2)
C7	C6	C11	C10	-0.4(2)	C7A	C6A	C11A	C10A	0.1(2)
C7	C8	C9	Br1	-179.87(11)	C7A	C8A	C9A	Br1A	178.95(12)
C7	C8	C9	C10	-0.3(2)	C7A	C8A	C9A	C10A	0.0(2)
C8	C9	C10	C11	-0.5(3)	C8A	C9A	C10A	C11A	1.0(3)
C9	C10	C11	C6	0.9(3)	C9A	C10A	C11A	C6A	-1.1(3)
C11	C6	C7	C8	-0.4(2)	C11A	C6A	C7A	C8A	0.9(2)
C12	C5	C6	C7	-155.02(14)	C12A	C5A	C6A	C7A	145.08(14)
C12	C5	C6	C11	24.7(2)	C12A	C5A	C6A	C11A	-34.4(2)
C12	C13	C17	O3	-137.58(15)	C12A	C13A	C17A	O3A	143.49(14)
C12	C13	C17	O4	36.9(2)	C12A	C13A	C17A	O4A	-33.2(2)
C13	N1	C3	C2	-174.83(12)	C13A	N1A	C3A	C2A	176.42(12)
C13	N1	C3	C4	-48.35(16)	C13A	N1A	C3A	C4A	50.34(16)
C13	N1	C14	O1	-169.58(13)	C13A	N1A	C14A	O1A	168.14(13)
C13	N1	C14	O2	8.3(2)	C13A	N1A	C14A	O2A	-9.3(2)
C14	O1	C1	C2	-53.16(18)	C14A	O1A	C1A	C2A	51.90(19)
C14	N1	C3	C2	-15.38(19)	C14A	N1A	C3A	C2A	19.59(19)
C14	N1	C3	C4	111.10(16)	C14A	N1A	C3A	C4A	-106.49(15)
C14	N1	C13	C12	-145.56(15)	C14A	N1A	C13A	C12A	141.66(15)
C14	N1	C13	C17	44.06(19)	C14A	N1A	C13A	C17A	-46.77(18)
C15	C4	C5	C6	-153.20(12)	C15A	C4A	C5A	C6A	150.50(12)
C15	C4	C5	C12	80.45(15)	C15A	C4A	C5A	C12A	-82.33(15)
C18	O3	C17	O4	1.7(2)	C18A	O3A	C17A	O4A	-2.1(2)

C18	O3	C17	C13	176.17(13)	C18A	O3A	C17A	C13A	-178.72(14)

Supplementary Table 7. Hydrogen Atom Coordinates (Å×10^4) and Isotropic Displacement Parameters (Å^2×10^3) for **4cc**.

Atom	*x*	*y*	*z*	U(eq)
H1A	7694.31	3945.88	9934.18	34
H1B	6778.64	4935.32	9233.16	34
H2A	7256.98	3103.7	9074.83	23
H2B	8638.67	3945.22	8614.22	23
H3	6520.46	4956.78	7728.48	18
H4	7057.81	3590.82	7365.83	17
H5	4099.91	3950.08	6640.75	19
H7	6820.13	4085.97	5660.7	22
H8	8533.77	5130.09	4507.54	25
H10	6751.56	7370.32	5186.97	29
H11	4981.9	6328.44	6319.66	26
H12	1991.81	5233.86	6753.53	20
H15A	5528.49	2444.7	8656.79	22
H15B	3595.97	2916.75	8229.19	22
H16A	4480.04	2383.55	7149.07	34
H16B	4698.41	1463.56	8032.27	34
H16C	6494.16	1977.91	7511.54	34
H18A	-362.55	7207.62	8050.55	36
H18B	663.15	7156.25	8835.88	36
H18C	-820.49	6398.89	8970.98	36
H1AA	3769.66	10318.84	571.25	31
H1AB	2910.46	11356.94	-85.84	31
H2AA	1608.5	11117.81	1261.01	24
H2AB	2905	12001.95	952.44	24
H3A	3589.83	10075.52	2195.75	18
H4A	2807.07	11344.63	2676.95	18
H5A	5644.95	10939.71	3503.2	19
H7A	3137.38	10683.73	4539.54	22
H8A	1371.44	9576.3	5625.93	24
H10A	2690.24	7591.4	4608.81	28
H11A	4524.02	8684.5	3548.41	25
H12A	7792.48	9652.85	3400.38	19

H15C	6330.3	12088.65	1973.43	23
H15D	4454.12	12590.37	1503.81	23
H16D	5102.92	13443.41	2287.79	34
H16E	5167.09	12458.8	3128	34
H16F	3241.29	12921.15	2690.8	34
H18D	9972.18	8402.48	825.27	47
H18E	10540.24	7970.33	1779.94	47
H18F	11359.84	8945.51	1101.32	47

Computational Methods.

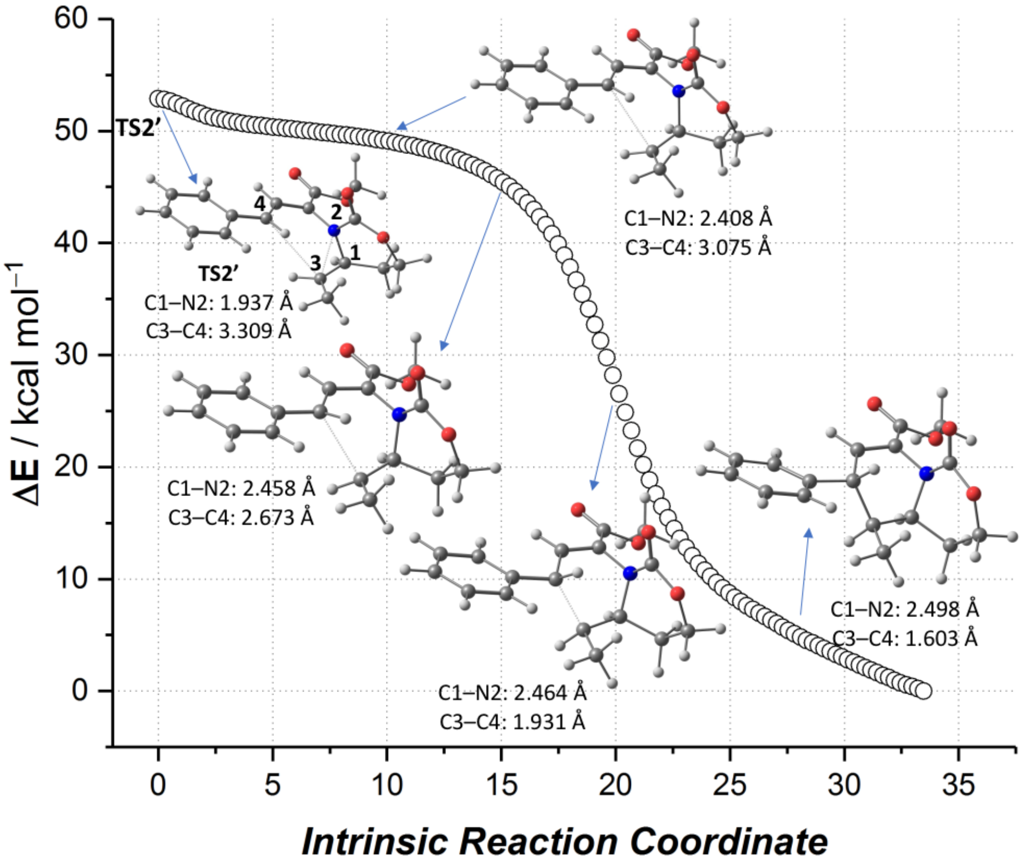

Supplementary Figure 9. Computed (SCM(CH_2Cl_2)-B3LYP-D3/def2-SVP level) intrinsic reaction coordinate for the transformation of **TS2'** into **4ba**.

Computational details

All the calculations reported in this paper were performed with the Gaussian 09 suite of programs.[19] Electron correlation was partially taken into account using the hybrid functional usually denoted as B3LYP[20-22] in conjunction with the D3 dispersion correction suggested by Grimme et al.[23] using the standard double- ζ quality def2-SVP[24] basis set for all atoms. The SMD continuum model was used to model the effects of the solvent (CH_2Cl_2). This level is denoted SMD(CH_2Cl_2)-B3LYP-D3/def2-SVP. Geometries were fully optimized in solution without any geometry or symmetry constraints. Reactants, intermediates, and products were characterized by frequency calculations,[25] and have positive definite Hessian matrices. Transition structures (TS's) show only one negative eigenvalue in their diagonalized force constant matrices, and their associated eigenvectors were confirmed to correspond to the motion along the reaction coordinate under consideration using the Intrinsic Reaction Coordinate (IRC) method.[26] Frequency calculations were also used to determine the difference between the potential (E) and Gibbs (G) energies, G −E, which contains the zero-point, thermal, and entropy energies. Potential energies were refined, E_{sol}, by means of single point (SP) calculations at the same level with a larger basis set, def2-TZVPP, [24] where all elements were described with a triple-ζ + polarization quality basis set. This level is denoted SMD(CH_2Cl_2)-B3LYP-D3/def2-TZVPP//SMD(CH_2Cl_2)-B3LYP-D3/def2-SVP. The ΔG and $\Delta G^{\ddagger}$ values given in the text were obtained from the Gibbs energy in solution, G_{sol}, which was calculated by adding the thermochemistry corrections, G − E, to the refined SP energies, E_{sol}, i.e., $G_{sol} = E_{sol} + G - E$. Cartesian coordinates (in Å) and total energies (in a.u.) of all the

stationary points discussed in the text. All calculations have been performed at the SMD(CH_2Cl_2)-B3LYP-D3/def2-TZVPP//SMD(CH_2Cl_2)-B3LYP-D3/def2-SVP level.

The coordinates for the relevant computed structures can be found in the Supplementary Data File.

Supplementary Figures.

Supplementary Figure 10. ^{1}H NMR (500 MHz, $CDCl_3$) for precursor to compound **2a**

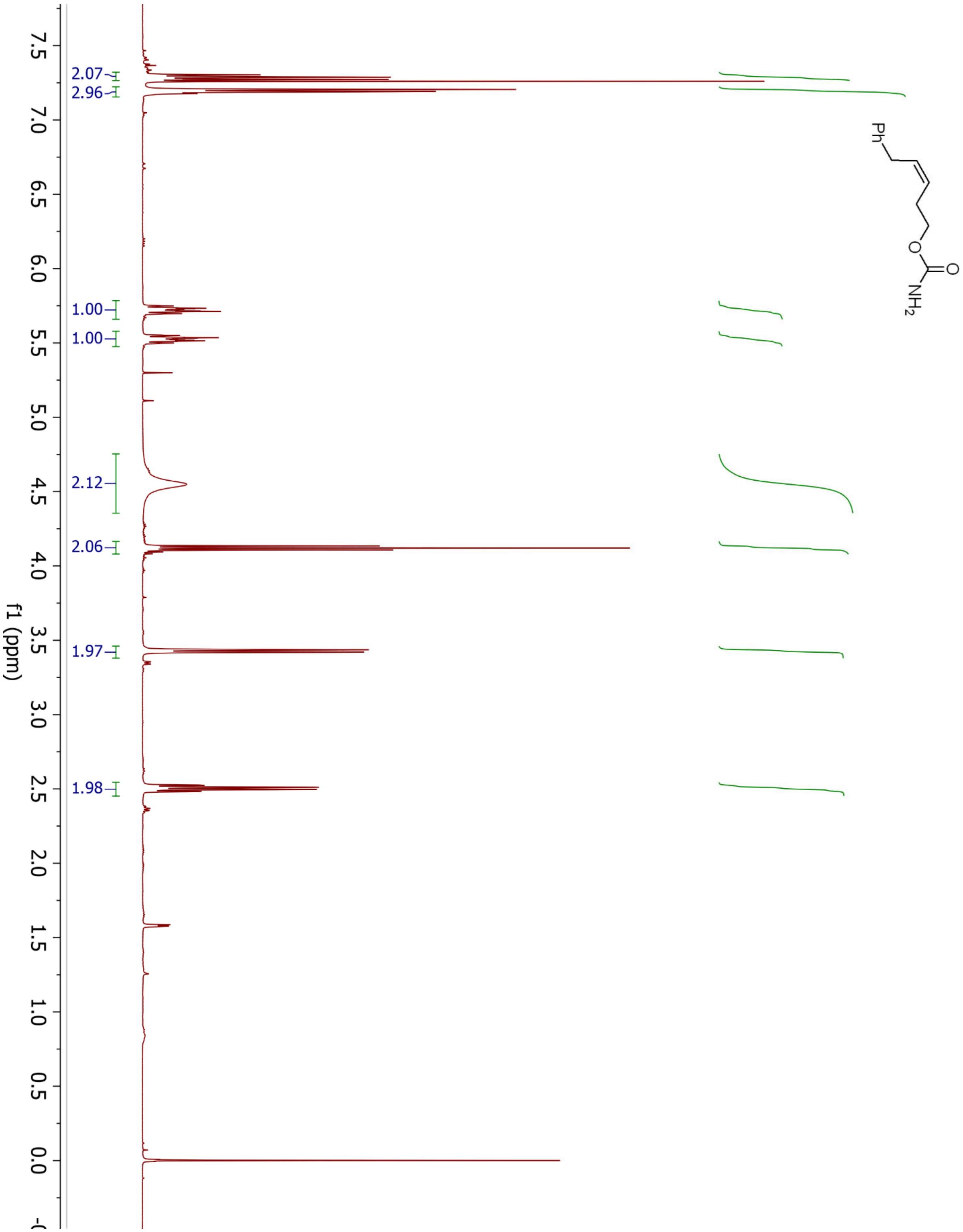

Supplementary Figure 11. ^{13}C NMR (126 MHz, $CDCl_3$) for precursor to compound **2a**

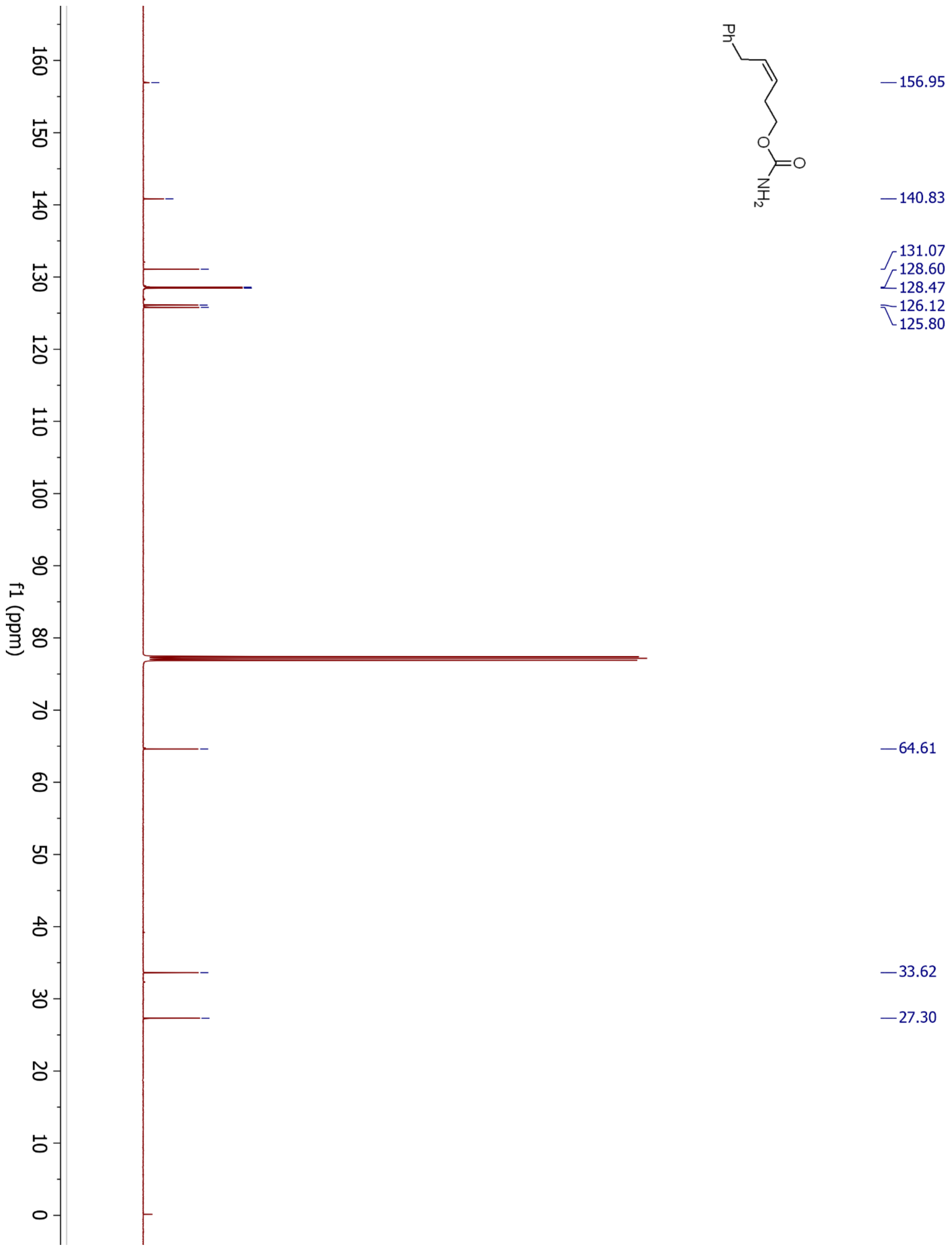

Supplementary Figure 12. ^{1}H NMR (500 MHz, $CDCl_3$) for precursor to compound **2b**

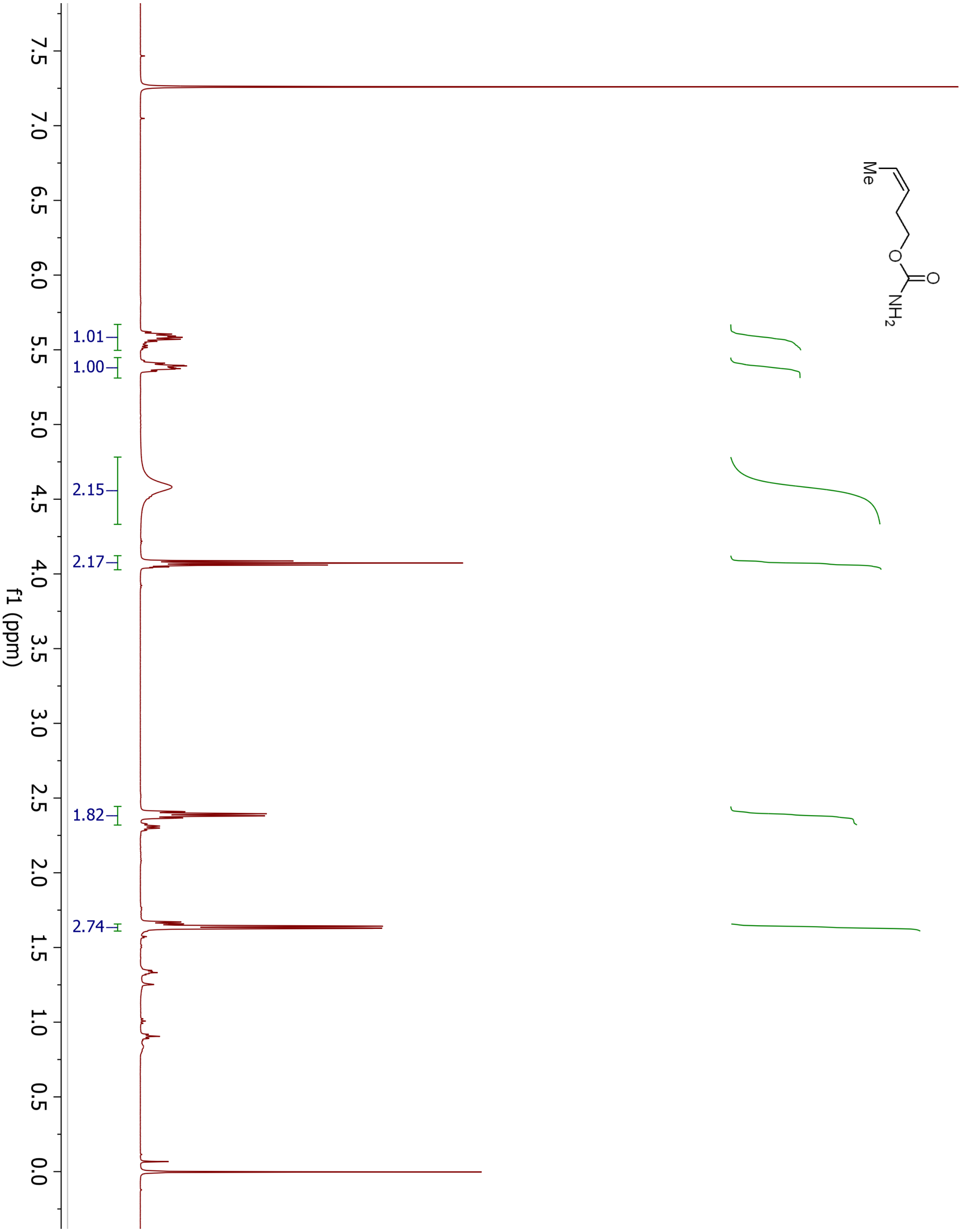

Supplementary Figure 13. ^{13}C NMR (126MHz, $CDCl_3$) for precursor to compound **2b**

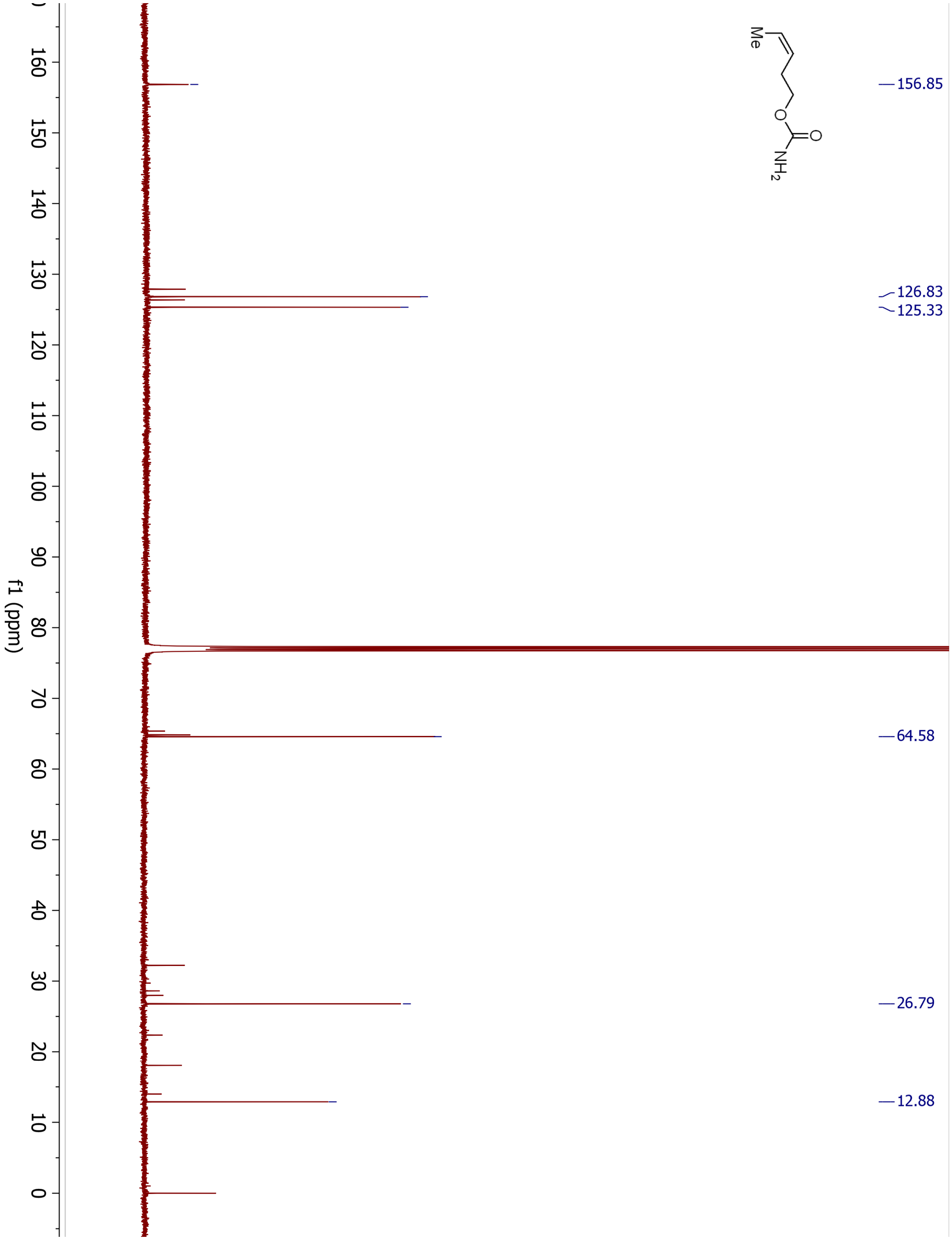

Supplementary Figure 14. ^{1}H NMR (500 MHz, $CDCl_3$) for precursor to compound **2e**

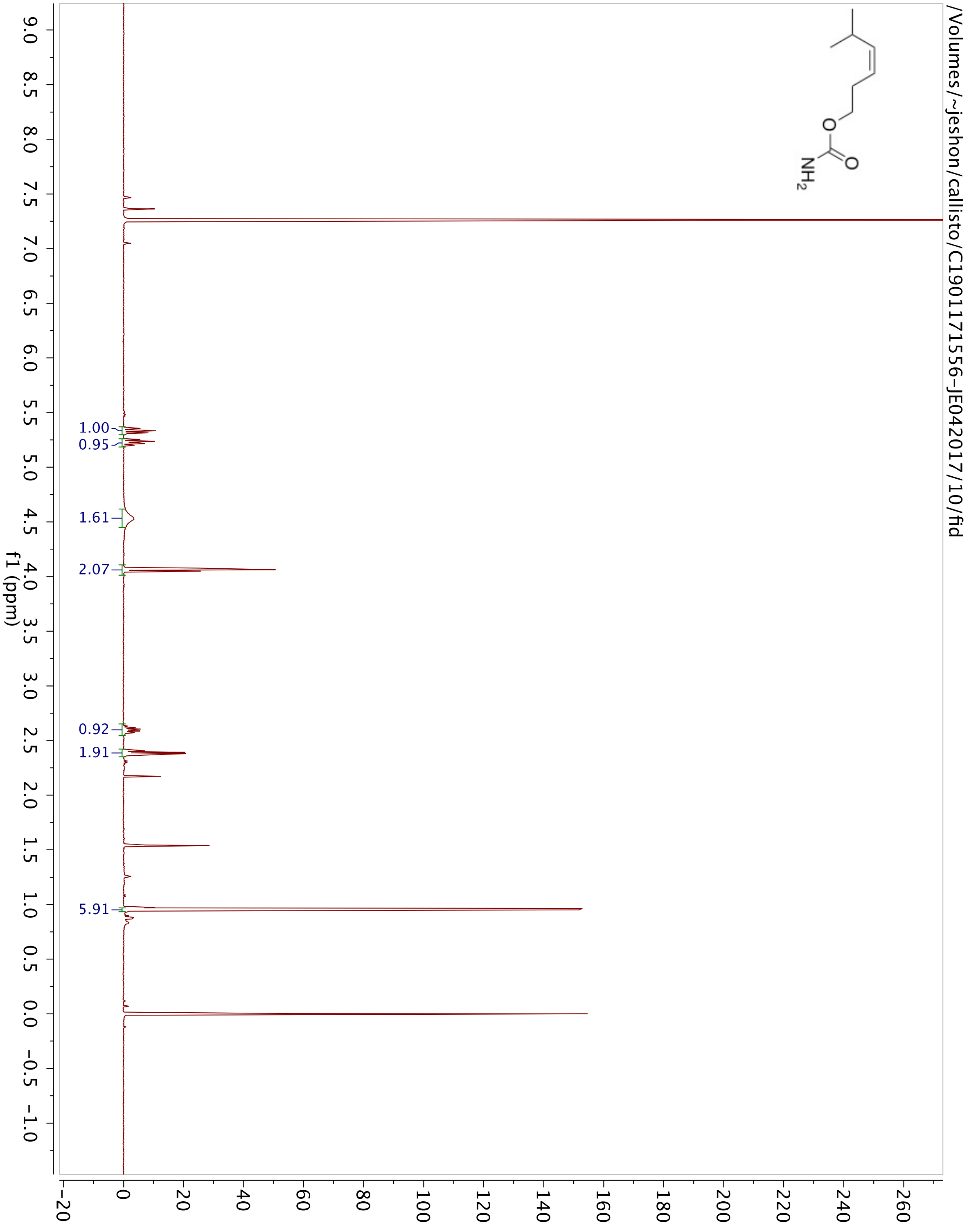

Supplementary Figure 15. ^{13}C NMR (126MHz, $CDCl_3$) for precursor to compound **2e**

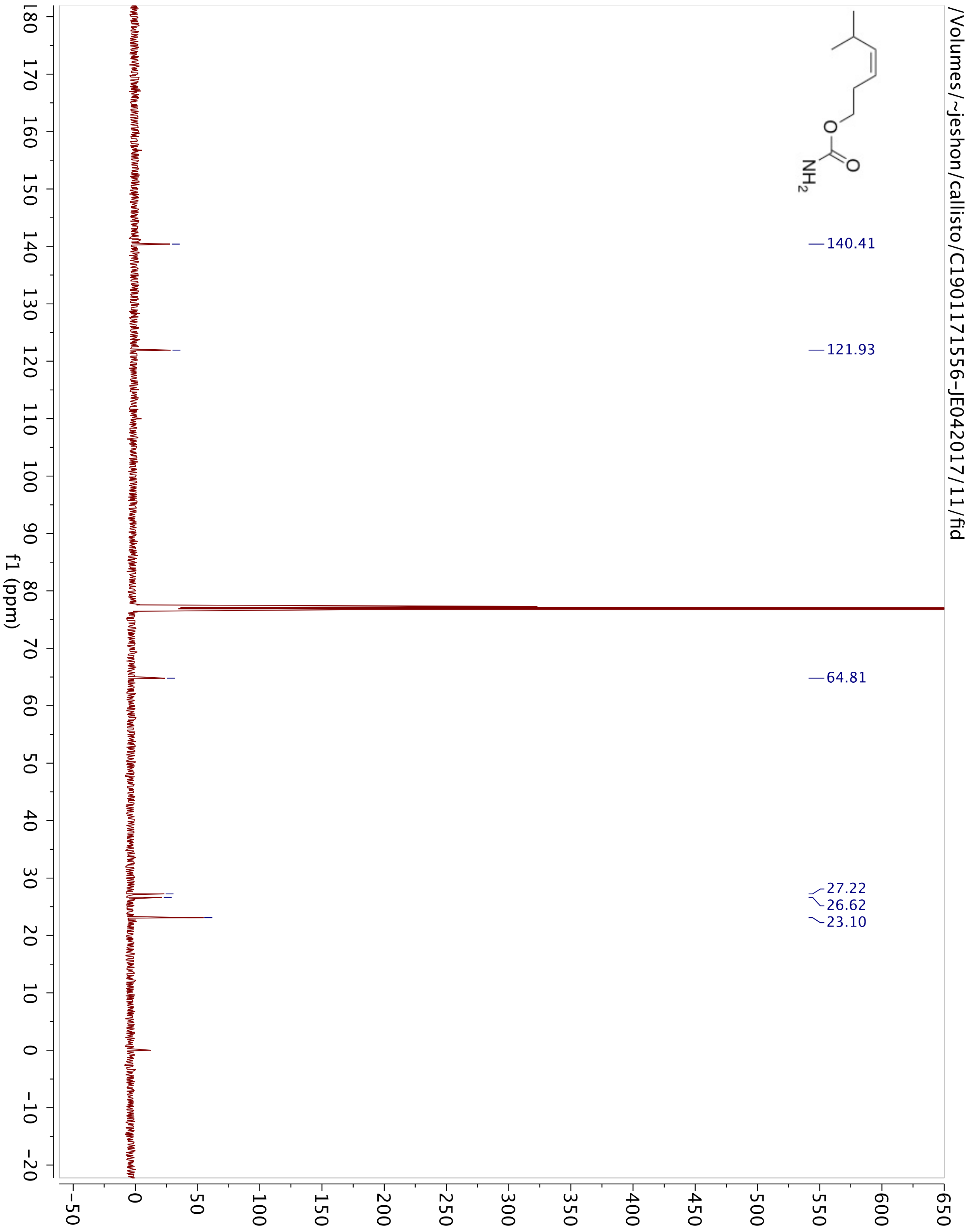

Supplementary Figure 16. ^{1}H NMR (500 MHz, $CDCl_3$) for precursor to compound **2f**

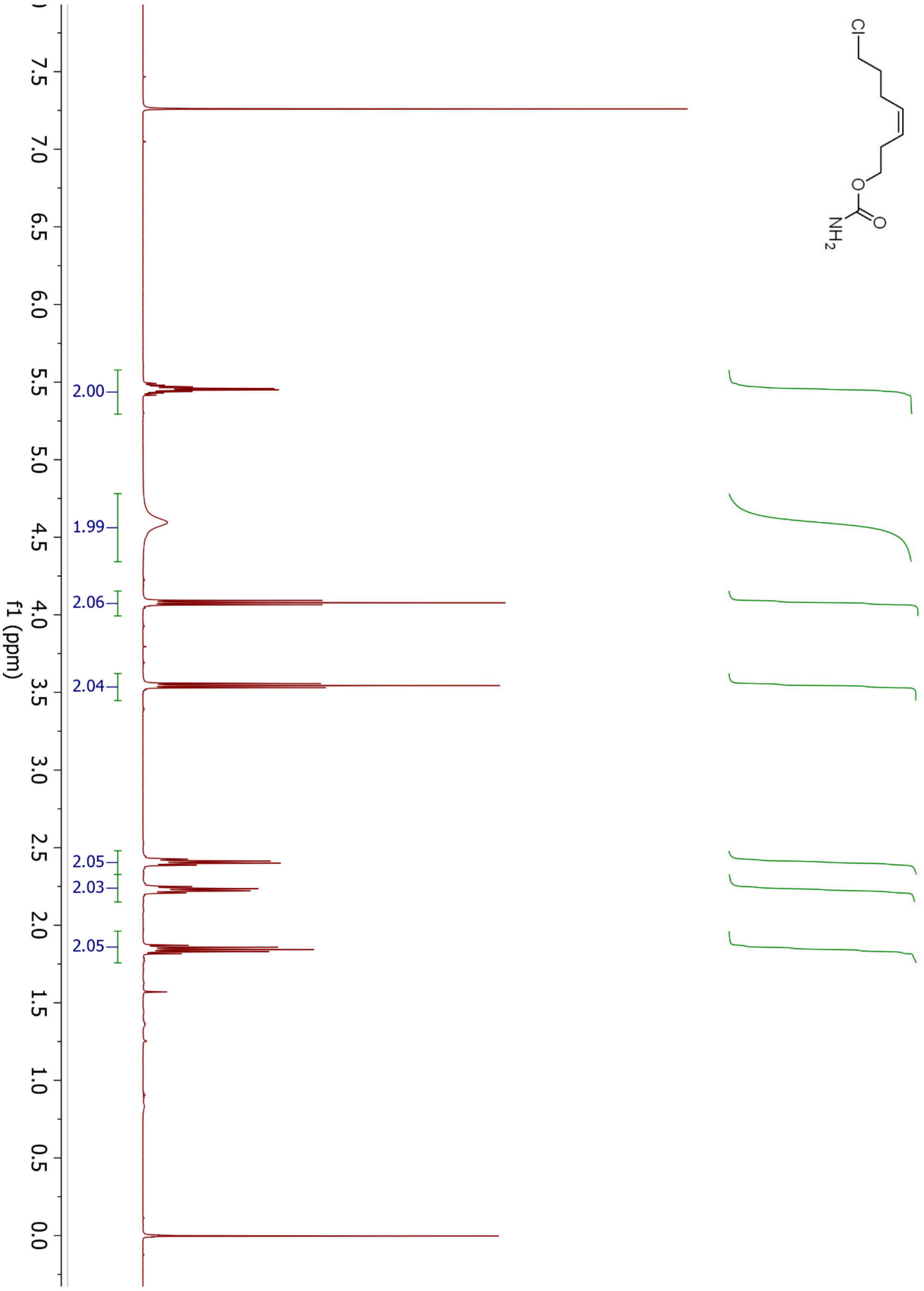

Supplementary Figure 17. ^{13}C NMR (126 MHz, $CDCl_3$) for precursor to compound **2f**

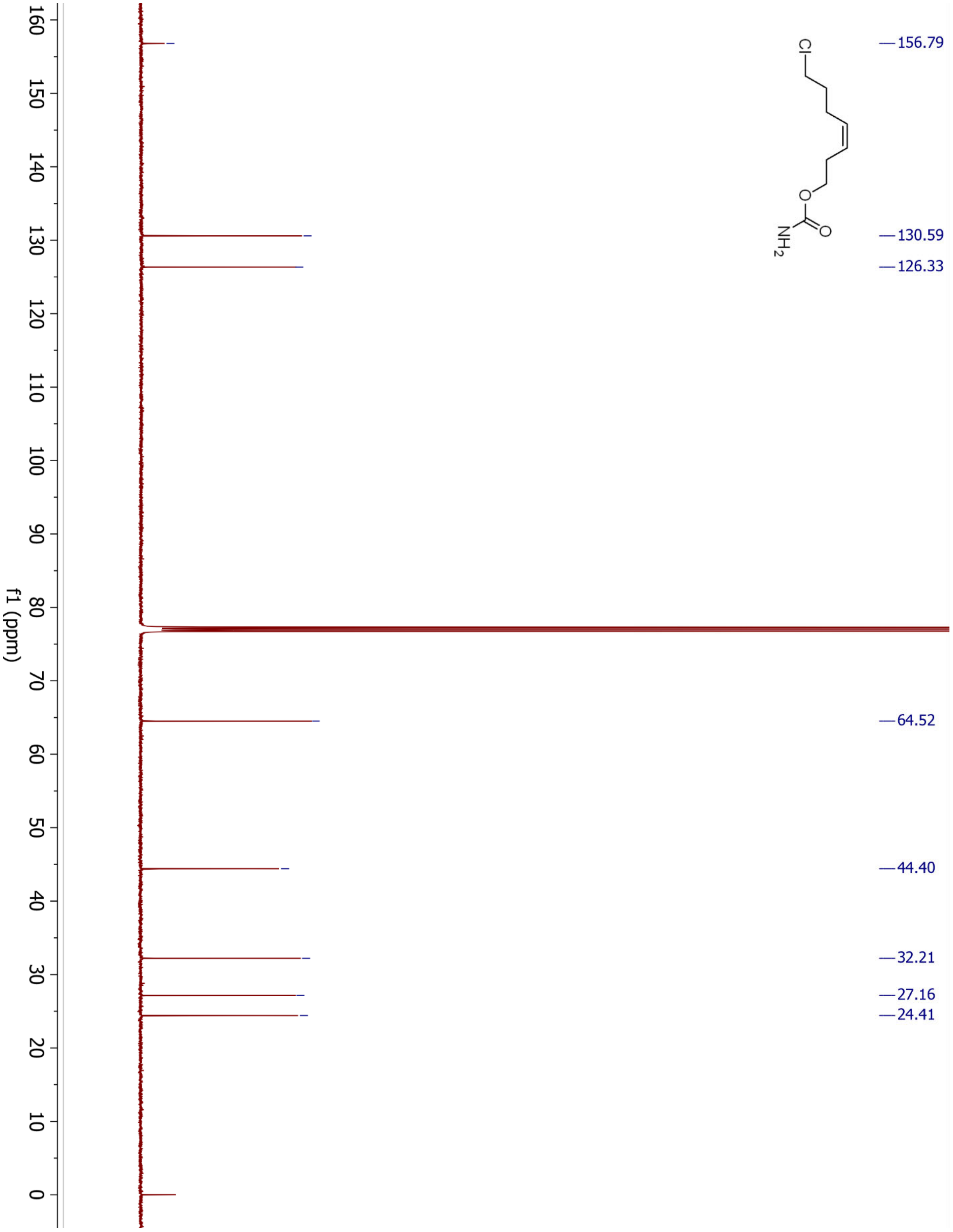

Supplementary Figure 18. ^{1}H NMR (500 MHz, $CDCl_3$) for precursor to compound **2h**

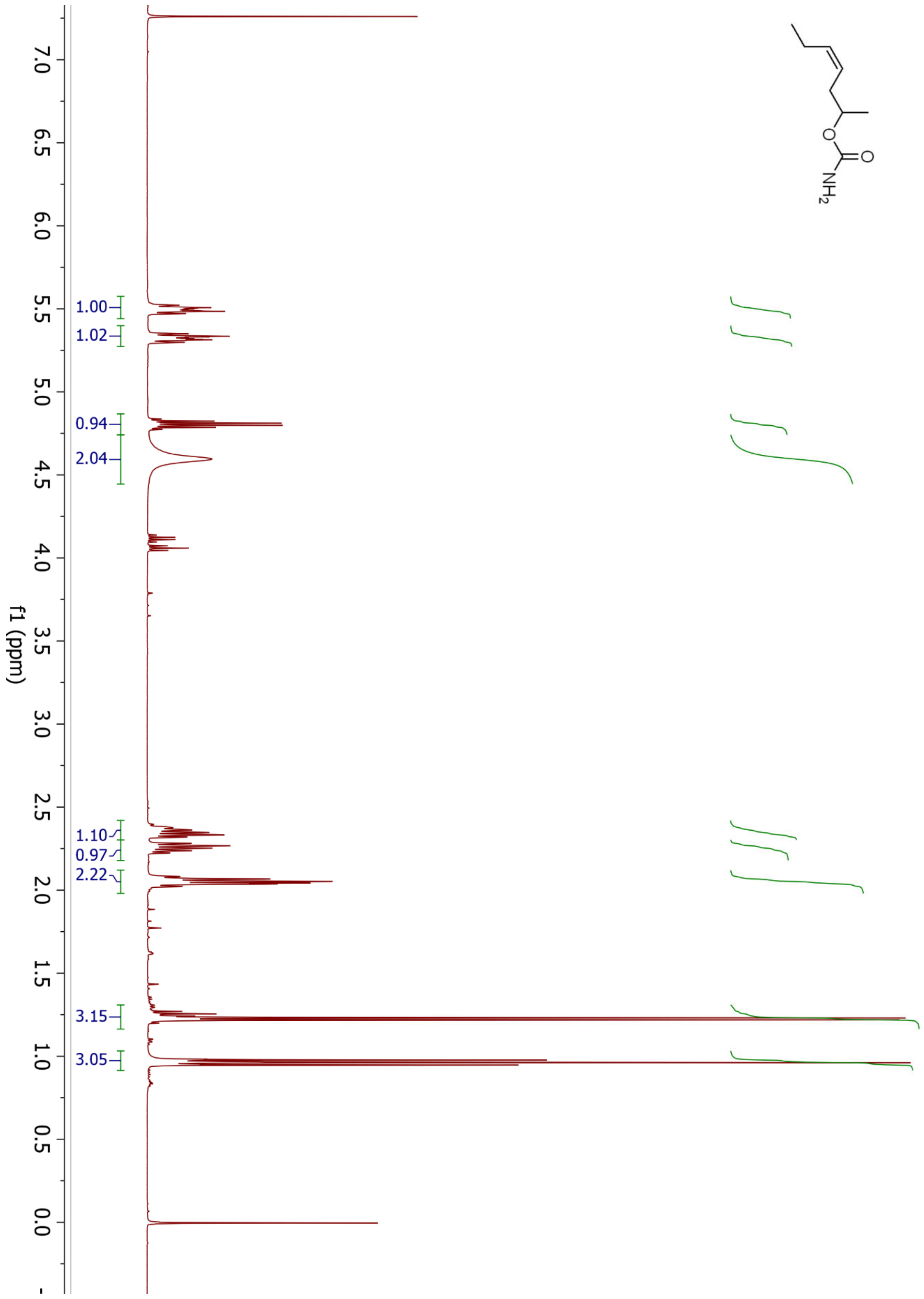

Supplementary Figure 19. ^{13}C NMR (126 MHz, $CDCl_3$) for precursor compound **2h**

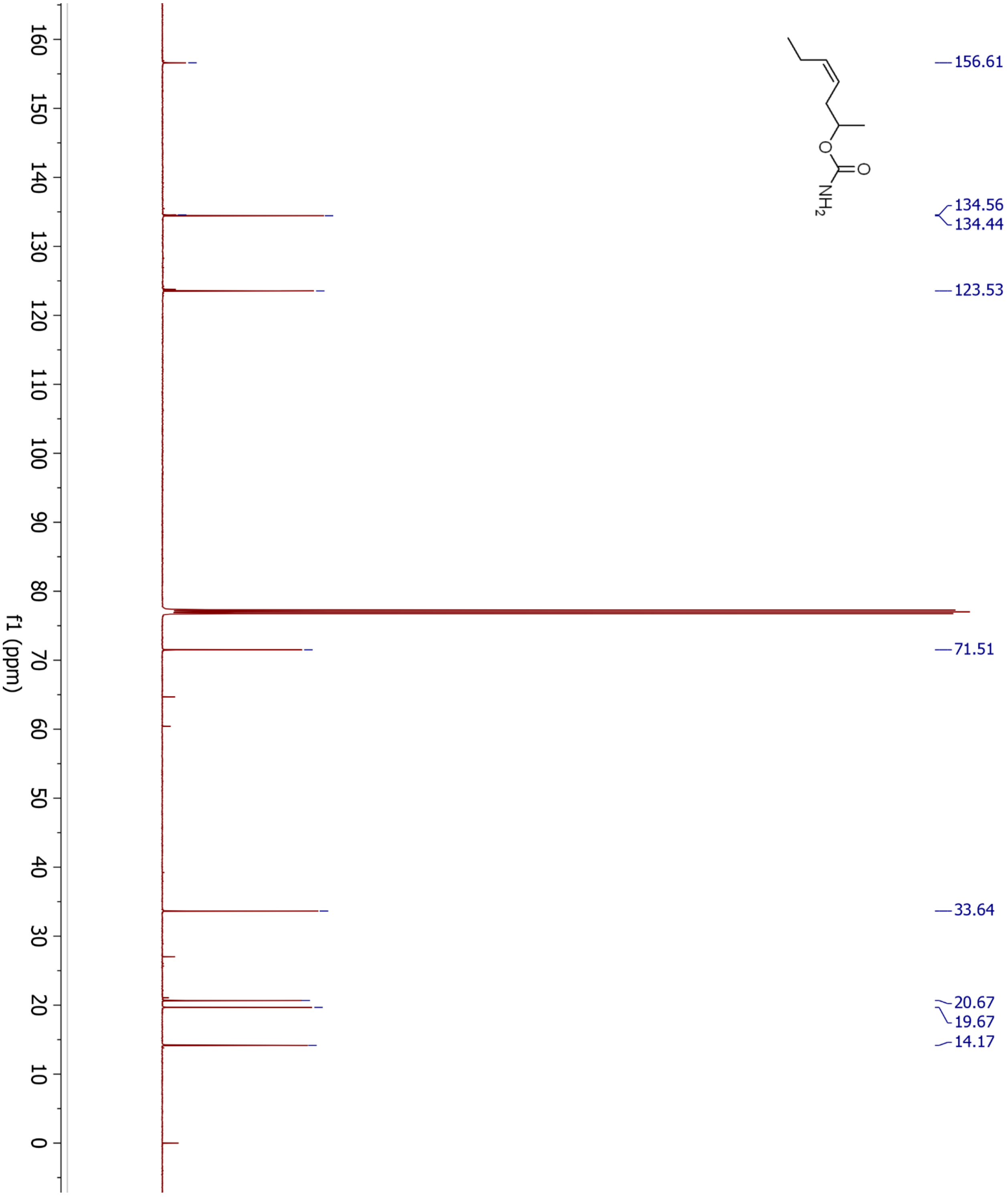

Supplementary Figure 20. ^{1}H NMR (500 MHz, $CDCl_3$) for compound **2a**

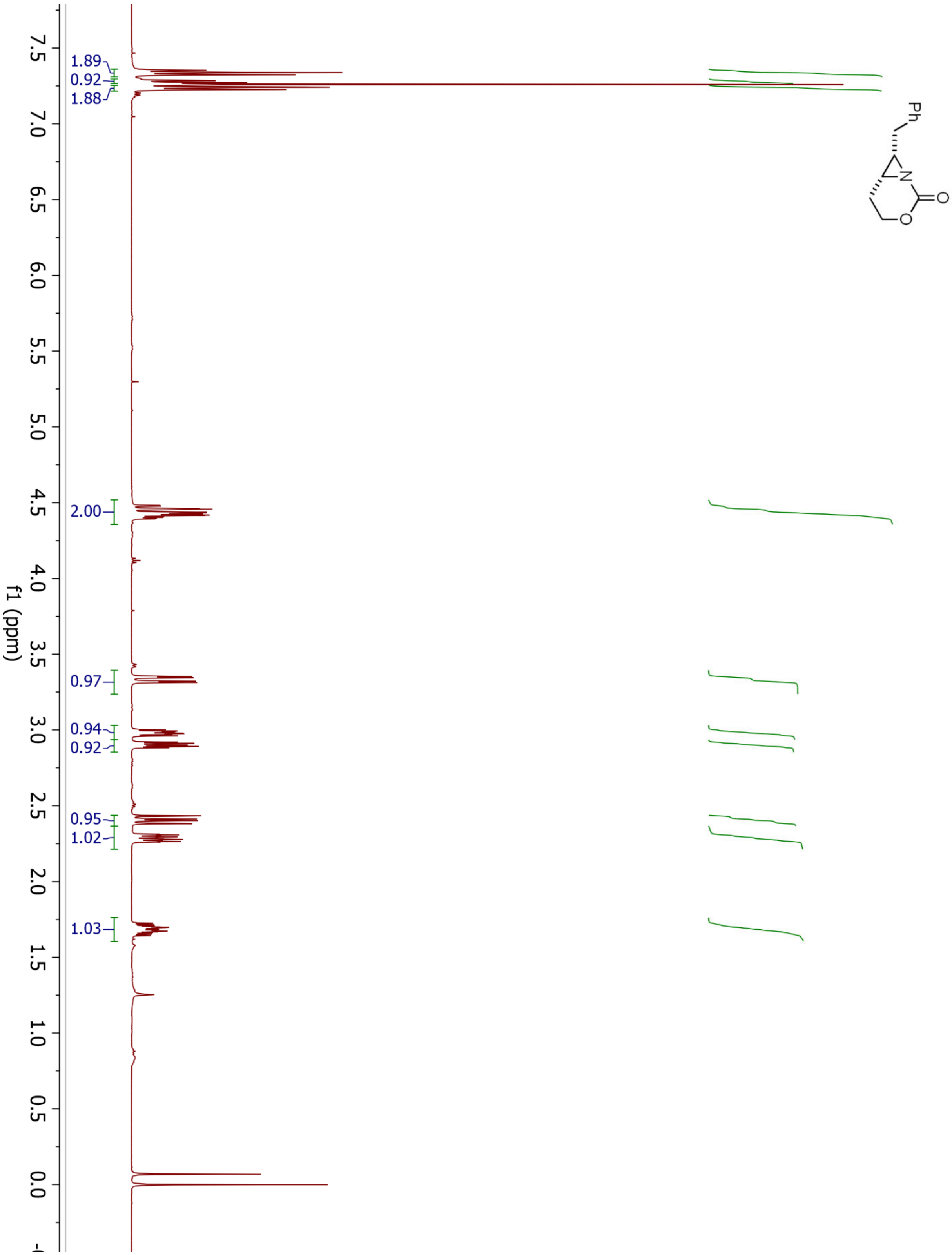

Supplementary Figure 21. ^{13}C NMR (126 MHz, $CDCl_3$) for compound **2a**

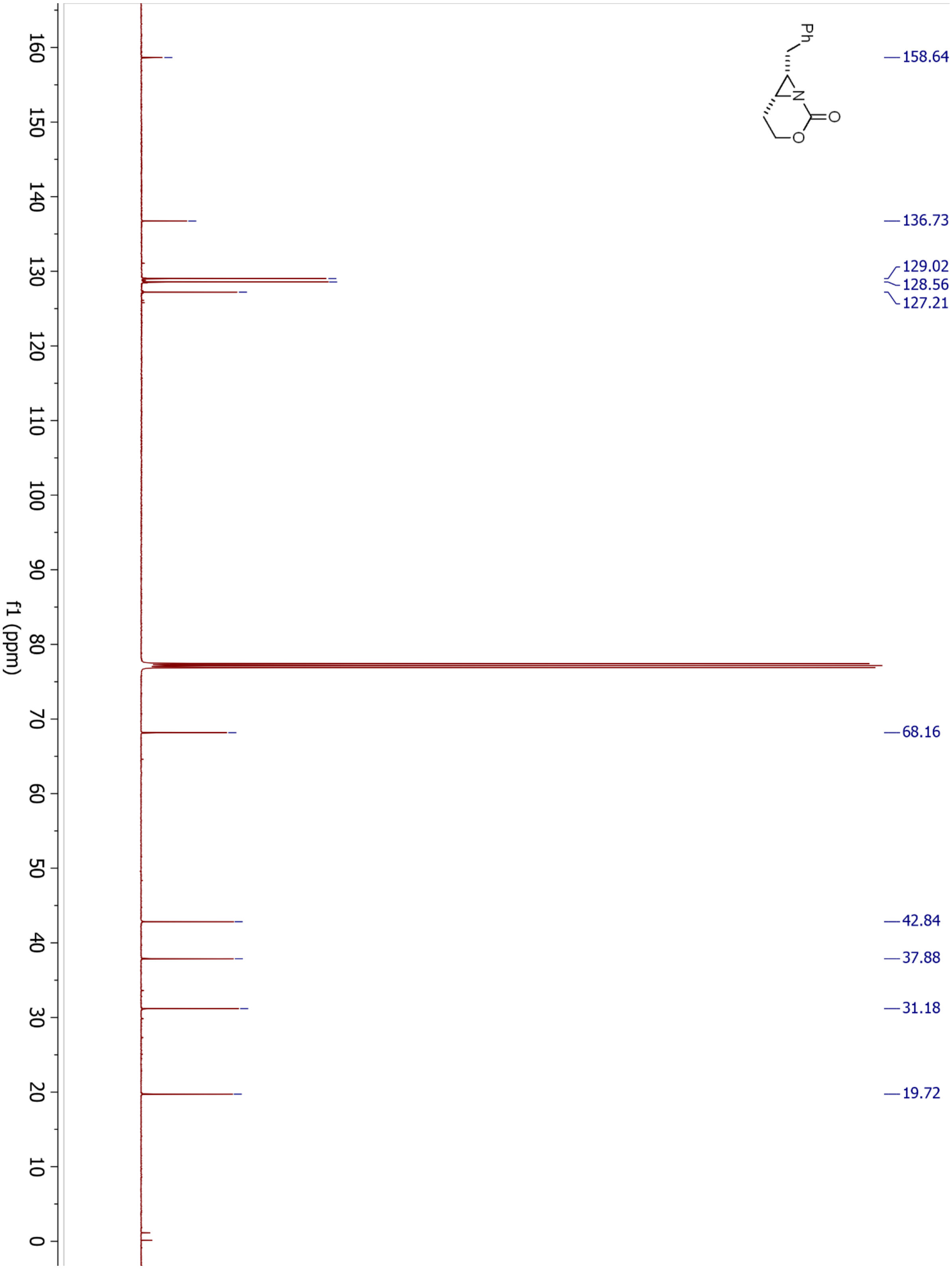

Supplementary Figure 22. ^{1}H NMR (500 MHz, $CDCl_3$) for compound **2b**

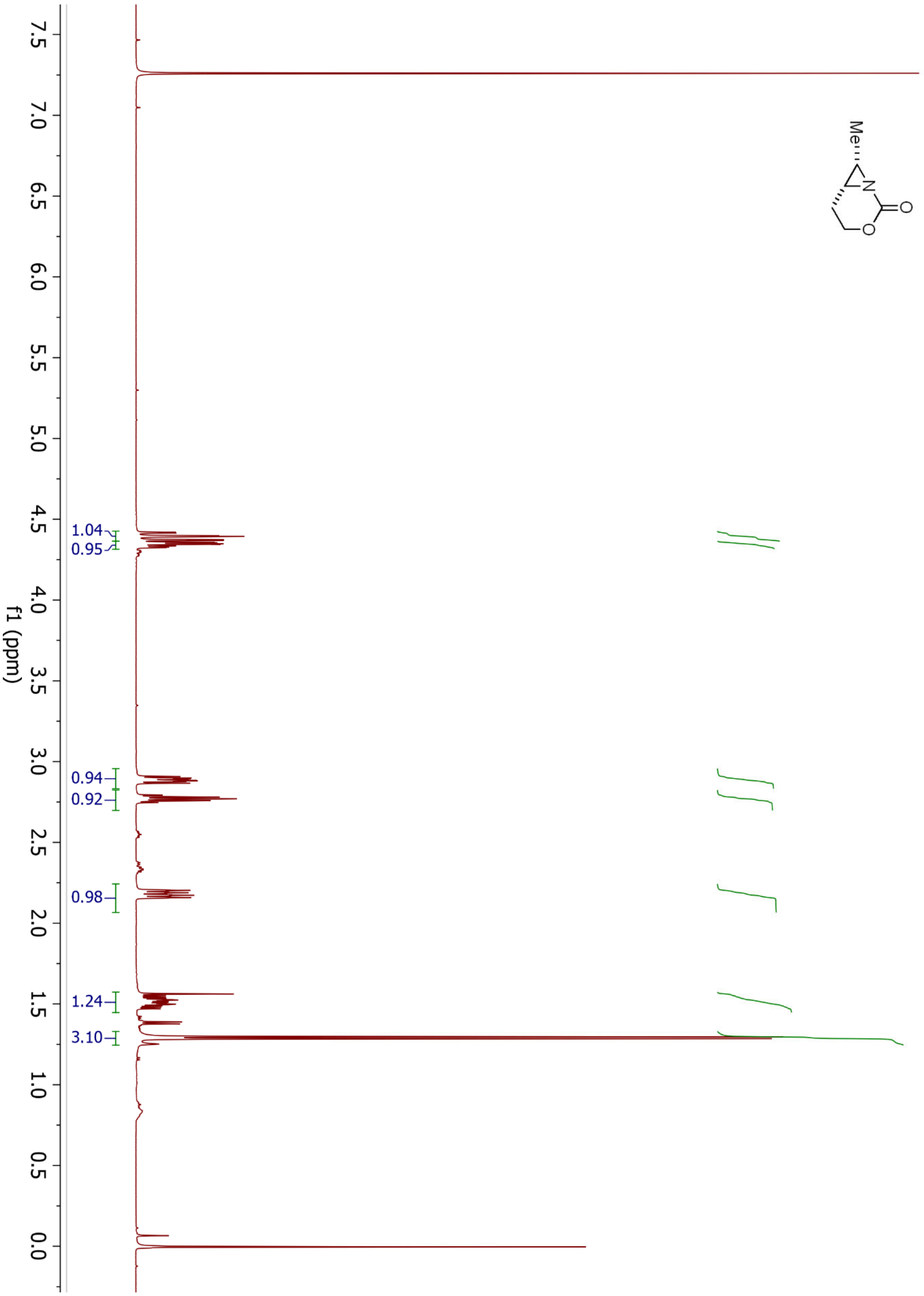

Supplementary Figure 23. ^{13}C NMR (126 MHz, $CDCl_3$) for compound **2b**

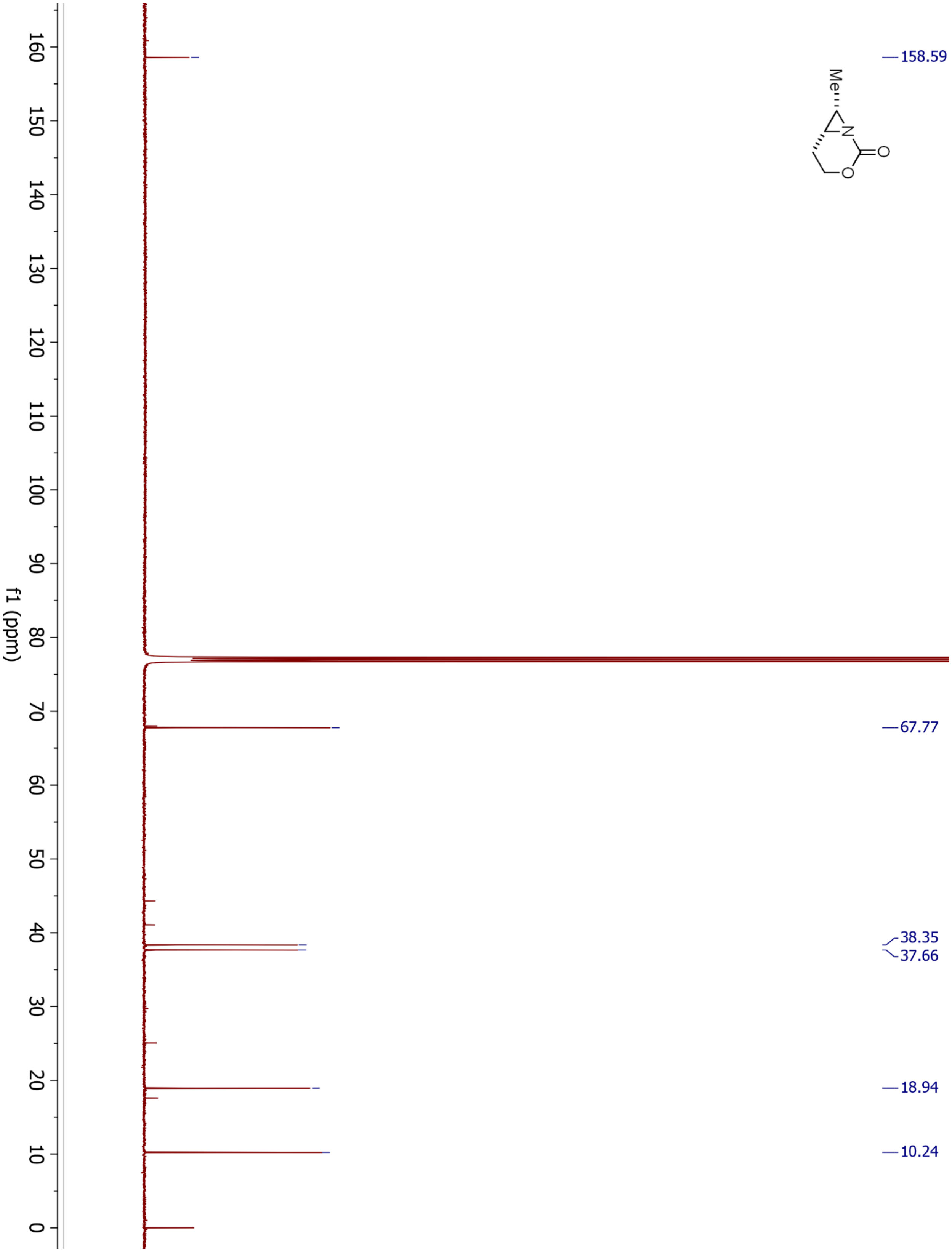

Supplementary Figure 24. ^{1}H NMR (500 MHz, $CDCl_3$) for compound **2d**

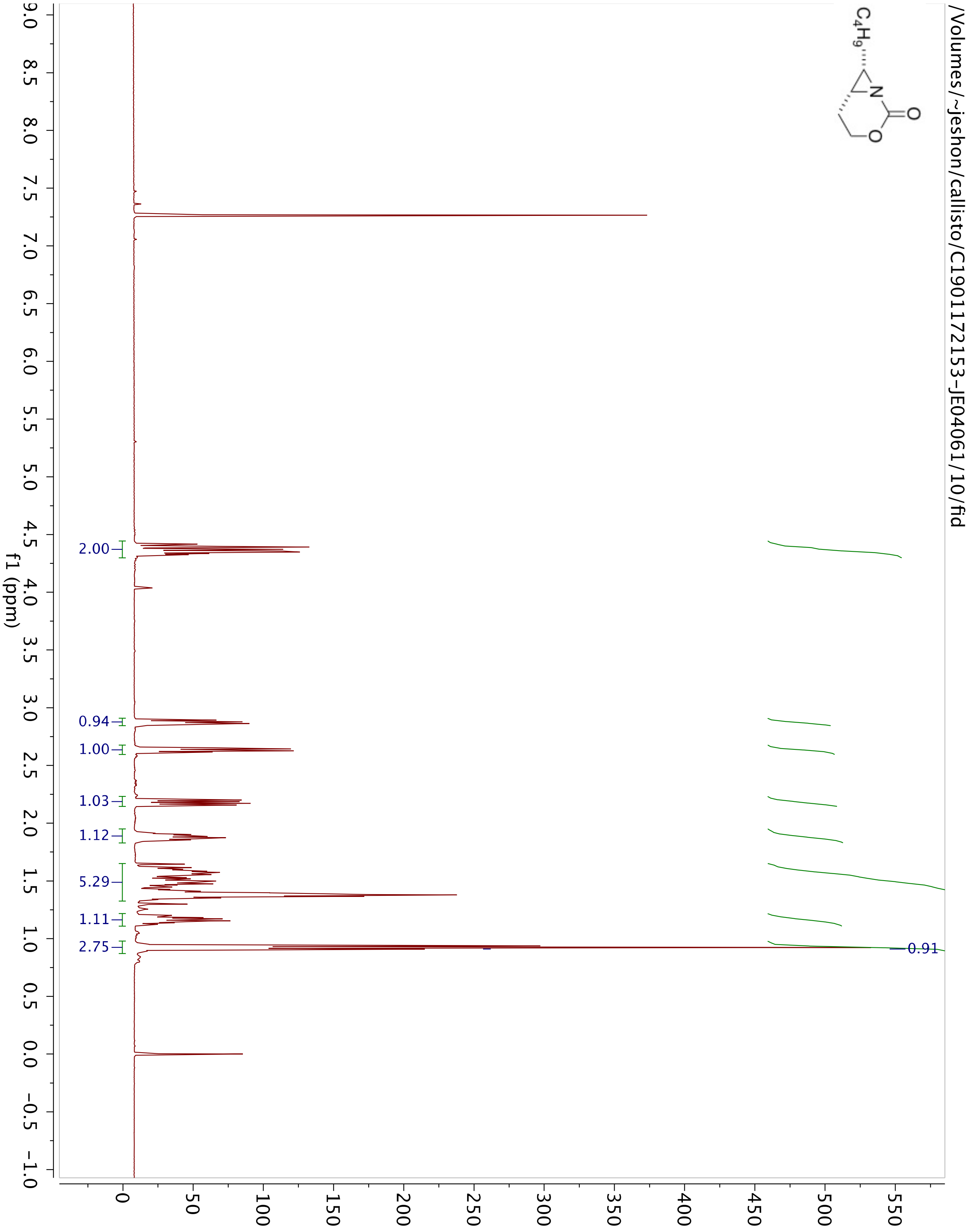

Supplementary Figure 25. ^{13}C NMR (126 MHz, $CDCl_3$) for compound **2d**

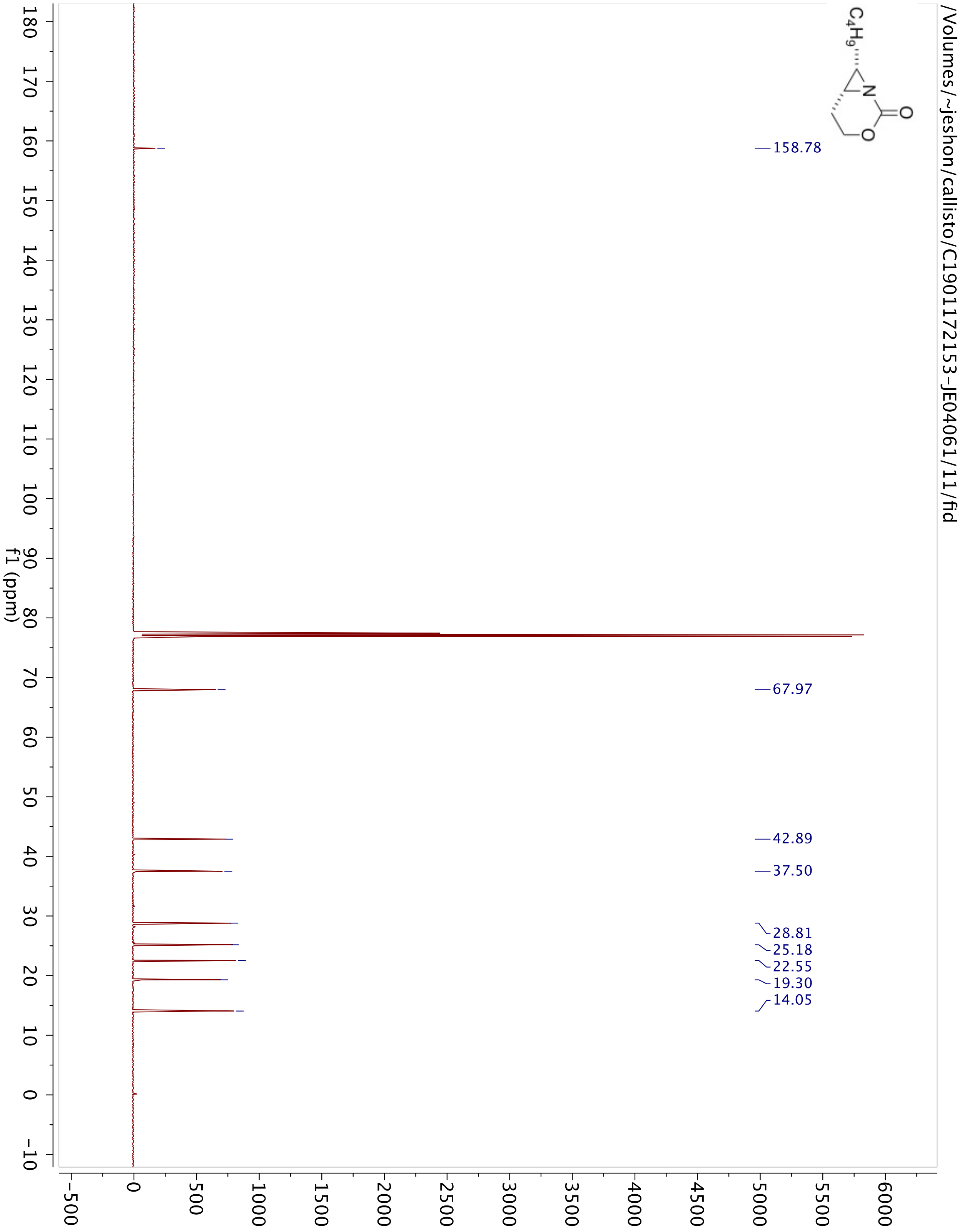

Supplementary Figure 26. ^{1}H NMR (500 MHz, $CDCl_3$) for compound **2e**

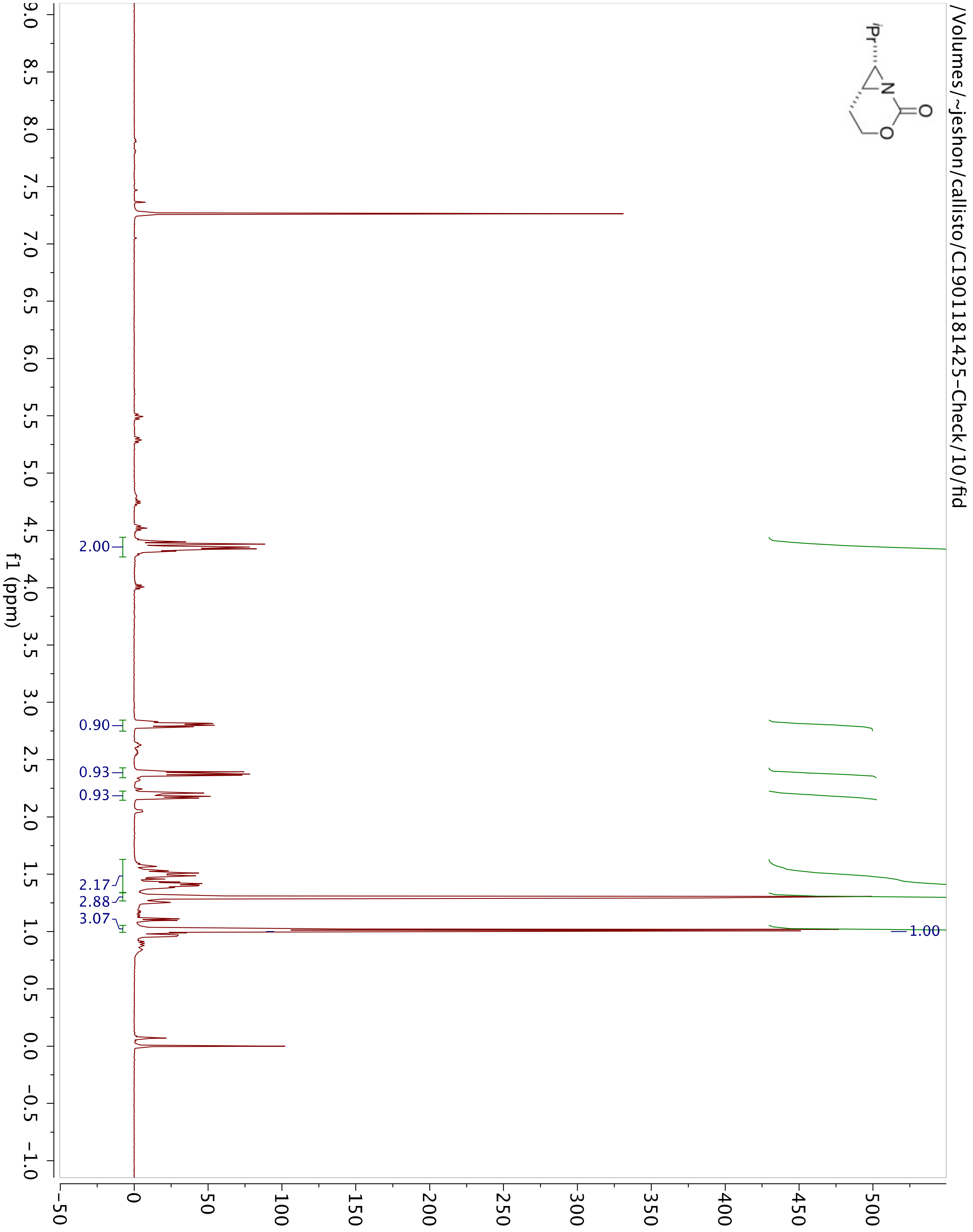

Supplementary Figure 27. ^{13}C NMR (126 MHz, $CDCl_3$) for compound **2e**

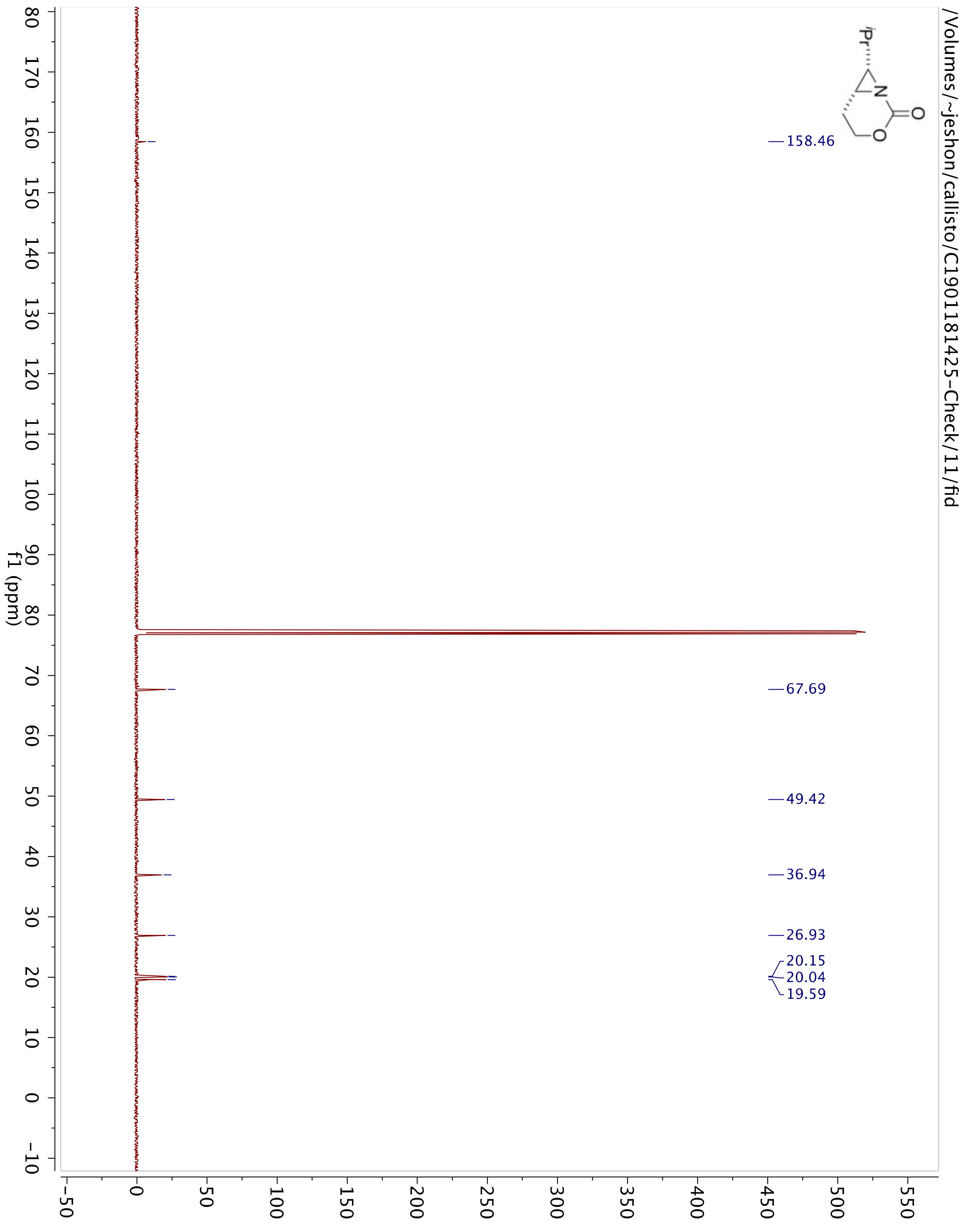

Supplementary Figure 28. ^{1}H NMR (500 MHz, $CDCl_3$) for compound **2f**

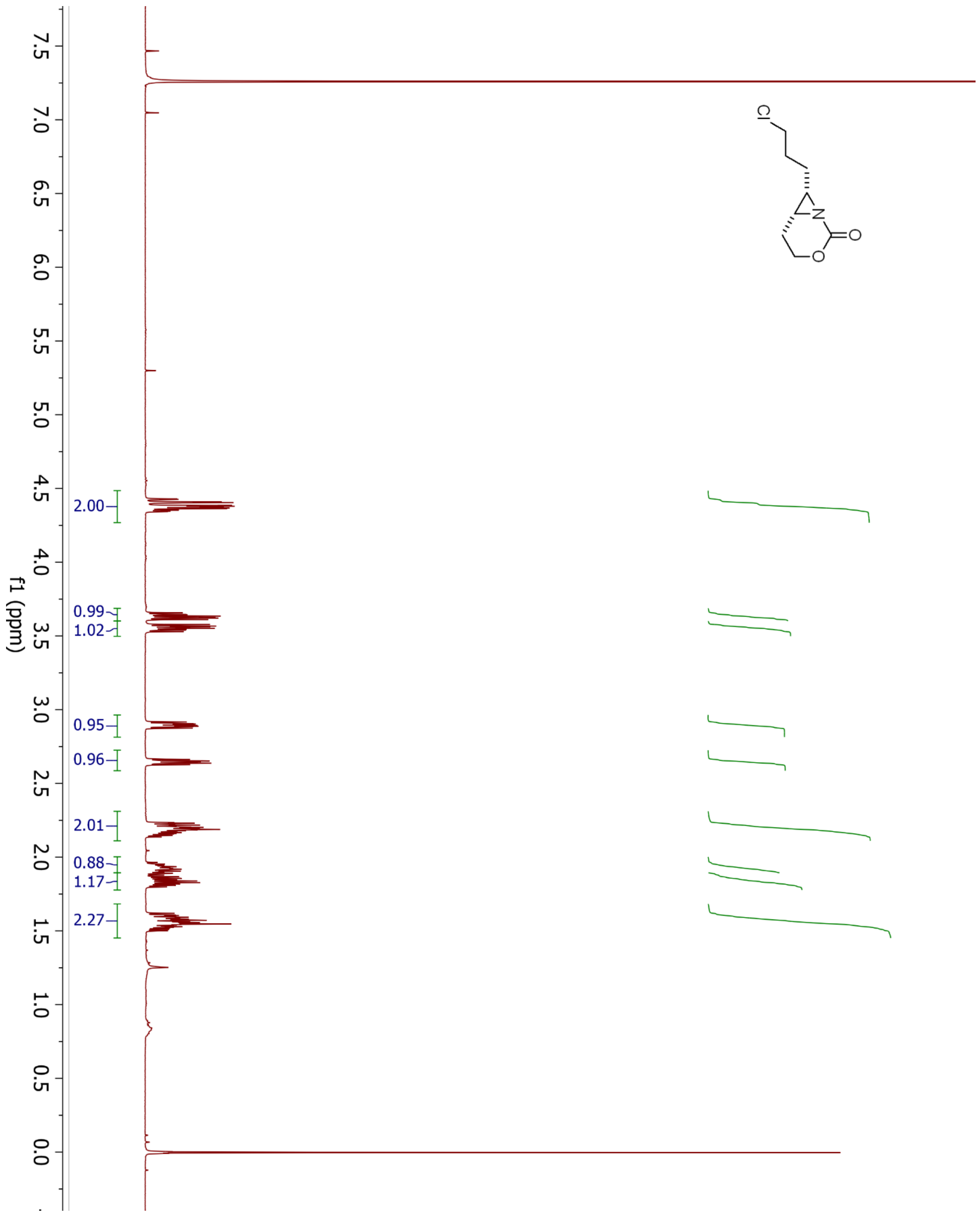

Supplementary Figure 29. ^{13}C NMR (126 MHz, $CDCl_3$) for compound **2f**

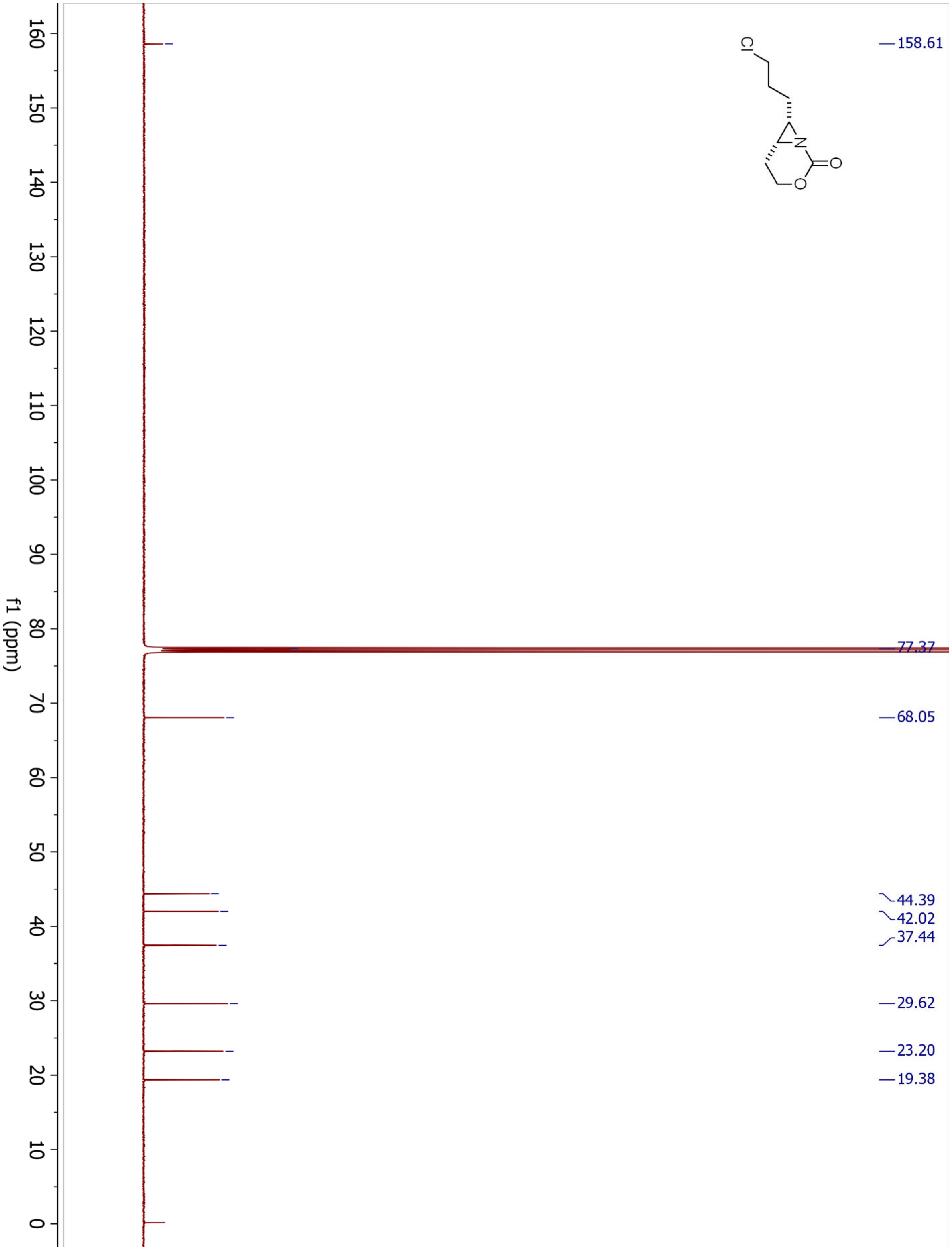

Supplementary Figure 30. ^{1}H NMR (500 MHz, $CDCl_3$) for compound **2g**

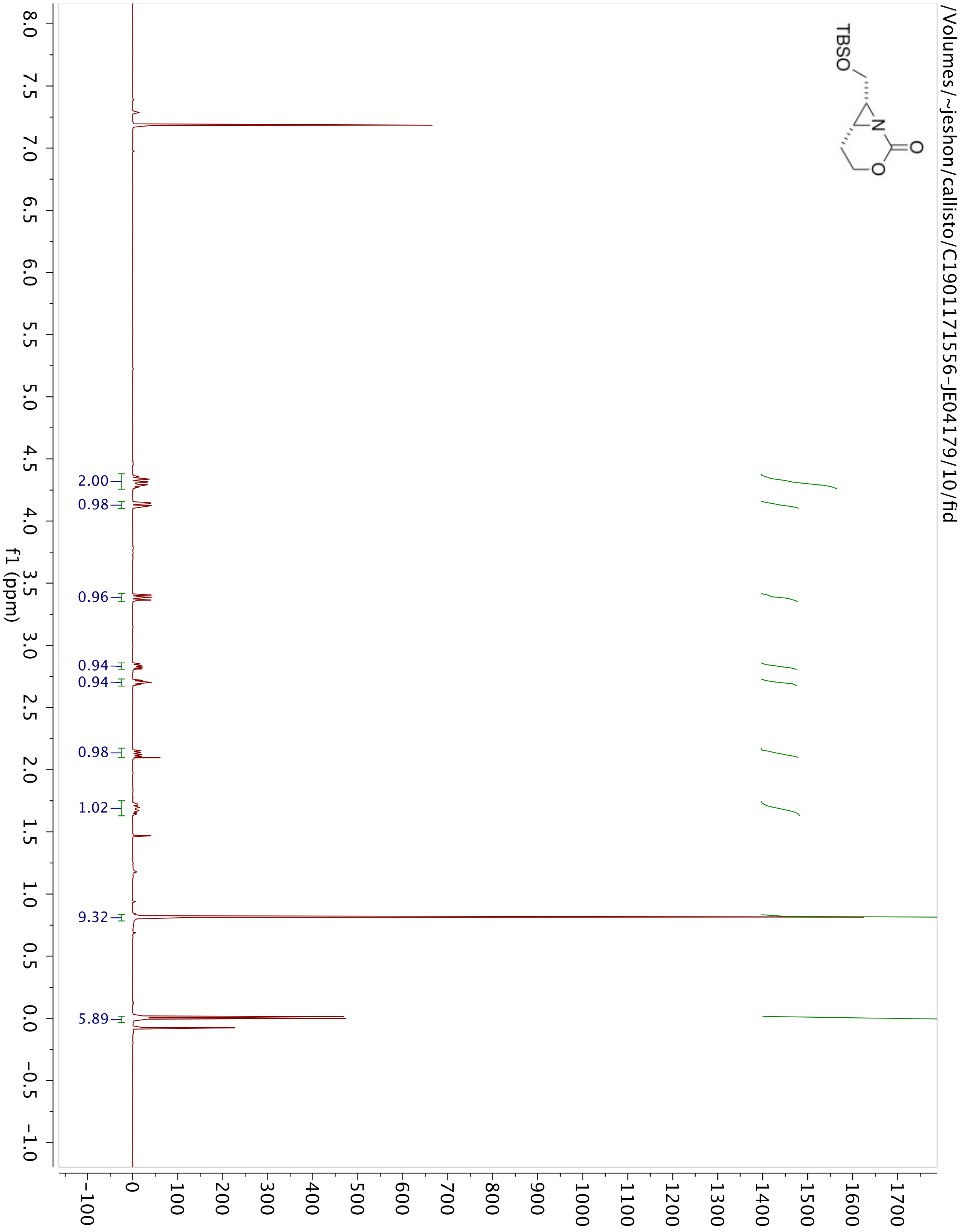

Supplementary Figure 31. ^{13}C NMR (126 MHz, $CDCl_3$) for compound **2g**

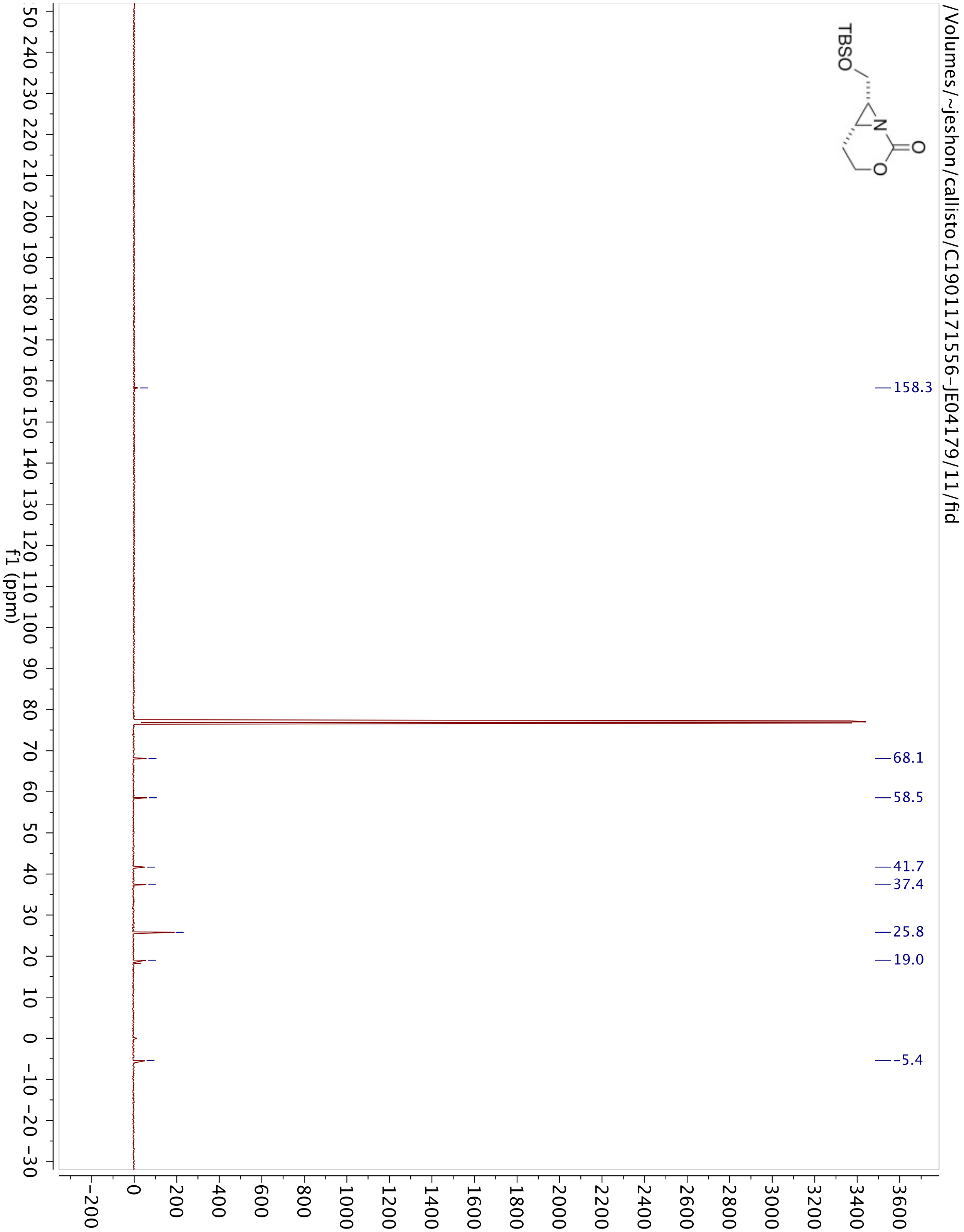

Supplementary Figure 32. ^{1}H NMR (500 MHz, $CDCl_3$) for compound **2h**

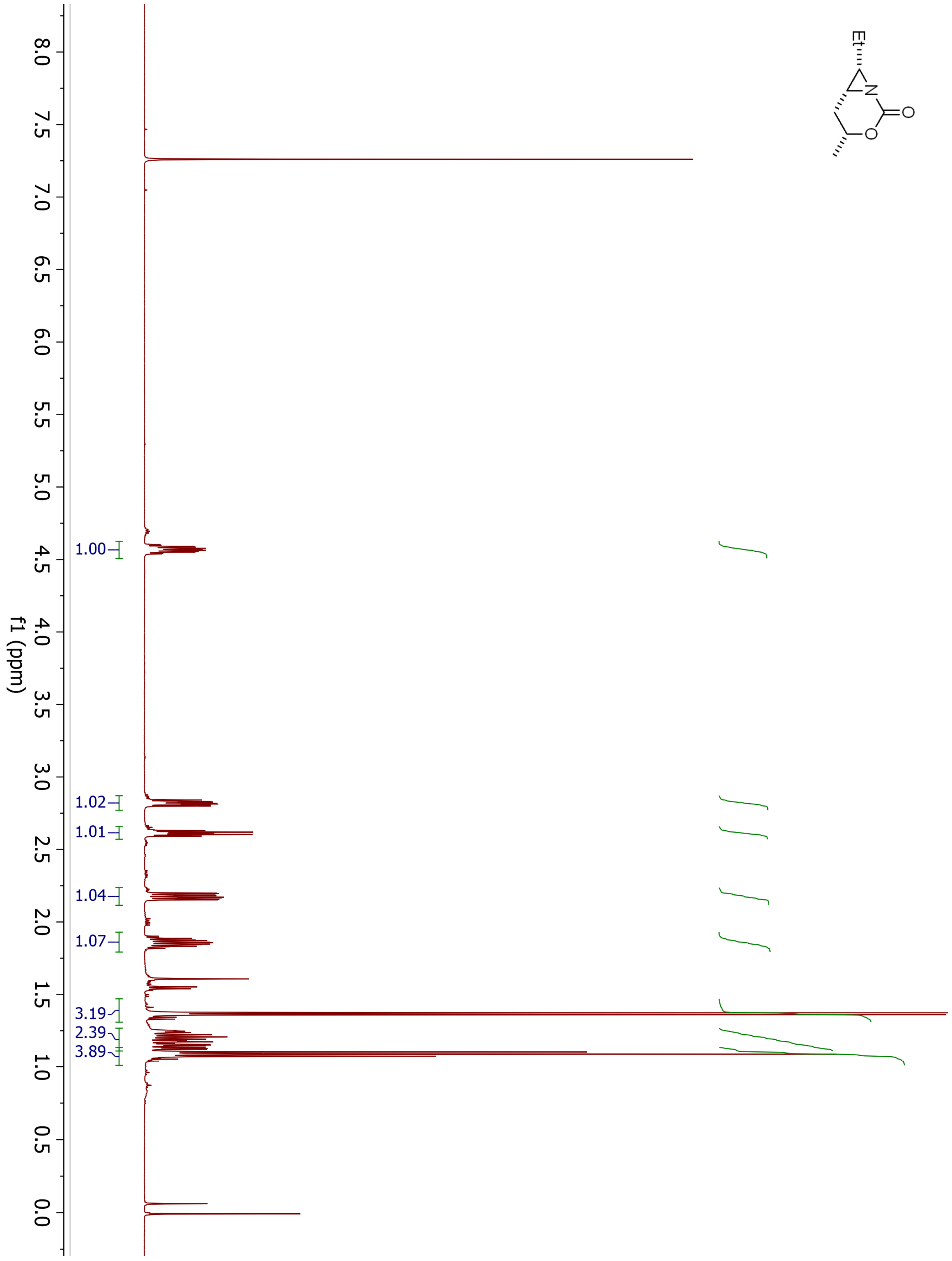

Supplementary Figure 33. ^{13}C NMR (126 MHz, $CDCl_3$) for compound **2h**

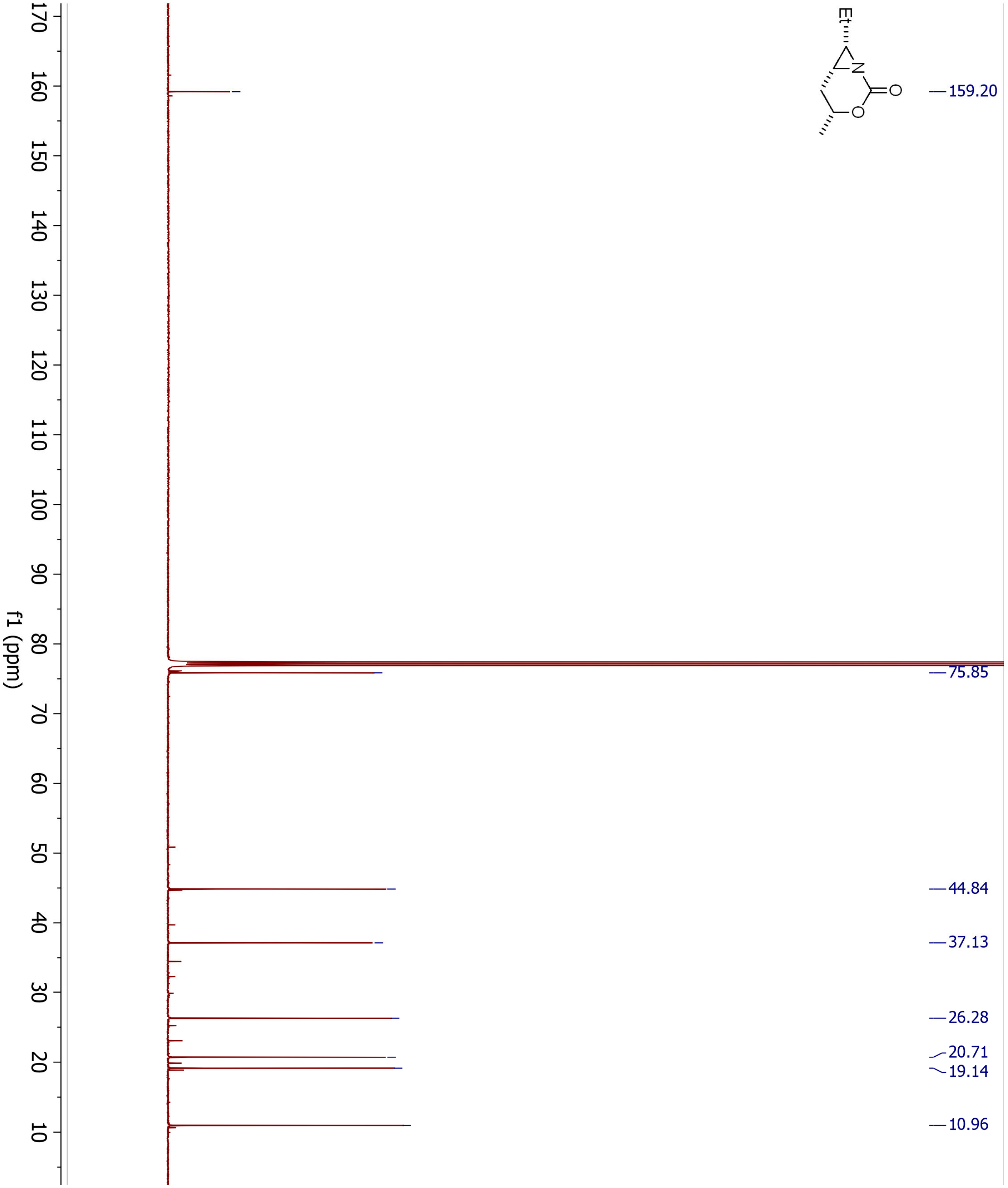

Supplementary Figure 34. ^{1}H NMR (500 MHz, $CDCl_3$) for compound **2i**

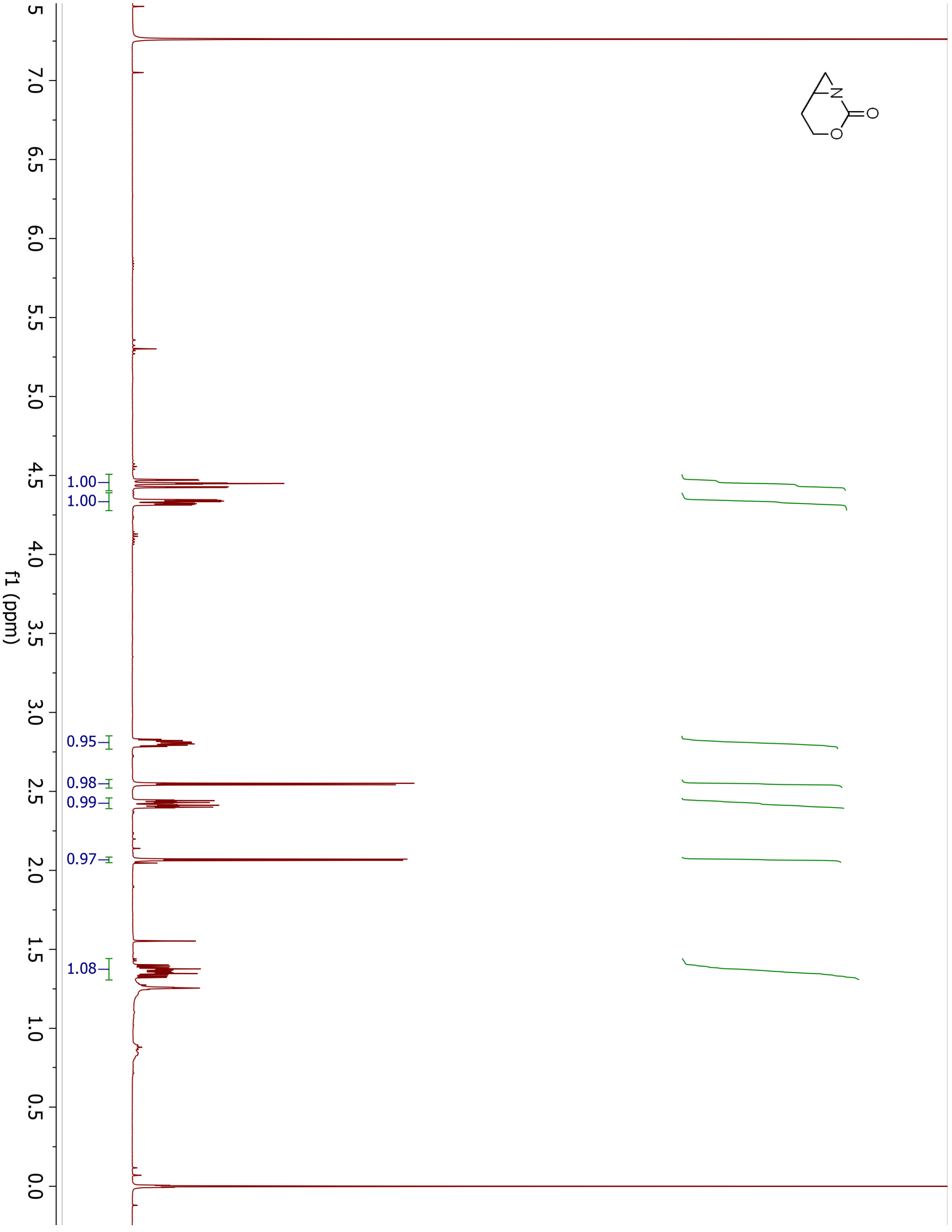

Supplementary Figure 35. ^{13}C NMR (500 MHz, $CDCl_3$) for compound **2i**

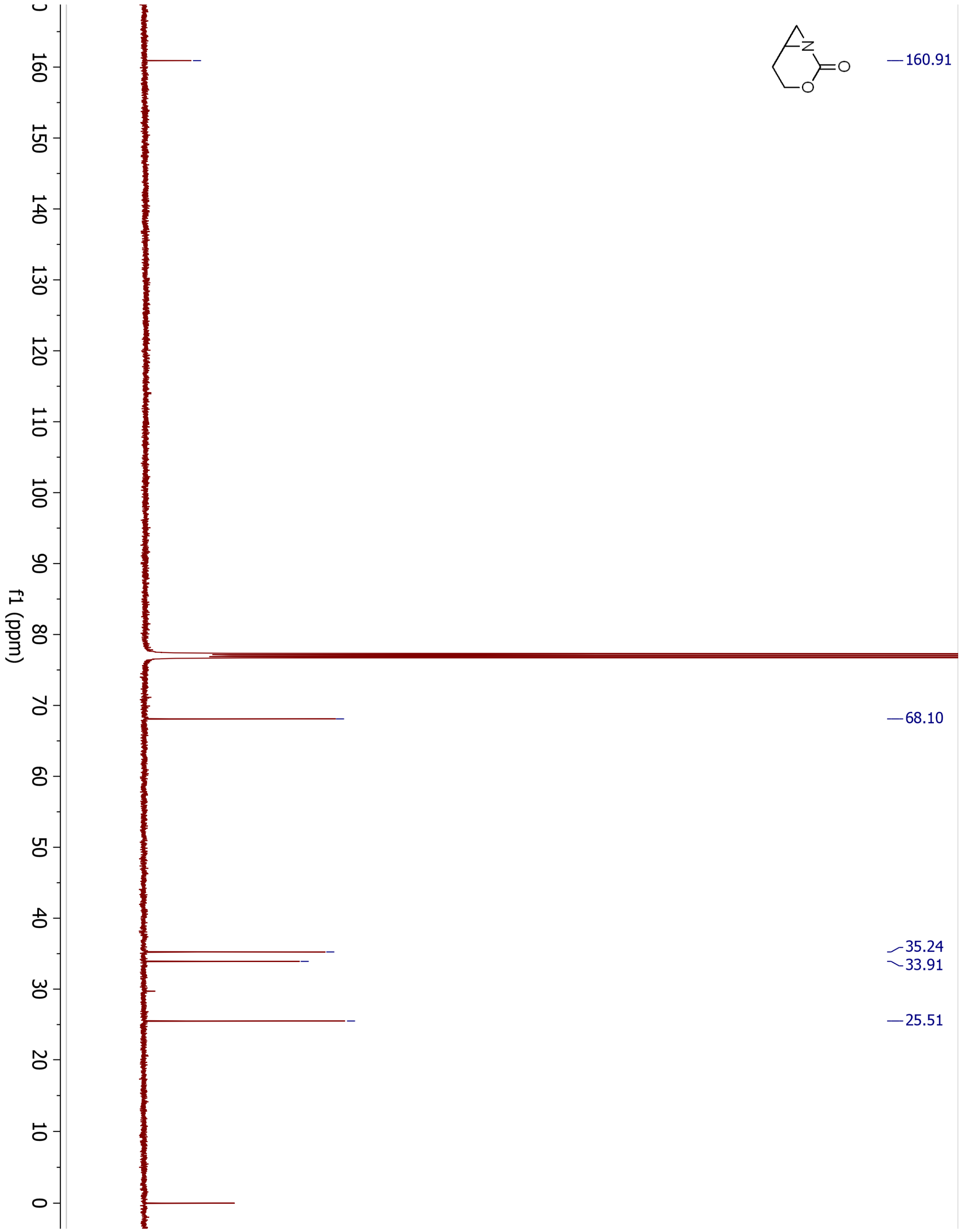

Supplementary Figure 36. ^{1}H NMR (500 MHz, $CDCl_3$) for compound **3c**

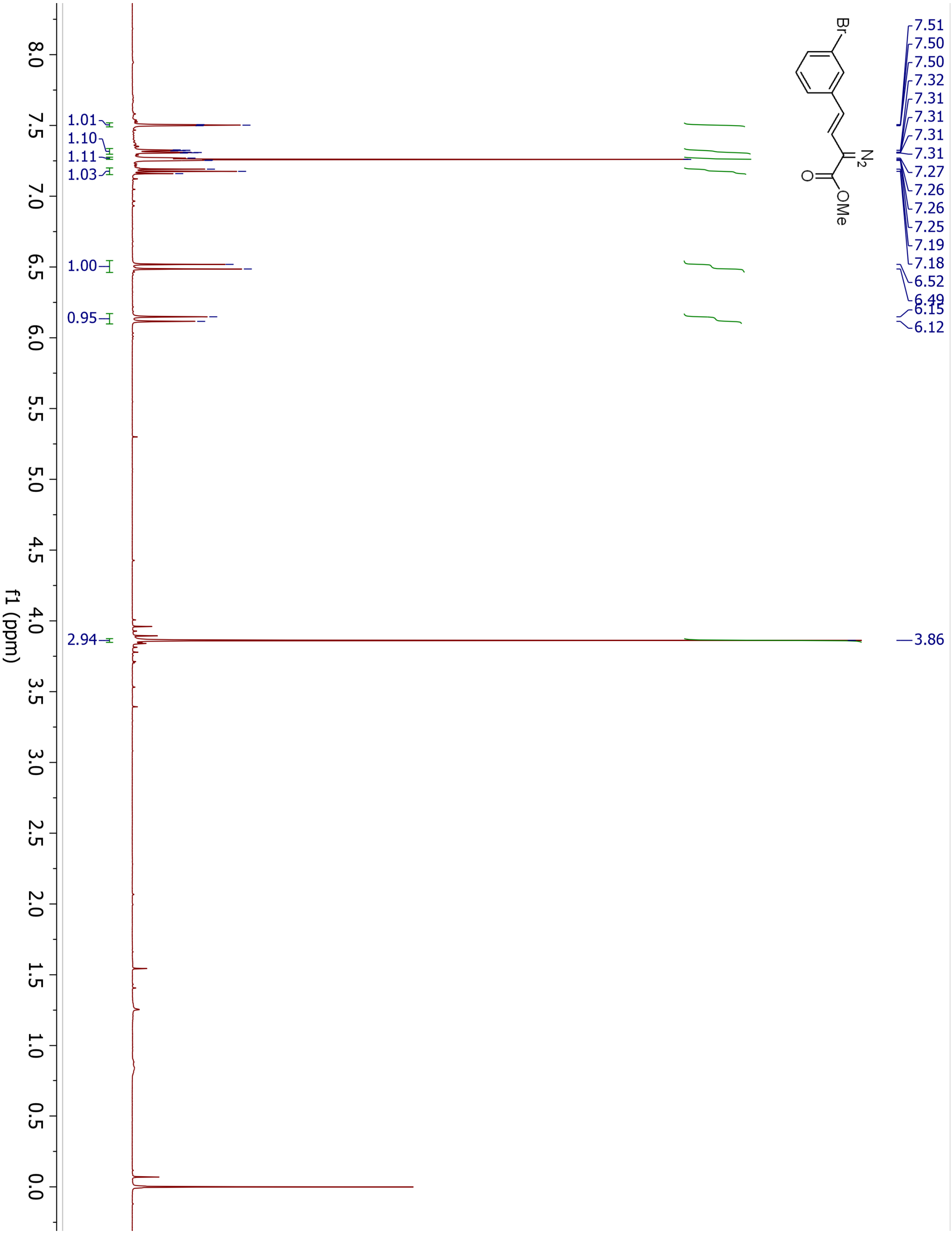

Supplementary Figure 37. ^{13}C NMR (126 MHz, $CDCl_3$) for compound **3c**

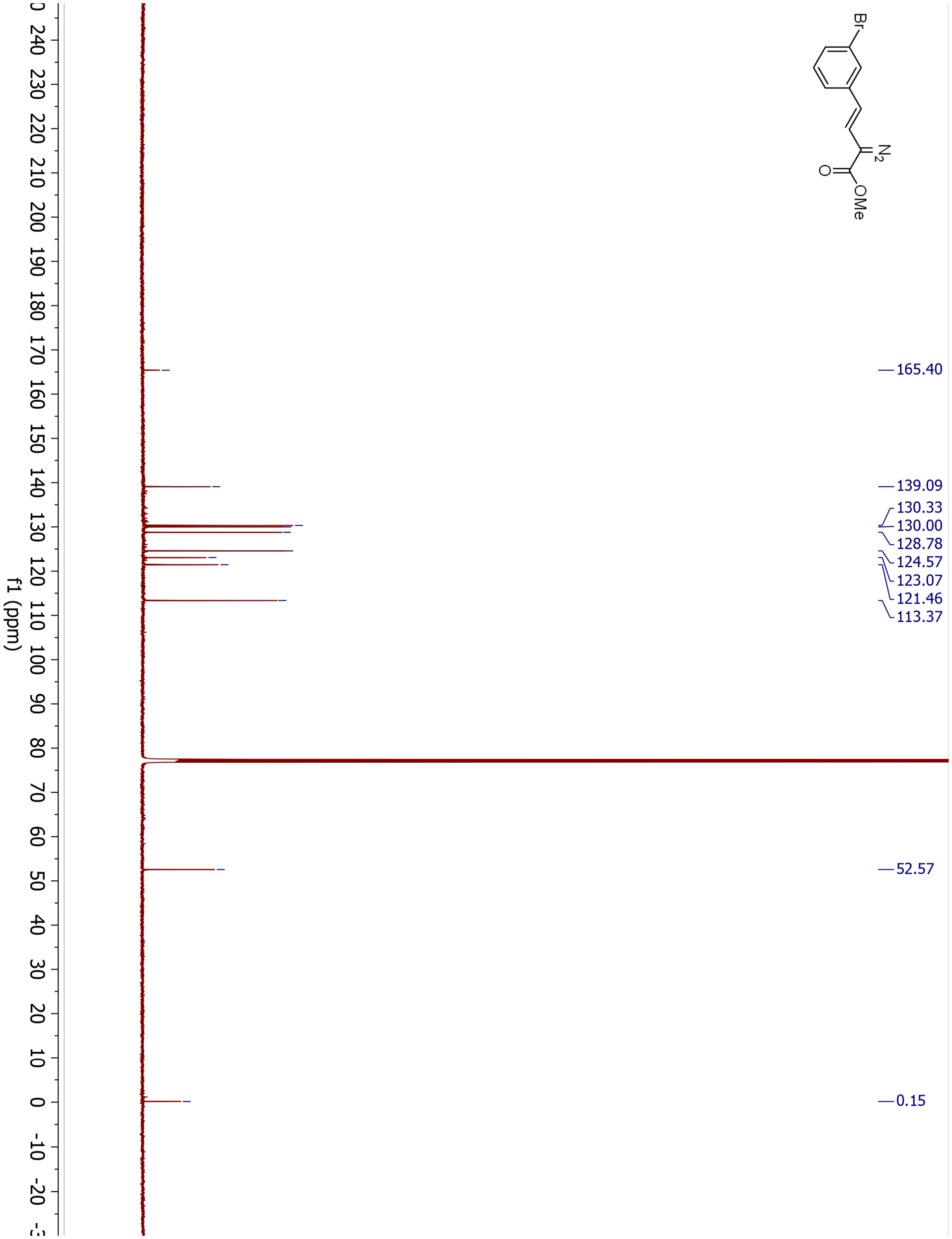

Supplementary Figure 38. ^{1}H NMR (500 MHz, $CDCl_3$) for compound **3g**

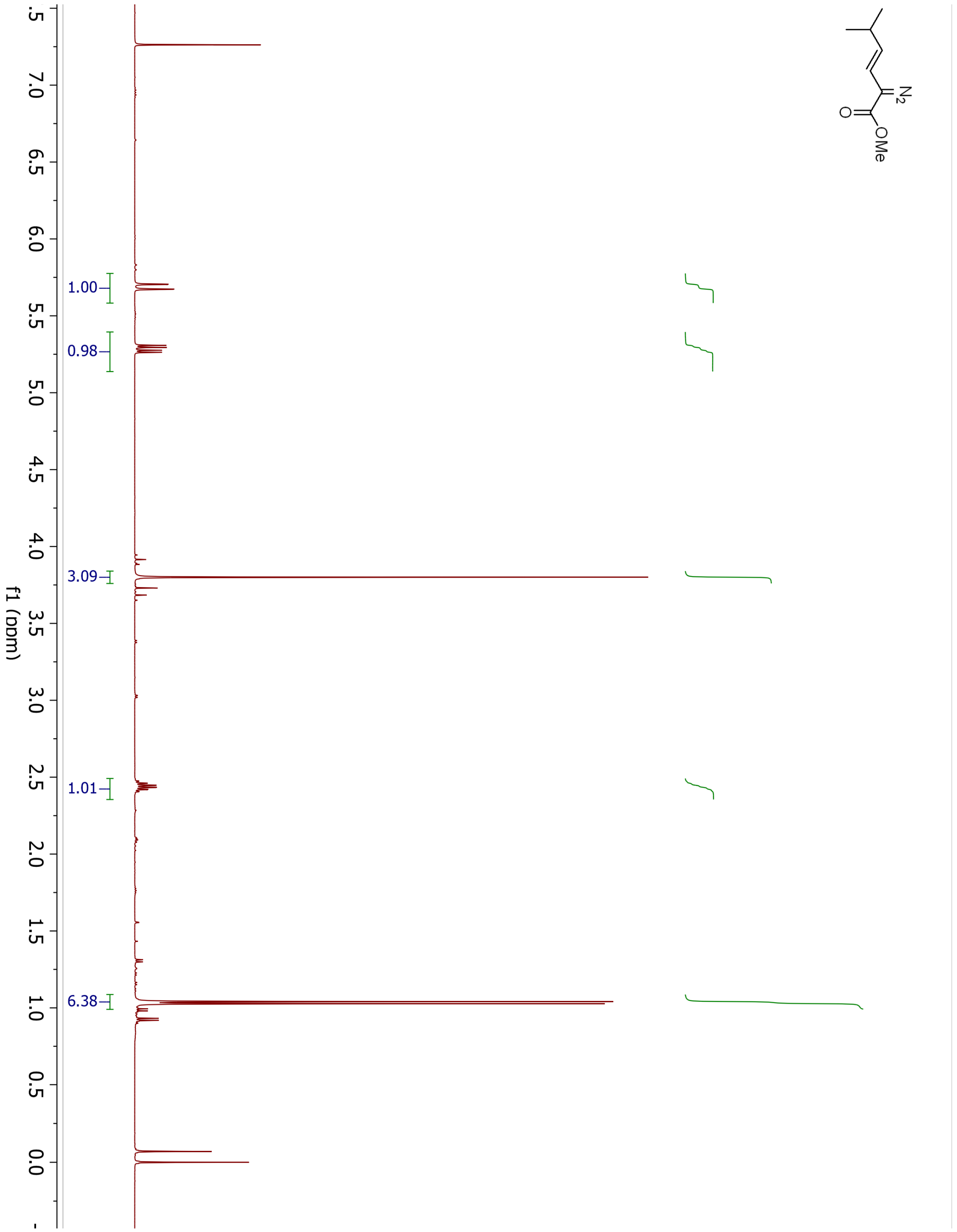

Supplementary Figure 39. ^{13}C NMR (126 MHz, $CDCl_3$) for compound **3g**

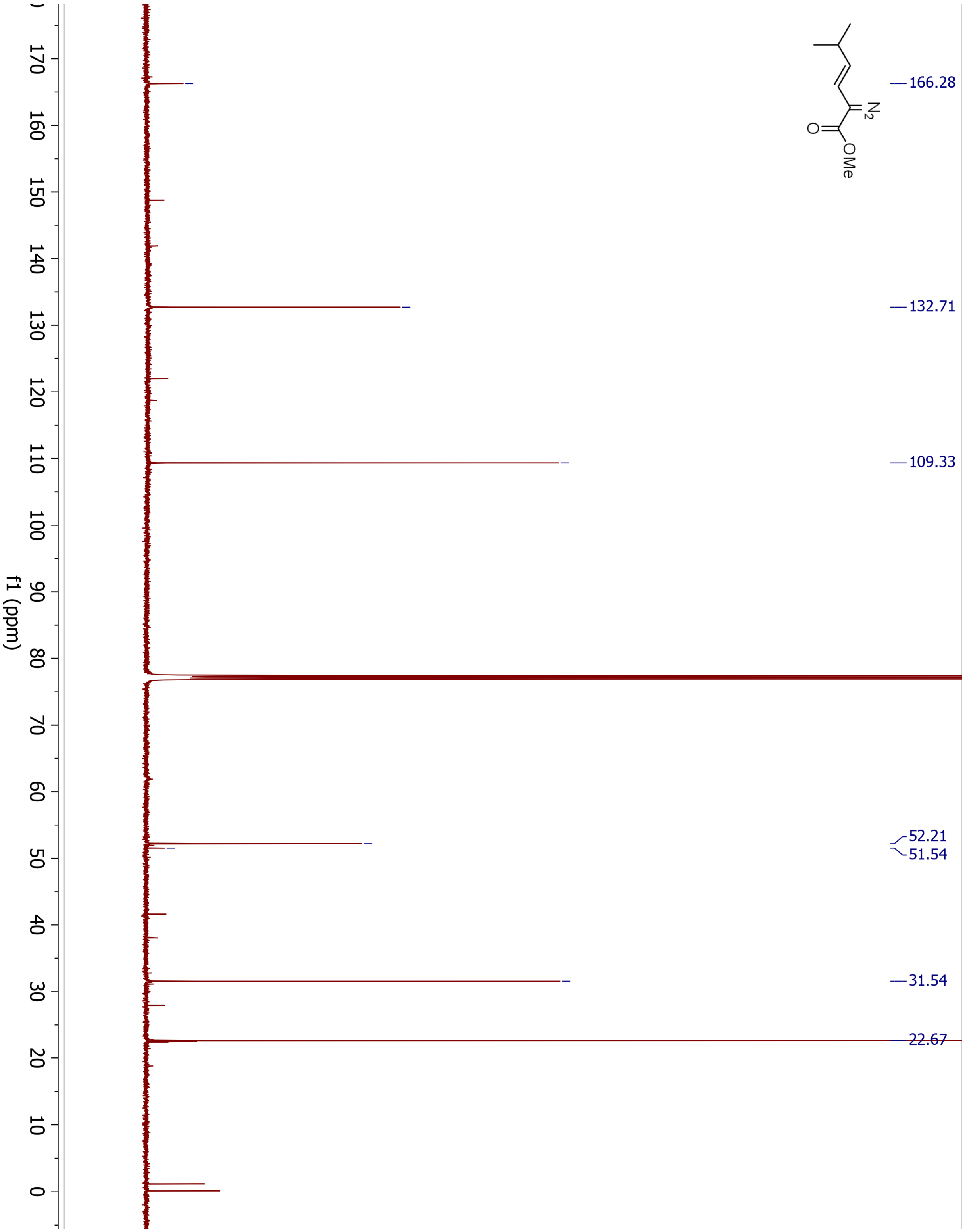

Supplementary Figure 40. ^1H NMR (500 MHz, $CDCl_3$) for compound **4aa**

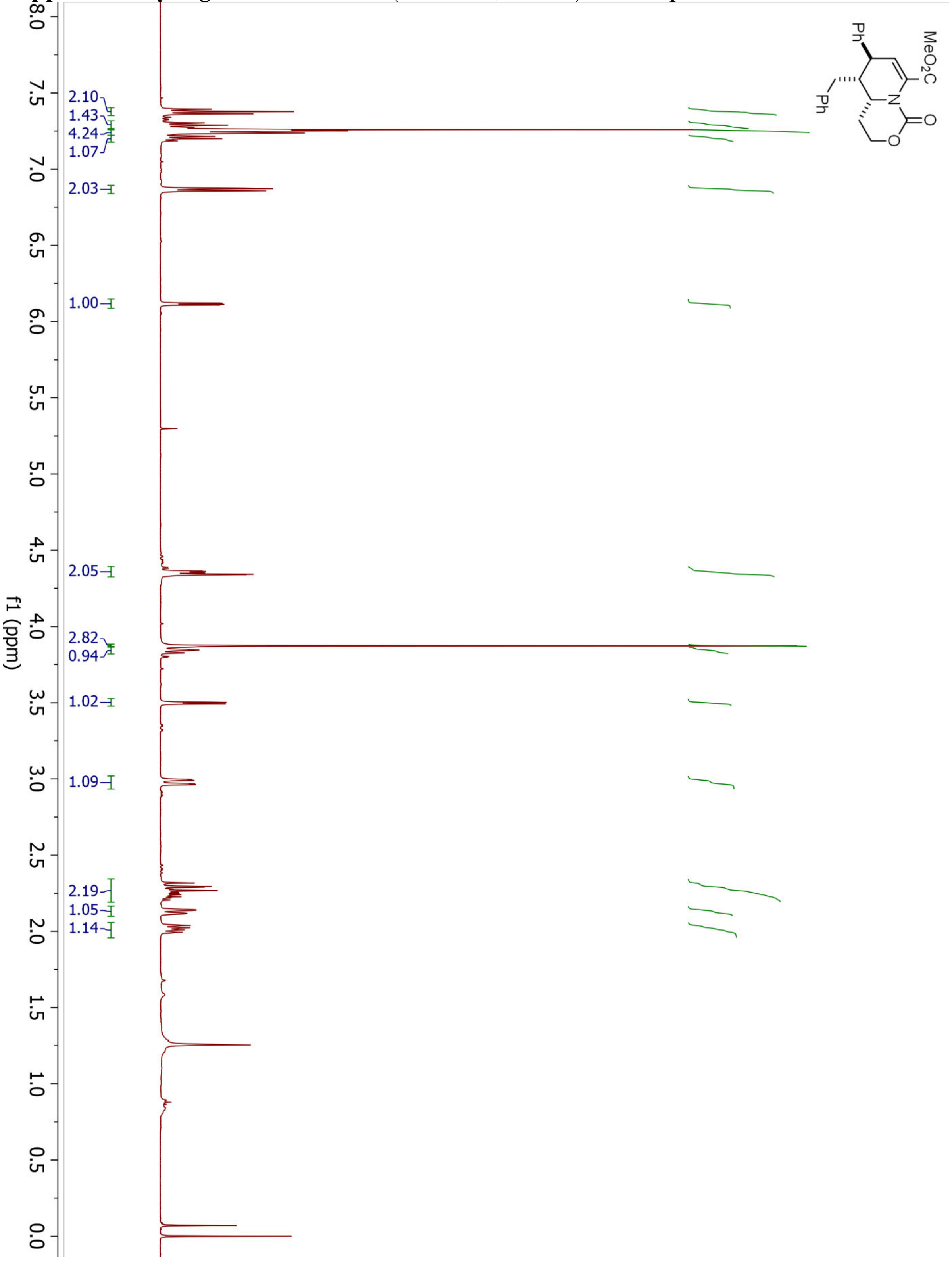

Supplementary Figure 41. ^{13}C NMR (126 MHz, $CDCl_3$) for compound **4aa**

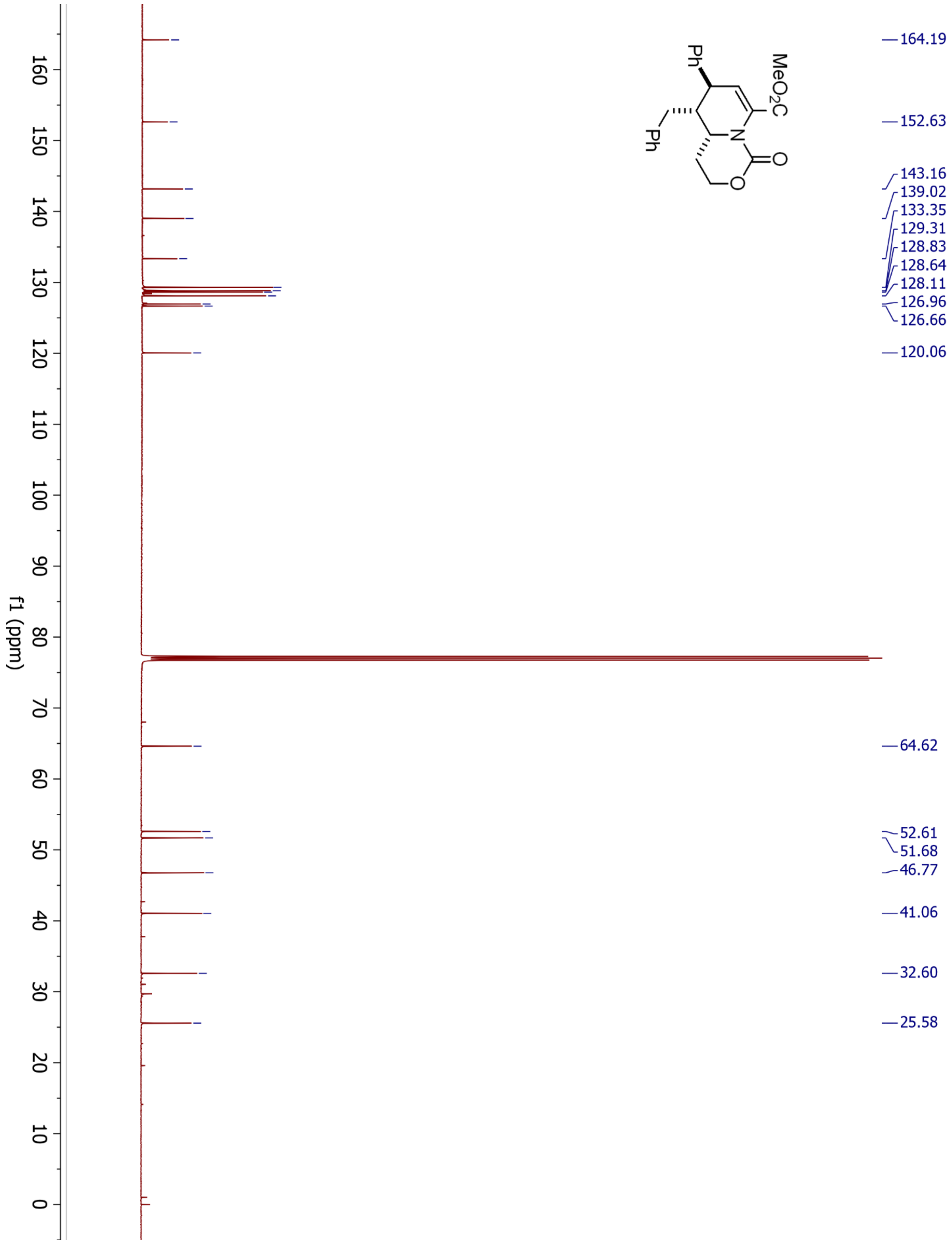

Supplementary Figure 42. ^{1}H NMR (500 MHz, $CDCl_3$) for compound **4ba**

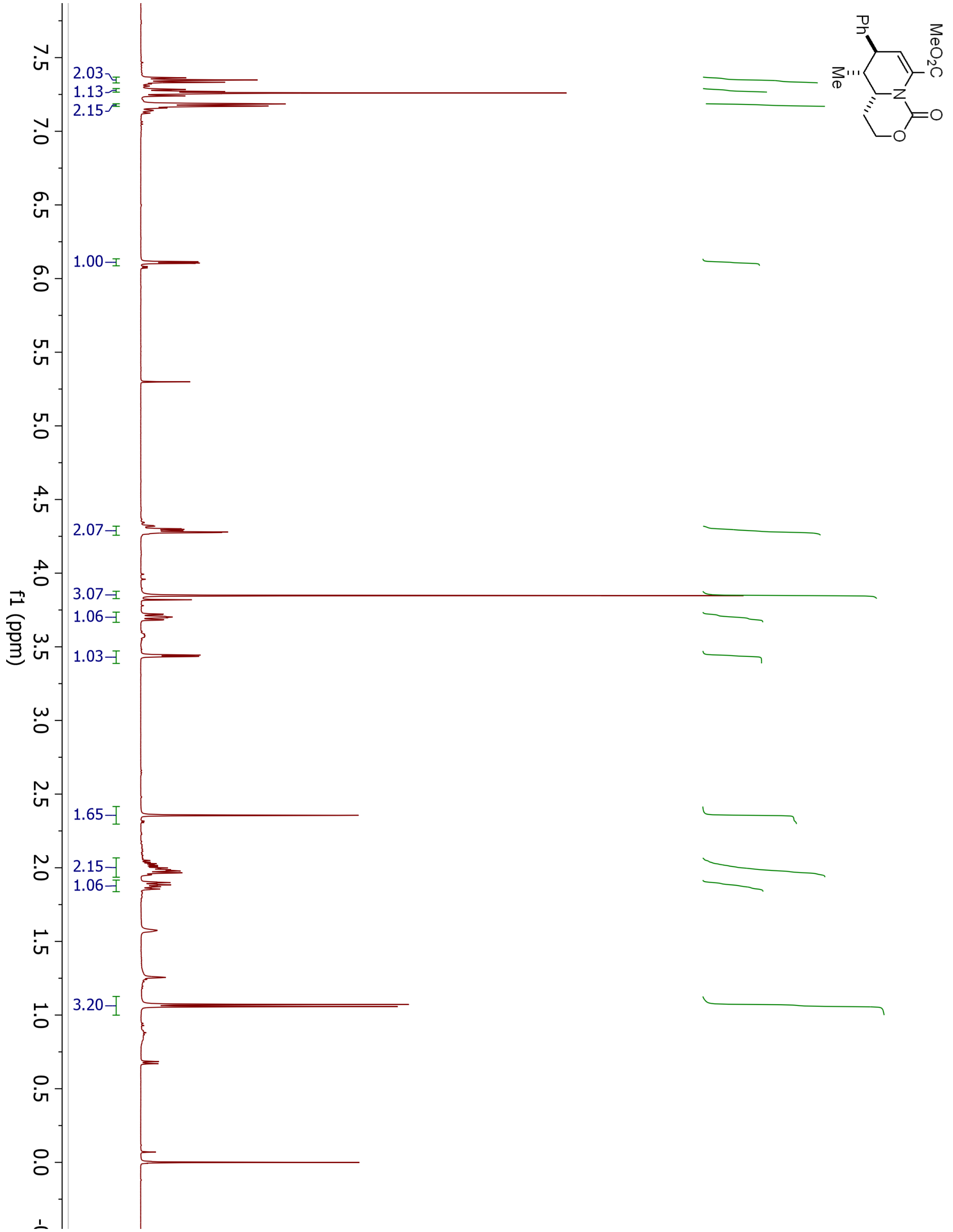

Supplementary Figure 43. ^{13}C NMR (126 MHz, $CDCl_3$) for compound **4ba**

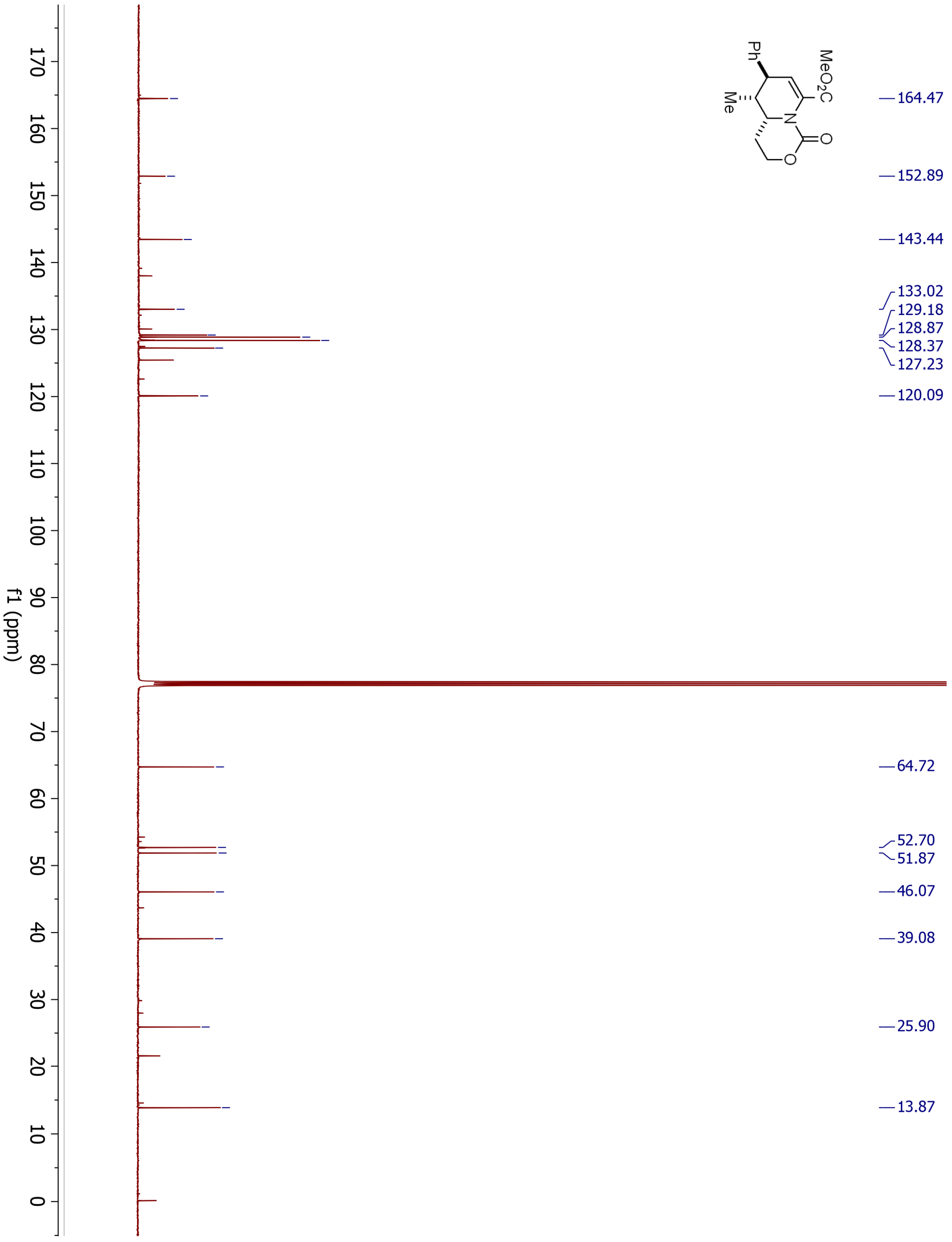

Supplementary Figure 44. ^{1}H NMR (500 MHz, $CDCl_3$) for compound **4ca**

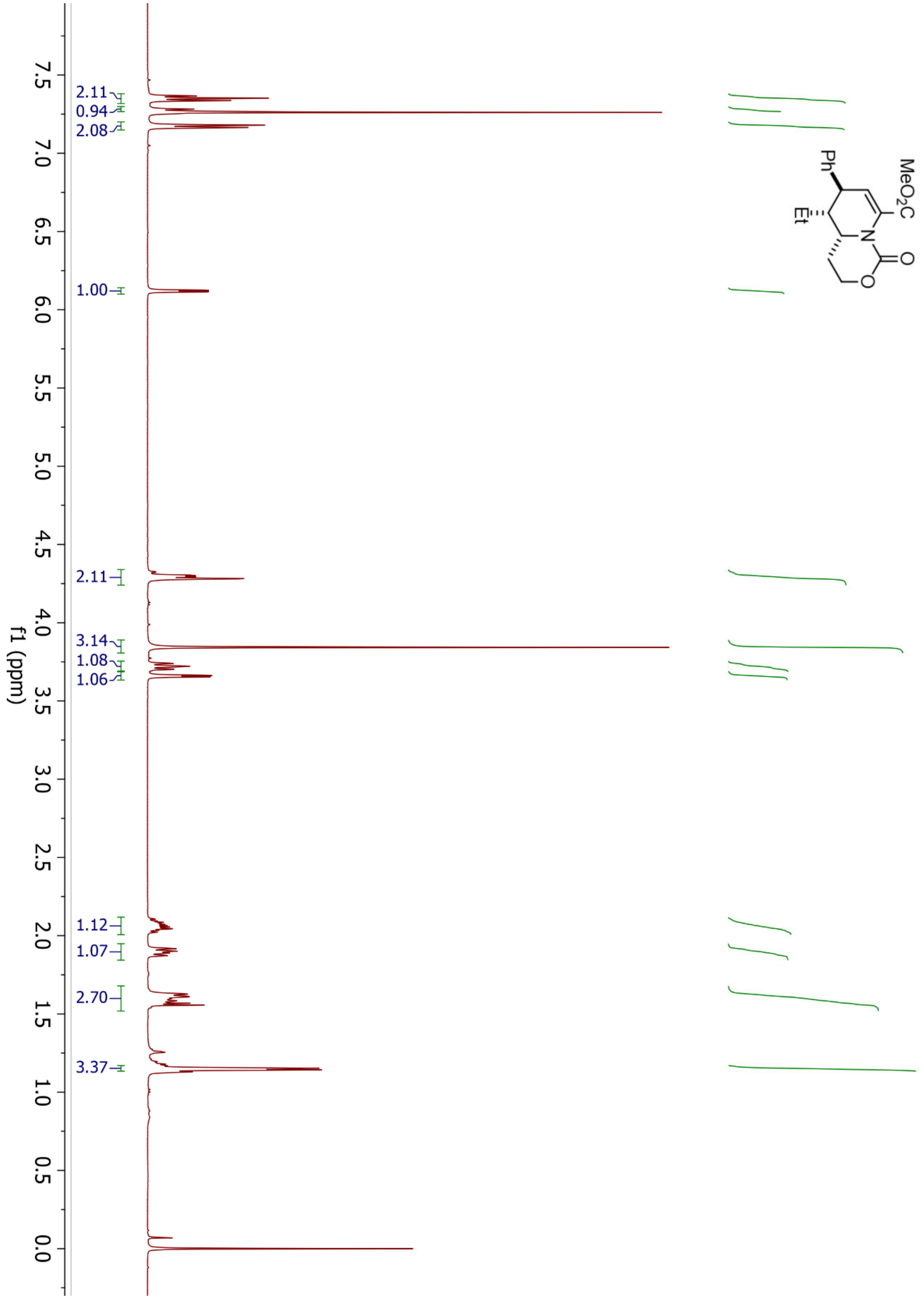

Supplementary Figure 45. ^{13}C NMR (126 MHz, $CDCl_3$) for compound **4ca**

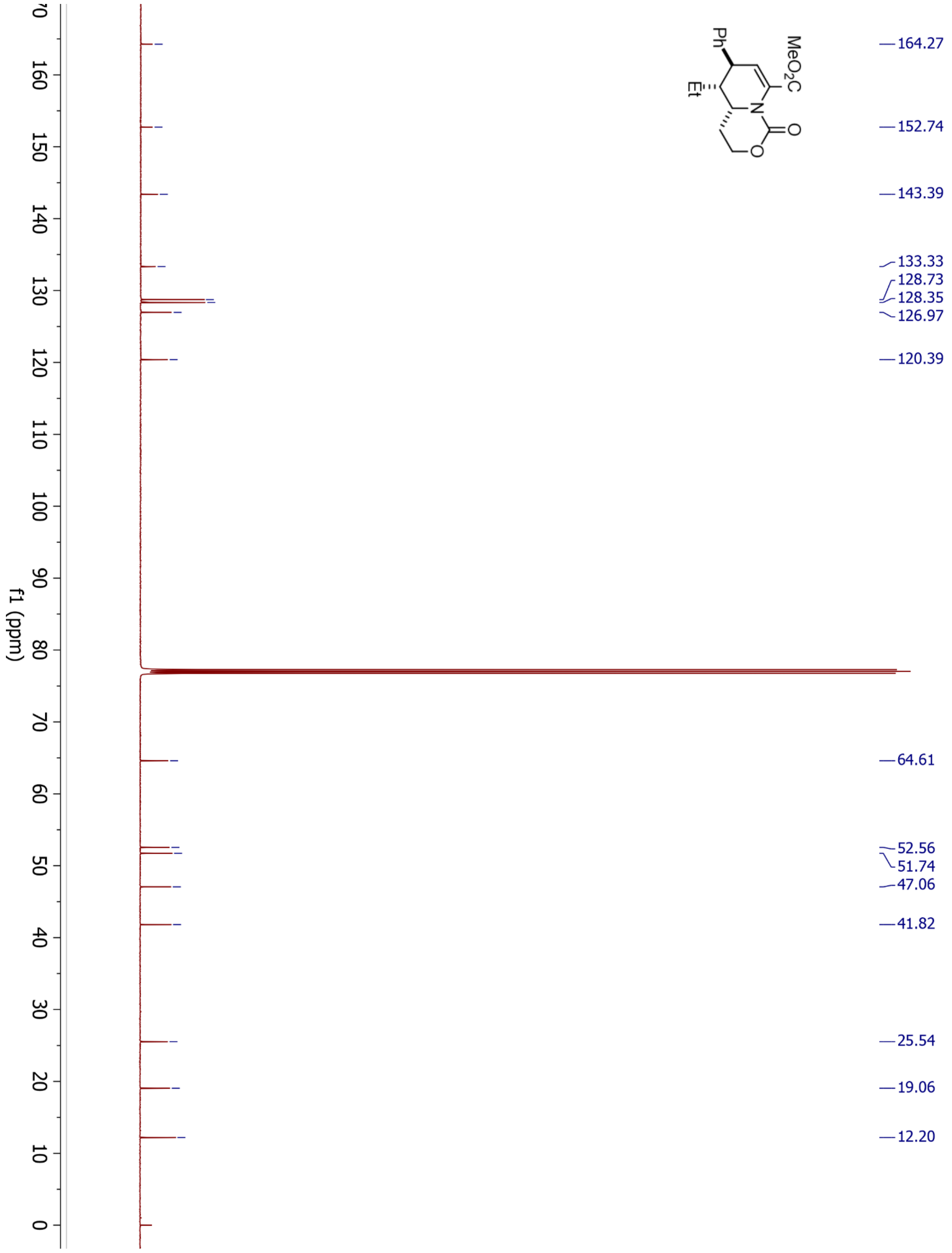

Supplementary Figure 46. ^{1}H NMR (500 MHz, $CDCl_3$) for compound **4da**

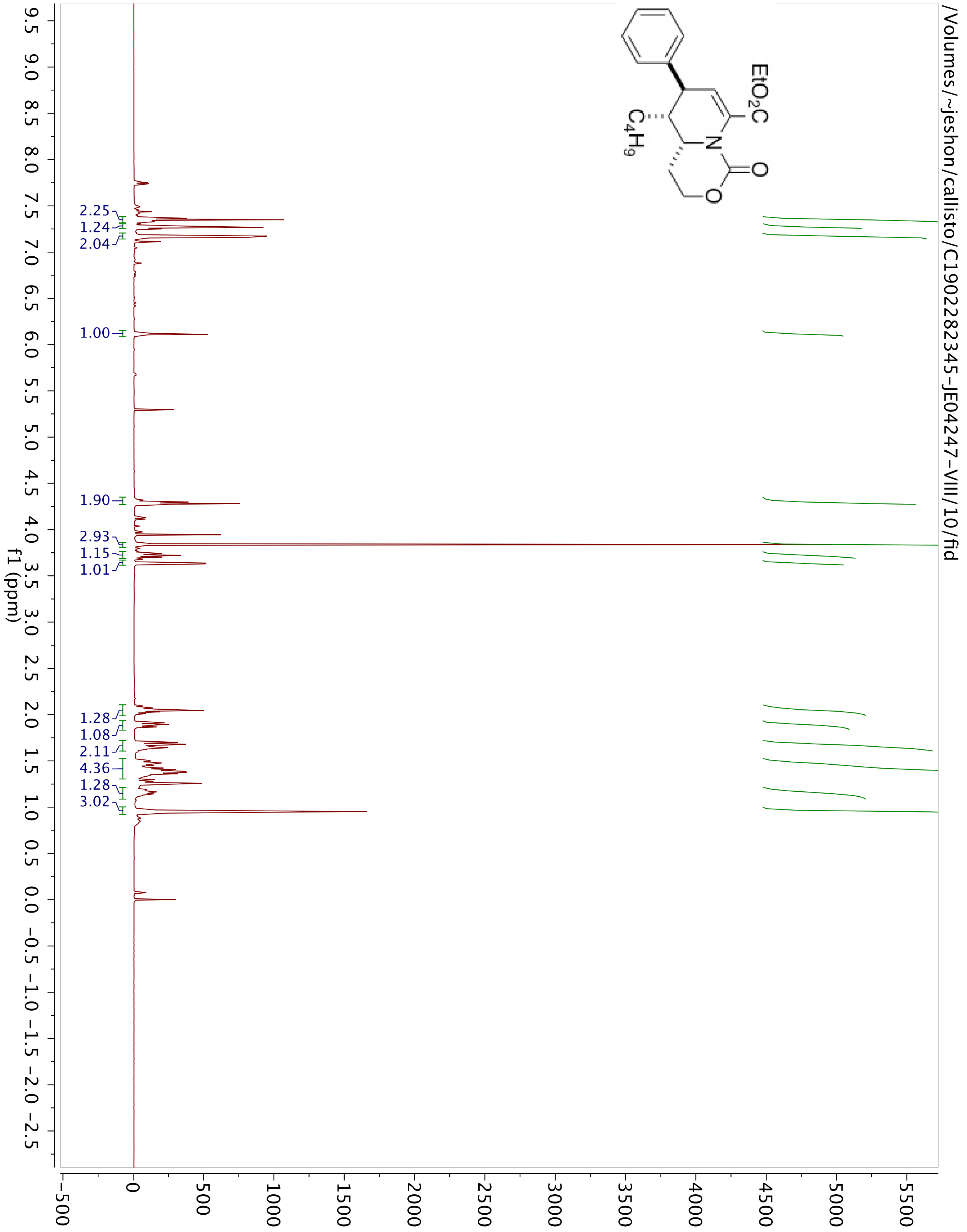

Supplementary Figure 47. ^{13}C NMR (126 MHz, $CDCl_3$) for compound **4da**

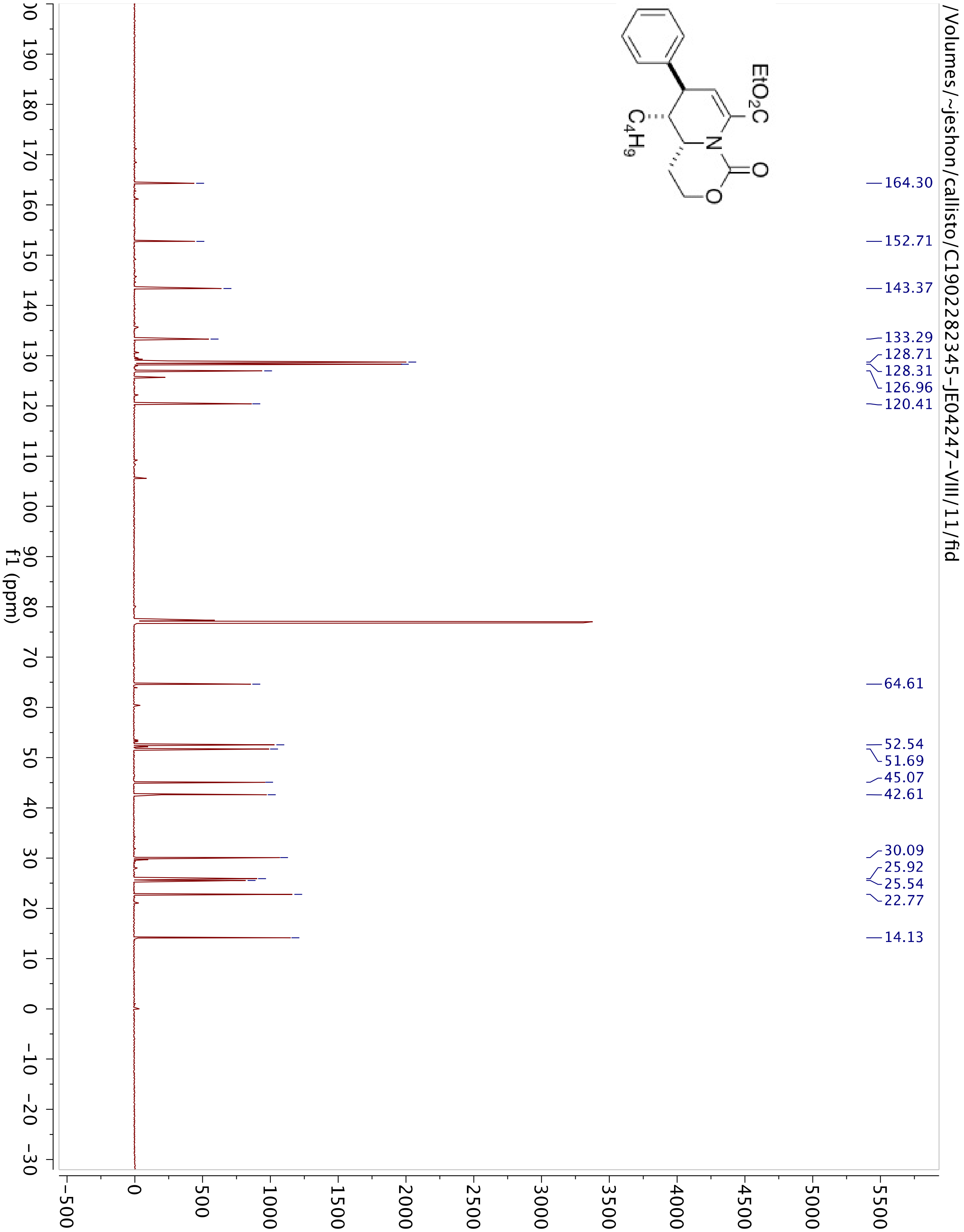

Supplementary Figure 48. ^{1}H NMR (500 MHz, $CDCl_3$) for compound **4ea**

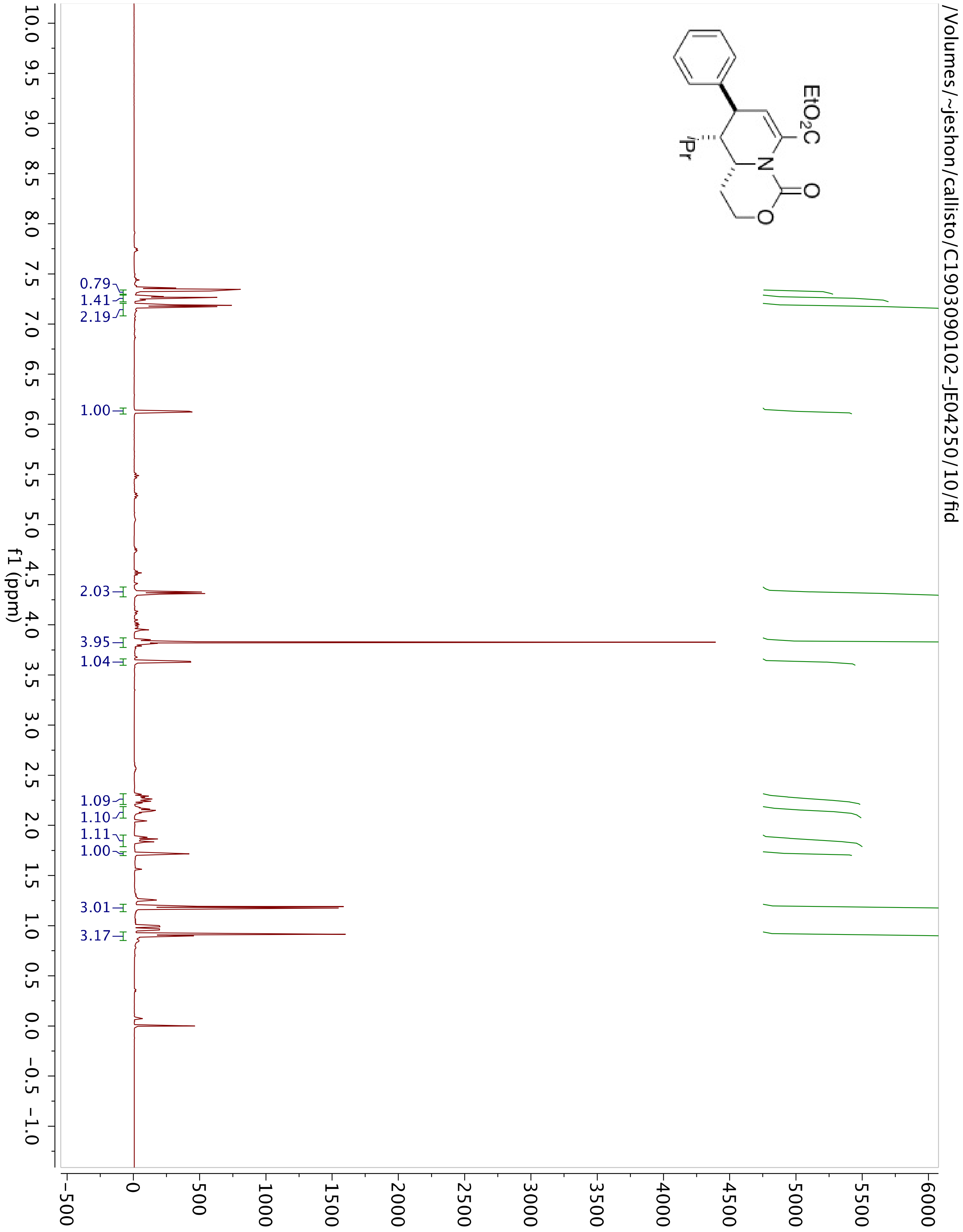

Supplementary Figure 49. ^{13}C NMR (126 MHz, $CDCl_3$) for compound **4ea**

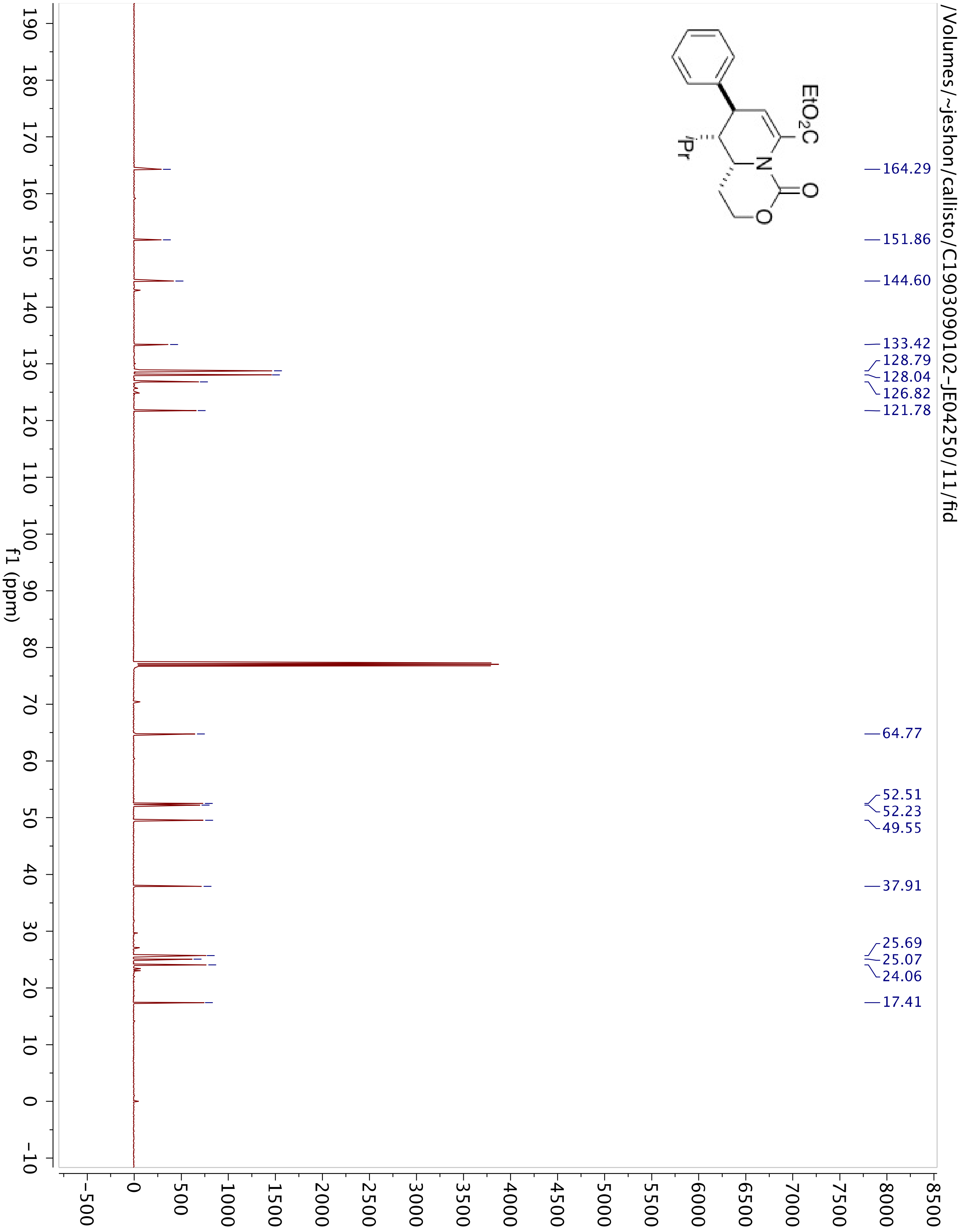

Supplementary Figure 50. ^{1}H NMR (500 MHz, $CDCl_3$) for compound **4fa**

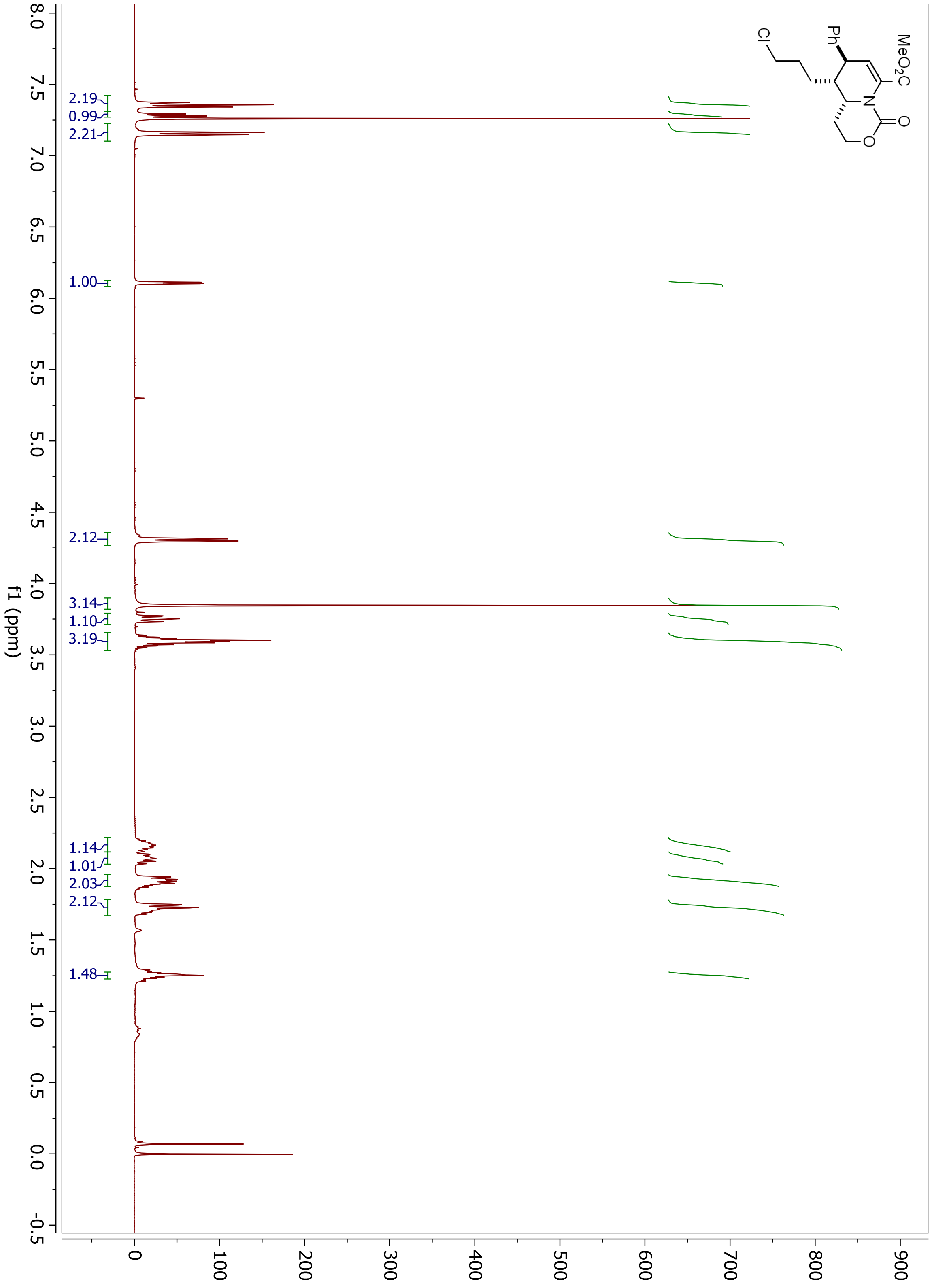

Supplementary Figure 51. ^{13}C NMR (126 MHz, $CDCl_3$) for compound **4fa**

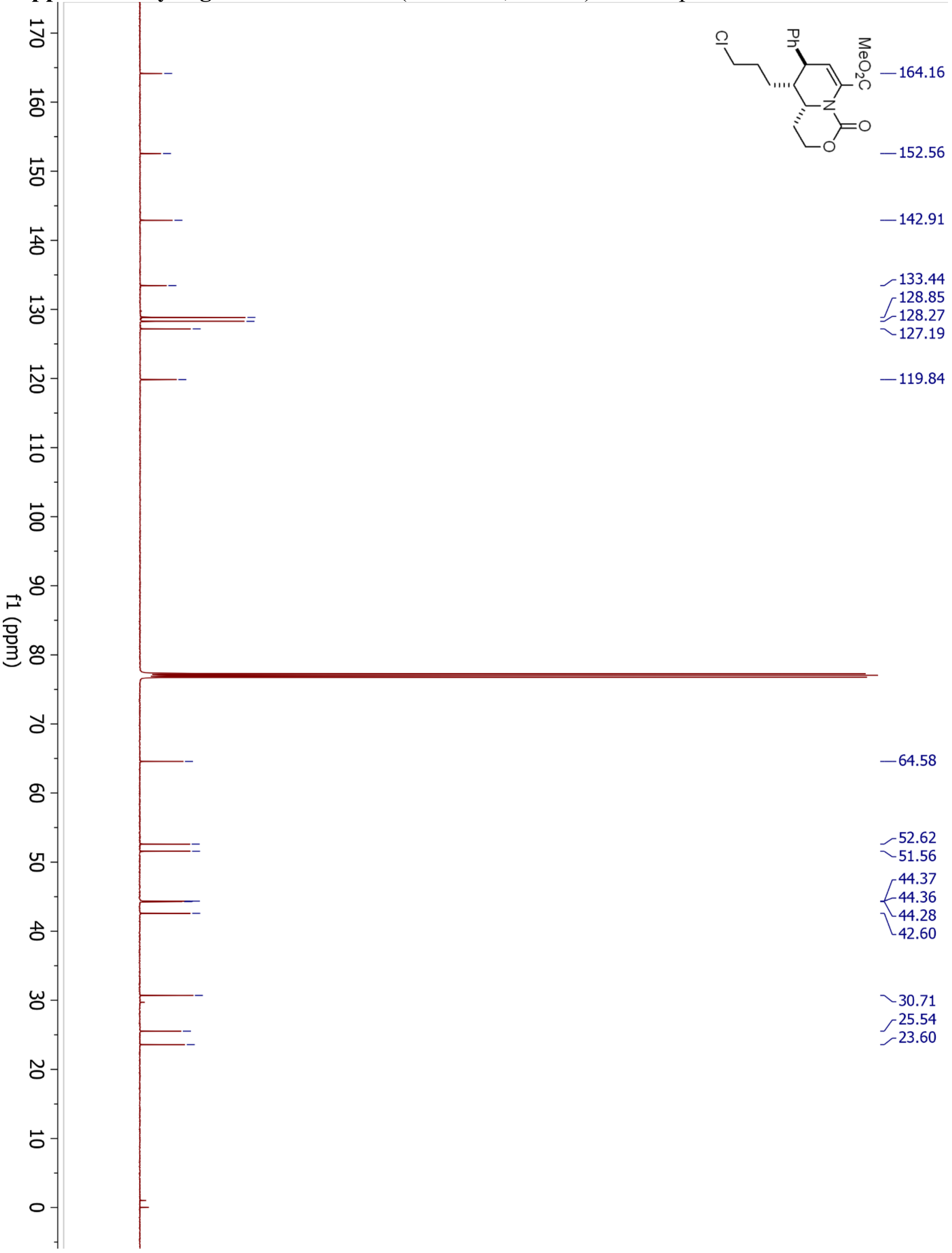

Supplementary Figure 52. ^{1}H NMR (500 MHz, $CDCl_3$) for compound **4ga**

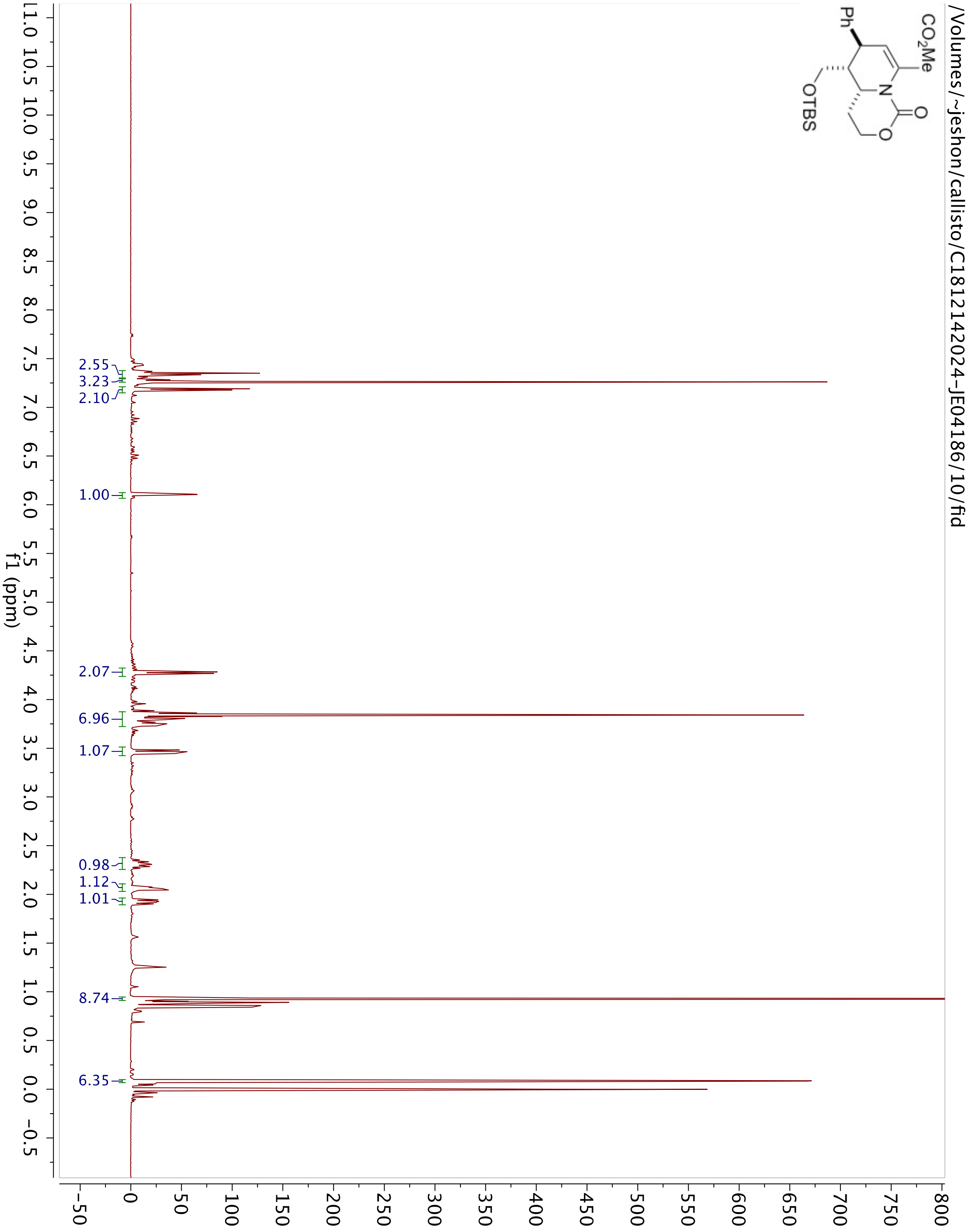

Supplementary Figure 53. ^{13}C NMR (126 MHz, $CDCl_3$) for compound **4ga**

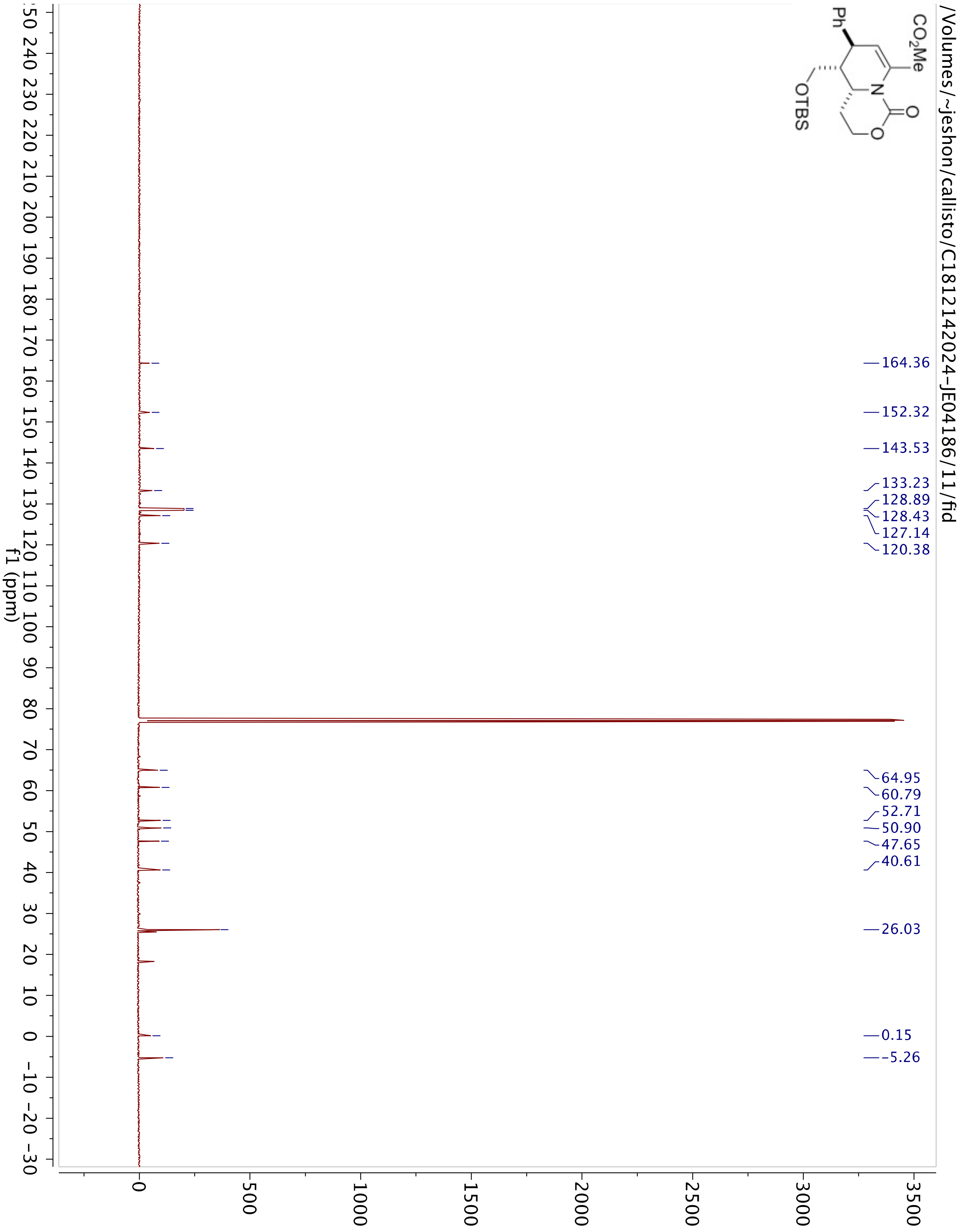

Supplementary Figure 54. ^{1}H NMR (500 MHz, $CDCl_3$) for compound **4ha**

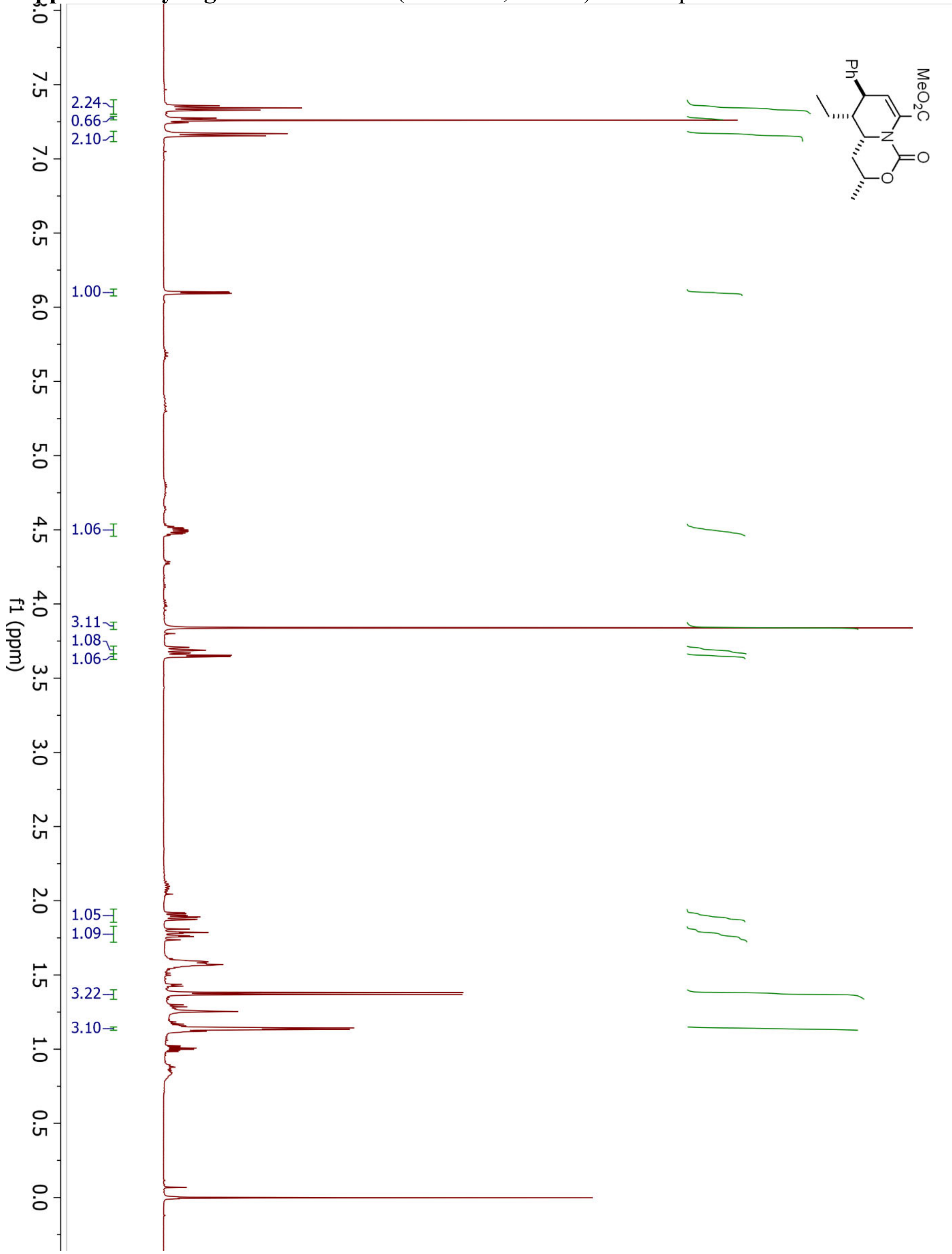

Supplementary Figure 55. ^{13}C NMR (126 MHz, $CDCl_3$) for compound **4ha**

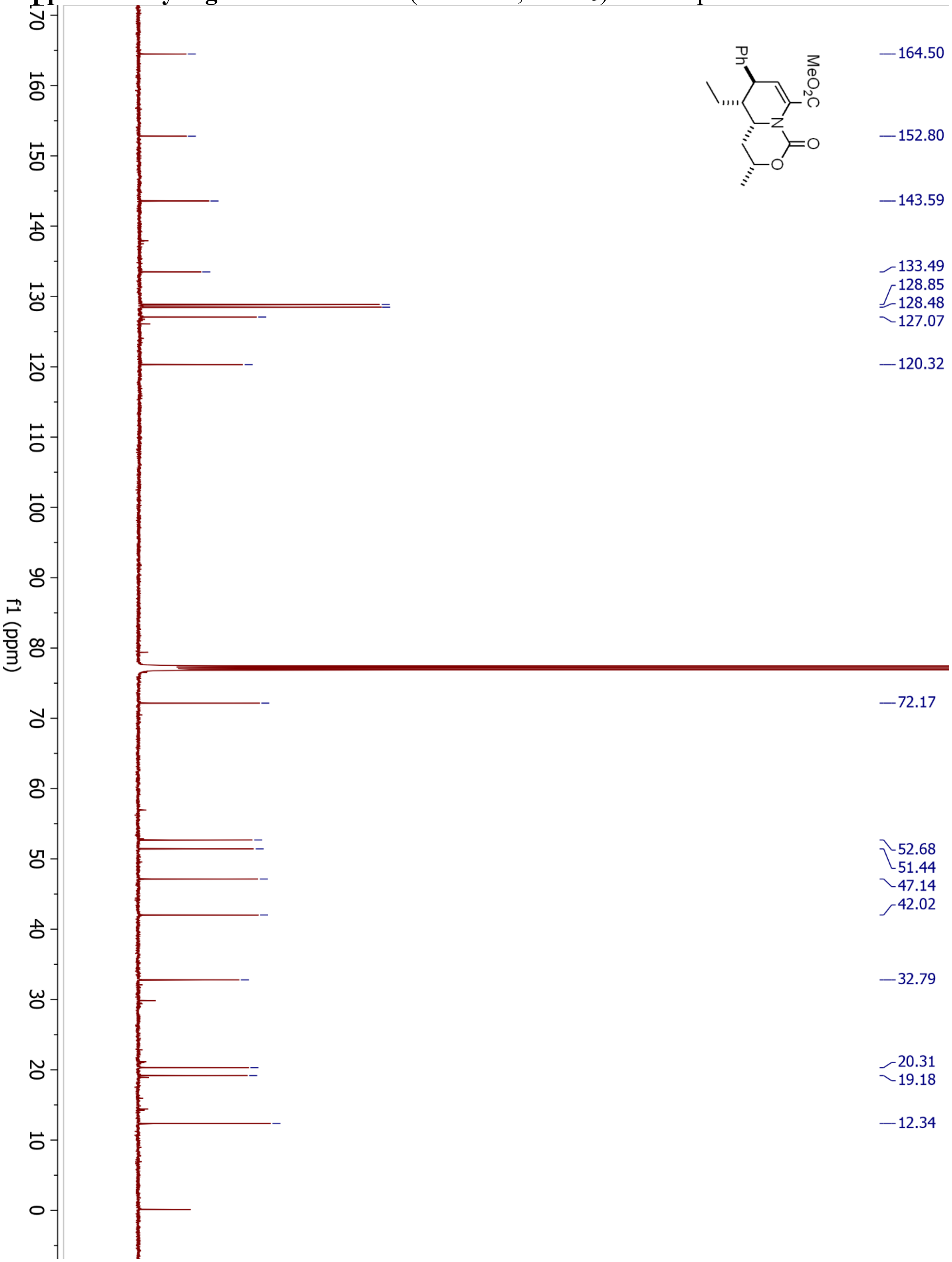

Supplementary Figure 56. ^{1}H NMR (500 MHz, $CDCl_3$) for compound **4ia**

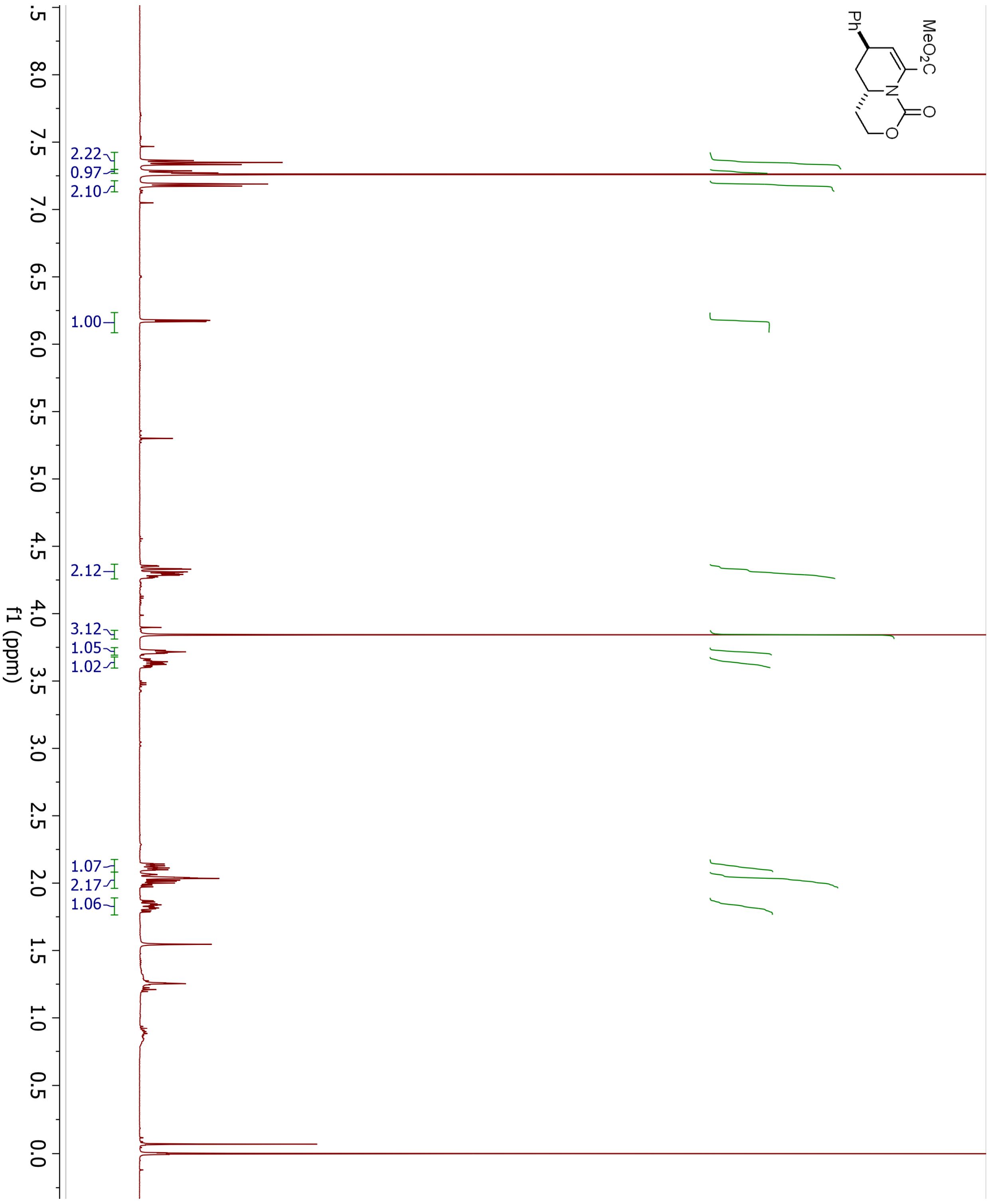

Supplementary Figure 57. ^{13}C NMR (126 MHz, $CDCl_3$) for compound **4ia**

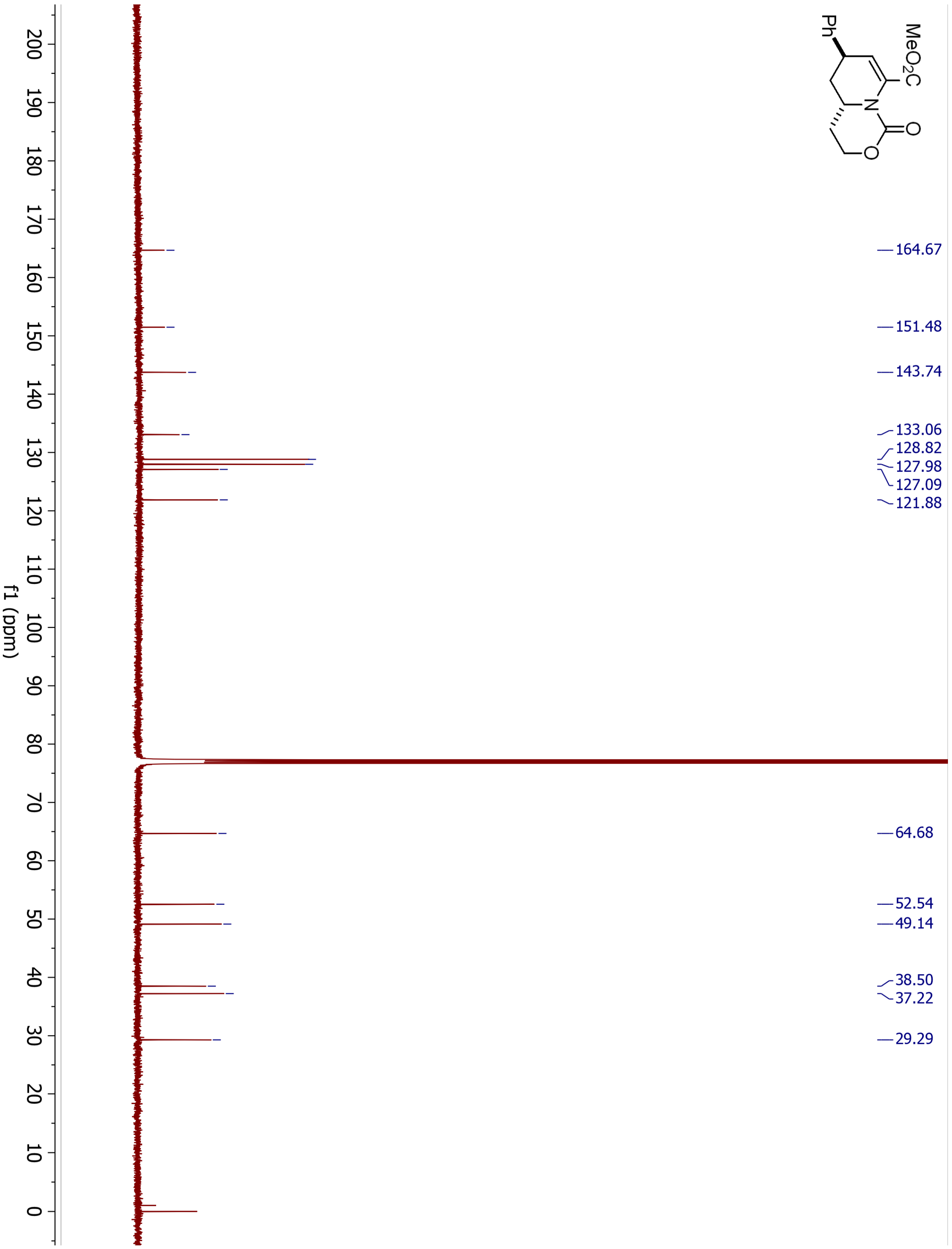

Supplementary Figure 58. ^{1}H NMR (500 MHz, $CDCl_3$) for compound **4ja**

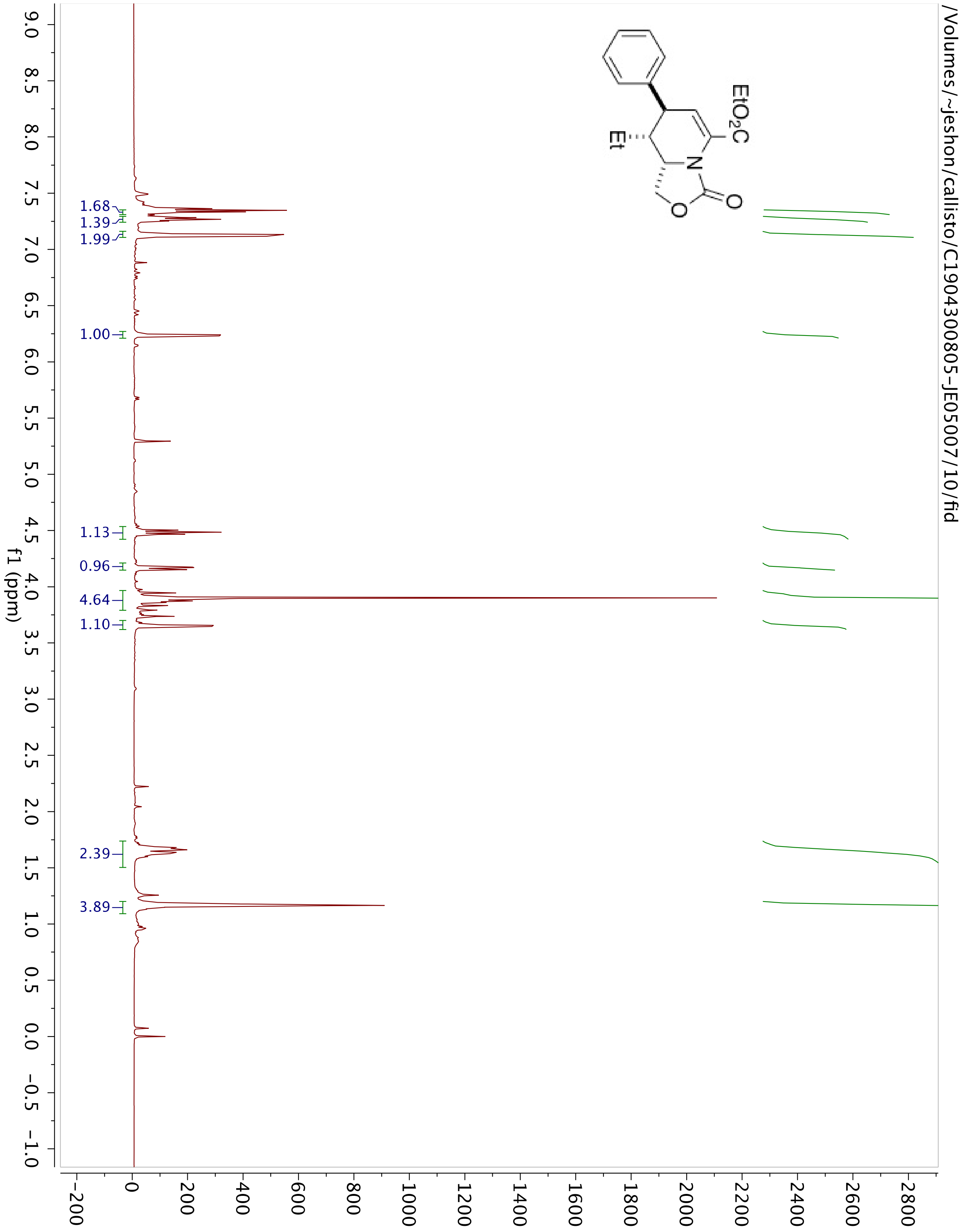

Supplementary Figure 59. ^{13}C NMR (126 MHz, $CDCl_3$) for compound **4ja**

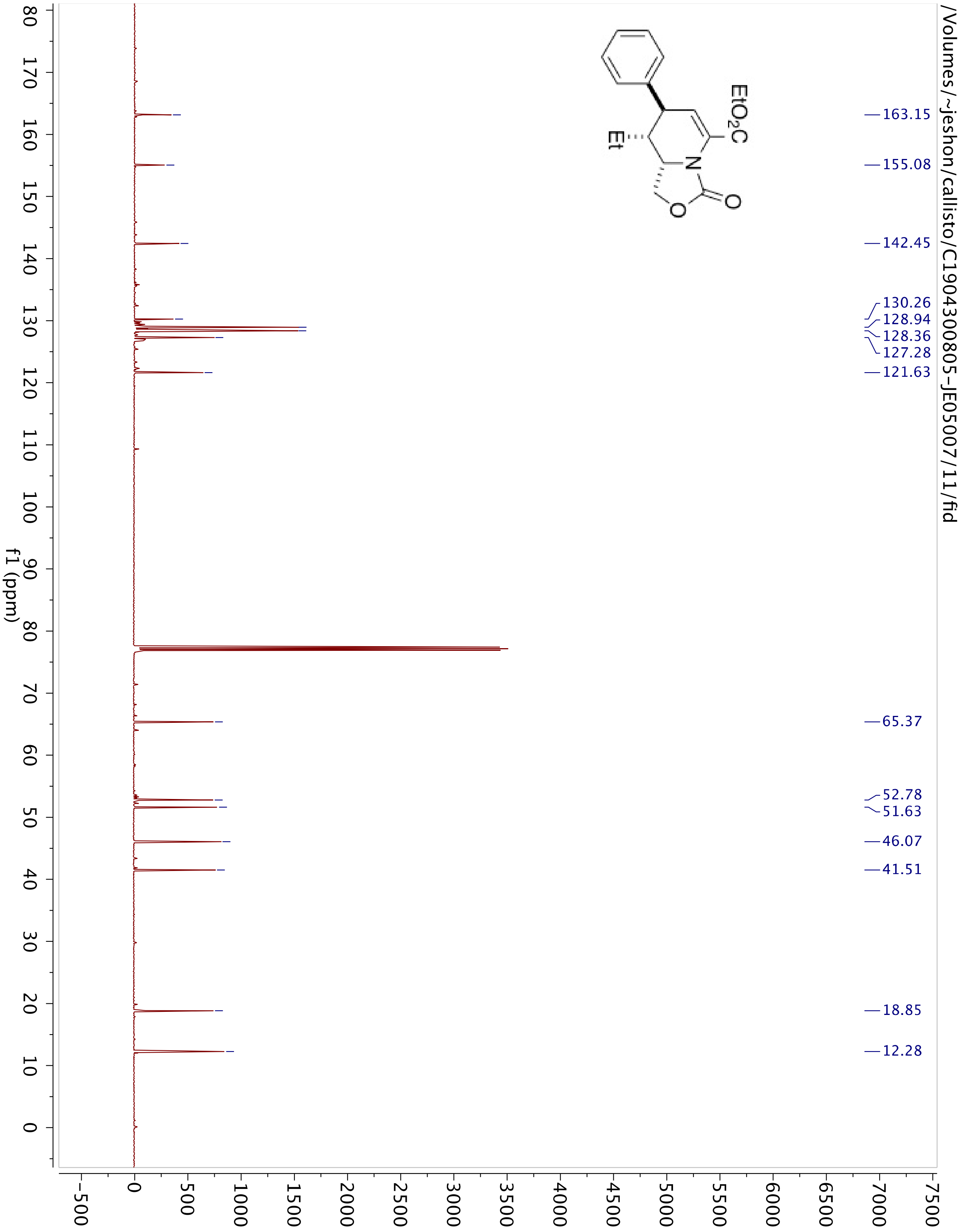

Supplementary Figure 60. ^{1}H NMR (500 MHz, $CDCl_3$) for compound **4cb**

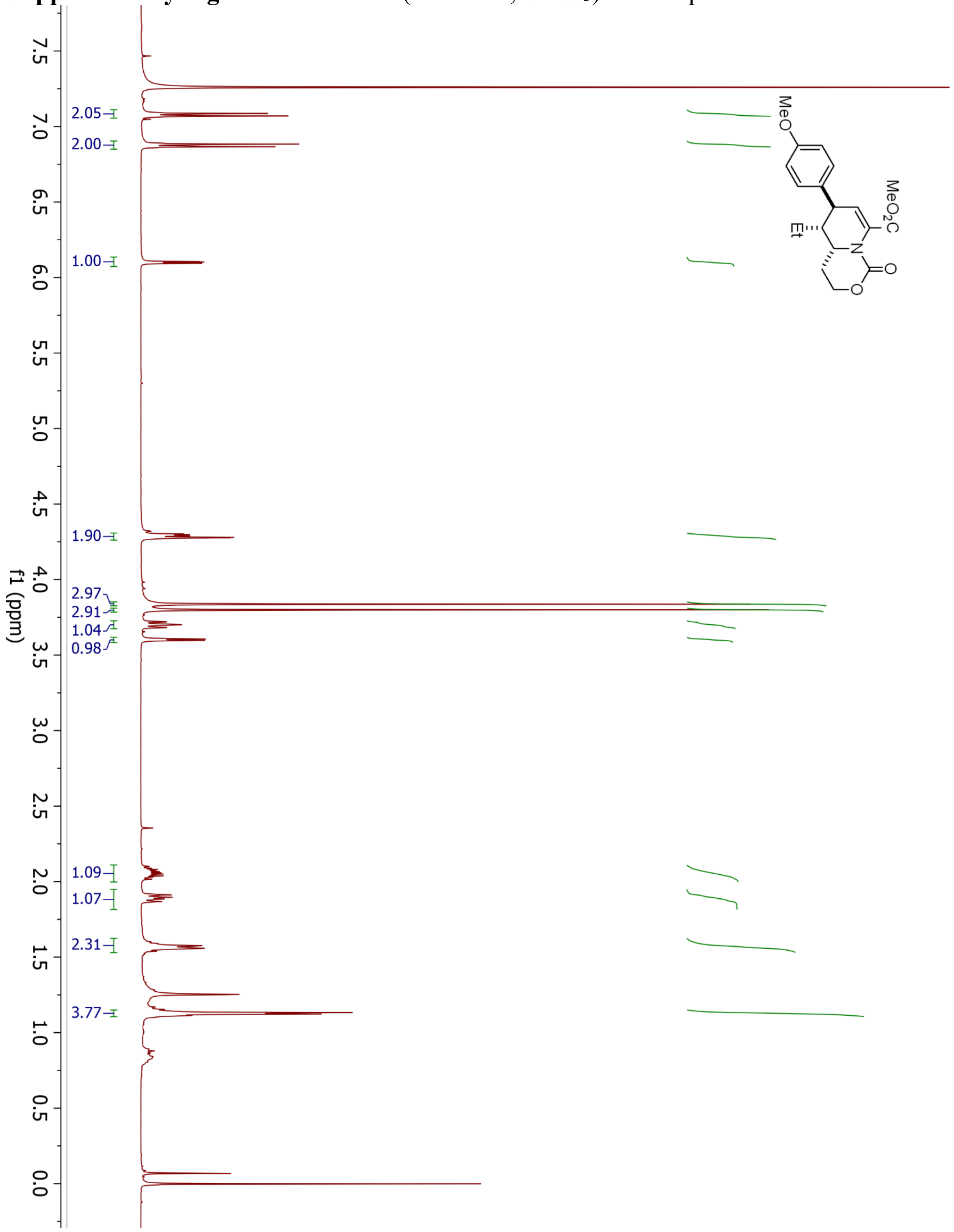

Supplementary Figure 61. ^{13}C NMR (126 MHz, $CDCl_3$) for compound **4cb**

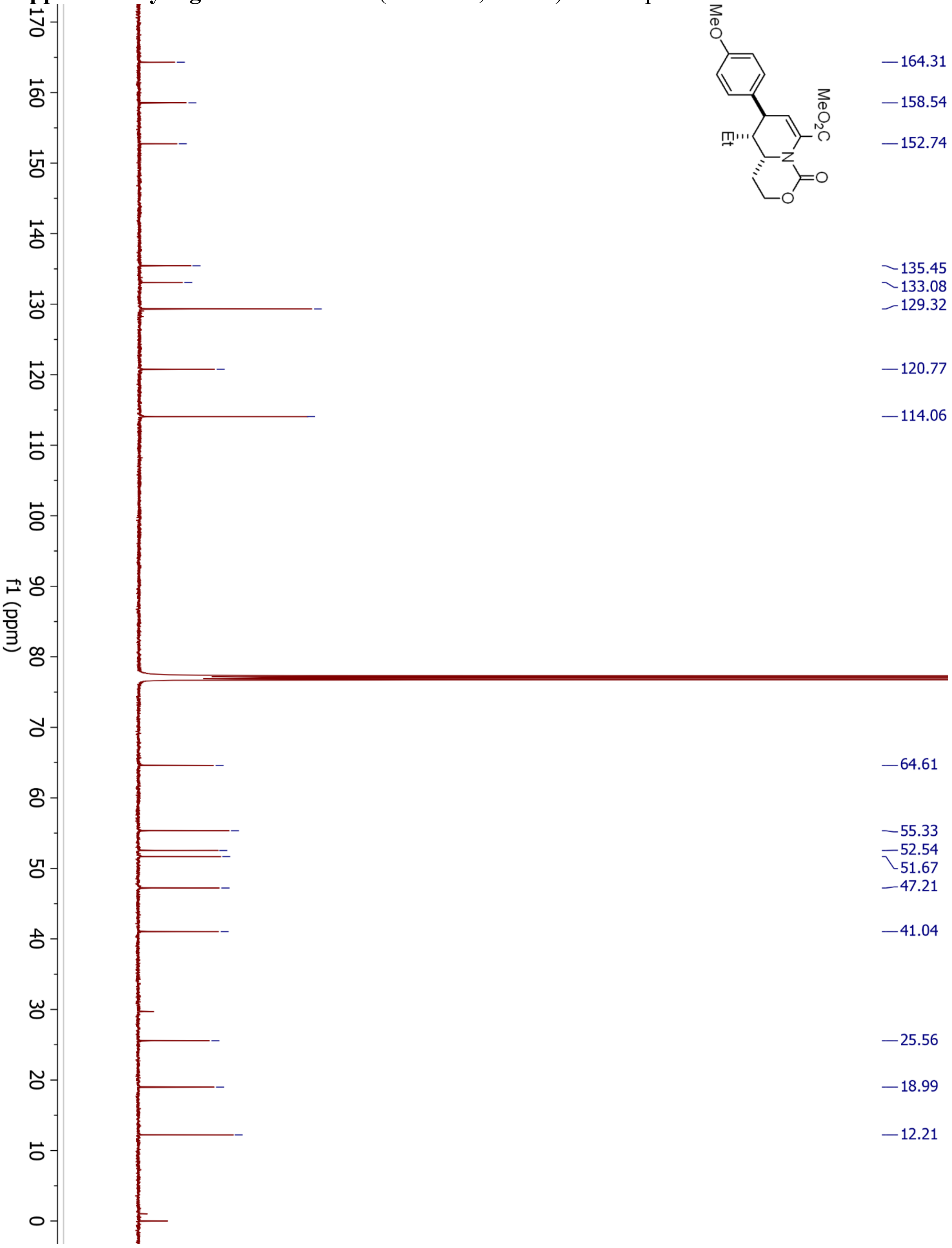

Supplementary Figure 62. ^{1}H NMR (500 MHz, $CDCl_3$) for compound **4cc**

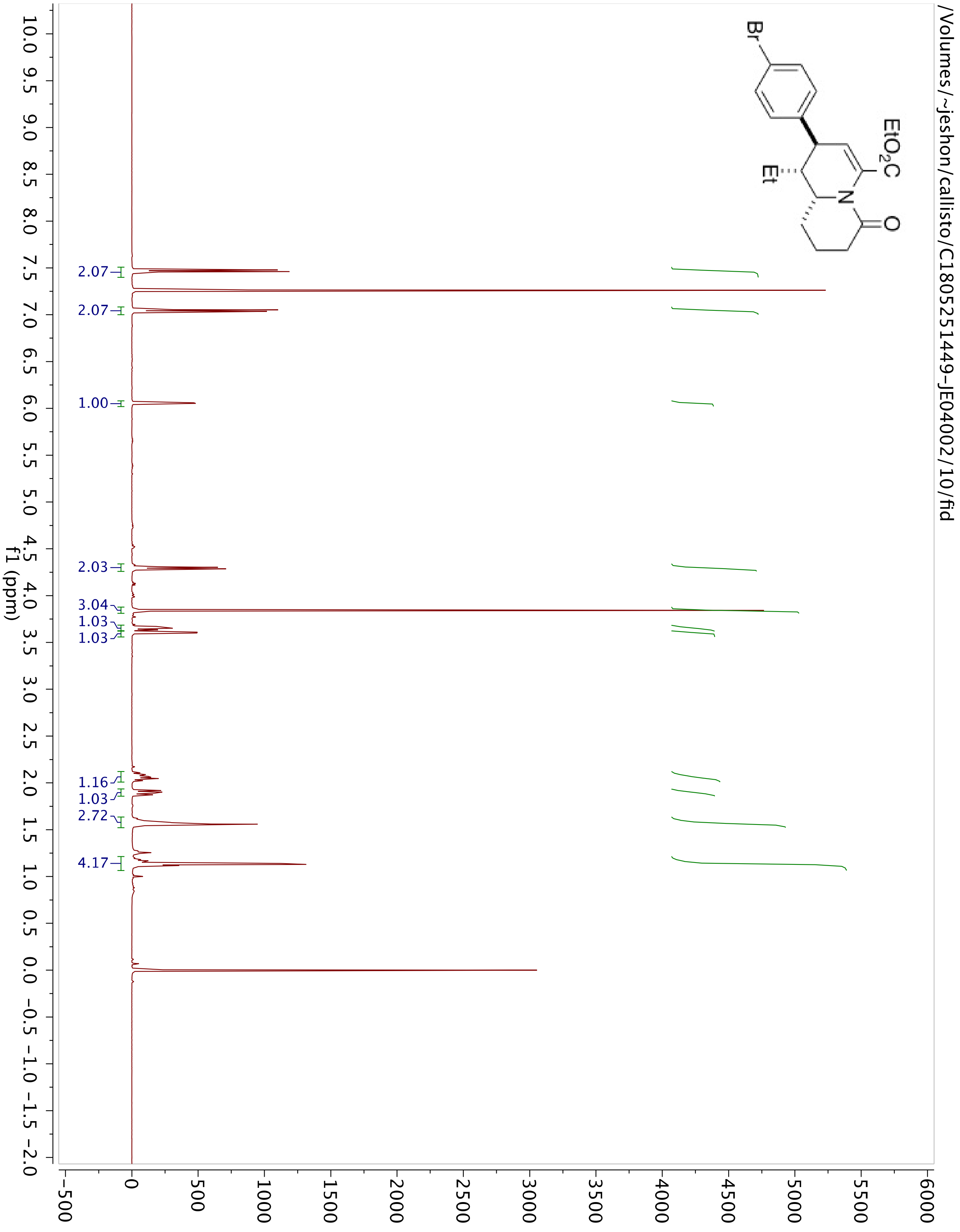

Supplementary Figure 63. ^{13}C NMR (126 MHz, $CDCl_3$) for compound **4cc**

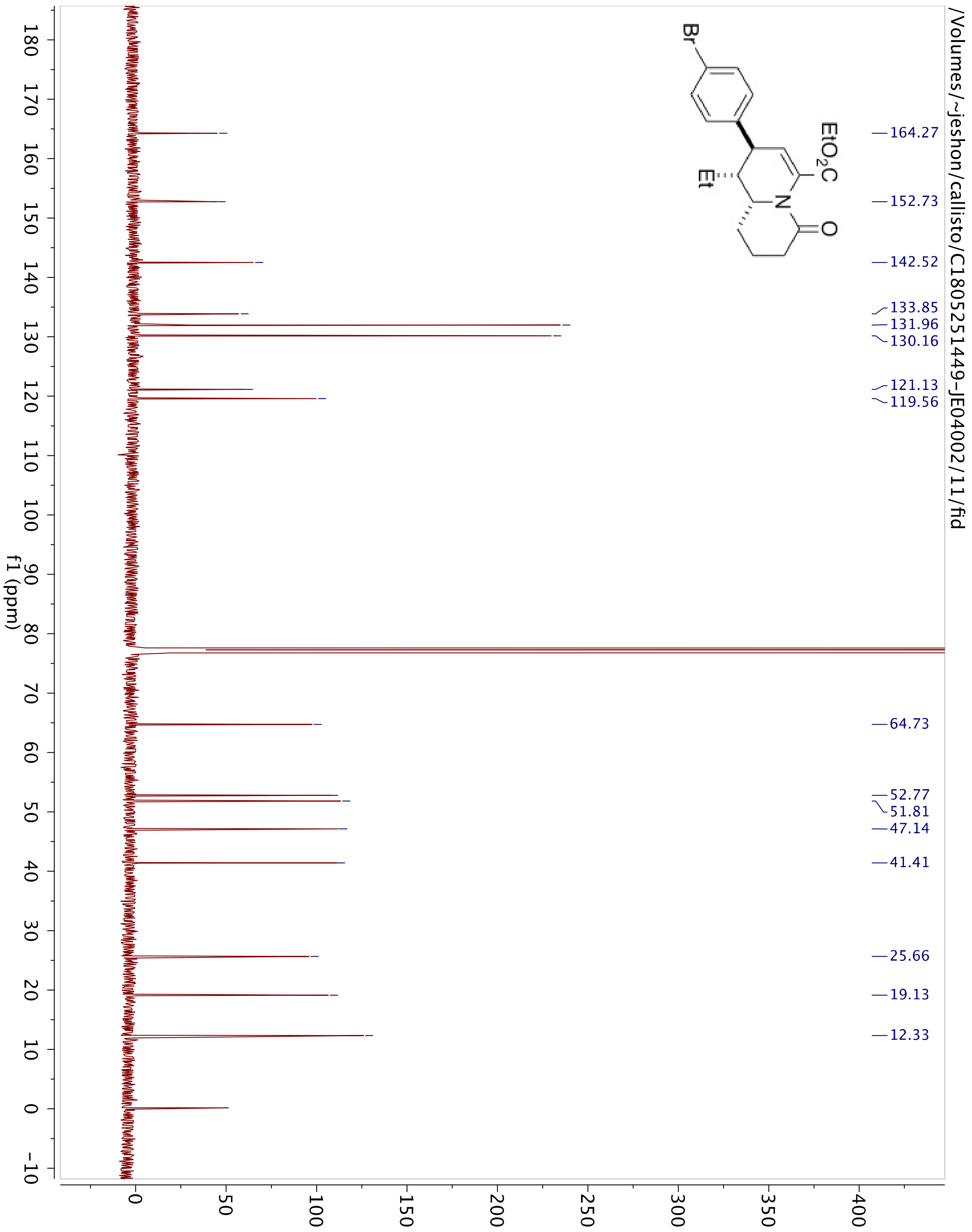

Supplementary Figure 64. ^{1}H NMR (500 MHz, $CDCl_3$) for compound **4cd**

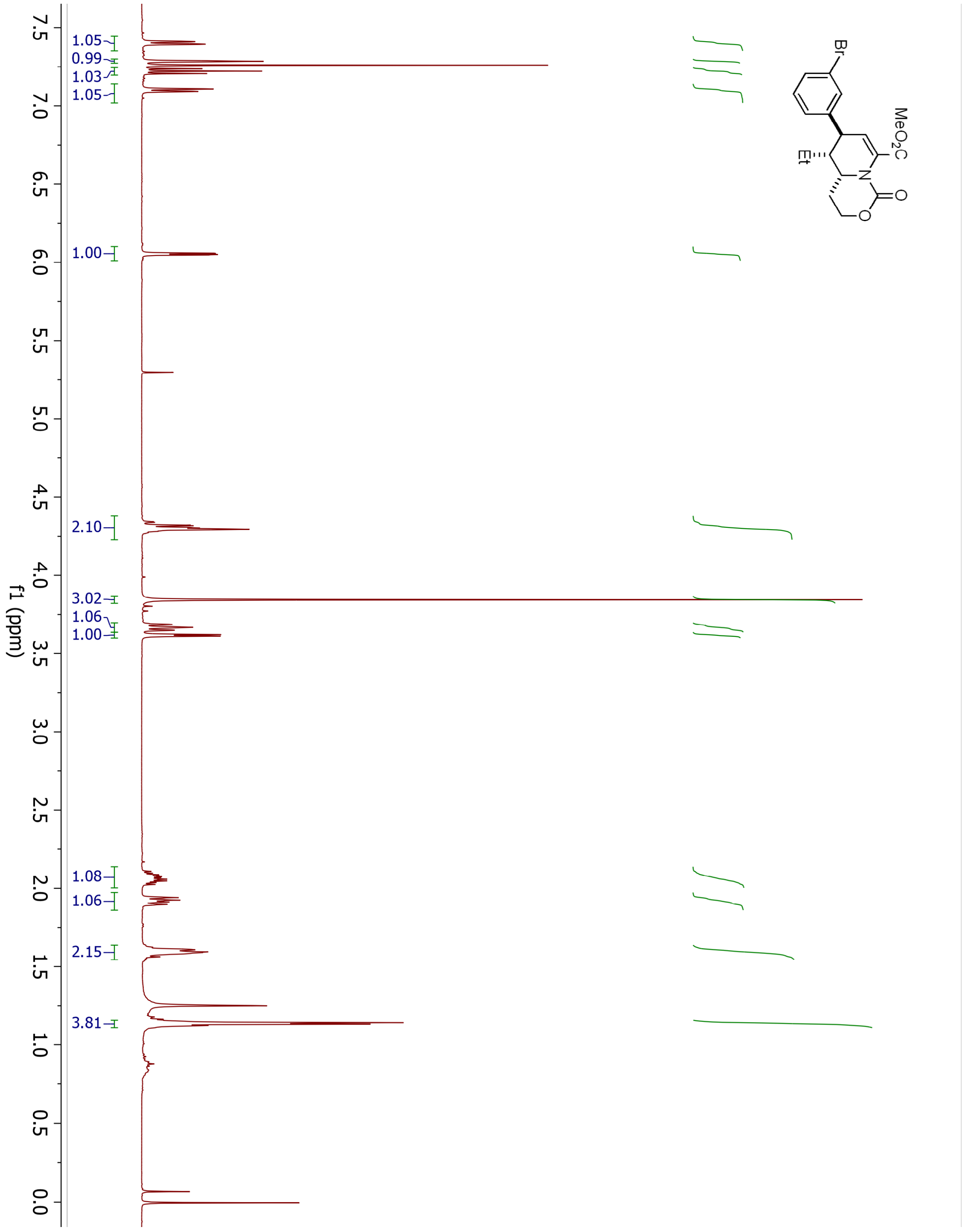

Supplementary Figure 65. ^{13}C NMR (126 MHz, $CDCl_3$) for compound **4cd**

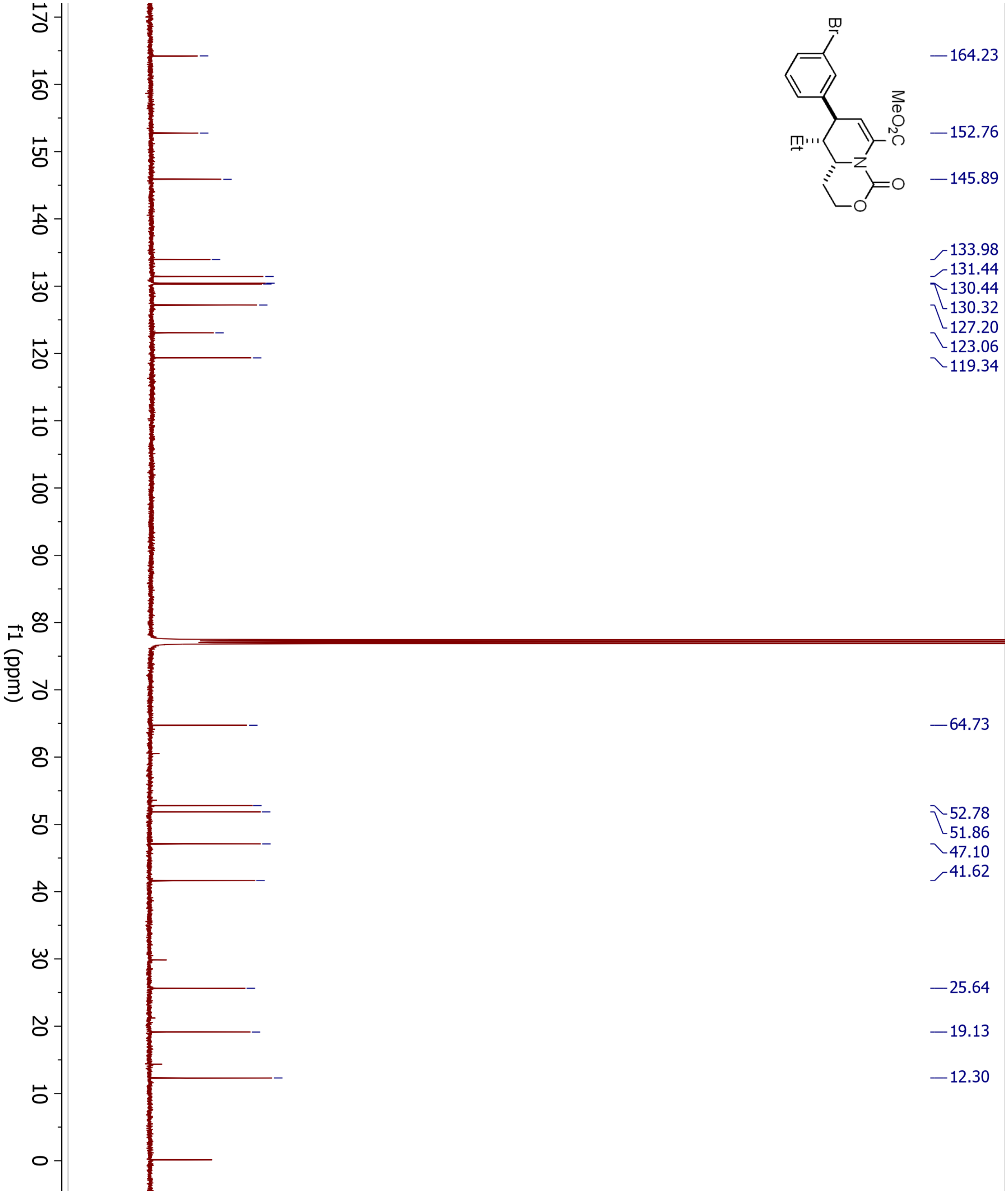

Supplementary Figure 66. ^{1}H NMR (500 MHz, $CDCl_3$) for compound **4ce**

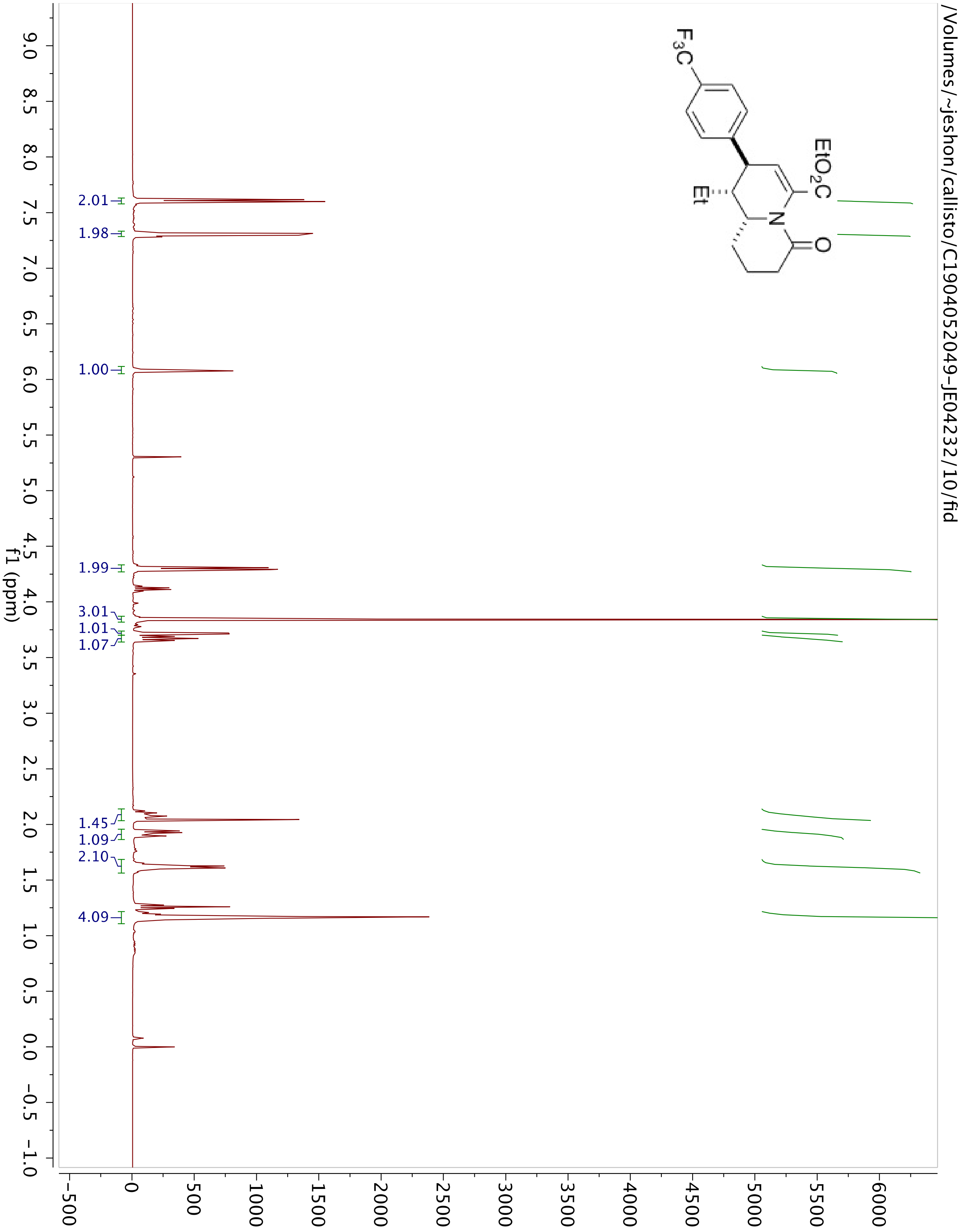

Supplementary Figure 67. ^{13}C NMR (126 MHz, $CDCl_3$) for compound **4ce**

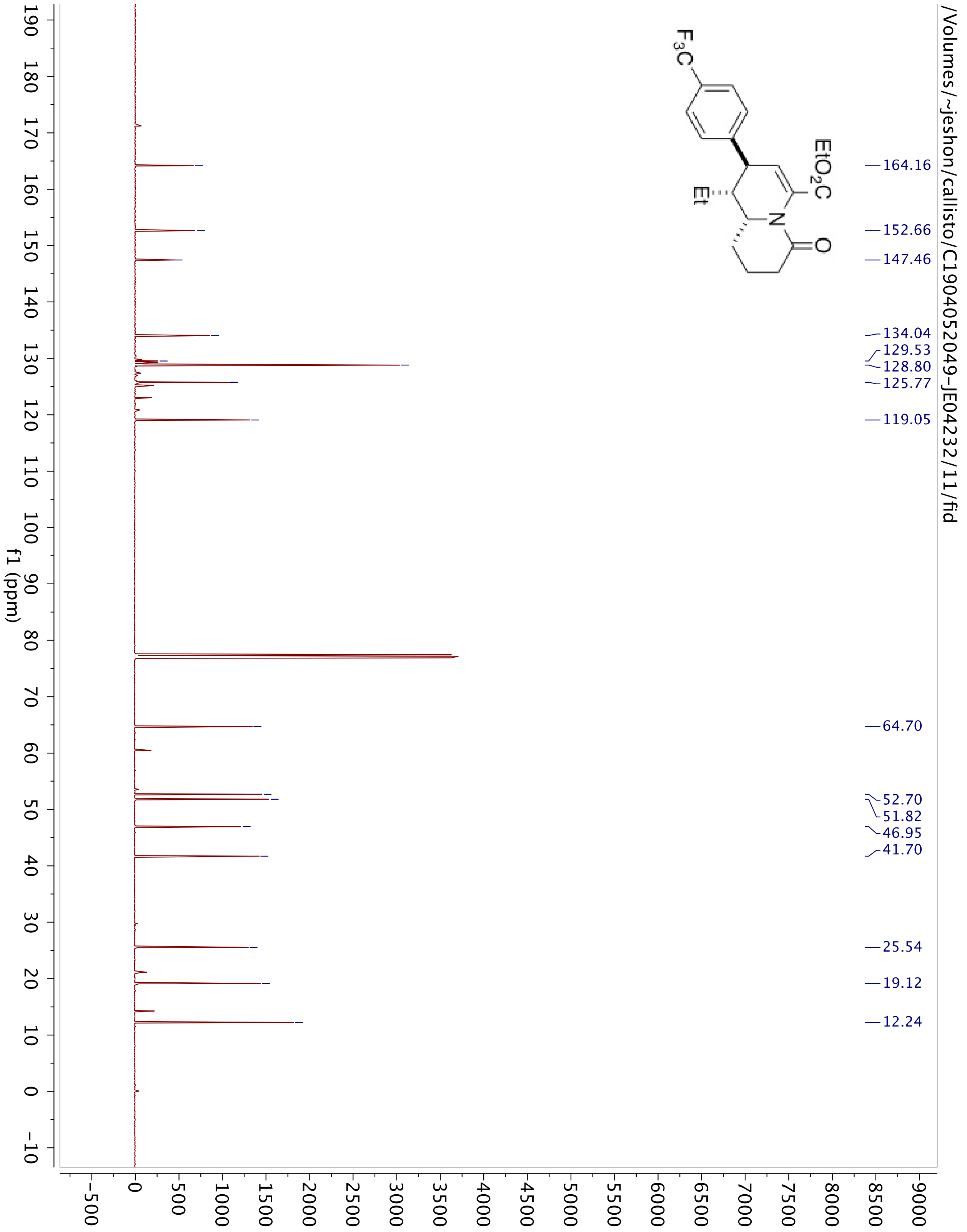

Supplementary Figure 68. ^1H NMR (500 MHz, $CDCl_3$) for compound **4cf**

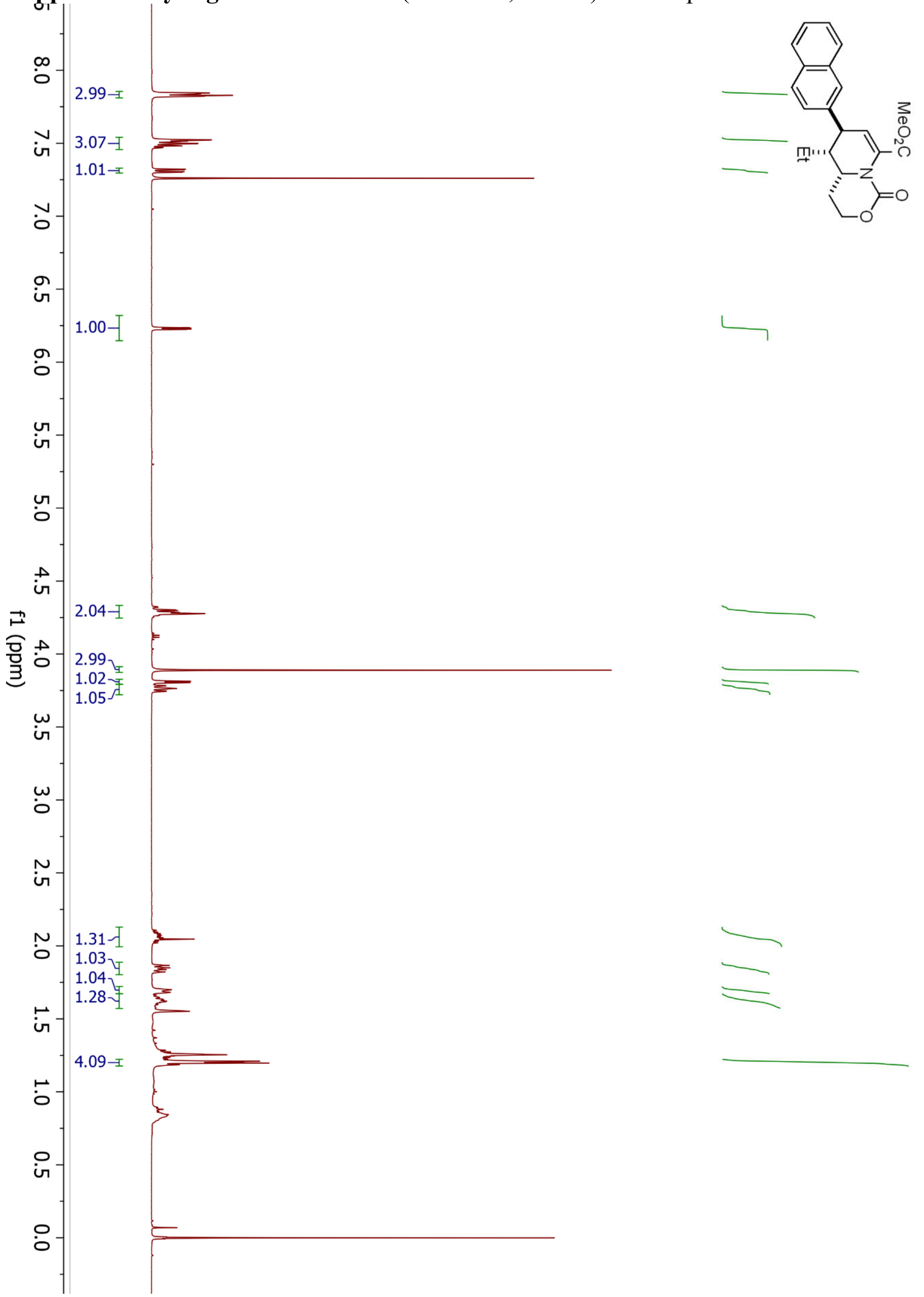

Supplementary Figure 69. ^{13}C NMR (126 MHz, $CDCl_3$) for compound **4cf**

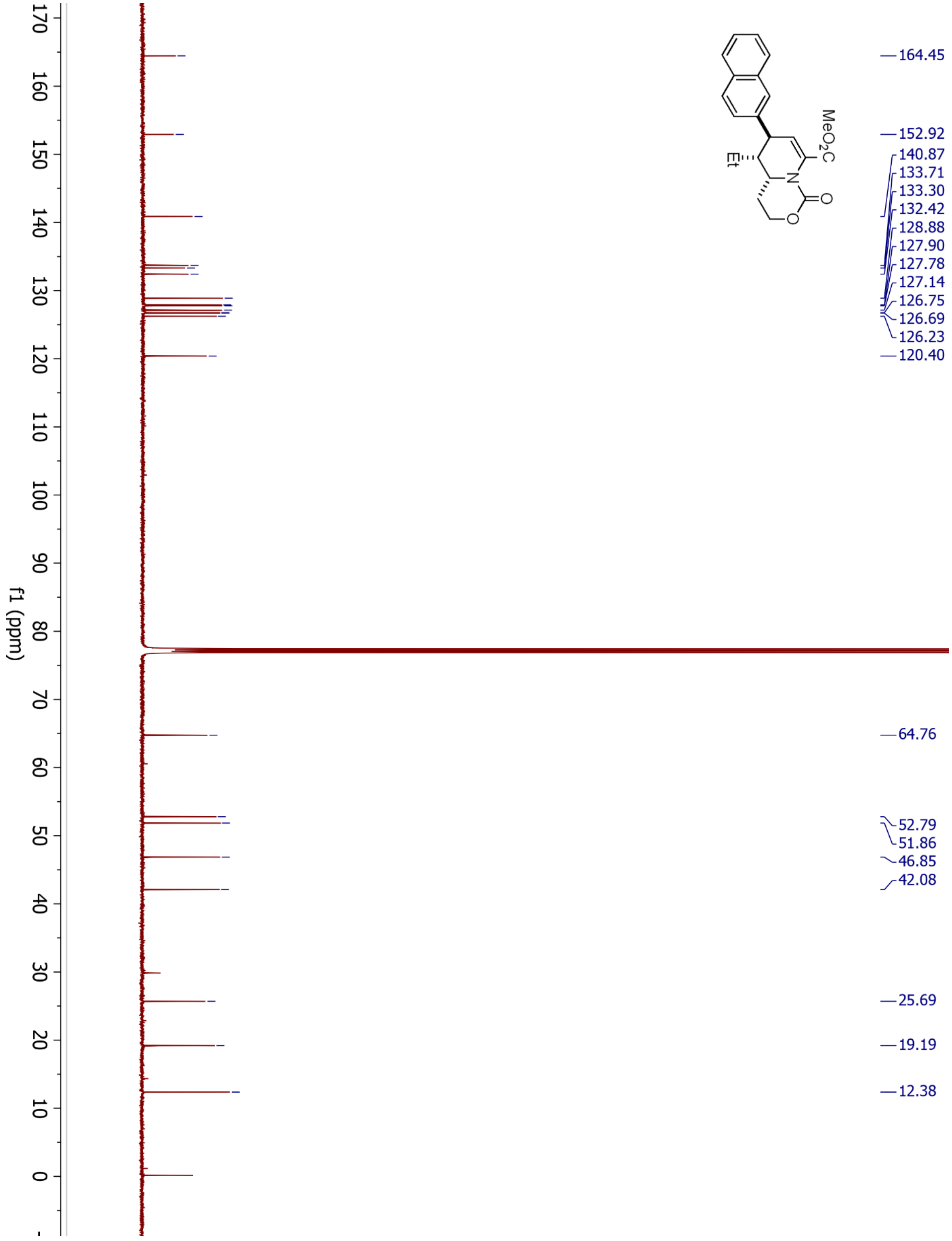

Supplementary Figure 70. ^{1}H NMR (500 MHz, $CDCl_3$) for compound **4cg**

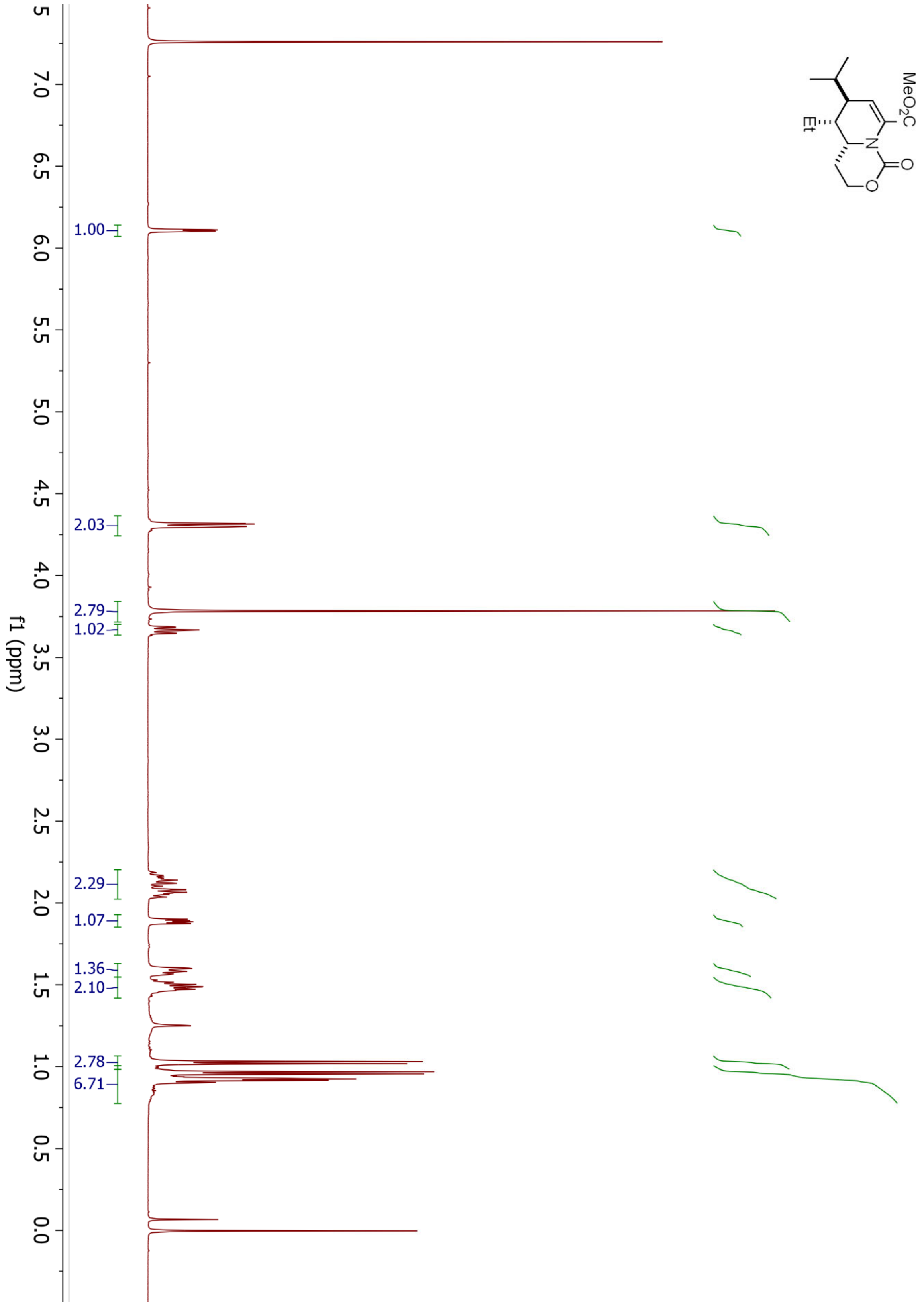

Supplementary Figure 71. ^{13}C NMR (126 MHz, $CDCl_3$) for compound **4cg**

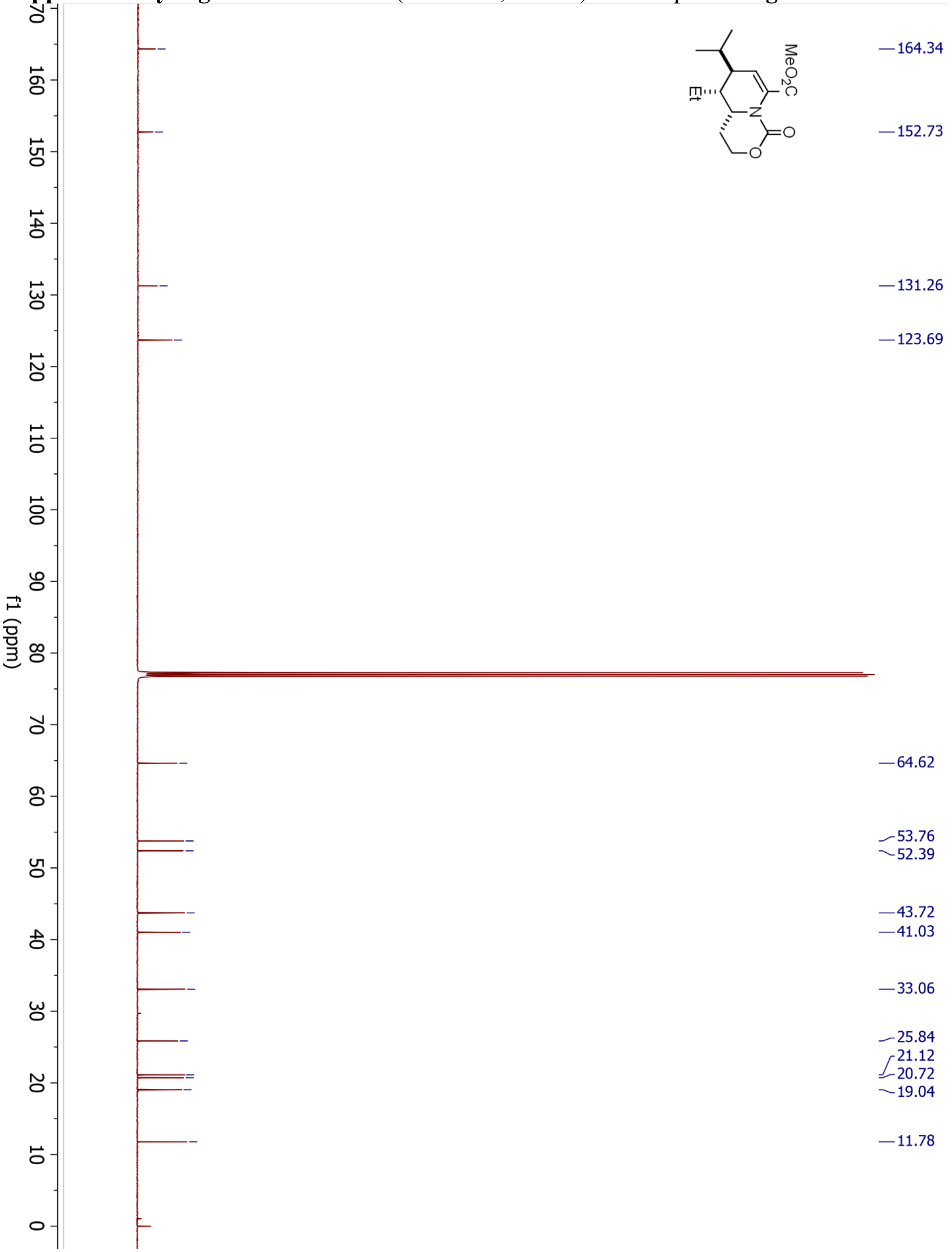

Supplementary Figure 72. ^{1}H NMR (500 MHz, CDCl$_3$) for Compound **4ch**

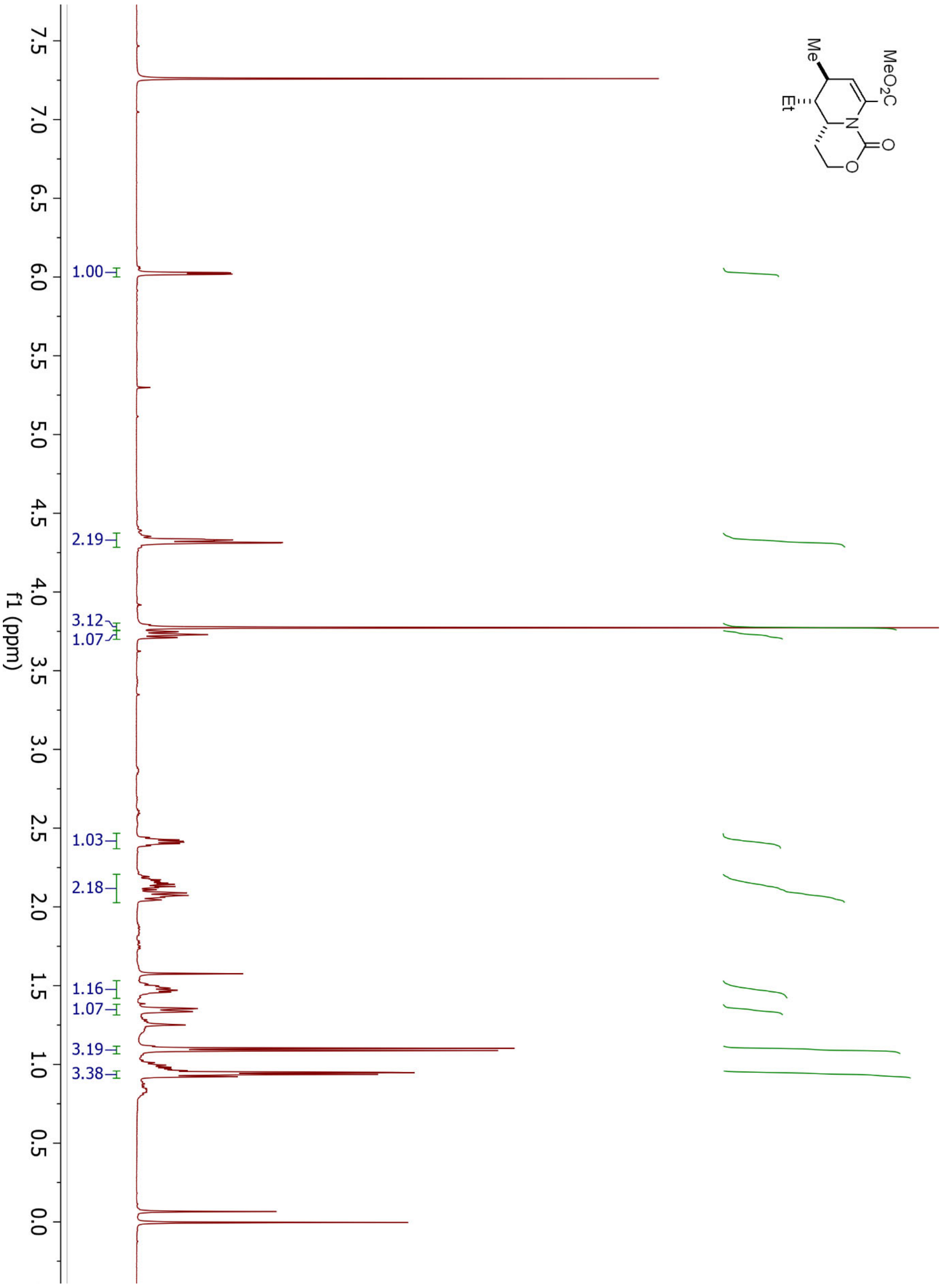

Supplementary Figure 73. ^{13}C NMR (126 MHz, $CDCl_3$) for compound **4ch**

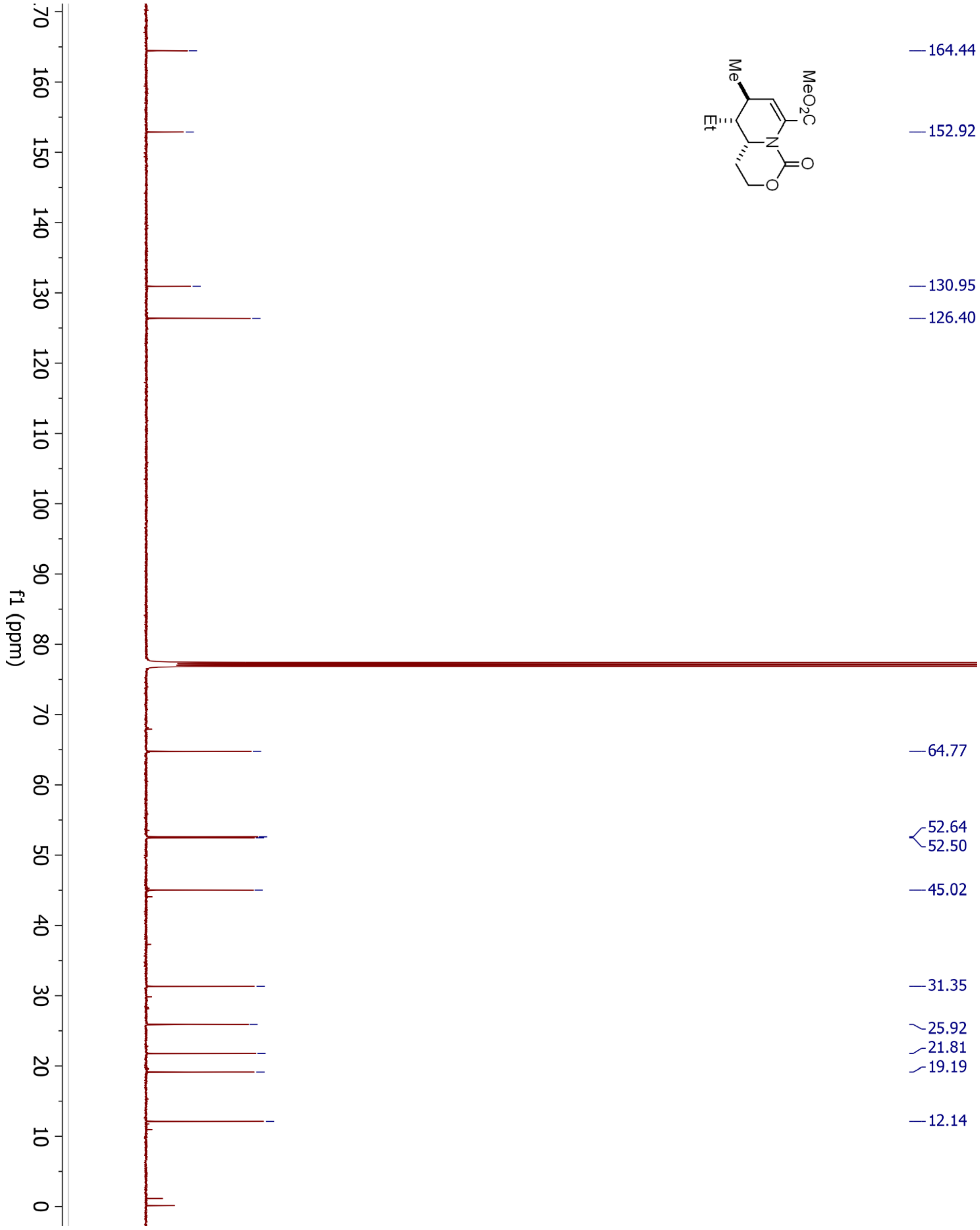

Supplementary Figure 74. ^{1}H NMR (500 MHz, $CDCl_3$) for compound **4ci**

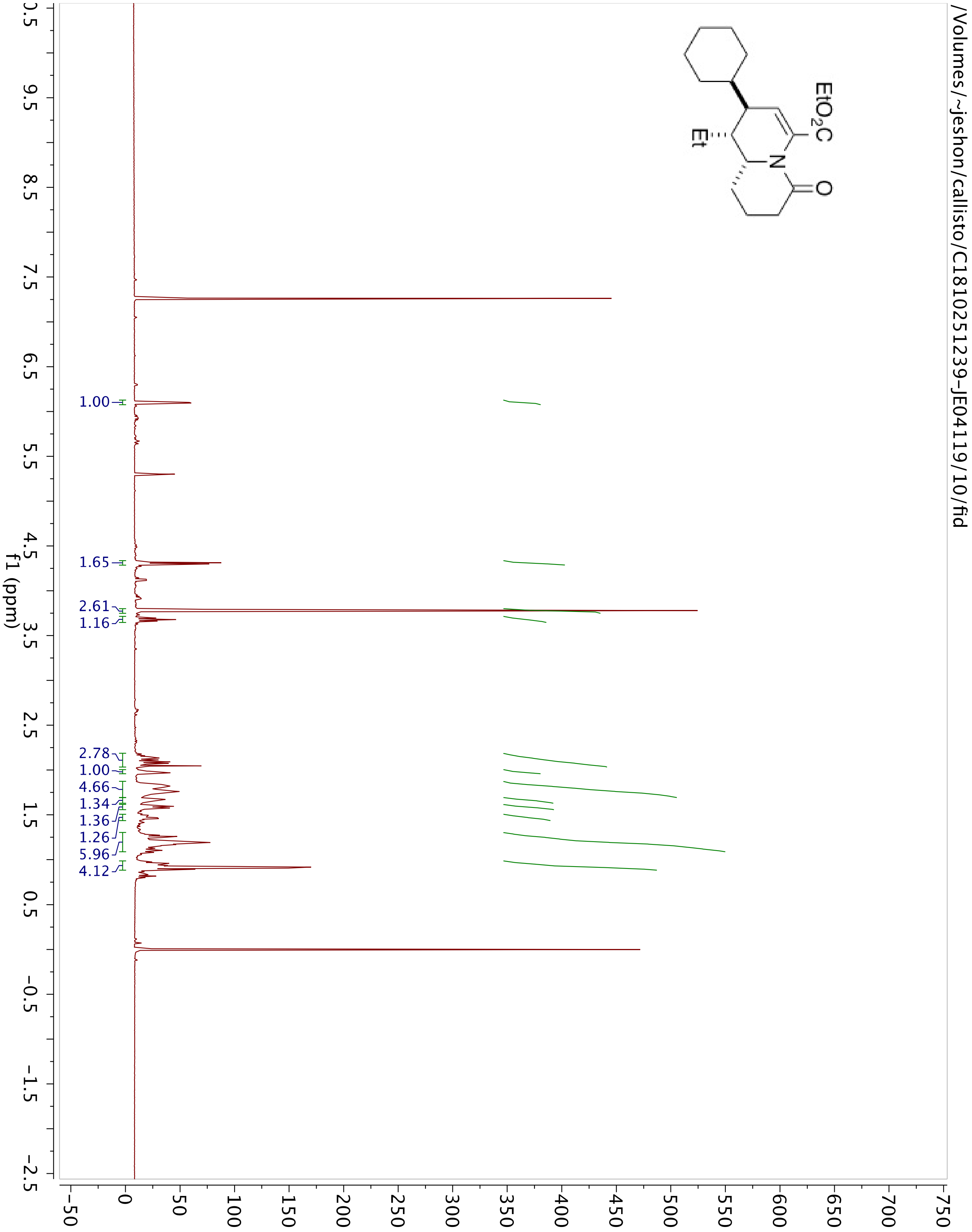

Supplementary Figure 75. ^{13}C NMR (126 MHz, $CDCl_3$) for compound **4ci**

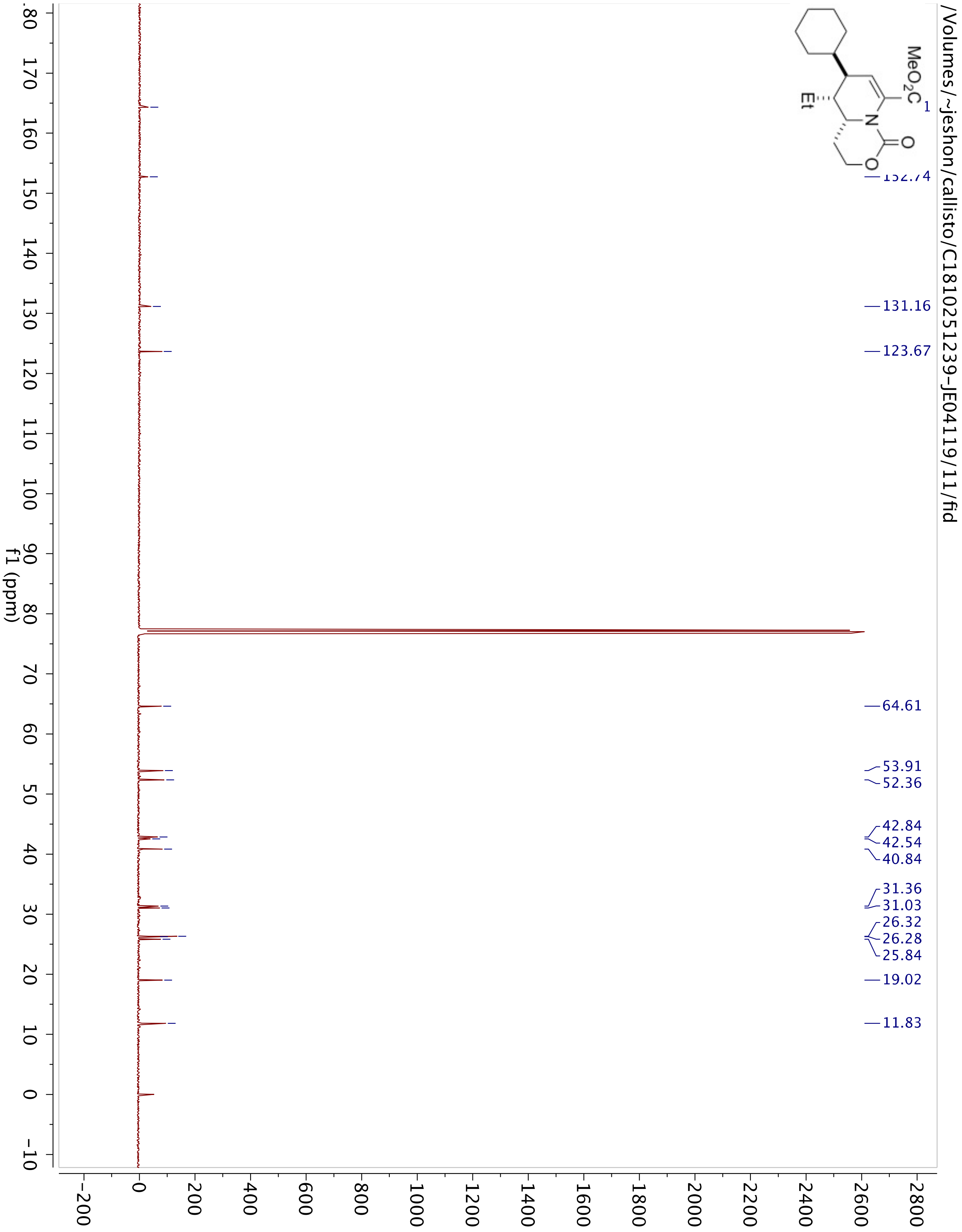

Supplementary Figure 76. ^{1}H NMR (500 MHz, $CDCl_3$) for compound **4cj**

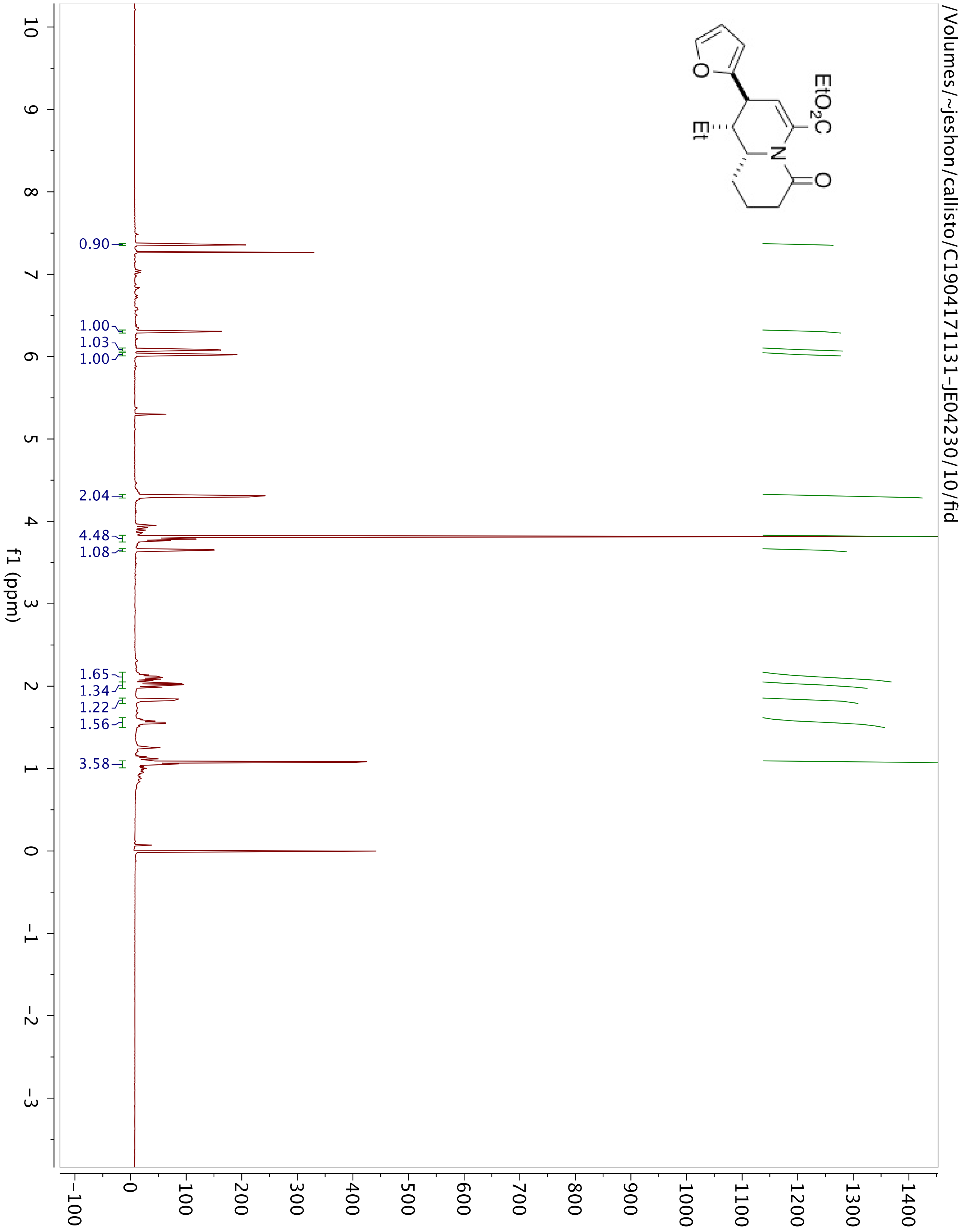

Supplementary Figure 77. ^{13}C NMR (126 MHz, $CDCl_3$) for compound **4cj**

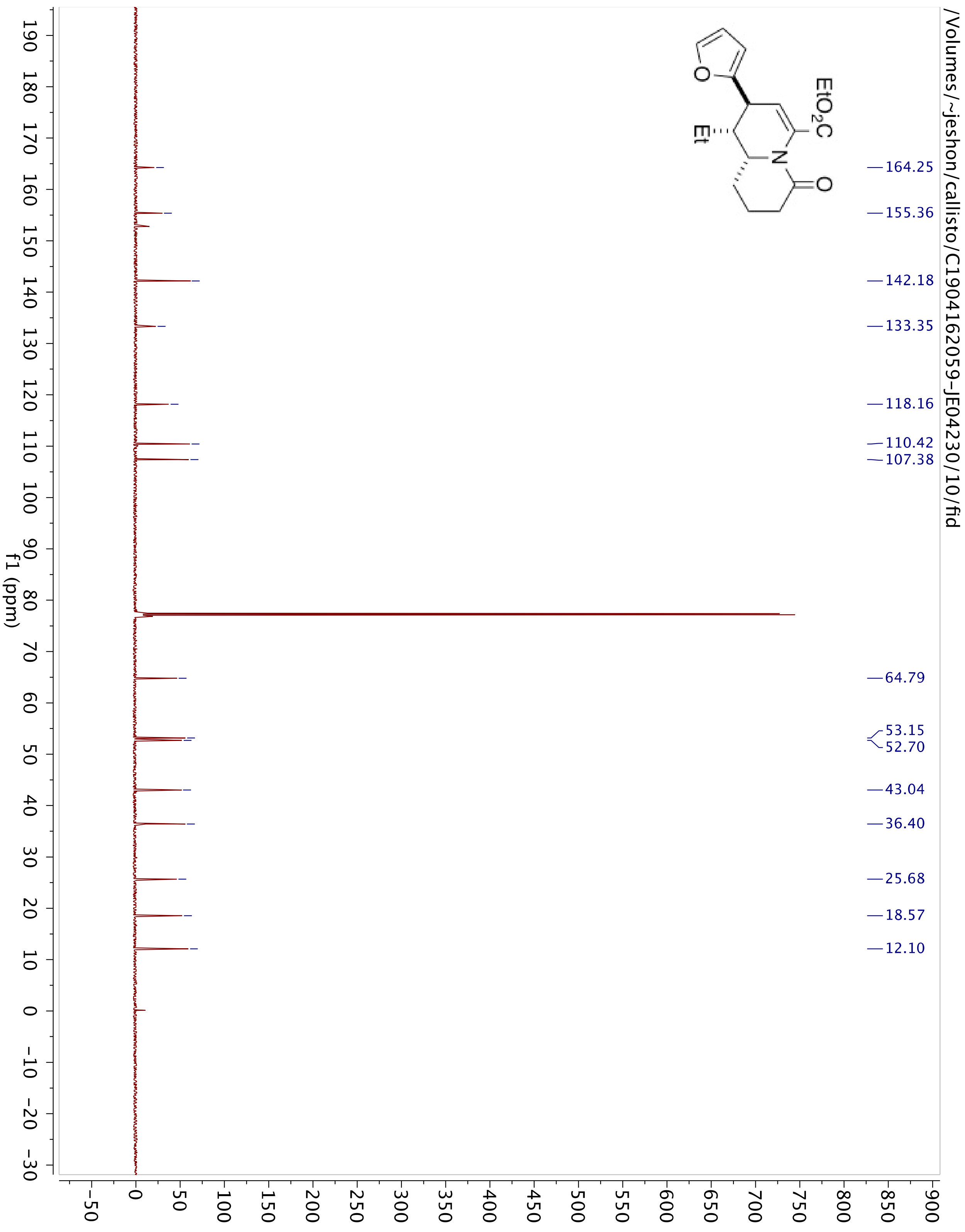

Supplementary Figure 78. ^{1}H NMR (500 MHz, $CDCl_3$) for compound **4ck**

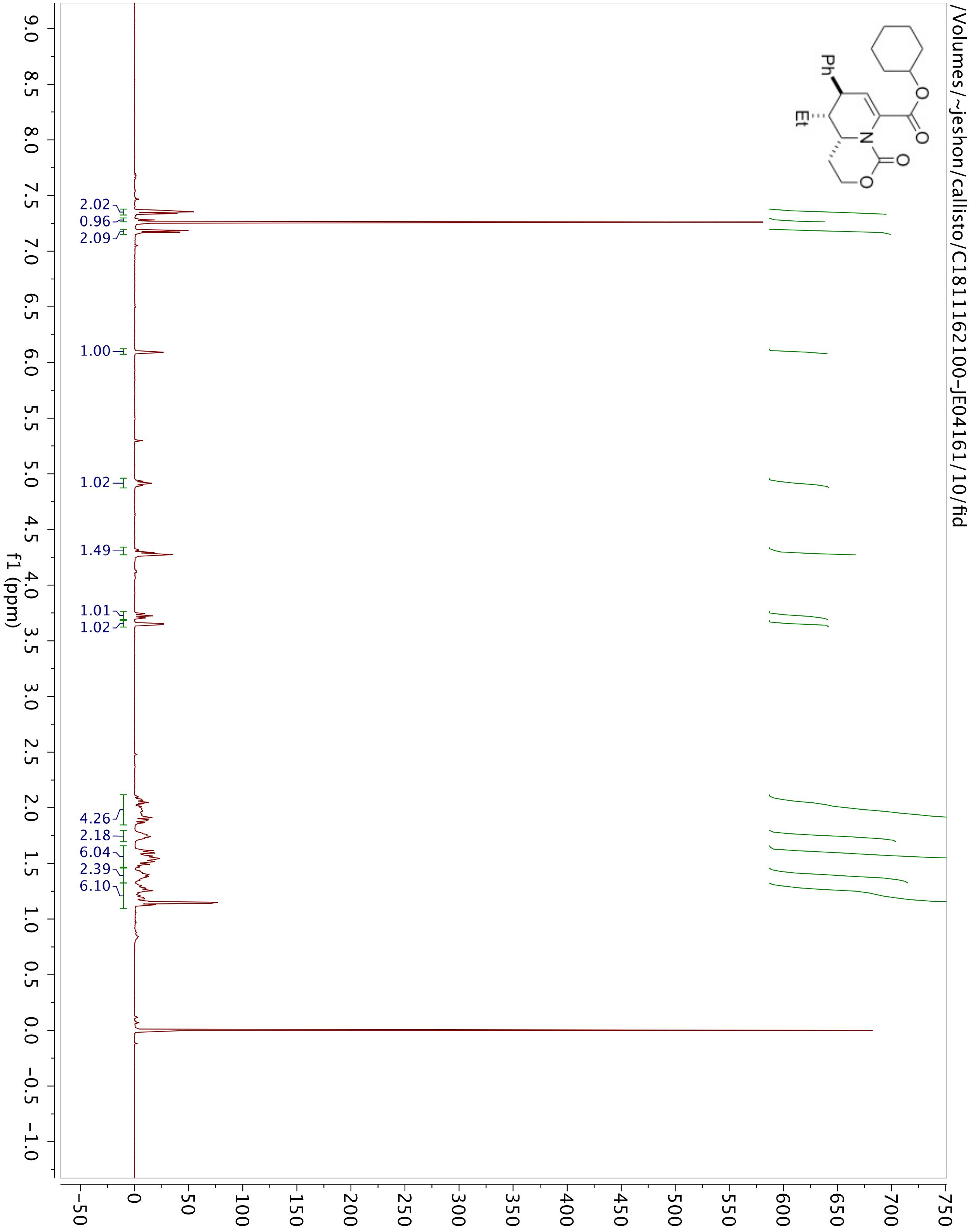

Supplementary Figure 79. ^{13}C NMR (126 MHz, $CDCl_3$) for compound **4ck**

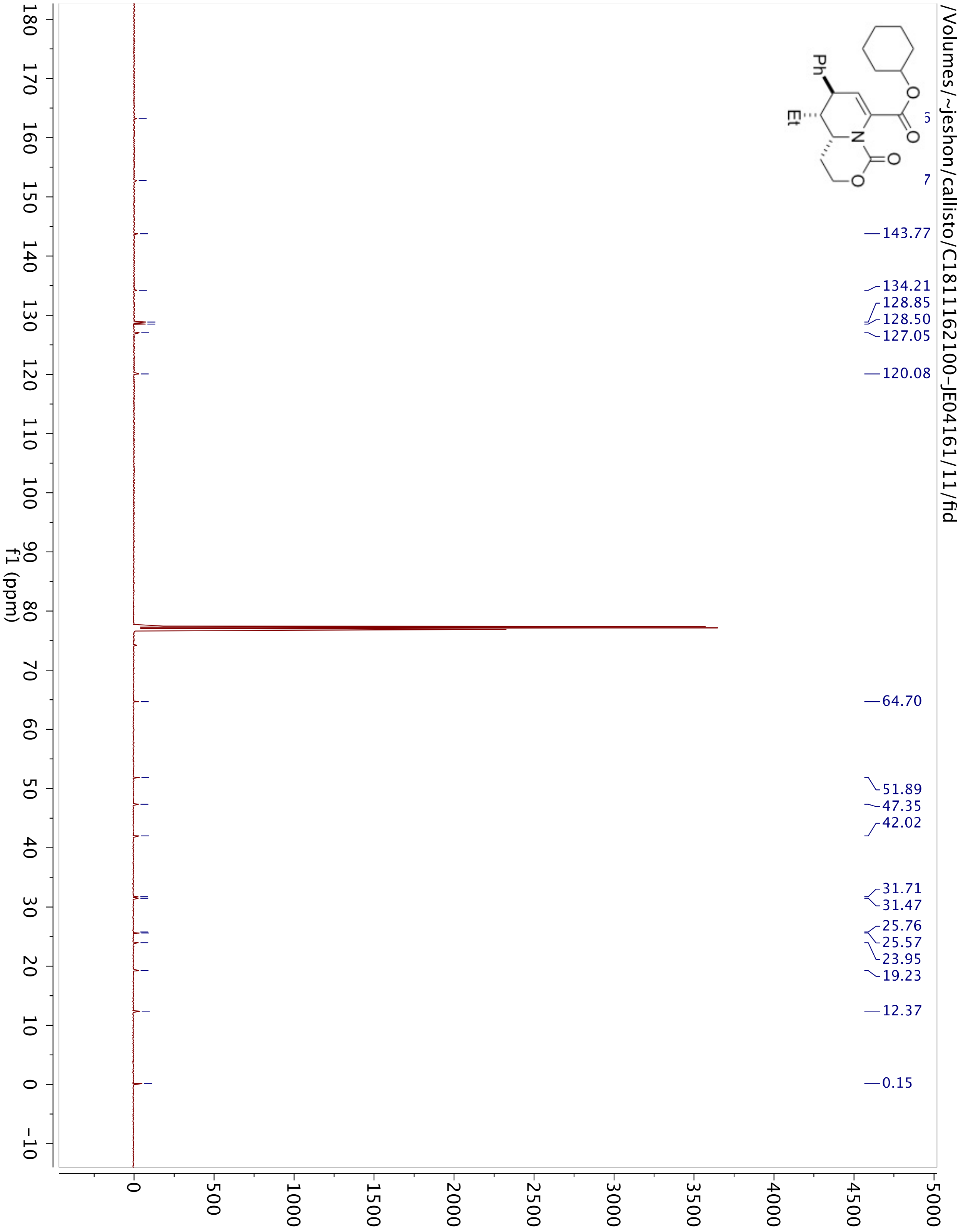

Supplementary Figure 80. ^{1}H NMR (500 MHz, $CDCl_3$) for compound **6**

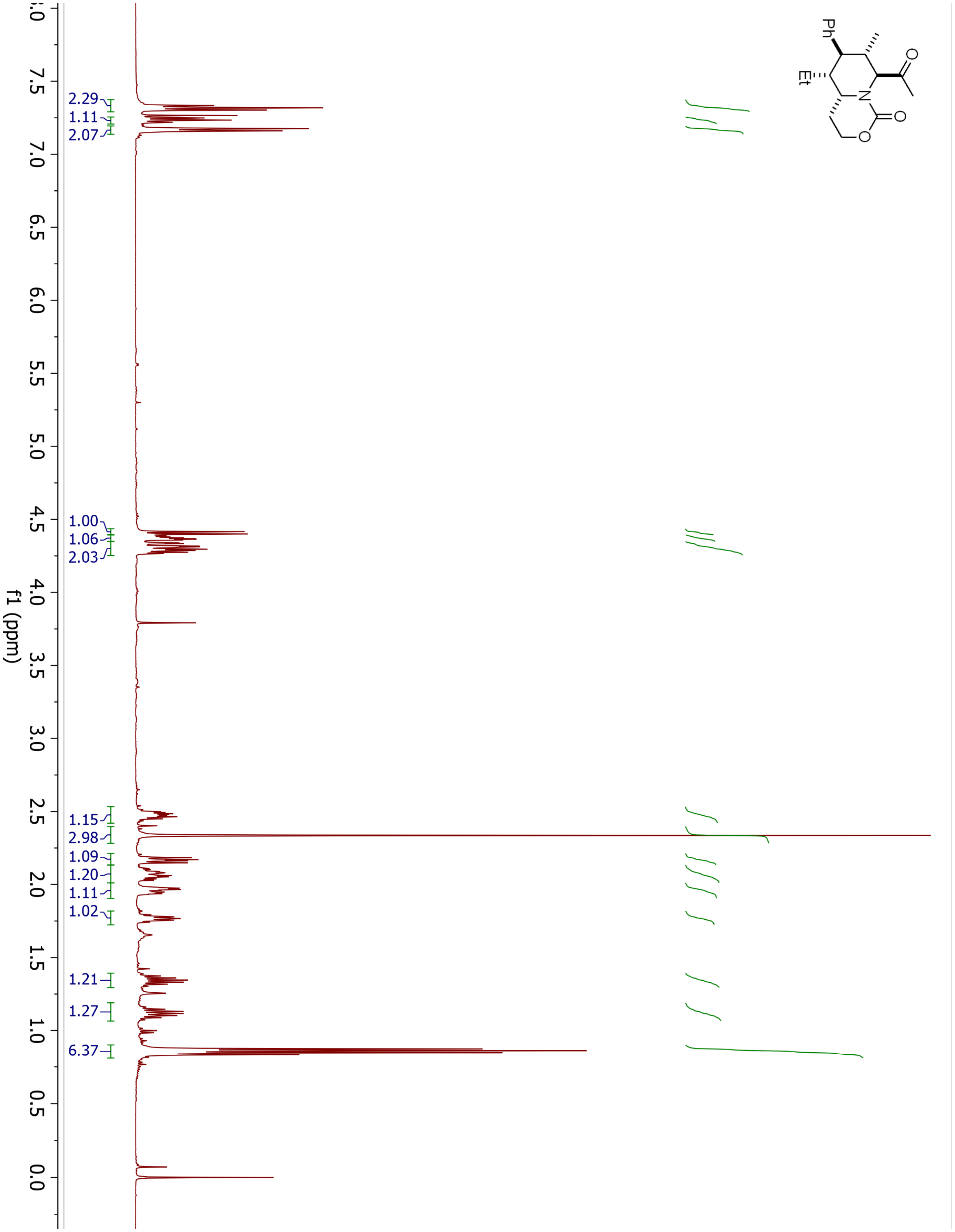

Supplementary Figure 81. ^{13}C NMR (126 MHz, $CDCl_3$) for compound **6**

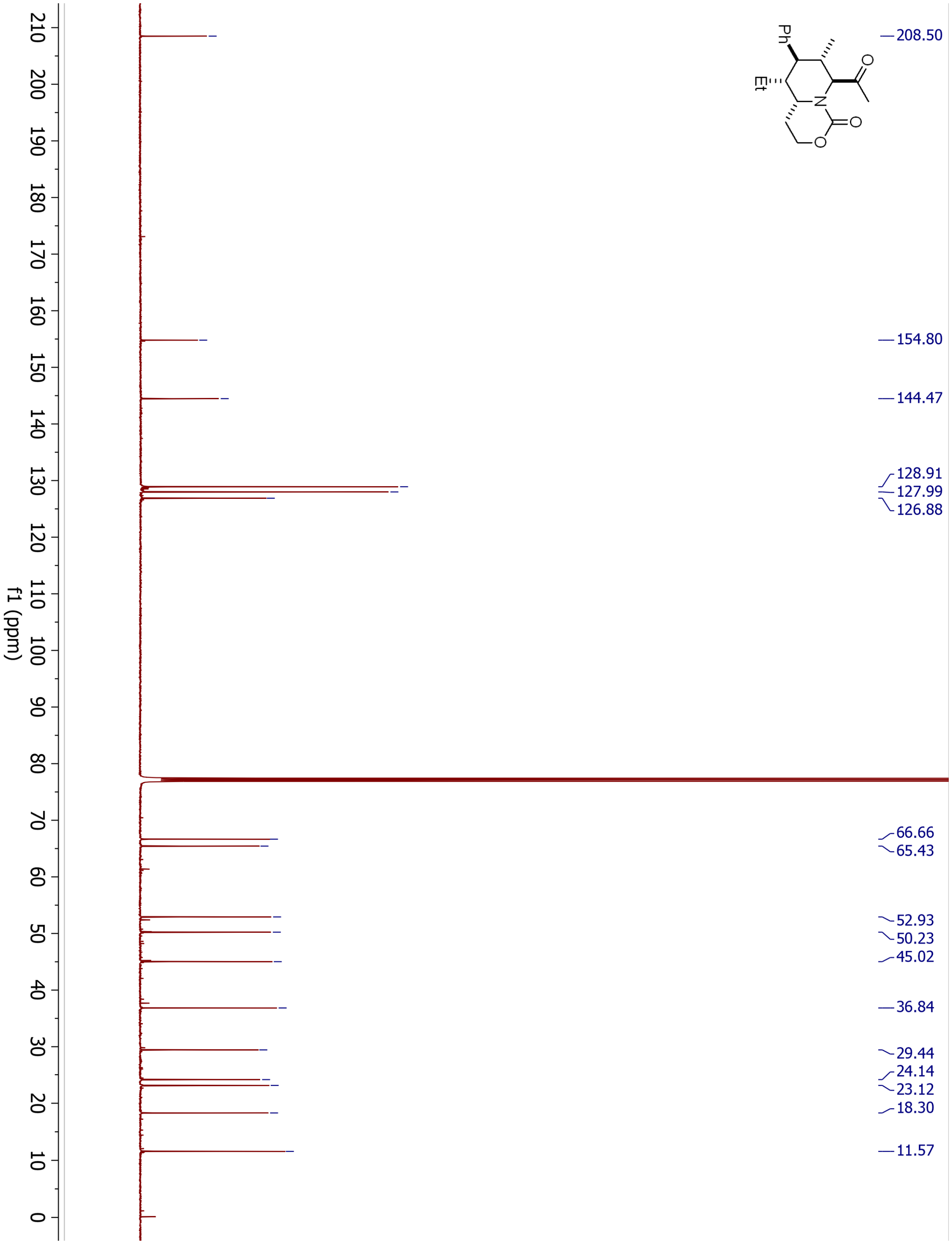

Supplementary Figure 82. ^{1}H NMR (500 MHz, $CDCl_3$) for compound **7**

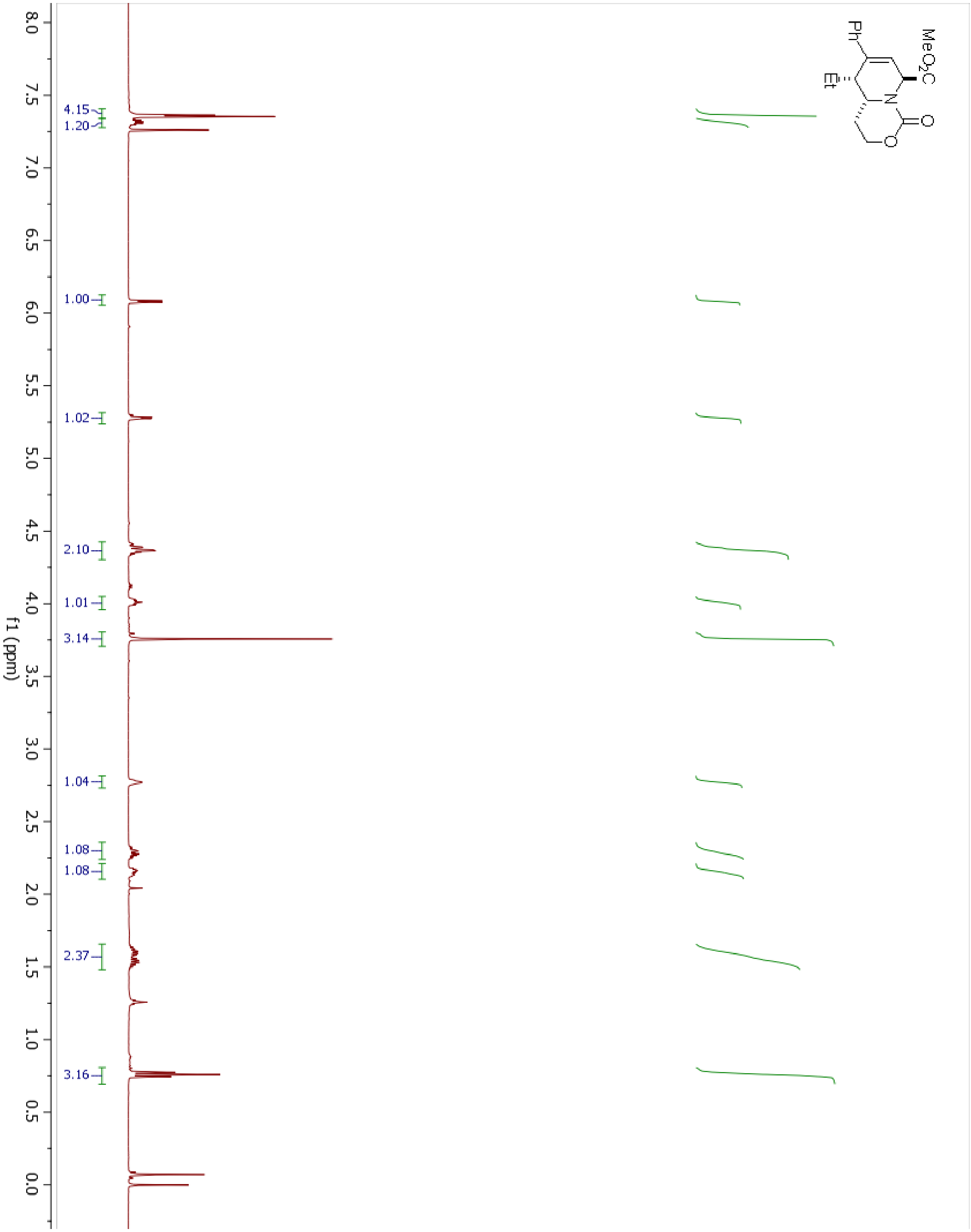

Supplementary Figure 83. ^{13}C NMR (126 MHz, $CDCl_3$) for compound **7**

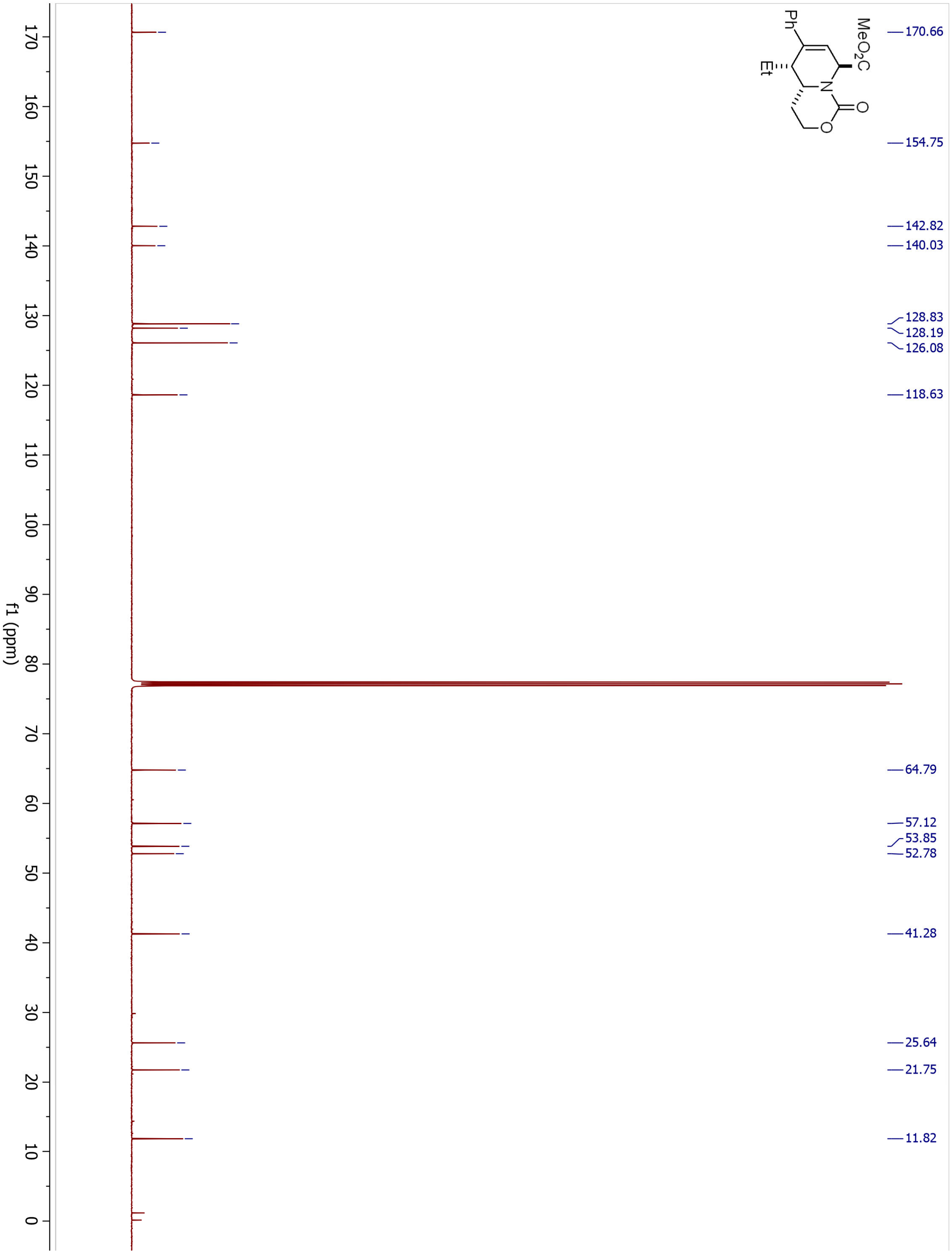

Supplementary Figure 84. ^{1}H NMR (500 MHz, $CDCl_3$) for compound **8**

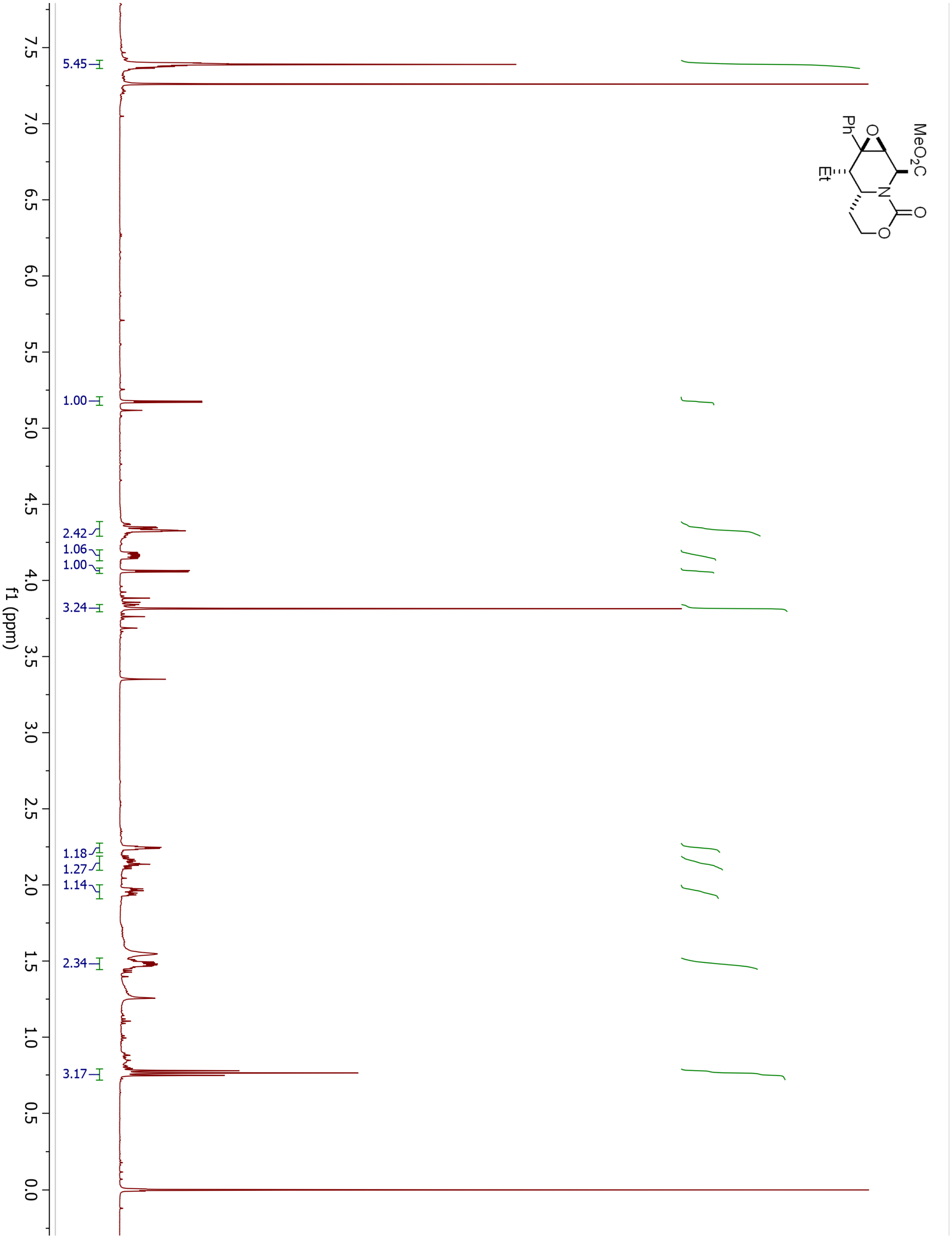

Supplementary Figure 85. ^{13}C NMR (126 MHz, $CDCl_3$) for compound **8**

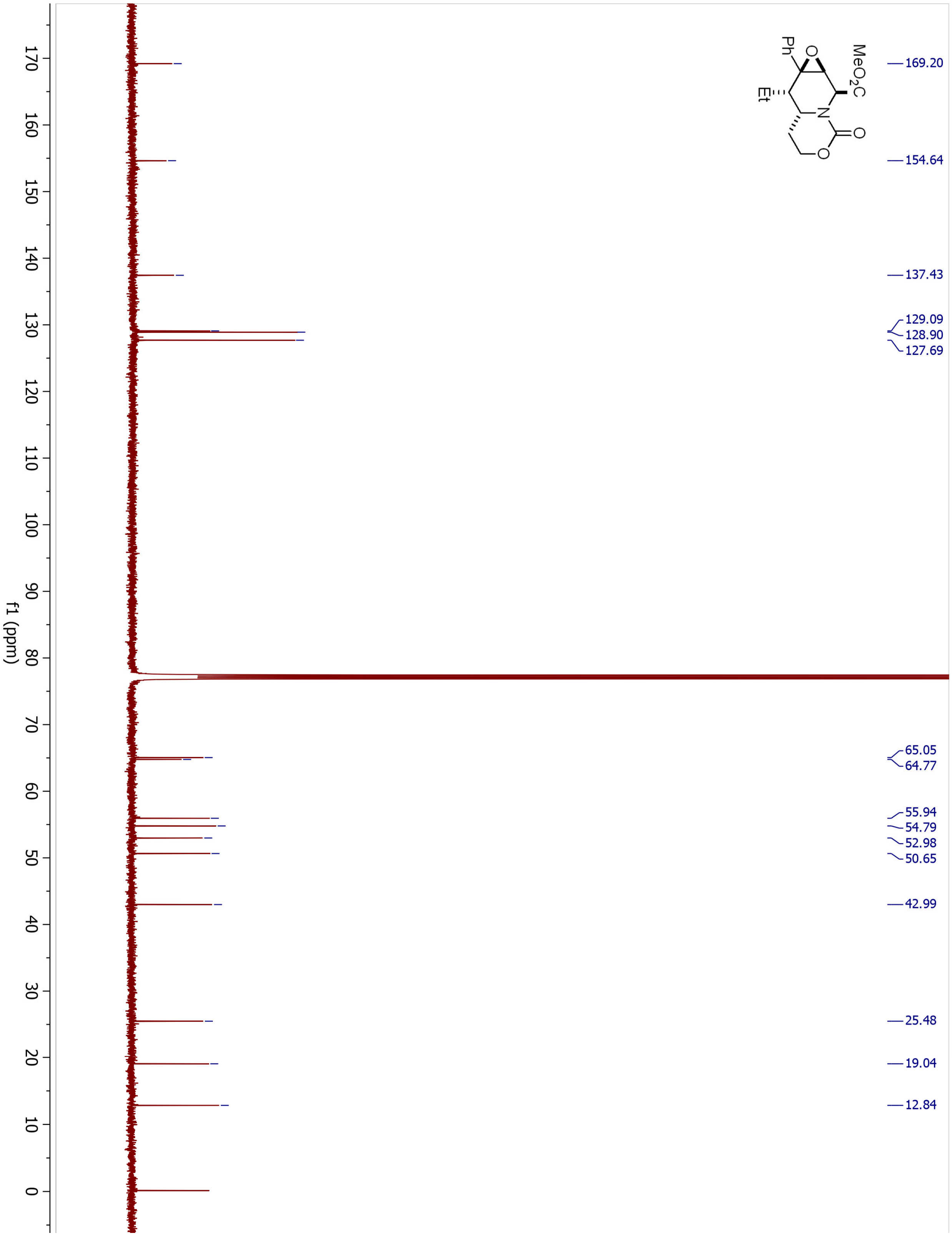

Supplementary Figure 86. ^{1}H NMR (500 MHz, $CDCl_3$) for compound **10**

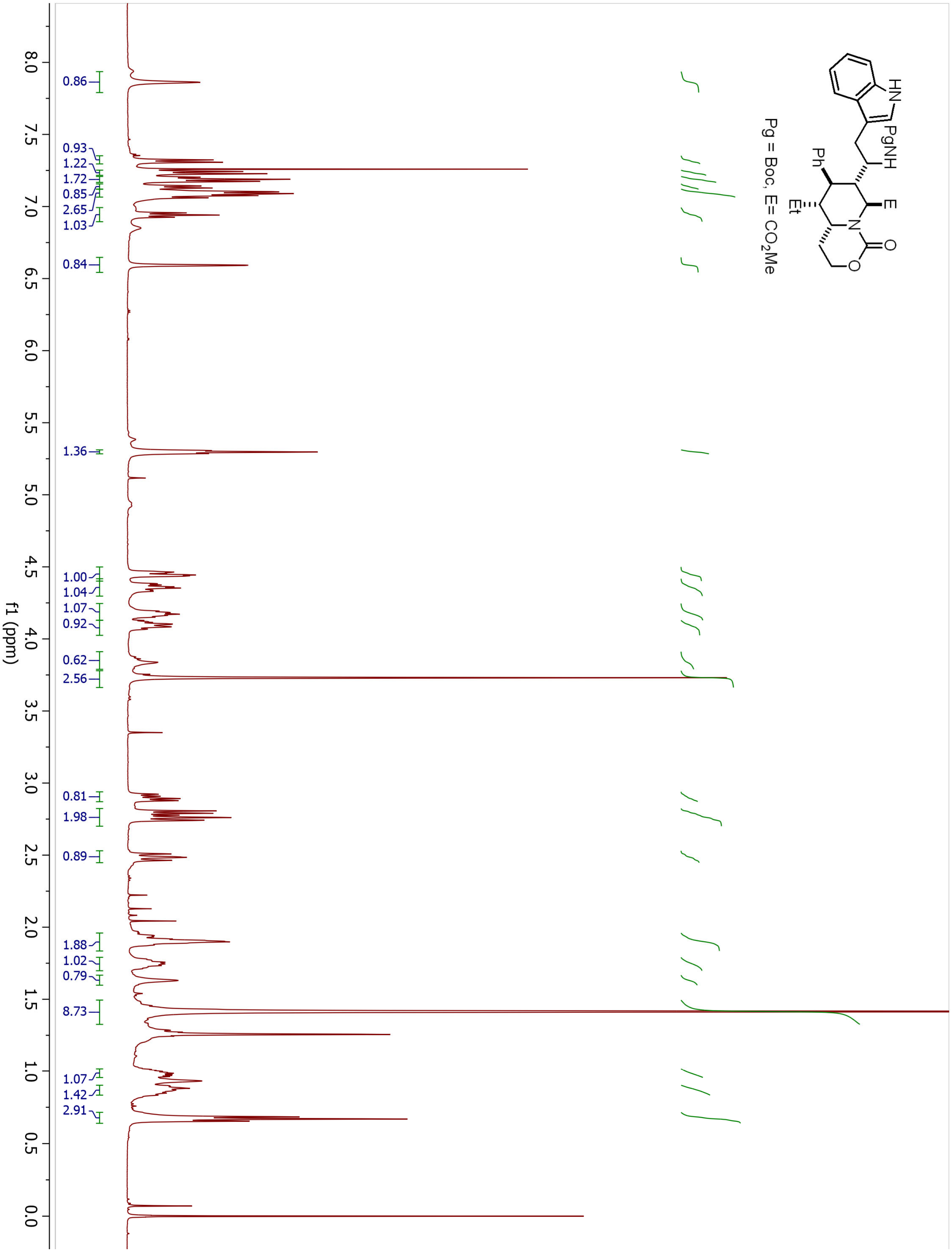

Supplementary Figure 87. ^{13}C NMR (126 MHz, $CDCl_3$) for compound **10**

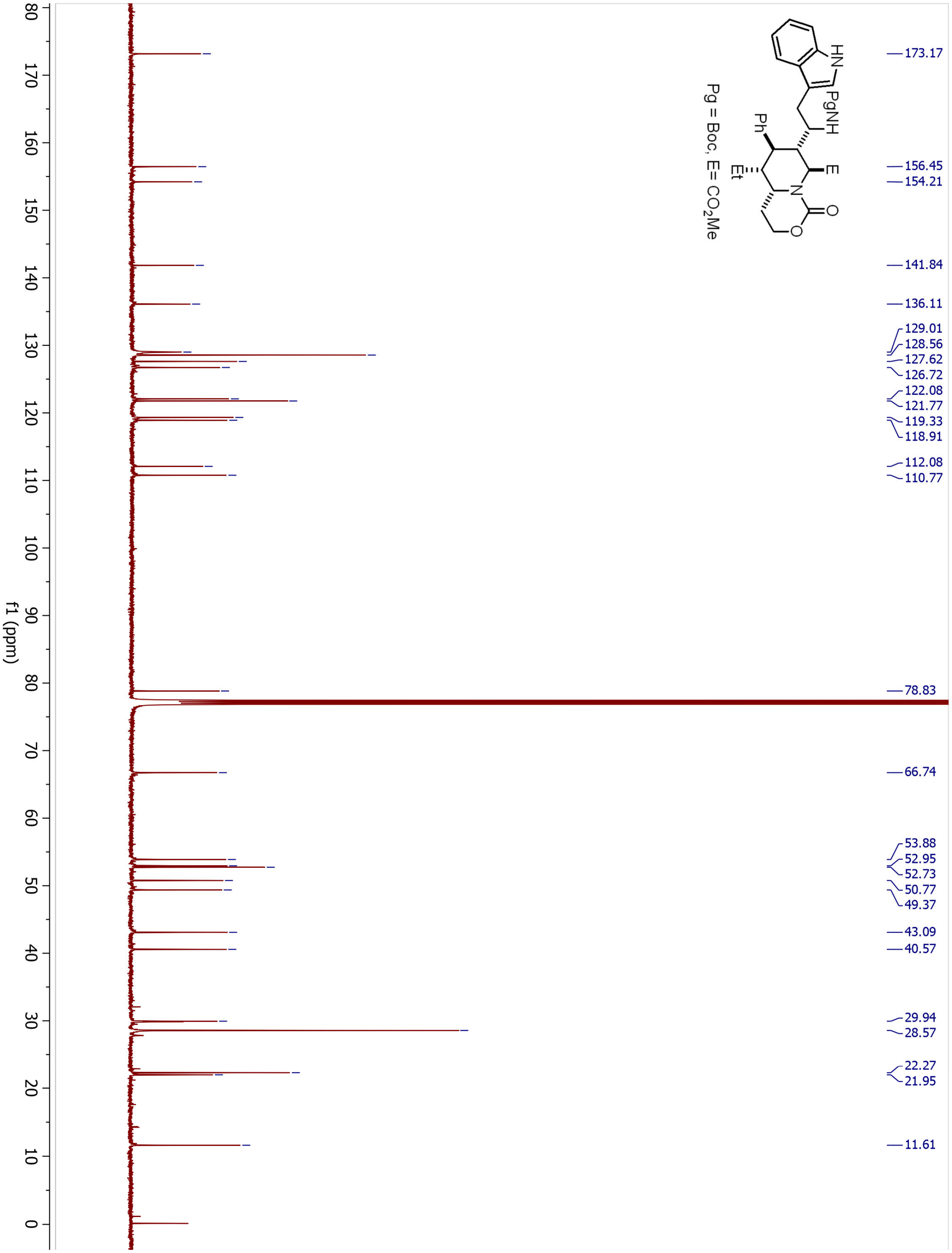

Supplementary Figure 88. ^{1}H NMR (500 MHz, $CDCl_3$) for compound **11a**

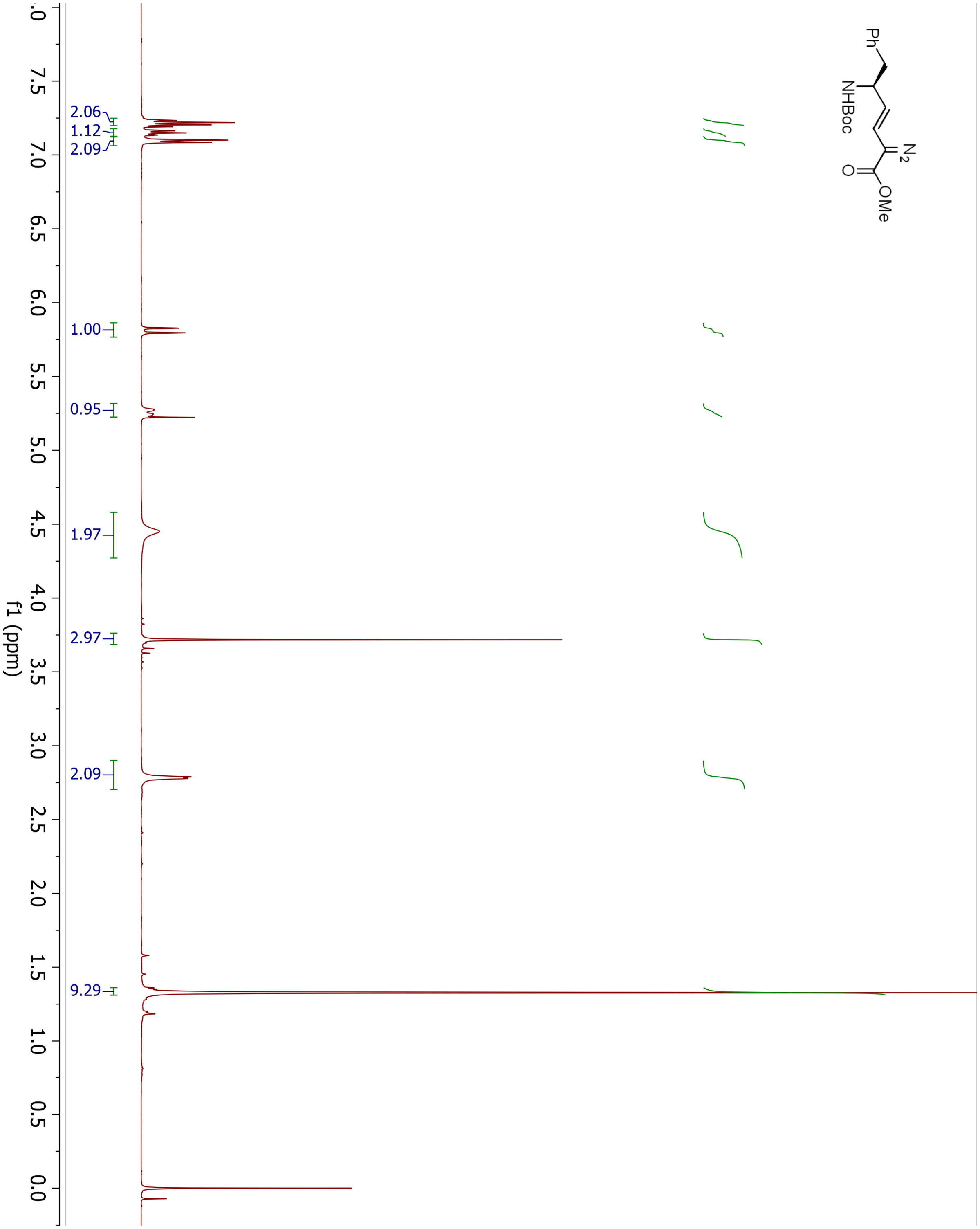

Supplementary Figure 89. ^{13}C NMR (126 MHz, $CDCl_3$) for compound **11a**

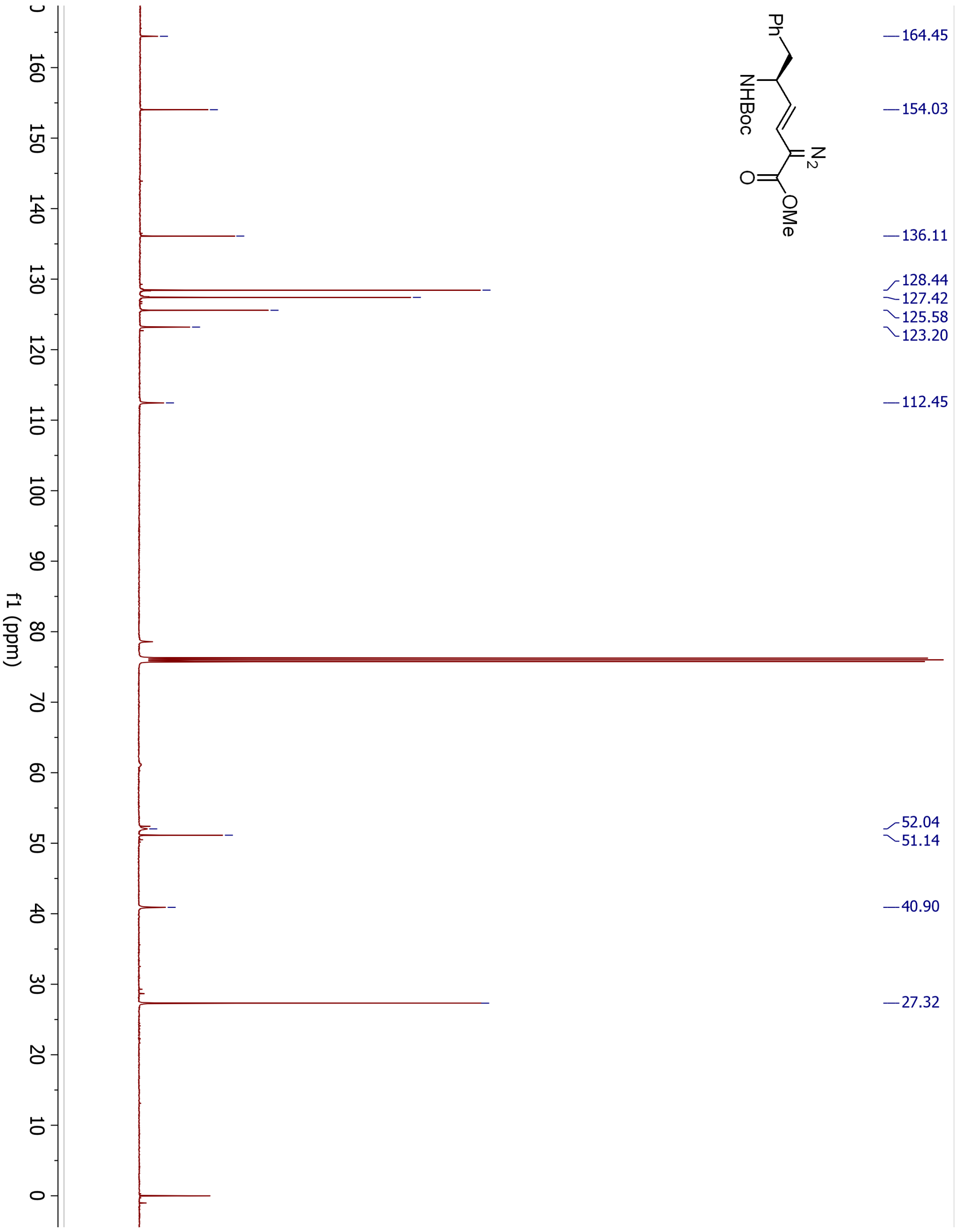

Supplementary Figure 90. ^{1}H NMR (500 MHz, $CDCl_3$) for compound **12a**

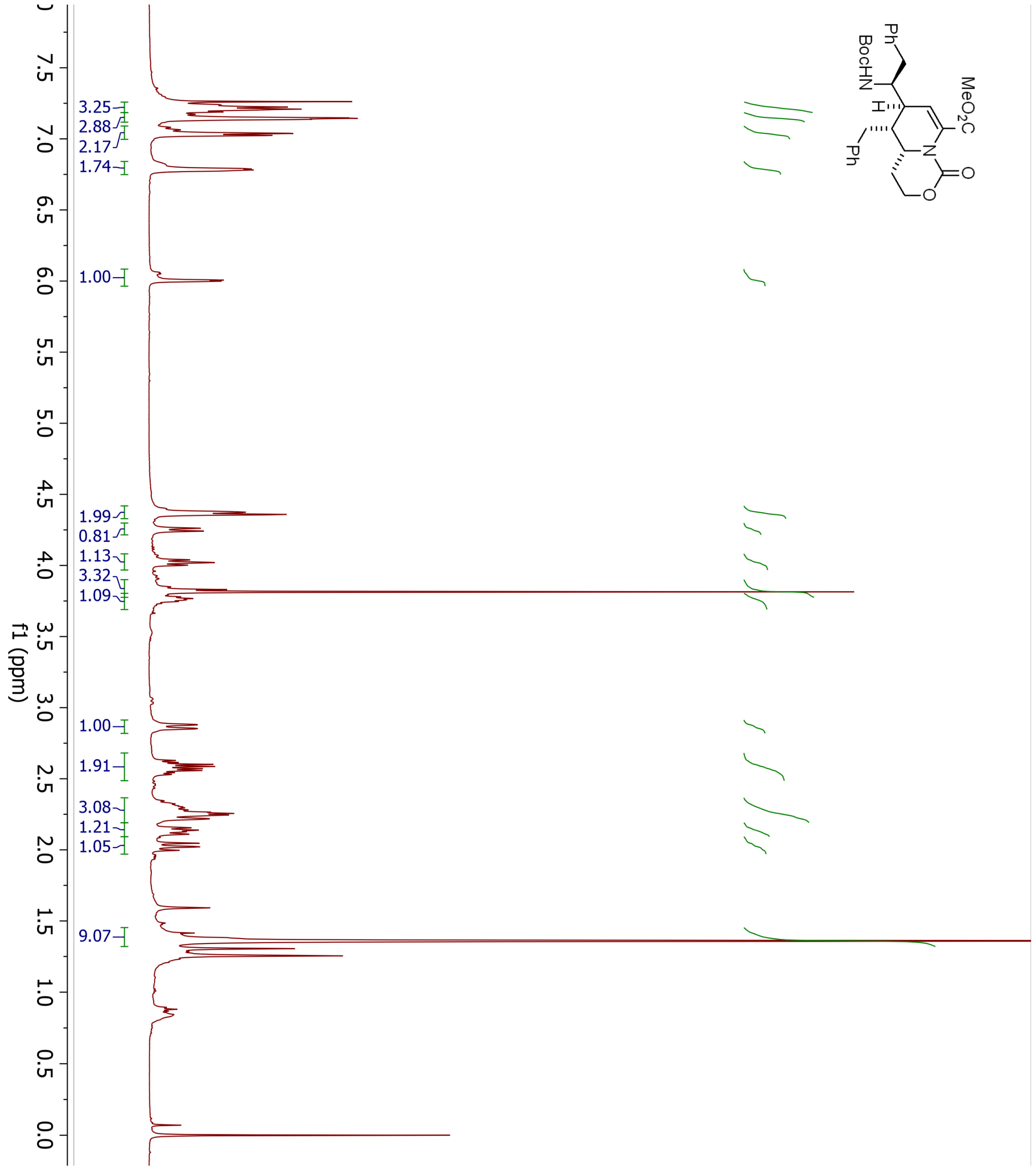

Supplementary Figure 91. ^{13}C NMR (126 MHz, $CDCl_3$) for compound **12a**

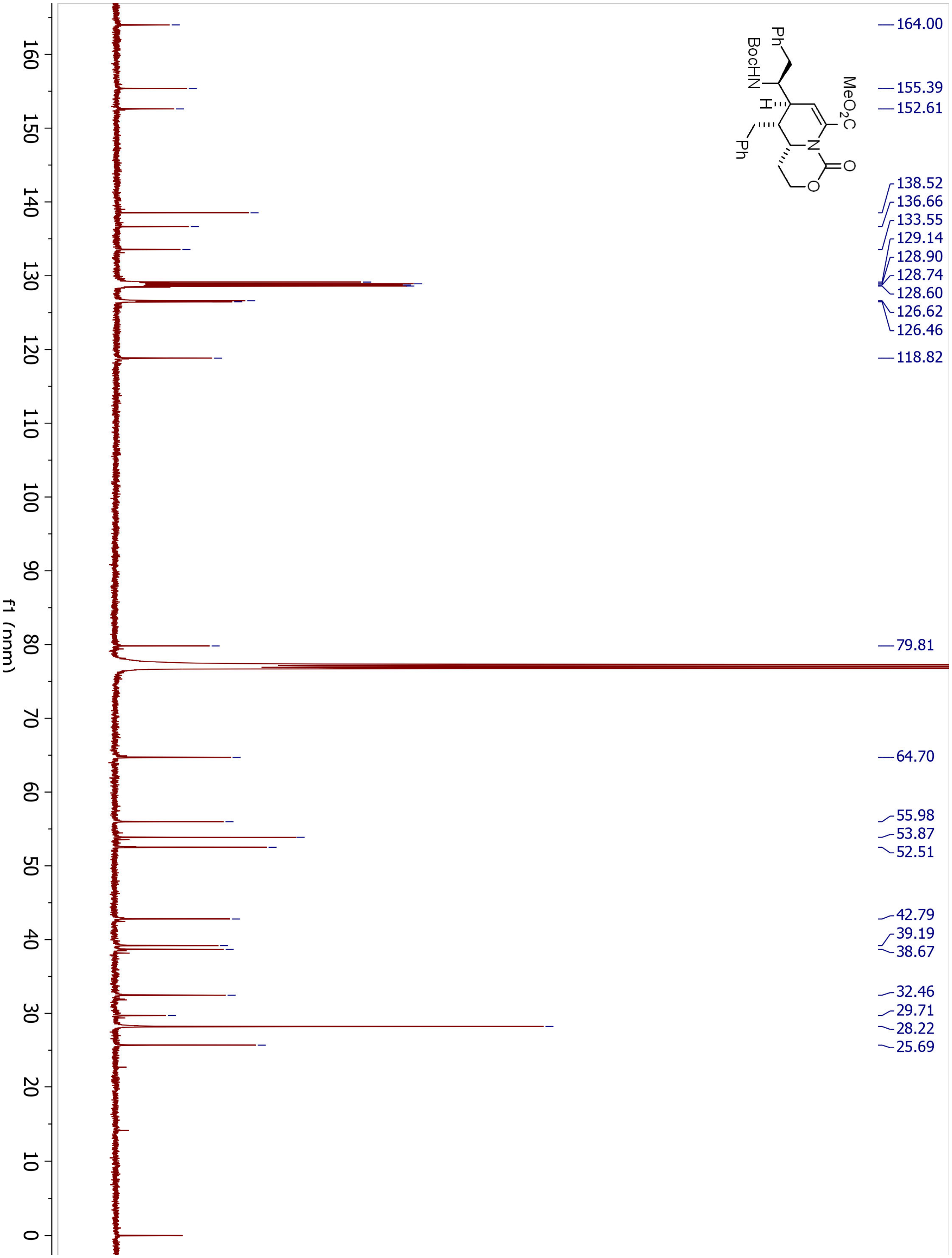

Supplementary Figure 92. ^{1}H NMR (500 MHz, C_6D_6) for compound **S1**

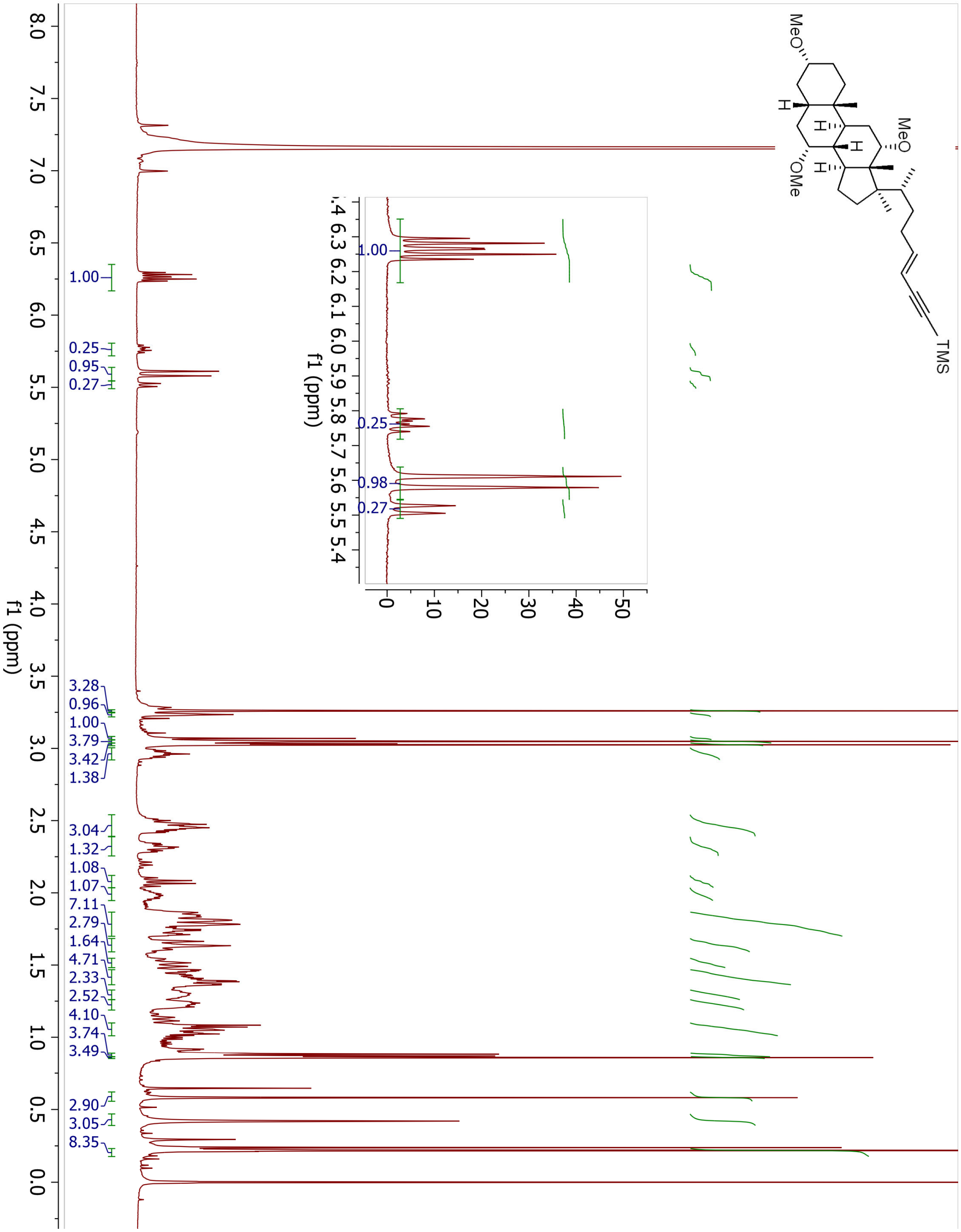

Supplementary Figure 93. ^{13}C NMR (126 MHz, C_6D_6) for compound **S1**

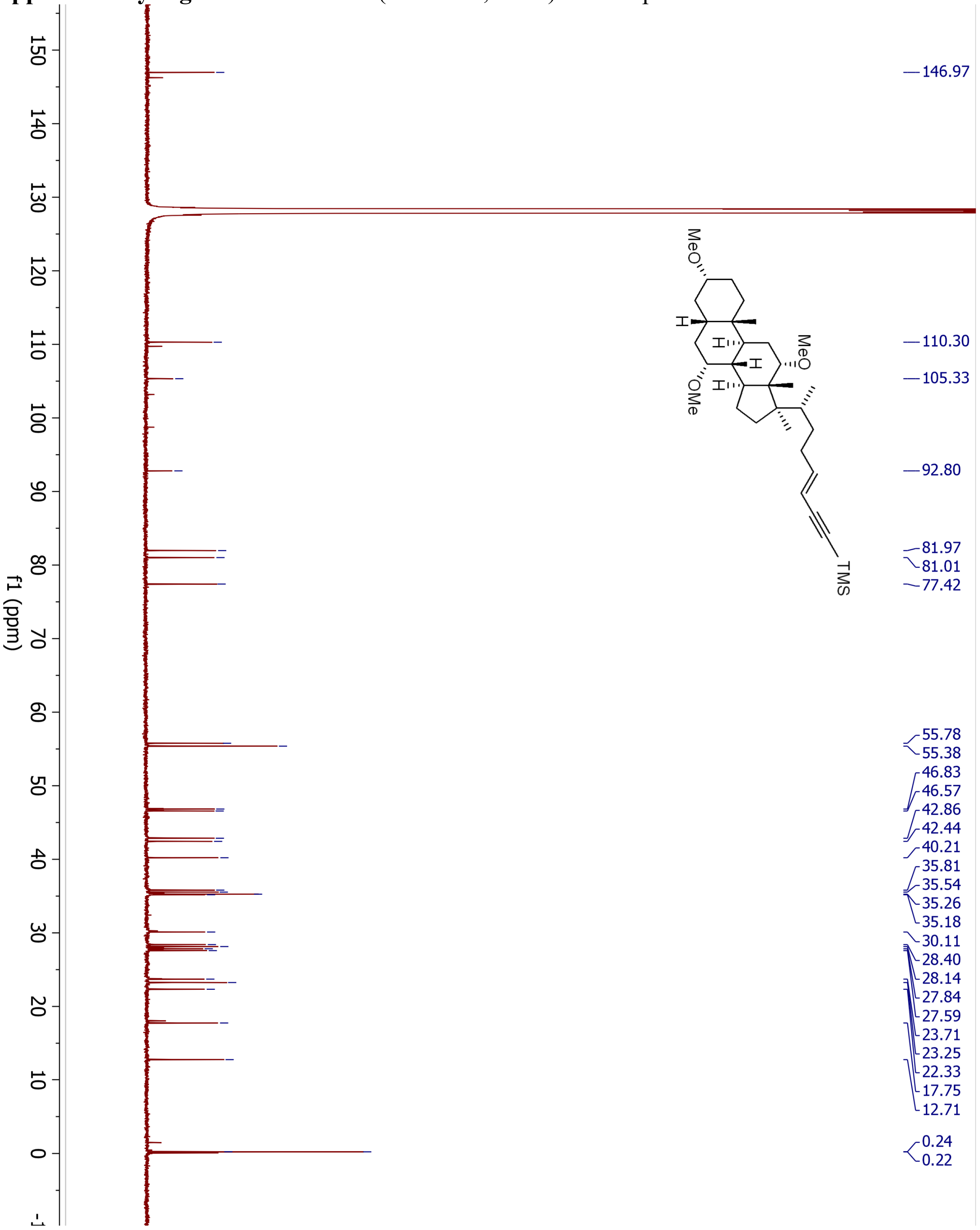

Supplementary Figure 94. ^{1}H NMR (500 MHz, C_6D_6) for compound **S2**

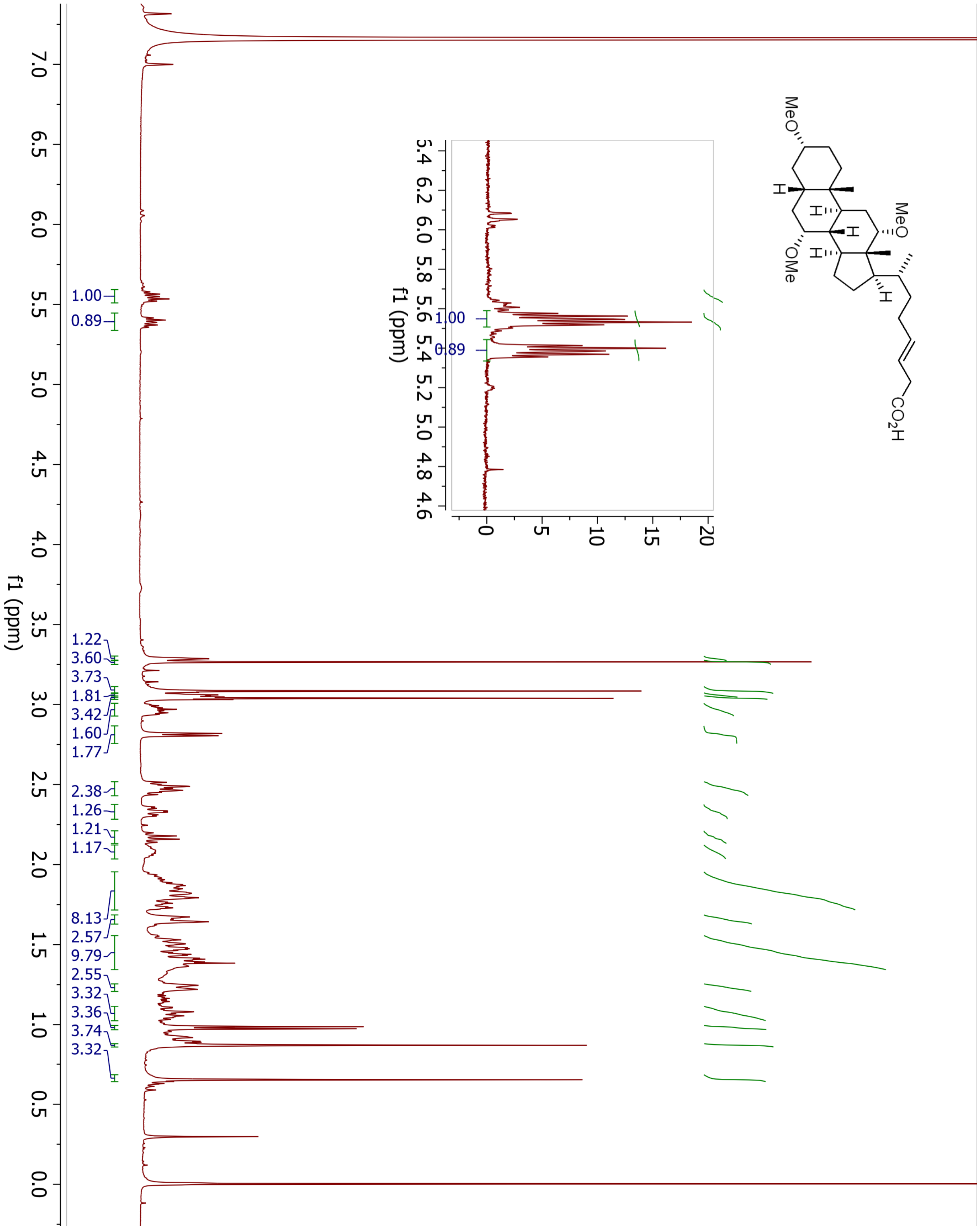

Supplementary Figure 95. ^{13}C NMR (126 MHz, C_6D_6) for compound **S2**

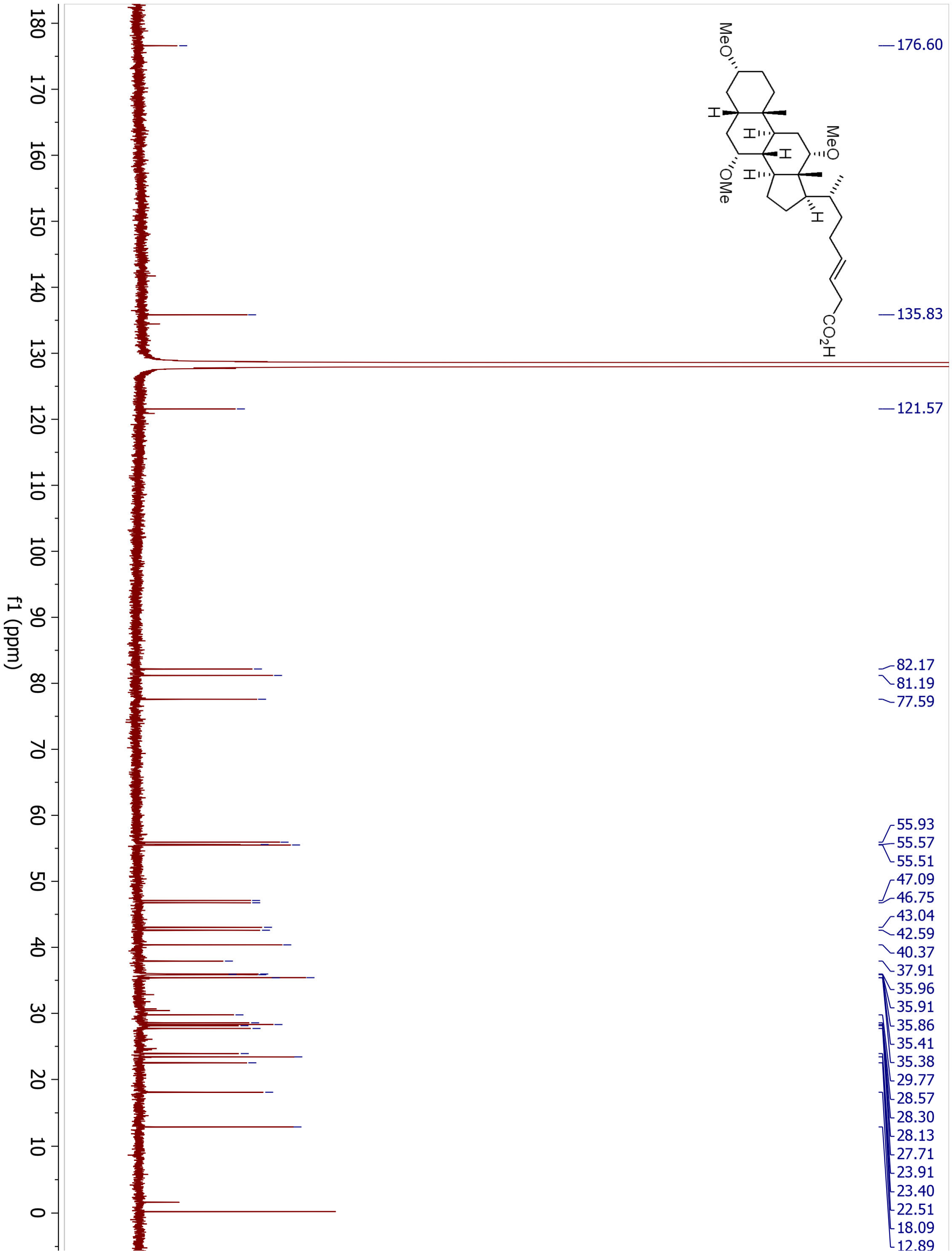

Supplementary Figure 96. ^{1}H NMR (500 MHz, C_6D_6) for compound **S3**

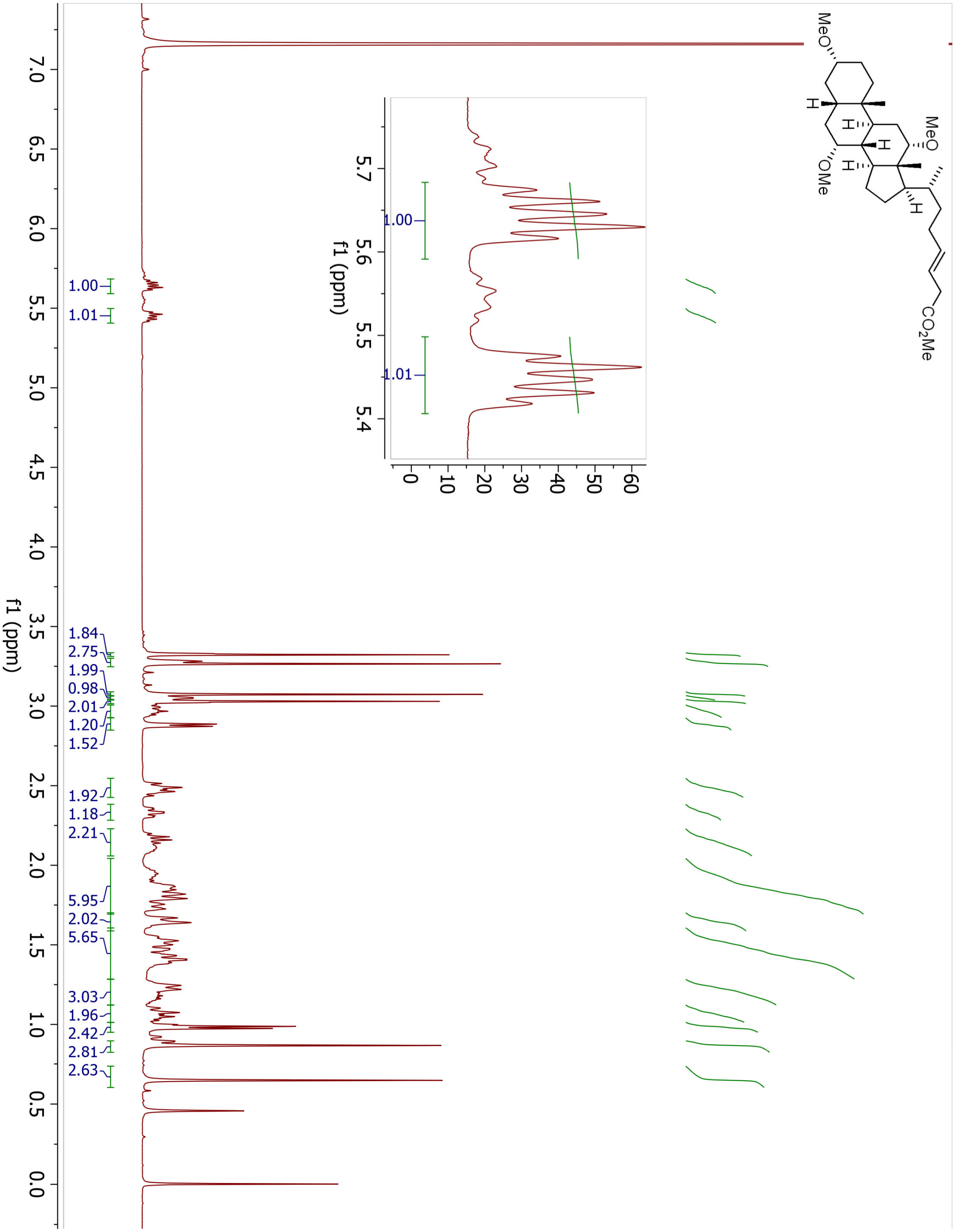

Supplementary Figure 97. ^{13}C NMR (126 MHz, C_6D_6) for compound **S3**

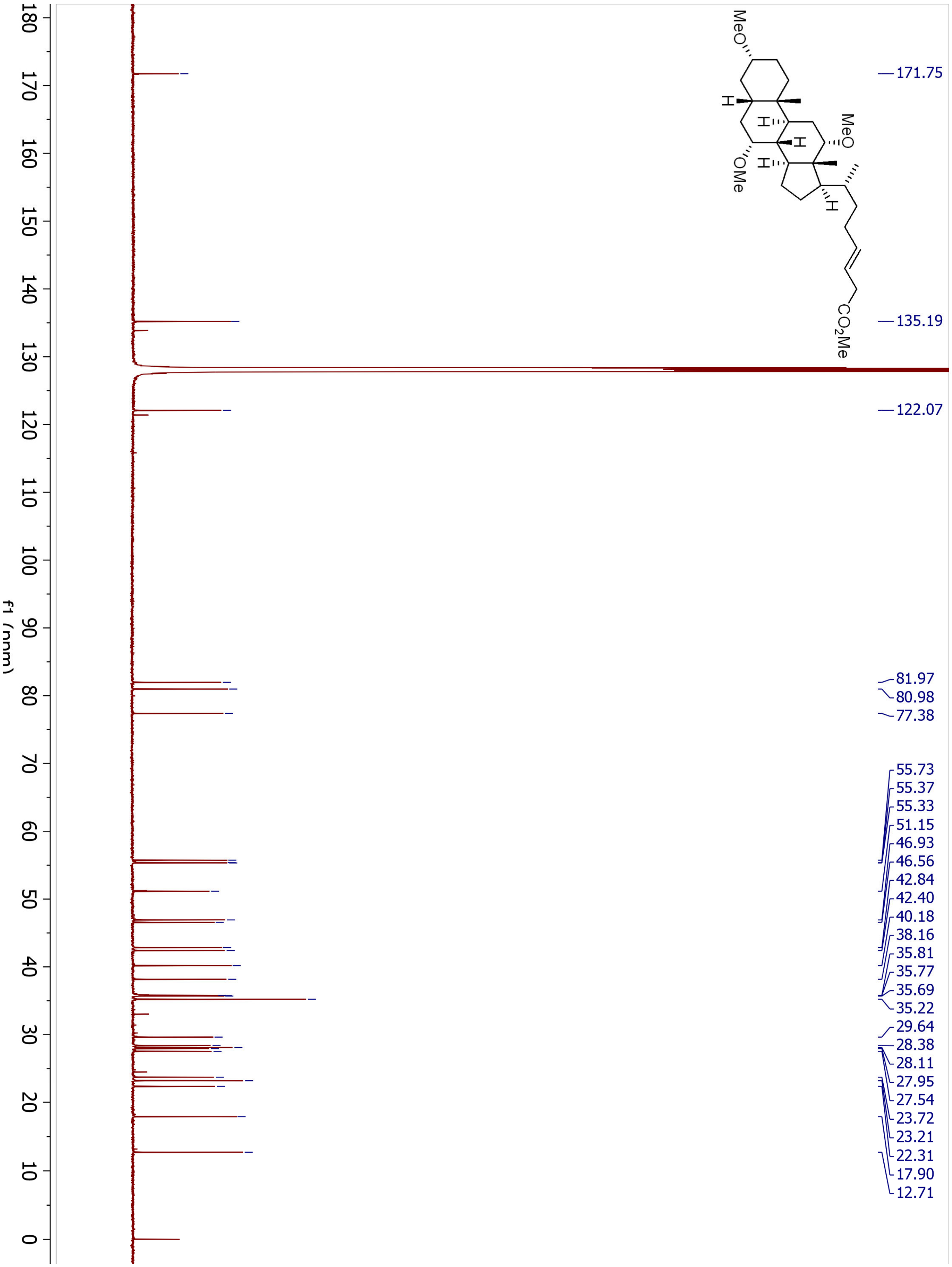

Supplementary Figure 98. ^{1}H NMR (500 MHz, C_6D_6) for compound **11b**

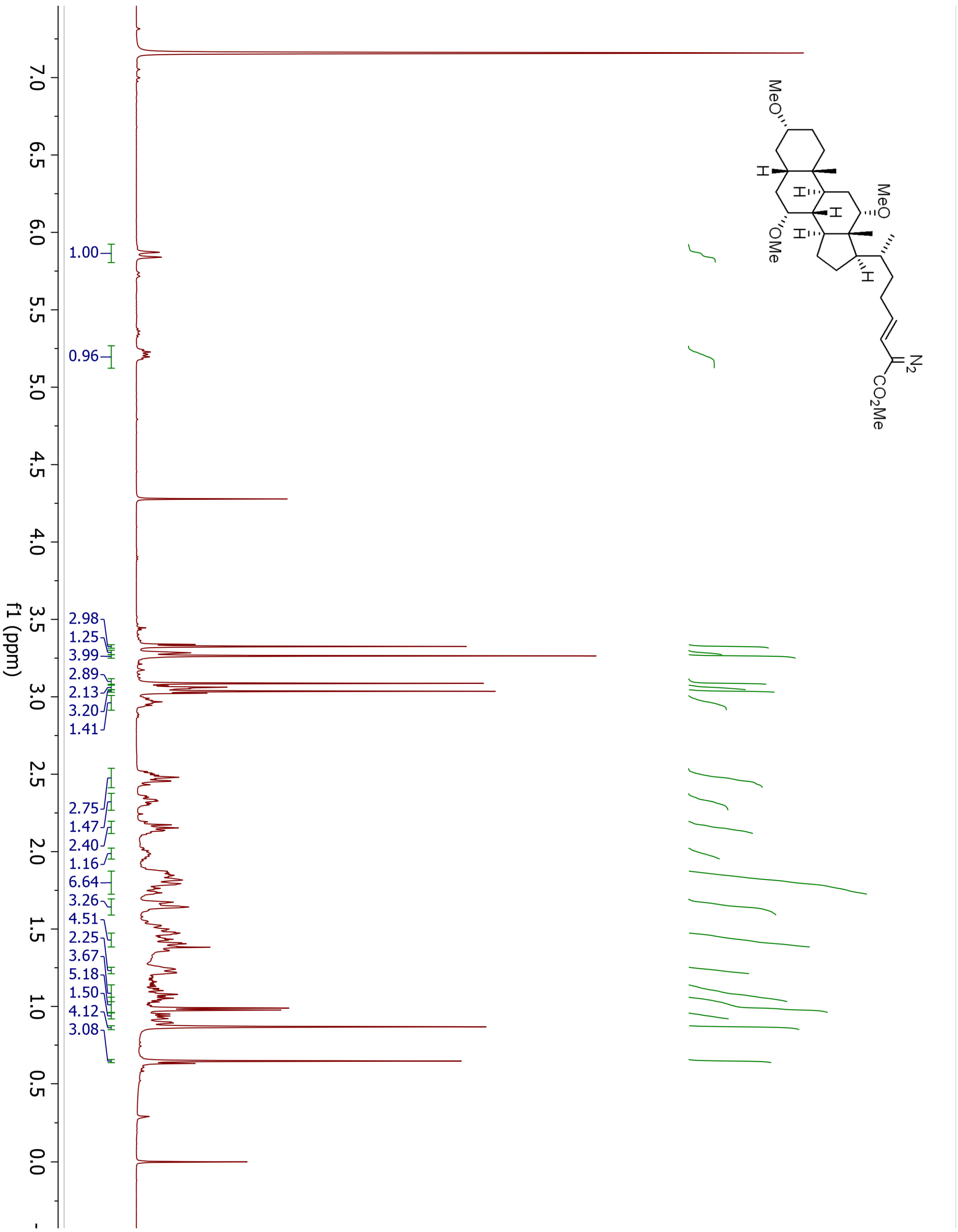

Supplementary Figure 99. ^{13}C NMR (126 MHz, C_6D_6) for compound **11b**

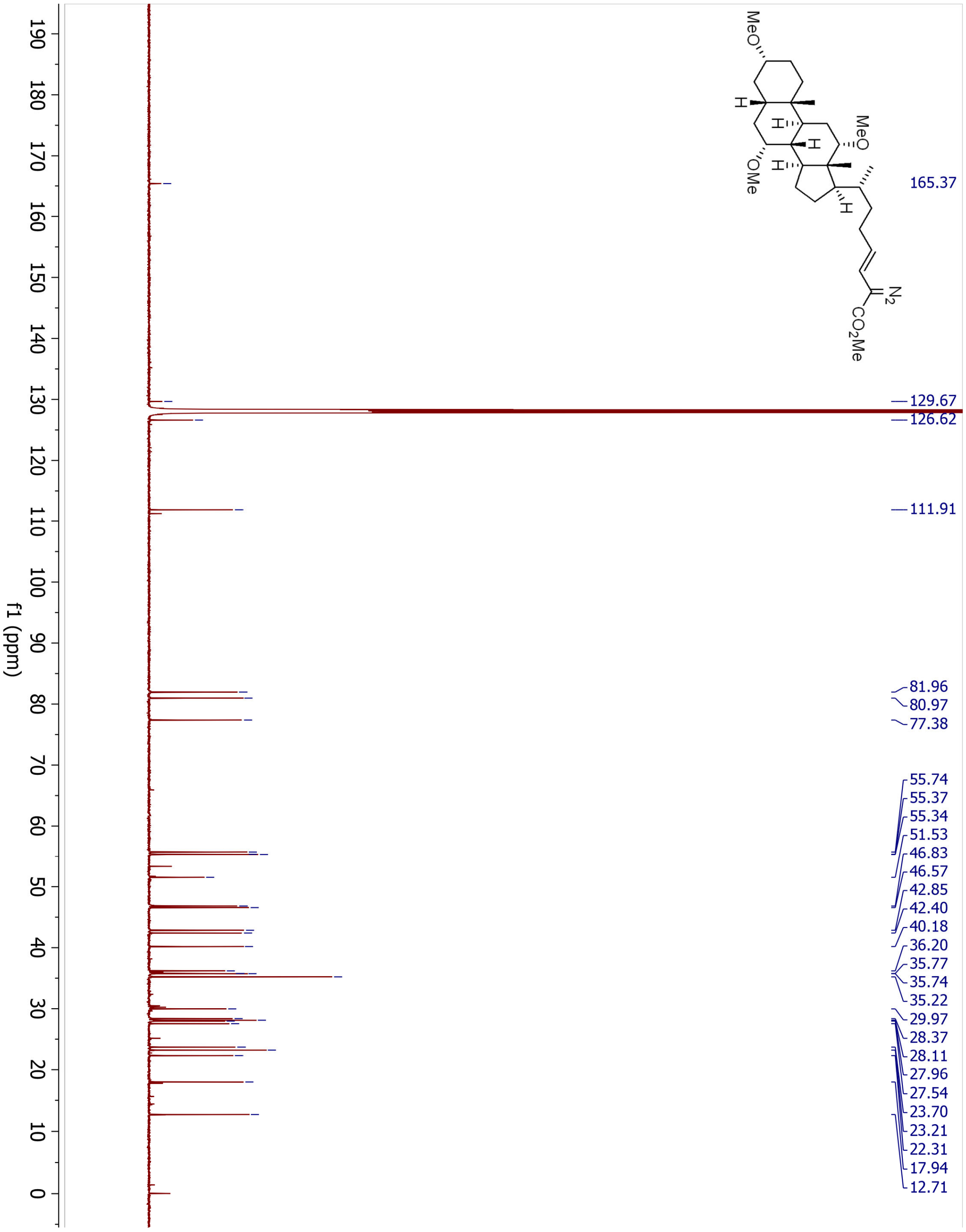

Supplementary Figure 100. ^{1}H NMR (500 MHz, CH_3CN-d_3) for **12b**

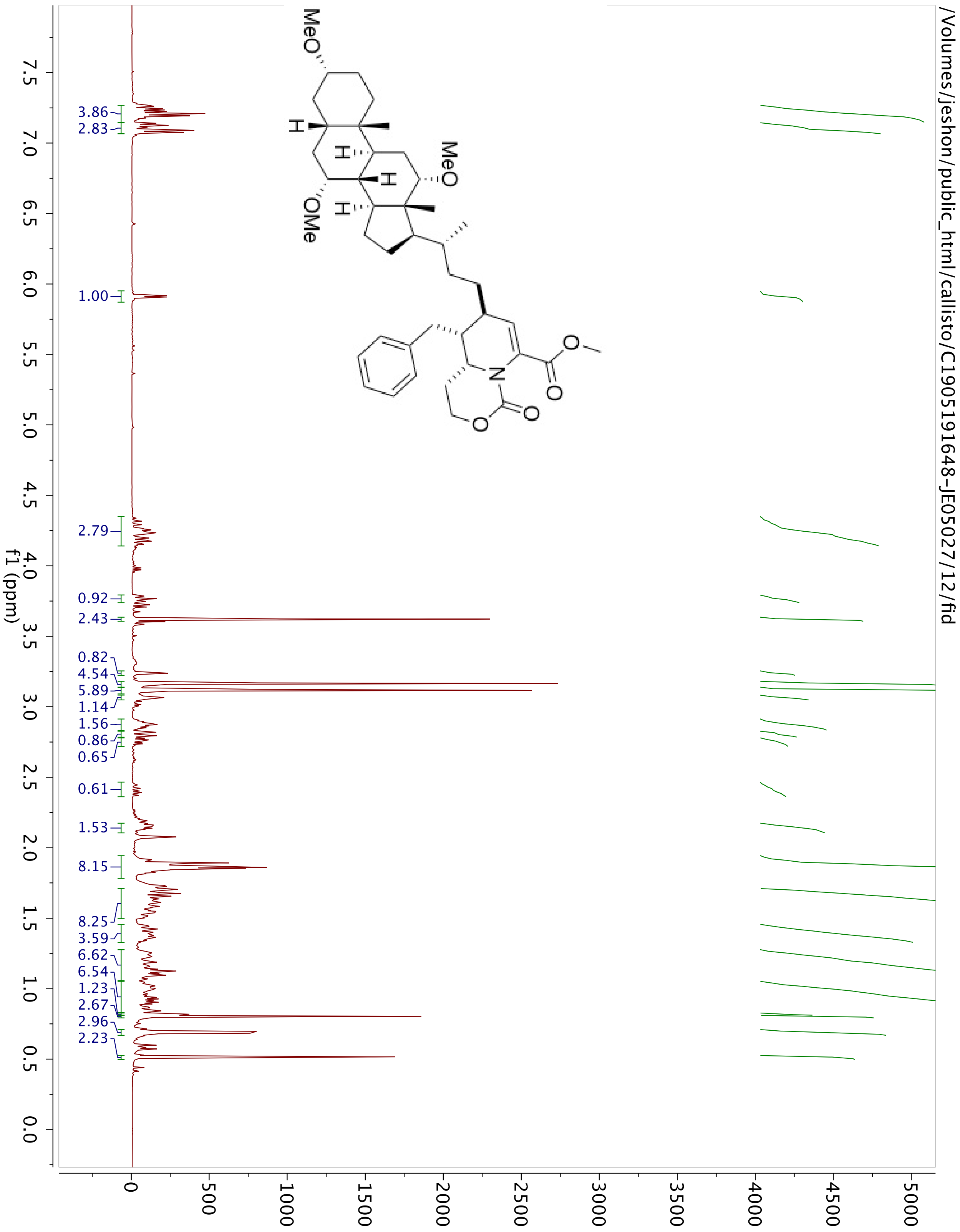

Supplementary Figure 101. ^{13}C NMR (126 MHz, CH_3CN) for compound **12b**

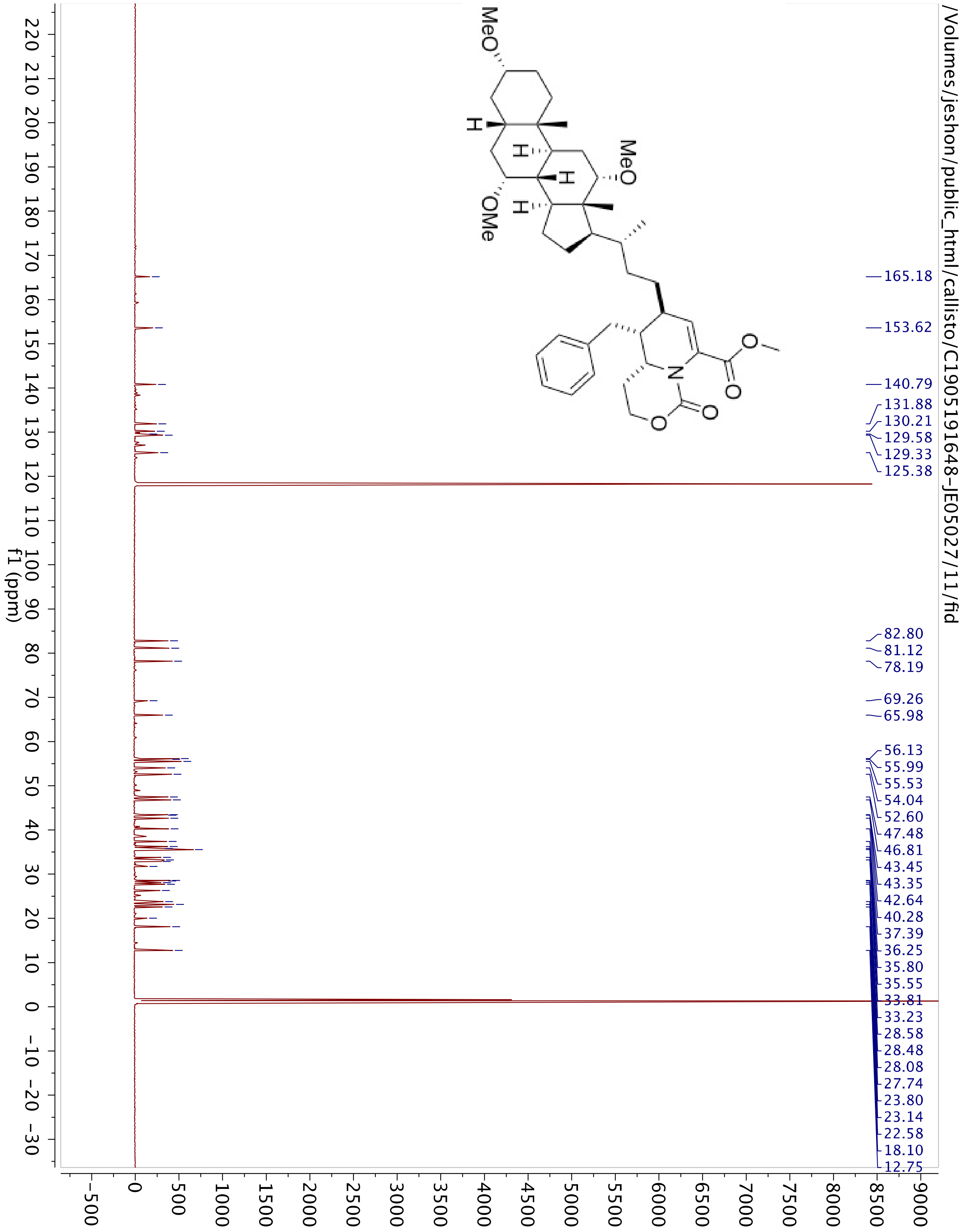

Supplementary References

1. Armarego, W.L.F. & Chai, C.L.L. Purification of Laboratory Chemicals 6th ed., Elsevier: Burlington, MA (2009).
2. Still, W. C., Kahn, M. & Mitra, A. Rapid chromatographic technique for preparative separations with moderate resolution. *J. Org. Chem.* **43**, 2923-2925 (1978).
3. Ju, M., Weatherly, C. D., Guzei, I. A. & Schomaker, J. M. Chemo- and Enantioselective Intramolecular Silver-catalyzed Aziridinations. *Angew. Chem. Int. Ed*. **56**, 9944-9948 (2017).
4. Lebel, H., Lectard, S. & Parmentier, M. Copper-Catalyzed Alkene Aziridination with N-Tosyloxycarbamates. *Org. Lett.* **9**, 4797-4800 (2007).
5. Lebel, H., Huard, K. & Lectard, S. *N*-Tosyloxycarbamates as a Source of Metal Nitrenes: Rhodium-Catalyzed C−H Insertion and Aziridination Reactions. *J. Am. Chem. Soc.* **127**, 141989-14199 (2005).
6. Manning, J. R. & Davies, H. M. L. One-flask synthesis of methyl arylvinyldiazoacetates and their application in enantioselective C–H functionalization: synthesis of (E)-methyl 2-diazo-4-phenylbut-3-enoate and (S,E)methyl 2-((R)-4-methyl-1,2-dihydronaphthalen-2-YL)-4-phenylbut-3-enoate. *Org. Synth.* **84**, 334-346 (2007).
7. Walji, A. M. & Davies, H. M. L. Direct Synthesis of (+)-Erogorgiaene through a Kinetic Enantiodifferentiating Step. *Angew. Chem. Int. Ed.* **44**, 1733-1735 (2005).
8. Johnson, R. L. Inhibition of renin by substrate analog inhibitors containing the olefinic amino acid 5 (S)-amino-7-methyl-3 (E)-octenoic acid. *J. Med. Chem*. **27**, 1351-1354 (1984).
9. Hudspeth, J. P.; Lunney, E. A.; Michniewicz, E. D.; Repine, J. T.; Roark, W. H.; Stier, M. A.; Tinney, F. J.; Woo, P. K. W.; Essenburg, A. D.; Kaltenbronn J. S. Renin inhibitors containing isosteric replacements of the amide bond connecting the P3 and P2 sites. *J. Med. Chem.* **33**, 838-845 (1990).
10. Jenmalm, A.; Berts, W.; Li, Y. L.; Luthman, K.; Csöregh, I.; Hacksell, U. Stereoselective Epoxidation of Phe-Gly and Phe-Phe Vinyl Isosteres. *J. Org. Chem.* **59**, 1139-1148 (1994).
11. Bruker-AXS (2016). *APEX3.* Version 2016.5-0. Madison, Wisconsin, USA.
12. Krause, L., Herbst-Irmer, R., Sheldrick, G. M. & Stalke, D. Comparison of Silver and Molybdenum Microfocus X-ray Sources for Single-Crystal Structure Determination. *J. Appl. Cryst.* **48***,* 3-10 (2015).
13. Sheldrick, G. M. (2013b). *XPREP*. Version 2013/1. Georg-August-Universität Göttingen, Göttingen, Germany.
14. Sheldrick, G. M. (2013a). The *SHELX* homepage, http://shelx.uni-ac.gwdg.de/SHELX/.

15. Sheldrick, G. M. SHELXT – Integrated space-group and crystal-structure determination. *Acta Cryst. A* **71**, 3-8 (2015a).
16. Sheldrick, G. M. Crystal structure refinement with SHELXL. *Acta Cryst. C* **71**, 3-8 (2015b).
17. Dolomanov, O. V., Bourhis, L. J., Gildea, R. J., Howard, J. A. K. & Puschmann, H. OLEX2: a complete structure solution, refinement and analysis program. *J. Appl. Crystallogr.* **42**, 339-341 (2009).
18. Guzei, I. A. (2007-2013). Programs *Gn*. University of Wisconsin-Madison, Madison, Wisconsin, USA.
19. Gaussian 09, Revision D.01, Frisch, M. J.; Trucks, G. W.; Schlegel, H. B.; Scuseria, G. E.; Robb, M. A.; Cheeseman, J. R.; Scalmani, G.; Barone, V.; Mennucci, B.; Petersson, G. A.; Nakatsuji, H.; Caricato, M.; Li, X.; Hratchian, H. P.; Izmaylov, A. F.; Bloino, J.; Zheng, G.; Sonnenberg, J. L.; Hada, M.; Ehara, M.; Toyota, K.; Fukuda, R.; Hasegawa, J.; Ishida, M.; Nakajima, T.; Honda, Y.; Kitao, O.; Nakai, H.; Vreven, T.; Montgomery, J. A., Jr.; Peralta, J. E.; Ogliaro, F.; Bearpark, M.; Heyd, J. J.; Brothers, E.; Kudin, K. N.; Staroverov, V. N.; Kobayashi, R.; Normand, J.; Raghavachari, K.; Rendell, A.; Burant, J. C.; Iyengar, S. S.; Tomasi, J.; Cossi, M.; Rega, N.; Millam, J. M.; Klene, M.; Knox, J. E.; Cross, J. B.; Bakken, V.; Adamo, C.; Jaramillo, J.; Gomperts, R.; Stratmann, R. E.; Yazyev, O.; Austin, A. J.; Cammi, R.; Pomelli, C.; Ochterski, J. W.; Martin, R. L.; Morokuma, K.; Zakrzewski, V. G.; Voth, G. A.; Salvador, P.; Dannenberg, J. J.; Dapprich, S.; Daniels, A. D.; Farkas, Ö.; Foresman, J. B.; Ortiz, J. V.; Cioslowski, J.; Fox, D. J. Gaussian, Inc., Wallingford CT, 2009.
20. Becke, A. D. Becke's three parameter hybrid method using the LYP correlation functional. *J. Chem. Phys*. **98**, 5648-5652 (1993).
21. Lee, C.; Yang, W.; Parr, R. G. Development of the Colle-Salvetti correlation-energy formula into a functional of the electron density. *Phys. Rev. B.* **37**, 785-789 (1998).
22. Vosko, S. H.; Wilk, L.; Nusair, M. Accurate spin-dependent electron liquid correlation energies for local spin density calculations: a critical analysis. *Can. J. Phys*. **58**, 1200-1211 (1980).
23. Grimme, S.; Antony, J.; Ehrlich, S.; Krieg, H. A consistent and accurate ab initio parametrization of density functional dispersion correction (DFT-D) for the 94 elements H-Pu. *J. Chem. Phys*. **132**, 154104-154123 (2010).
24. Weigend, F.; Ahlrichs, R. Balanced basis sets of split valence, triple zeta valence and quadruple zeta valence quality for H to Rn: Design and assessment of accuracy. *Phys. Chem. Chem. Phys*. **7**, 3297-3305 (2005).
25. McIver, J. W.; Komornicki, A. K. Structure of transition states in organic reactions. General theory and an application to the cyclobutene-butadiene

isomerization using a semiempirical molecular orbital method. *J. Am. Chem. Soc.* **94**, 2625-2633 (1972).

26. González, C.; Schlegel, H. B. Reaction path following in mass-weighted internal coordinates. *J. Phys. Chem*. **94**, 5523-5527 (1990).
27. Chu, L.; Ohta, C.; Zuo, Z.; MacMillan, D. W. C. Carboxylic acids as a traceless activation group for conjugate additions: a three-step synthesis of (±)-pregabalin. *J. Am. Chem. Soc*. **136**, 10886-10889 (2014).
