# Supplementary material for: Intermolecular [3+3] ring expansion of aziridines to dehydropiperi-dines through the intermediacy of aziridinium ylides
Source: Nat Commun. 2020 Mar 9;11:1273. doi: 10.1038/s41467-020-15134-x (PMC7062875; doi:10.1038/s41467-020-15134-x)
Supplement: Supplementary file 2 — Description of Additional Supplementary Files [file 41467_2020_15134_MOESM2_ESM.pdf]

Description of Additional Supplementary Files

File Name: Supplementary Data 1

Description: Intermolecular [3+3] Ring-Expansion of Aziridines to Dehydropiperidines through the Intermediacy of Aziridinium Ylides
