# Supplementary material for: Intermolecular [3+3] ring expansion of aziridines to dehydropiperi-dines through the intermediacy of aziridinium ylides
Source: Nat Commun. 2020 Mar 9;11:1273. doi: 10.1038/s41467-020-15134-x (PMC7062875; doi:10.1038/s41467-020-15134-x)
Supplement: Supplementary file 3 — Supplementary Data 1 [file 41467_2020_15134_MOESM3_ESM.pdf]

Supplementary Data File 1

2b, E = -439.426005

N	0.262344000	-0.539114000	-0.948473000
C	0.848806000	0.795236000	-0.891354000
C	1.583461000	-0.383547000	-0.320214000
H	1.209079000	1.138283000	-1.868937000
C	0.104868000	1.866386000	-0.112266000
C	-1.045236000	1.299079000	0.724484000
O	-1.687216000	0.161318000	0.109043000
C	-0.886730000	-0.877142000	-0.241211000
O	-1.217593000	-2.017801000	-0.020856000
H	-0.707954000	0.995664000	1.728914000
H	-1.838113000	2.046650000	0.858032000
H	0.784812000	2.436048000	0.541734000
H	-0.304495000	2.575161000	-0.847857000
H	2.392151000	-0.783004000	-0.945054000
C	1.781988000	-0.639707000	1.151603000
H	1.020096000	-0.160260000	1.780849000
H	1.754373000	-1.722168000	1.356808000
H	2.769158000	-0.262542000	1.463080000

3a-Rh$_2$, E = -1711.649206

Rh	0.466384000	0.261091000	0.002211000
C	-0.921406000	1.726662000	0.051352000
Rh	2.143460000	-1.548448000	0.016834000
O	1.031665000	-2.429618000	1.518524000
C	-0.036508000	-1.887091000	1.915309000
O	-0.512866000	-0.786395000	1.481126000
O	3.171180000	-0.438473000	1.423076000
C	2.706427000	0.668584000	1.816805000
O	1.627105000	1.207294000	1.408682000
O	0.973308000	-2.511985000	-1.387086000
C	-0.085317000	-1.967712000	-1.805624000
O	-0.535128000	-0.835002000	-1.427935000
O	3.115392000	-0.531635000	-1.492077000
C	2.643516000	0.563299000	-1.913119000
O	1.589194000	1.132230000	-1.484998000
C	3.362053000	1.245204000	-3.049706000
H	4.435006000	1.011529000	-3.021870000
H	2.949865000	0.863075000	-3.998852000
H	3.199433000	2.330865000	-3.016806000
C	-0.893070000	-2.682607000	-2.857986000
H	-0.760636000	-2.158675000	-3.818966000
H	-0.562613000	-3.723527000	-2.965897000
H	-1.962635000	-2.642307000	-2.603889000
C	3.460634000	1.406317000	2.892893000
H	4.535678000	1.190555000	2.826800000
H	3.274389000	2.486678000	2.828429000
H	3.097061000	1.051623000	3.872175000
C	-0.821723000	-2.561953000	3.010089000
H	-0.479079000	-3.593822000	3.159316000
H	-0.680993000	-1.995072000	3.945149000
H	-1.894736000	-2.542704000	2.769068000

C	-2.321491000	1.603928000	0.055330000
H	-2.927013000	2.513946000	0.146065000
C	-2.960129000	0.378729000	-0.038198000
H	-2.329235000	-0.506859000	-0.115992000
C	-4.380833000	0.135519000	-0.044399000
C	-5.351660000	1.164610000	0.052484000
C	-4.821986000	-1.207422000	-0.147100000
C	-6.706634000	0.853875000	0.046297000
C	-6.180508000	-1.512682000	-0.153711000
C	-7.124260000	-0.482580000	-0.056288000
H	-5.039461000	2.207509000	0.132151000
H	-4.078154000	-2.005423000	-0.220092000
H	-7.448725000	1.652385000	0.121049000
H	-6.508173000	-2.551821000	-0.233512000
H	-8.191534000	-0.718927000	-0.060761000
C	-0.389108000	3.092521000	0.276553000
O	-0.409681000	3.609455000	1.375222000
O	0.132593000	3.651397000	-0.814353000
C	0.767162000	4.926551000	-0.644158000
H	0.054698000	5.674557000	-0.263200000
H	1.611817000	4.844713000	0.057102000
H	1.129000000	5.222121000	-1.636888000

TS1, E = -2151.068188

Rh	0.751513000	-0.216715000	0.158461000
C	-1.027164000	0.649616000	0.885287000
N	-1.523895000	2.220884000	-0.424767000
C	-2.890828000	2.109258000	-0.980991000
C	-1.703743000	1.783865000	-1.826935000
H	-3.409076000	1.225799000	-0.599860000
C	-3.774646000	3.344961000	-0.997127000
C	-3.035507000	4.635940000	-0.637811000
O	-1.957455000	4.389202000	0.295512000
C	-1.015887000	3.546976000	-0.189994000
O	0.107047000	3.888910000	-0.409943000
H	-2.609140000	5.129818000	-1.526774000
H	-3.703175000	5.351984000	-0.142399000
H	-4.261835000	3.458012000	-1.978707000
H	-4.571763000	3.174067000	-0.258774000
Rh	2.876961000	-1.279549000	-0.522855000
O	2.235361000	-2.966833000	0.485012000
C	1.128943000	-2.955118000	1.096373000
O	0.330430000	-1.968013000	1.166881000
O	3.694027000	-0.537550000	1.220959000
C	2.992663000	0.189539000	1.980636000
O	1.773341000	0.498321000	1.793330000
O	1.899645000	-1.936229000	-2.223234000
C	0.674506000	-1.683640000	-2.383180000
O	-0.057490000	-1.022394000	-1.575310000
O	3.344325000	0.492887000	-1.477729000
C	2.479400000	1.417636000	-1.514619000
O	1.335789000	1.384499000	-0.967449000
C	2.806334000	2.660595000	-2.303426000
H	3.883916000	2.871794000	-2.259666000

H	2.537919000	2.484022000	-3.358601000
H	2.220061000	3.508137000	-1.926599000
C	-0.011308000	-2.204230000	-3.621242000
H	0.704223000	-2.695975000	-4.292189000
H	-0.792671000	-2.922520000	-3.324958000
H	-0.509370000	-1.373212000	-4.144154000
C	3.642102000	0.772246000	3.210824000
H	3.776947000	1.855669000	3.059049000
H	2.983185000	0.636094000	4.081030000
H	4.619894000	0.309263000	3.396104000
C	0.700134000	-4.206421000	1.821530000
H	-0.334323000	-4.458556000	1.542840000
H	1.371071000	-5.044927000	1.595092000
H	0.709744000	-4.011528000	2.906129000
H	-1.489243000	0.714497000	-1.919776000
C	-1.179634000	2.606188000	-2.972941000
H	-1.459241000	3.667613000	-2.919074000
H	-0.083193000	2.534257000	-3.001025000
H	-1.580534000	2.196362000	-3.913319000
C	-2.282024000	-0.082158000	0.927701000
H	-3.034206000	0.268254000	1.644614000
C	-2.543873000	-1.193977000	0.190363000
H	-1.768900000	-1.545543000	-0.491170000
C	-3.761981000	-2.000171000	0.223412000
C	-4.912587000	-1.648339000	0.964975000
C	-3.787415000	-3.199346000	-0.521611000
C	-6.037882000	-2.470069000	0.961950000
C	-4.914457000	-4.022725000	-0.521642000
C	-6.043439000	-3.660670000	0.221010000
H	-4.928167000	-0.722641000	1.543911000
H	-2.904363000	-3.483497000	-1.100597000
H	-6.919996000	-2.183650000	1.540526000
H	-4.913009000	-4.949145000	-1.101581000
H	-6.928567000	-4.302189000	0.222599000
C	-0.790397000	1.384307000	2.167061000
O	-0.989710000	0.839408000	3.236025000
O	-0.289104000	2.611505000	2.055892000
C	0.088708000	3.279835000	3.259600000
H	-0.770083000	3.382873000	3.940903000
H	0.891331000	2.725976000	3.769351000
H	0.448751000	4.270305000	2.953378000

INT1, E = -2151.083394

Rh	0.552639000	-0.412020000	0.176635000
C	-0.583800000	1.501855000	0.728913000
N	-0.162627000	2.592852000	-0.234180000
C	-1.188247000	3.513646000	-0.869402000
C	-0.540403000	2.477203000	-1.710173000
H	-2.189328000	3.267914000	-0.512371000
C	-0.886604000	4.983397000	-0.817794000
C	0.560200000	5.284100000	-1.120845000
O	1.450480000	4.377797000	-0.421308000
C	1.184351000	3.185586000	0.082552000
O	1.966471000	2.596099000	0.755490000
H	0.786661000	5.206420000	-2.192743000
H	0.850277000	6.286621000	-0.780942000

H	-1.531863000	5.510099000	-1.538200000
H	-1.151942000	5.339531000	0.189610000
Rh	1.714589000	-2.472174000	-0.501687000
O	0.371814000	-3.518113000	0.663735000
C	-0.510636000	-2.889214000	1.318438000
O	-0.657759000	-1.628794000	1.339903000
O	2.923721000	-2.206841000	1.146924000
C	2.749027000	-1.201903000	1.901431000
O	1.876073000	-0.298451000	1.744489000
O	0.404187000	-2.581605000	-2.103474000
C	-0.481758000	-1.691652000	-2.249668000
O	-0.654832000	-0.689080000	-1.488298000
O	2.981486000	-1.287218000	-1.619035000
C	2.773568000	-0.040672000	-1.681622000
O	1.842908000	0.583601000	-1.086467000
C	3.683382000	0.777693000	-2.564559000
H	4.661978000	0.289764000	-2.667234000
H	3.229918000	0.853791000	-3.566949000
H	3.800462000	1.792938000	-2.161581000
C	-1.442325000	-1.819628000	-3.406019000
H	-1.466110000	-0.877909000	-3.975371000
H	-1.164470000	-2.652246000	-4.064769000
H	-2.455482000	-1.986608000	-3.005687000
C	3.641467000	-1.093329000	3.113820000
H	4.667979000	-1.393024000	2.858028000
H	3.627865000	-0.074023000	3.521184000
H	3.269211000	-1.788962000	3.884398000
C	-1.503364000	-3.702825000	2.111259000
H	-1.078884000	-4.678563000	2.383026000
H	-1.821295000	-3.155787000	3.009224000
H	-2.391930000	-3.872892000	1.480556000
H	-1.128680000	1.567934000	-1.832385000
C	0.461436000	2.629394000	-2.827099000
H	1.361447000	3.199562000	-2.574578000
H	0.779245000	1.630159000	-3.145895000
H	-0.041370000	3.127324000	-3.671115000
C	-1.931293000	0.915111000	0.652338000
H	-2.076395000	0.328660000	1.562499000
C	-2.940975000	0.853496000	-0.250715000
H	-2.935223000	1.422911000	-1.180954000
C	-4.112281000	-0.021578000	-0.102729000
C	-4.131520000	-1.134461000	0.769784000
C	-5.268256000	0.221668000	-0.877008000
C	-5.263477000	-1.943290000	0.878471000
C	-6.399279000	-0.591465000	-0.769520000
C	-6.407478000	-1.676982000	0.113926000
H	-3.234374000	-1.383684000	1.339708000
H	-5.275269000	1.067432000	-1.571563000
H	-5.248587000	-2.799772000	1.558902000
H	-7.281018000	-0.375420000	-1.379716000
H	-7.290622000	-2.315846000	0.197911000
C	-0.379112000	1.978330000	2.142929000
O	-0.403124000	1.278684000	3.126474000
O	-0.261440000	3.329113000	2.218598000
C	0.043413000	3.879789000	3.503253000
H	-0.737641000	3.622751000	4.234574000

H	1.012878000	3.504736000	3.866472000
H	0.089622000	4.967846000	3.366547000

INT2, E = -1015.282384

C	0.551999000	0.900073000	-0.188694000
N	1.291218000	-0.356331000	-0.158447000
C	2.223648000	-0.664503000	-1.321619000
C	0.965874000	-1.429910000	-1.225566000
H	2.198205000	0.162463000	-2.033650000
C	3.612563000	-1.103175000	-0.956863000
C	3.646624000	-2.026905000	0.238429000
O	2.782986000	-1.564965000	1.310112000
C	1.696346000	-0.823849000	1.203292000
O	1.043180000	-0.500669000	2.147294000
H	3.339366000	-3.050826000	-0.013497000
H	4.647945000	-2.072080000	0.684433000
H	4.070632000	-1.609231000	-1.820943000
H	4.193493000	-0.192347000	-0.751386000
H	0.173093000	-1.029440000	-1.861345000
C	0.748594000	-2.868744000	-0.838433000
H	1.278721000	-3.187546000	0.066573000
H	-0.324046000	-3.048874000	-0.682824000
H	1.084657000	-3.505399000	-1.671955000
C	-0.863137000	0.908104000	-0.139037000
C	1.334374000	2.093090000	-0.089885000
O	0.920973000	3.246893000	-0.104707000
O	2.682470000	1.819123000	0.040120000
C	3.544891000	2.947220000	0.129306000
H	3.500585000	3.561379000	-0.785312000
H	3.285076000	3.586415000	0.988339000
H	4.562280000	2.552326000	0.257952000
C	-1.734808000	-0.138431000	0.011588000
H	-1.333163000	-1.138843000	0.196350000
C	-3.190670000	-0.055663000	0.005372000
C	-3.914857000	1.132128000	-0.271732000
C	-3.954290000	-1.216366000	0.287249000
C	-5.309094000	1.153775000	-0.253648000
H	-3.374801000	2.052438000	-0.507477000
C	-5.349877000	-1.192705000	0.301150000
H	-3.429431000	-2.152546000	0.503079000
C	-6.043444000	-0.006344000	0.032237000
H	-5.833000000	2.089839000	-0.469969000
H	-5.902293000	-2.110502000	0.524686000
H	-7.136351000	0.015152000	0.042068000
H	-1.264791000	1.922108000	-0.237432000

TS2, E = -1015.264461

C	0.442061000	0.843680000	0.190551000
N	1.104025000	-0.324226000	0.333571000
C	2.050918000	-1.167322000	-1.657013000
C	0.923963000	-1.546818000	-0.883962000
H	1.883321000	-0.345410000	-2.358742000
C	3.471498000	-1.503649000	-1.386633000
C	3.693714000	-2.097614000	-0.011546000

O	3.171297000	-1.312560000	1.080461000
C	2.206744000	-0.389707000	1.215314000
O	2.240222000	0.323280000	2.192791000
H	3.269710000	-3.108344000	0.059546000
H	4.769312000	-2.177926000	0.201629000
H	3.874887000	-2.227612000	-2.124650000
H	4.082177000	-0.591001000	-1.507827000
H	-0.012676000	-1.205290000	-1.336901000
C	0.716348000	-2.878577000	-0.180600000
H	1.363085000	-3.021901000	0.693146000
H	-0.322797000	-2.952922000	0.169553000
H	0.897401000	-3.706453000	-0.883566000
C	-0.980545000	0.909194000	0.063915000
C	1.203112000	2.095850000	-0.001919000
O	0.737889000	3.216541000	0.088964000
O	2.489292000	1.859662000	-0.340034000
C	3.341977000	2.999373000	-0.453981000
H	2.963147000	3.705621000	-1.209149000
H	3.422742000	3.523149000	0.511802000
H	4.326450000	2.618944000	-0.755477000
C	-1.850812000	-0.119527000	0.293201000
H	-1.446367000	-1.060653000	0.676554000
C	-3.298131000	-0.089919000	0.117269000
C	-3.994310000	1.017967000	-0.423566000
C	-4.058093000	-1.219931000	0.500004000
C	-5.380657000	0.994624000	-0.562022000
H	-3.442299000	1.904892000	-0.742271000
C	-5.446467000	-1.241501000	0.358033000
H	-3.541969000	-2.088941000	0.918664000
C	-6.117039000	-0.133815000	-0.173232000
H	-5.894889000	1.863626000	-0.981748000
H	-6.009224000	-2.127964000	0.663349000
H	-7.204189000	-0.148046000	-0.286230000
H	-1.361280000	1.886581000	-0.243028000

TS2', E = -1015.274842

C	0.670116000	0.994794000	0.080088000
N	1.337729000	-0.248494000	0.065774000
C	2.178138000	-0.643629000	-1.133166000
C	0.954988000	-1.380866000	-1.458926000
H	2.335631000	0.243951000	-1.749581000
C	3.494612000	-1.261303000	-0.730051000
C	3.327378000	-2.348307000	0.309574000
O	2.311939000	-2.030058000	1.291233000
C	1.455428000	-1.006110000	1.275555000
O	0.808037000	-0.739566000	2.250634000
H	3.042650000	-3.310973000	-0.135350000
H	4.254033000	-2.503100000	0.879827000
H	4.003647000	-1.670628000	-1.615807000
H	4.110009000	-0.439891000	-0.333927000
H	0.196539000	-0.776126000	-1.961278000
C	0.649842000	-2.821187000	-1.259263000
H	-0.357820000	-3.050491000	-1.629772000
H	1.386885000	-3.445732000	-1.795528000

H	0.694095000	-3.130913000	-0.201240000
C	-0.746778000	1.042578000	0.102383000
C	1.456695000	2.199013000	-0.035771000
O	1.019668000	3.341862000	-0.077500000
O	2.805418000	1.949683000	-0.079504000
C	3.650052000	3.092232000	-0.179202000
H	3.440603000	3.671552000	-1.093234000
H	3.524245000	3.759022000	0.688924000
H	4.681199000	2.714443000	-0.209273000
C	-1.607419000	-0.024393000	0.136666000
H	-1.192320000	-1.029011000	0.254013000
C	-3.062390000	0.034998000	0.053388000
C	-3.798257000	1.242609000	-0.047982000
C	-3.807007000	-1.170233000	0.070403000
C	-5.190248000	1.238243000	-0.127071000
H	-3.270822000	2.199358000	-0.064071000
C	-5.200546000	-1.172142000	-0.011729000
H	-3.270447000	-2.120758000	0.150927000
C	-5.906576000	0.032672000	-0.110882000
H	-5.725918000	2.189207000	-0.202873000
H	-5.740760000	-2.123378000	0.004321000
H	-6.997920000	0.034600000	-0.173527000
H	-1.146305000	2.059141000	0.044034000

INT3, E = -1015.335060

N	1.270914000	-0.066270000	-1.148547000
C	1.522818000	1.857512000	1.286335000
C	0.275738000	1.951571000	1.776471000
H	2.092391000	0.952971000	1.524670000
C	2.228135000	2.851299000	0.401651000
C	2.298418000	2.459541000	-1.075763000
O	3.123998000	1.299340000	-1.310045000
C	2.639979000	0.051498000	-1.378632000
O	3.368593000	-0.863521000	-1.700042000
H	1.293637000	2.293973000	-1.489580000
H	2.788671000	3.261616000	-1.647406000
H	1.737500000	3.837263000	0.437841000
H	3.261841000	2.996752000	0.761306000
H	-0.081504000	1.121437000	2.399554000
C	-0.717641000	3.056192000	1.564732000
H	-0.341017000	3.861867000	0.917427000
H	-1.642095000	2.660629000	1.107045000
H	-1.022193000	3.504725000	2.527472000
C	-1.553048000	-0.270349000	-0.580552000
H	-1.168506000	0.654666000	-1.022115000
C	-0.643790000	-1.222734000	-0.254232000
H	-0.939502000	-2.158184000	0.225142000
C	0.785016000	-1.027755000	-0.444357000
C	-2.997730000	-0.320979000	-0.383072000
C	-3.664667000	-1.441314000	0.162803000
C	-3.766045000	0.807349000	-0.744513000
C	-5.046000000	-1.426221000	0.340003000
C	-5.150396000	0.820236000	-0.565132000

C	-5.794249000	-0.296204000	-0.021414000
H	-3.098160000	-2.329763000	0.449202000
H	-3.262797000	1.679218000	-1.171473000
H	-5.547188000	-2.300240000	0.763807000
H	-5.728532000	1.703135000	-0.849405000
H	-6.877974000	-0.289440000	0.121463000
C	1.665738000	-2.051267000	0.262277000
O	1.510214000	-3.241337000	0.134374000
O	2.571429000	-1.470335000	1.044027000
C	3.534939000	-2.329356000	1.675408000
H	3.032620000	-3.090924000	2.290407000
H	4.154229000	-2.824454000	0.912330000
H	4.155003000	-1.678645000	2.303556000

TS3, E = -1015.302020

N	1.396743000	0.390498000	-0.582141000
C	0.650374000	1.368615000	1.206916000
C	-0.753846000	1.257546000	1.183544000
H	1.184648000	0.596508000	1.762839000
C	1.385591000	2.680483000	1.057392000
C	1.933291000	2.954170000	-0.341072000
O	2.972050000	2.044352000	-0.712378000
C	2.680252000	0.743414000	-0.931084000
O	3.507938000	0.029155000	-1.464526000
H	1.126355000	2.923229000	-1.090743000
H	2.402804000	3.947495000	-0.372293000
H	0.733574000	3.528137000	1.322680000
H	2.234626000	2.693644000	1.759300000
H	-1.177472000	0.515965000	1.864903000
C	-1.625171000	2.445519000	0.871864000
H	-1.300815000	2.967764000	-0.042347000
H	-2.676300000	2.152224000	0.743438000
H	-1.593236000	3.179025000	1.698092000
C	-1.267377000	-0.145520000	-0.462806000
H	-0.982349000	0.684545000	-1.112439000
C	-0.320836000	-1.184121000	-0.349221000
H	-0.622170000	-2.191779000	-0.055758000
C	1.034781000	-0.888841000	-0.442555000
C	-2.713802000	-0.376652000	-0.314289000
C	-3.228082000	-1.344653000	0.572570000
C	-3.622824000	0.400675000	-1.059970000
C	-4.604380000	-1.537395000	0.695950000
C	-5.000626000	0.204074000	-0.939139000
C	-5.496024000	-0.765788000	-0.060916000
H	-2.543376000	-1.941303000	1.180088000
H	-3.237860000	1.160160000	-1.745471000
H	-4.986257000	-2.290842000	1.389882000
H	-5.690400000	0.811429000	-1.531051000
H	-6.573981000	-0.918316000	0.037957000
C	2.025633000	-1.976257000	-0.119116000
O	2.007370000	-3.084277000	-0.603088000
O	2.885644000	-1.576595000	0.825423000
C	3.960555000	-2.468783000	1.137390000
H	3.579868000	-3.439813000	1.489967000

H	4.592689000	-2.629465000	0.250253000
H	4.543373000	-1.982097000	1.929491000

4ba, E = -1015.378313

N	-1.268351000	-0.432082000	0.140358000
C	-0.129717000	-1.241923000	0.619677000
C	0.799833000	-1.563093000	-0.574881000
H	0.446858000	-0.588142000	1.292481000
C	-0.632295000	-2.414720000	1.462066000
C	-1.900851000	-3.023850000	0.902803000
O	-2.898633000	-2.015445000	0.664663000
C	-2.580648000	-0.860192000	0.052983000
O	-3.448482000	-0.221822000	-0.508748000
H	-1.727643000	-3.564149000	-0.039511000
H	-2.356533000	-3.724856000	1.615397000
H	0.150928000	-3.184101000	1.537908000
H	-0.840131000	-2.053603000	2.481742000
H	1.651093000	-2.134262000	-0.172826000
C	0.141669000	-2.390154000	-1.682417000
H	-0.761772000	-1.897765000	-2.077903000
H	0.841028000	-2.525906000	-2.522419000
H	-0.139760000	-3.393117000	-1.329684000
C	1.354484000	-0.211292000	-1.130563000
H	1.654332000	-0.385260000	-2.177900000
C	0.299624000	0.861279000	-1.141809000
H	0.553535000	1.822353000	-1.594289000
C	-0.904226000	0.743733000	-0.552306000
C	2.615875000	0.235364000	-0.393010000
C	2.577121000	1.131064000	0.687302000
C	3.859351000	-0.302480000	-0.769577000
C	3.745749000	1.471636000	1.379604000
C	5.027247000	0.035803000	-0.081646000
C	4.974582000	0.925676000	0.998432000
H	1.624285000	1.571214000	0.990696000
H	3.909095000	-0.998681000	-1.612279000
H	3.692809000	2.171327000	2.218301000
H	5.984212000	-0.392426000	-0.392551000
H	5.887949000	1.192846000	1.536642000
C	-1.817793000	1.931032000	-0.498665000
O	-1.963769000	2.721967000	-1.402769000
O	-2.370158000	2.066599000	0.713732000
C	-3.341164000	3.106035000	0.857069000
H	-2.899417000	4.093921000	0.653321000
H	-4.185376000	2.942586000	0.168915000
H	-3.691756000	3.058706000	1.896048000
